# Oxadendralenes in asymmetric organocatalysis for the construction of tetrahydroisochromenes†
†Electronic supplementary information (ESI) available: Experimental procedures and full compound characterization, including NMR data, UPC^2^ traces and crystallographic data. CCDC 1419567, 1408759, 1405309, 1405275, 1405277 and 1419566. For the ESI and crystallographic data in CIF or other electronic format see DOI: 10.1039/c6sc00185h


**DOI:** 10.1039/c6sc00185h

**Published:** 2016-02-17

**Authors:** Niels Hammer, Lars A. Leth, Julian Stiller, Magnus E. Jensen, Karl Anker Jørgensen

**Affiliations:** a Department of Chemistry , Aarhus University , DK-8000 Aarhus C , Denmark . Email: kaj@chem.au.dk

## Abstract

Oxadendralenes are integrated in a novel manner into a one-pot cascade utilizing synergistic catalysis for the construction of valuable and complex bicyclic heterocyclic scaffolds. The construction is based on the organocatalytic activation of the oxadendralenes generating a vinylogous iminium-ion intermediate which is set-up for a 1,6-addition with an enamine formed from an aldehyde and the same organocatalyst. This reaction generates a cyclic oxadendralenic intermediate, which acts as an electron-deficient heterodiene reacting in a Lewis-acid catalyzed hetero-Diels–Alder reaction with vinyl ethers to form tetrahydroisochromenes with five continuous stereocenters in high yields, >20 : 1 dr and 99% ee. This synergistic organo- and Lewis-acid catalysed system also displays high tolerance for variation in oxadendralenes and aldehydes, which provides tetrahydroisochromenes with high diversity in the substituent pattern and the same excellent stereoselectivities. Mechanistic studies have been performed to account for the activation modes and stereochemical outcome of the reaction. The reaction concept has been extended to also include a sequential organocatalytic reaction of oxadendralenes with aldehydes, in which the enamine formed from the aldehyde and the organocatalyst act both in the first catalytic cycle forming the cyclic oxadendralenic intermediate and in a second catalytic cycle leading to tetrahydroisochromenes in good yields and excellent stereoselectivities. Mechanistic studies reveal that the stereochemistry of the organocatalyst has an influence on the diastereoselectivity of the reaction sequence. Some transformations of the tetrahydroisochromenes are also presented. The chiral tetrahydroisochromenes formed might be applied in the diversified synthesis of important drugs.

## Abstract 2

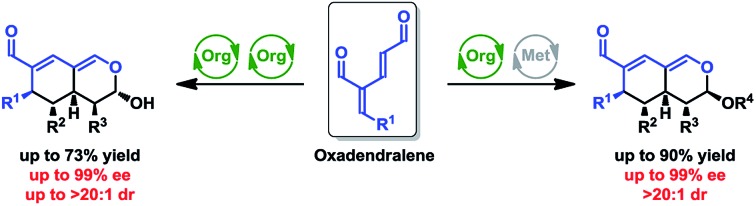
Oxadendralenes are integrated in a novel manner into a one-pot cascade utilizing synergistic catalysis for the construction of valuable and complex bicyclic heterocyclic scaffolds.

## Introduction

The diversified synthesis of intricate molecular structures is becoming an increasingly applied strategy in organic synthesis.^1^ However, gaining easy access to important molecular scaffolds while being able to vary the structural components and the stereochemical information still constitutes a challenging task.

Catalysis provides one of the most efficient ways to perform asymmetric operations and within recent years organocatalysis has evolved from being merely a proof of concept into a powerful synthetic tool for the construction of chiral compounds.^2^ This rapidly growing field now features a large number of examples displaying complex strategies aimed at the synthesis of natural compound-resembling targets, attaining high yields and stereoselectivities.^3^ The unique ability of organocatalysis to use simple starting materials to build up complex nature-inspired molecules in a “mix & hit” fashion enhances its applicability in academia and industry.^4^ In addition, organocatalytic methodologies are highly tolerable against other synthetic modifications, allowing one-pot strategies to be easily implemented, which enables the rapid synthesis of compounds with increased molecular complexity.^5^ One of the challenges remaining is the combination of metal catalysis and organocatalytic processes. Recently, the problematic compatibility of these two types of catalysis has received significant attention, as it holds the potential to facilitate unprecedented transformations.^6^

**Fig. 1:**
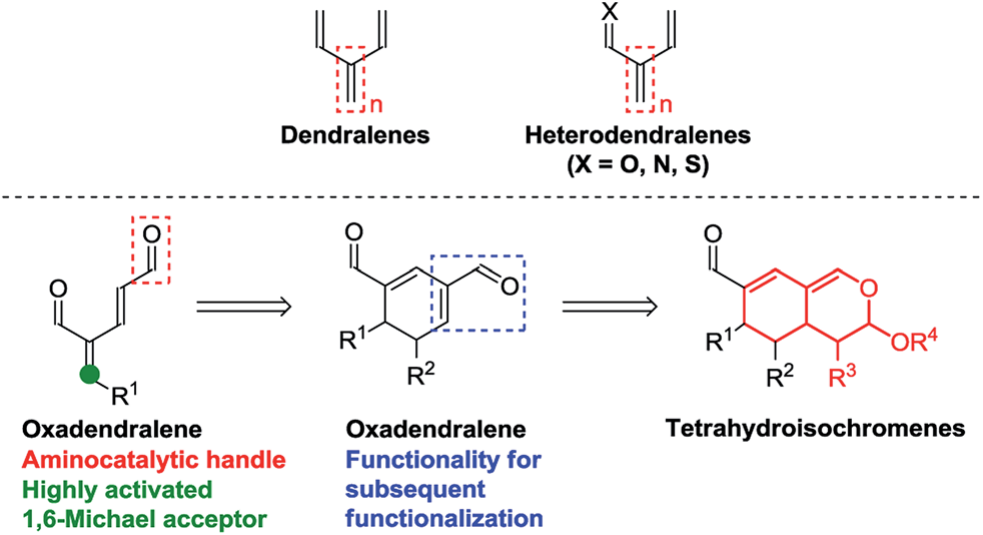
Top: Dendralenes and heterodendralenes. Bottom: Overview of the envisioned reaction sequence.

One of the most efficient synthetic routes towards attaining intricate cyclic frameworks is the application of cycloadditions. Dendralenes (Fig. 1, top) are well-known for their synthetic value in Diels–Alder reactions, as they have been shown to undergo multiple cycloadditions as part of reaction cascades with dienophiles.^7^ However, there are only a few examples featuring their heteroatom analogs (Fig. 1, top), despite the ability of heterodendralenes to facilitate hetero-Diels–Alder reactions yielding unprecedented polycyclic frameworks with exceptional atom economy.^8^

The hetero-Diels–Alder reaction in particular has been subject to a vast amount of research as it offers a simple approach for the synthesis of six-membered oxygen- and nitrogen-containing heterocycles which is of great importance in medicinal chemistry.^9^

We envisioned the possibility of designing a reaction sequence integrating oxadendralenes in a novel manner into a one-pot cascade utilizing synergistic catalysis for the construction of valuable and complex core structures. Such a construction is envisioned to proceed *via* an organocatalytic annulation sequence forming a cyclic oxadendralene (Fig. 1, bottom). Inspired by the idea of constructing heterocyclic molecular complexity through utilizing hetero-Diels–Alder reactions, we sought to incorporate the oxadendralenic intermediate into a hetero-Diels–Alder reaction to generate the bicyclic tetrahydroisochromene scaffold (Fig. 1, bottom).

**Fig. 2:**
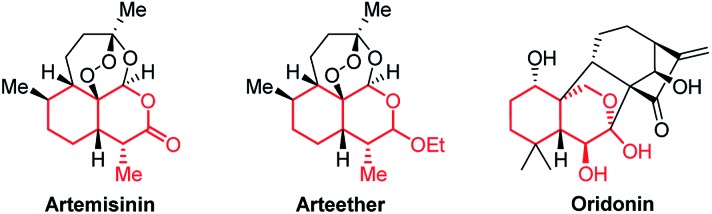
Structure of artemisinin, arteether and oridonin, containing the tetrahydroisochromene scaffold (marked in red).

Bicyclic tetrahydroisochromenes are interesting and valuable motifs as they are present in some very important natural products. For instance, they can be found in the core structure of the anti-malarial artemisinin (Fig. 2), which was recently awarded the Nobel Prize in Physiology or Medicine.^10^ Furthermore, they resemble promising intermediates towards the total synthesis of artemisinin and its analogs.^11^ As of today, artemisinin and its analogs are among the most widely used anti-malarial drugs, displaying a very high potency against the malaria-causing *Plasmodium* parasite; however, examples of resistant strains are beginning to emerge, stressing the importance of analog-development.^12^ Additionally, the anti-cancer agent oridonin also includes the tetrahydroisochromene core structure. Oridonin has been found to exhibit a broad spectrum of remarkable anti-cancer and anti-bacterial properties, such as displaying selective induction of apoptosis in leukemia cells and general anti-proliferative activity in tumors.^13^

Herein, we present two novel organocatalysis-initiated strategies, enabling the enantioselective formation of a comprehensive tetrahydroisochromene library with full stereocontrol of all sp^3^-carbon centers (five stereocenters). The two-step one-pot cascades employ novel oxadendralenes in a sequential catalysis system, giving rise to the products in excellent enantio- and diastereoselectivities.

### Strategic design

**Scheme 1:**
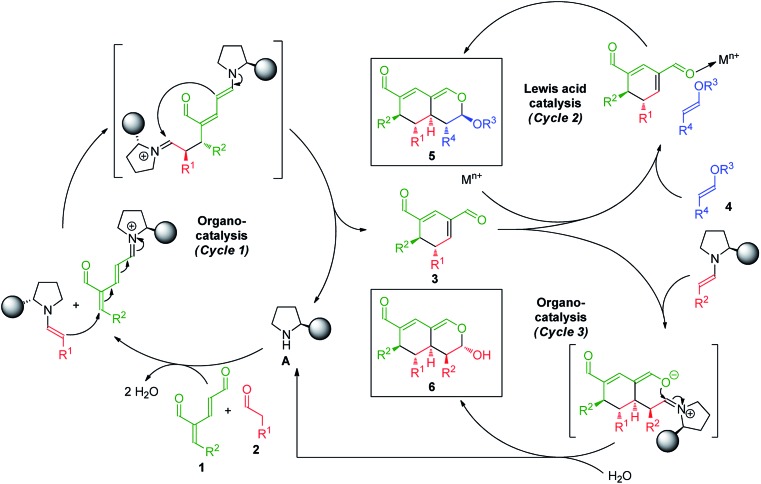
Synthetic strategy integrating oxadendralene **1** for the construction of the bicyclic tetrahydroisochromenes **5** and **6***via* a combination of organocatalysis and Lewis-acid catalysis (cycles 1 and 2) or sequential organocatalysis (cycles 1 and 3).

The synthetic strategy integrating oxadendralenes for the enantioselective construction of bicyclic tetrahydroisochromenes is presented in Scheme 1. We envisioned that the activation of oxadendralenic dienal **1** by an organocatalyst **A** would generate a vinylogous iminium-ion set-up for a 1,6-addition reaction^14^ with an enamine formed from an aldehyde **2** and the same organocatalyst. Thus, we assume the possible involvement of the unprecedented organocatalytic double activation of both the oxadendralenic dienal and aldehyde, in order to explain the stereochemical reaction outcome as outlined at the top of cycle 1, Scheme 1.

The outcome of the first catalytic cycle in Scheme 1 is a new cyclic oxadendralenic intermediate **3** which is susceptible to a hetero-Diels–Alder reaction as an electron-deficient heterodiene (an inverse-electron demand hetero-Diels–Alder reaction). By adding a Lewis-acid compatible with the organocatalytic reaction conditions, the LUMO energy of the electron-deficient oxadendralenic intermediate **3** will be lowered,^15^ favoring a regioselective hetero-Diels–Alder cycloaddition with vinyl ether **4** to form tetrahydroisochromene **5** with five continuous stereocenters (Scheme 1, cycle 2).

Furthermore, we envisioned an alternative reaction pathway in which the cyclic oxadendralenic intermediate **3** might function as an acceptor in an organocatalytic enamine facilitated cycloaddition. This concept is based on the condensation of aldehyde **2** with organocatalyst **A** raising the HOMO energy, which enables an enantioselective cycloaddition^16^ generating tetrahydroisochromene product **6** with very high enantioselectivity (Scheme 1, cycle 3).

## Results and discussion

**Scheme 2:**
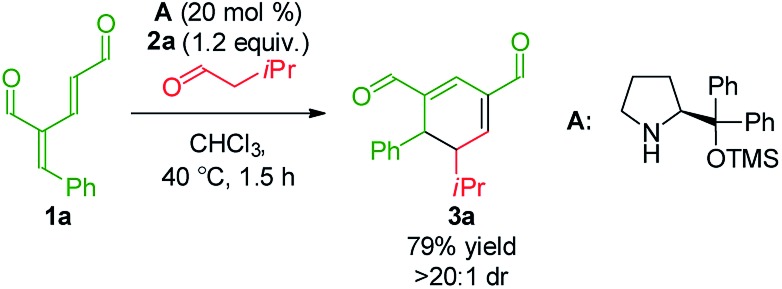
Testing the viability of the organocatalytic annulation for the formation of the oxadendralenic intermediate **3**.

To develop the reaction concept, we started by focusing on the organocatalytic annulation reaction between oxadendralenic dienal **1a** and isovaleric aldehyde **2a** to investigate the formation of the envisaged cyclic oxadendralenic intermediate **3a** (Scheme 2).

**Fig. 3:**
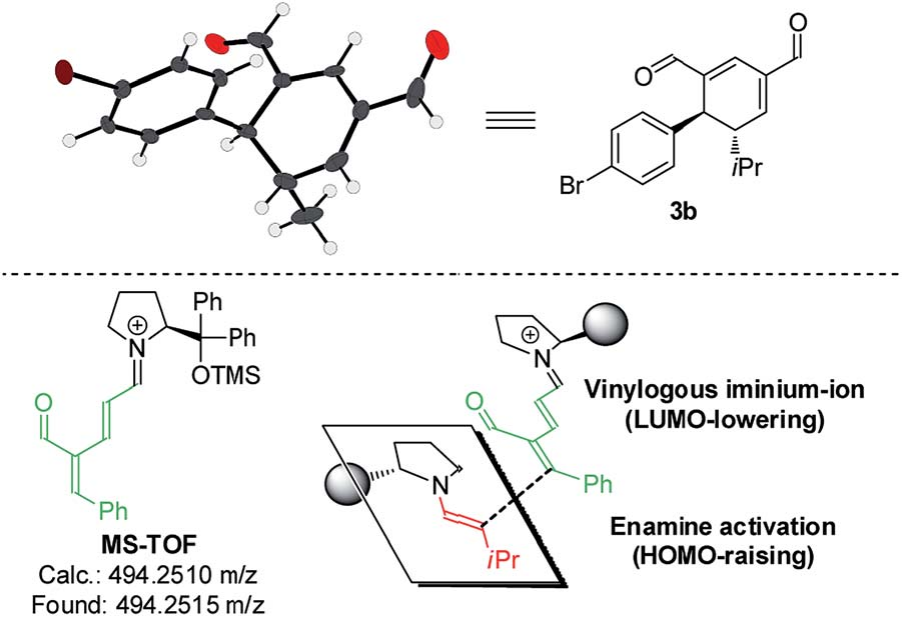
Top: X-ray structure of intermediate **3b**. Bottom: MS-TOF analysis of **1a** condensed with catalyst **A** and the enamine approach to account for the observed stereochemistry of the double activation strategy.

Upon the addition of isovaleric aldehyde **2a** to oxadendralenic dienal **1a** and 20 mol% diphenylprolinol-silyl ether catalyst **A**,^17^ we were delighted to discover that the enamine underwent exclusive 1,6-conjugate addition, facilitating full conversion of the envisioned annulation of the cyclic oxadendralenic intermediate **3a** within only 1.5 h. Compound **3**, formed by the organocatalytic reaction, is a new cyclic oxadendralene, designed to be the intermediate required for the hetero-Diels–Alder reaction leading to the formation of the desired tetrahydroisochromenes. The absolute stereochemistry of the optically active cyclic oxadendralene **3b** was obtained through X-ray analysis of the reaction product derived from the reaction between *para*-brominated oxadendralenic dienal **1b** and **2a** with the addition of 20 mol% **A** (Fig. 3, top).

Notably, the absolute configuration of compound **3b** corresponds to the stereochemistry obtained if the enamine approaches the vinylogous iminium-ion activated oxadendralenic dienal from the bottom face, corresponding to the synergistic double condensation of both compounds with organocatalyst **A** (Fig. 3, bottom). There are examples of Michael acceptors undergoing organocatalytic enamine addition to conjugated esters and sulfones. However, the reaction times of these are in the range of 40–120 h, employing an equal or larger catalyst loading than in the present case.^18^ The short reaction time under the present reaction conditions might support the proposed double activation; however, we set out to provide evidence for the hypothesis. The investigations into the compatibility of oxadendralenic dienals with a secondary aminocatalyst demonstrated that upon mixing oxadendralenic dienal **1a** with organocatalyst **A** the corresponding vinylogous iminium-ion was identified as the most abundant ion in MS-TOF (Fig. 3, bottom). Essentially, we propose that the reaction is initiated by a classic enamine performing a 1,6-conjugate addition to the vinylogous iminium-ion-activated species of the oxadendralenic dienal **1**, followed by an intramolecular aldol condensation forming a reactive oxadendralenic intermediate *in situ* (Scheme 1, cycle 1).

### Combined organo- and Lewis-acid catalysis

The investigation towards the formation of substituted tetrahydroisochromenes was initiated by the reaction of oxadendralenic dienal **1a** with isovaleric aldehyde **2a**. Using the previously mentioned conditions, full conversion into intermediate **3a** was reached within 1.5 h at 40 °C in the presence of 20 mol% **A** in CHCl_3_. Gratifyingly, we discovered that upon the one-pot addition of ethyl vinyl ether **4a** the inverse-electron demand hetero-Diels–Alder reaction proceeded smoothly forming **5a** in a 7 : 1 dr and 99% ee, with a modest 61% yield (Table 1, entry 1). Encouraged by these findings we turned our attention towards the effect of solvents. Performing the reaction in Et_2_O and CH_3_CN resulted in a significant decrease in the yield; however, with a slight improvement in the diastereomeric ratio (Table 1, entries 2 and 3). Notably, EtOH increased the diastereomeric ratio to 11 : 1, nevertheless the yields of **3a** and **5a** were lower (Table 1, entry 4). Upon thorough analysis of the solvent screening we learned that the oxidation of intermediate **3a** into an aromatic 2,4-dicarbaldehyde species was competing with the inverse-electron demand hetero-Diels–Alder reaction, consequently decreasing the yield substantially. To solve this problem we decided to explore the application of the catalytic Lewis-acid activation of the hetero-Diels–Alder cycloaddition. The addition of MgCl_2_ and Yb(fod)_3_ showed no improvements in the reaction (Table 1, entries 5 and 6), whereas the addition of 10 mol% Eu(fod)_3_^19^ gave **5a** in 75% yield, a 15 : 1 dr and 99% ee (Table 1, entry 7). A further improvement was observed when changing the number of equivalents of **1a**, furnishing the desired tetrahydroisochromene **5a** in a diasteromerically pure 88% isolated yield, with a 14 : 1 dr and 99% ee (Table 1, entry 8).

**Table 1:** Optimization of the organocatalytic annulation of oxadendralenic dienal **1a** with **2a** followed by the one-pot inverse-electron demand hetero-Diels–Alder reaction of intermediate **3a** and vinyl ethers **4a** and **b** to form tetrahydroisochromenes **5a** and **b**^*a*^

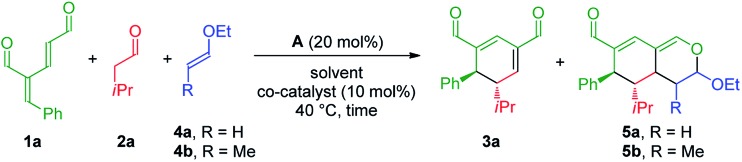								
Entry	R	Solvent	Co-catalyst	*t* (h)	Equiv. of **1a**	Yield of (**3a**/**5a** and **b**) (%)^*b*^	dr of **5a** and **b**^*d*^	ee (%) of **5a** and **b**^*e*^
1	H	CHCl_3_	—	24	1	10/61	7 : 1	99
2	H	Et_2_O	—	24	1	37/34	8 : 1	99
3	H	CH_3_CN	—	24	1	14/33	10 : 1	99
4	H	EtOH	—	24	1	0/53	11 : 1	99
5^*f*^	H	CHCl_3_	MgCl_2_	24	1	12/60	8 : 1	nd
6^*f*^	H	CHCl_3_	Yb(fod)_3_	24	1	nd^*h*^	nd	nd
7^*f*^	H	CHCl_3_	Eu(fod)_3_	24	1	0/75	15 : 1	99
8^*f*^	H	CHCl_3_	Eu(fod)_3_	30	1.5	0/88^*c*^	14 : 1 (>20 : 1)	99
9	Me	CHCl_3_	—	24	1.5	65/29	>20 : 1	99
10^*f*^	Me	CHCl_3_	Eu(fod)_3_	26	1.5	0/90^*c*^	>20 : 1 (>20 : 1)	99
11^*g*^	Me	CHCl_3_	Eu(fod)_3_	24	1.5	18/40	>20 : 1	nd
12^*g*^	Me	CHCl_3_	—	24	1.5	60/28	>20 : 1	99

^*a*^Experiments performed on a 0.1 mmol scale. See the ESI for details.

^*b*^Yields were determined using 1,3,5-tris(trifluoromethyl)benzene as an internal standard unless otherwise noted.

^*c*^Isolated yield determined after FC.

^*d*^Determined using ^1^H NMR analysis of the crude mixture, see dr of the isolated compound in brackets.

^*e*^Determined using chiral stationary phase UPC^2^.

^*f*^Co-catalyst and dienophile were added after 1.5 h of reaction time.

^*g*^All reactants were added simultaneously.

^*h*^The reaction yielded a complex mixture of unidentified products.

Having established the optimal reaction conditions with unsubstituted vinyl ether **4a** as the substrate for the inverse-electron demand hetero-Diels–Alder reaction, we began exploring the possible employment of substituted vinyl ether **4b**, which would potentially generate a fifth stereocenter in the tetrahydroisochromene scaffold. Applying similar conditions as for the reaction utilizing the unsubstituted vinyl ether **4a**, the initial results revealed promising reactivity, as **5b** was formed in 29% yield after 24 h with complete diastereoselectivity and 99% ee (Table 1, entry 9). These observations indicated a considerably longer reaction time for the cycloaddition as high amounts of intermediate **3a** were still present in the crude reaction mixture, attributable to the more bulky dienophile, which could lead to the increased oxidation of intermediate **3a**. Consequently, to accelerate the second reaction step a Lewis acid was added to catalyze the cycloaddition. In accordance with the prior screening results, the addition of Eu(fod)_3_ facilitated the formation of **5b** with an isolated yield of 90%, >20 : 1 dr and 99% ee (Table 1, entry 10). Finally, we explored the possibility of adding all of the reactants simultaneously, potentially simplifying the reaction procedure while strengthening the synthetic applicability. Encouragingly, the unoptimized results demonstrated the potential yield of **5b** to be high, without the addition of Eu(fod)_3_ (Table 1, entries 11 and 12).

We also tested the reaction for the formation of **5a** with different loadings of organocatalyst **A**. The formation of **5a** with 10 mol% of **A** gave 69% yield and the same stereoinduction, while with 5 mol% of **A**, only 36% yield of **5a** was achieved. Performing the same reactions in the presence of Eu(fod)_3_ leads to a significant lowering of the yields compared to the use of 20 mol%. Using 5 mol% of **A** and 10 mol% Eu(fod)_3_ only gave 29% yield of **5a**.

**Scheme 3:**
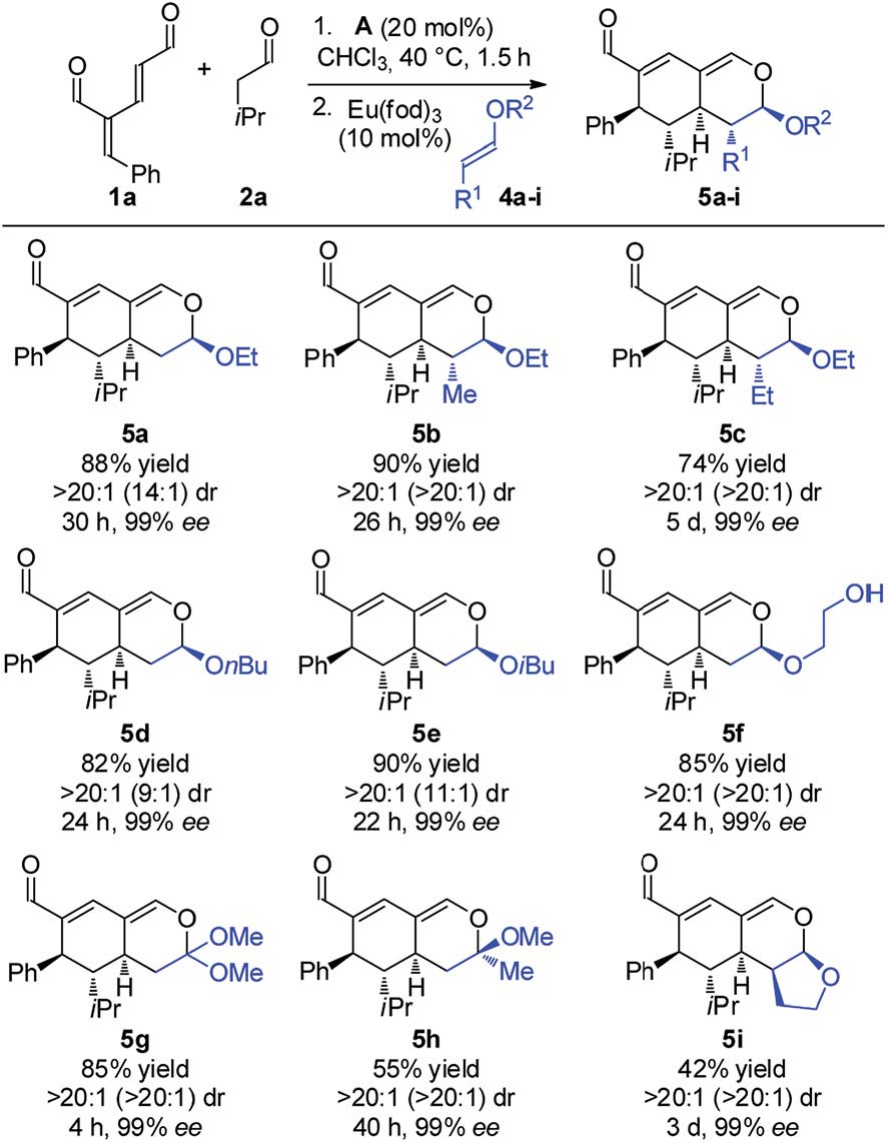
Scope of vinyl ethers **4a–i**.

With the optimized reaction conditions in hand, the scope of the vinyl ether dienophiles **4** in the organocatalytic cascade reaction, using oxadendralenic dienal **1a** and isovaleric aldehyde **2a** as the reaction partners, was investigated (Scheme 3).

Using unsubstituted vinyl ether **4a**, tetrahydroisochromene **5a** was isolated after 30 h of reaction time in a very good yield with perfect diastereoselectivity and an excellent enantioselectivity of 99% ee. Employing substituted vinyl ethers generated products (**5b** and **c**) with five consecutive stereocenters in 99% ee, >20 : 1 dr and high yields. Interestingly, extending the substituent of the vinyl ether by one carbon reduced the reaction rate of the inverse-electron demand hetero-Diels–Alder reaction significantly; however, variations of the ether substituent into branched or linear carbon chains were easily tolerated (**5d** and **e**). Furthermore, it was possible to incorporate a free hydroxy group, showing the robustness of the reaction (**5f**). By utilizing a double activated vinyl ether the reaction rate of the cycloaddition was accelerated considerably, reaching full conversion within 4 h and forming a fully substituted carbon center (**5g**). By means of a similar strategy, a tetrasubstituted stereocenter was installed (**5h**). Delightfully, it proved possible to use a cyclic dienophile, which proceeded to form the tricyclic product **5i** in 42% yield. To demonstrate the utility of the procedure, a scaled-up experiment was performed on a gram scale (3.0 mmol) for the formation of **5b** following the general conditions (see the ESI†).

**Scheme 4:**
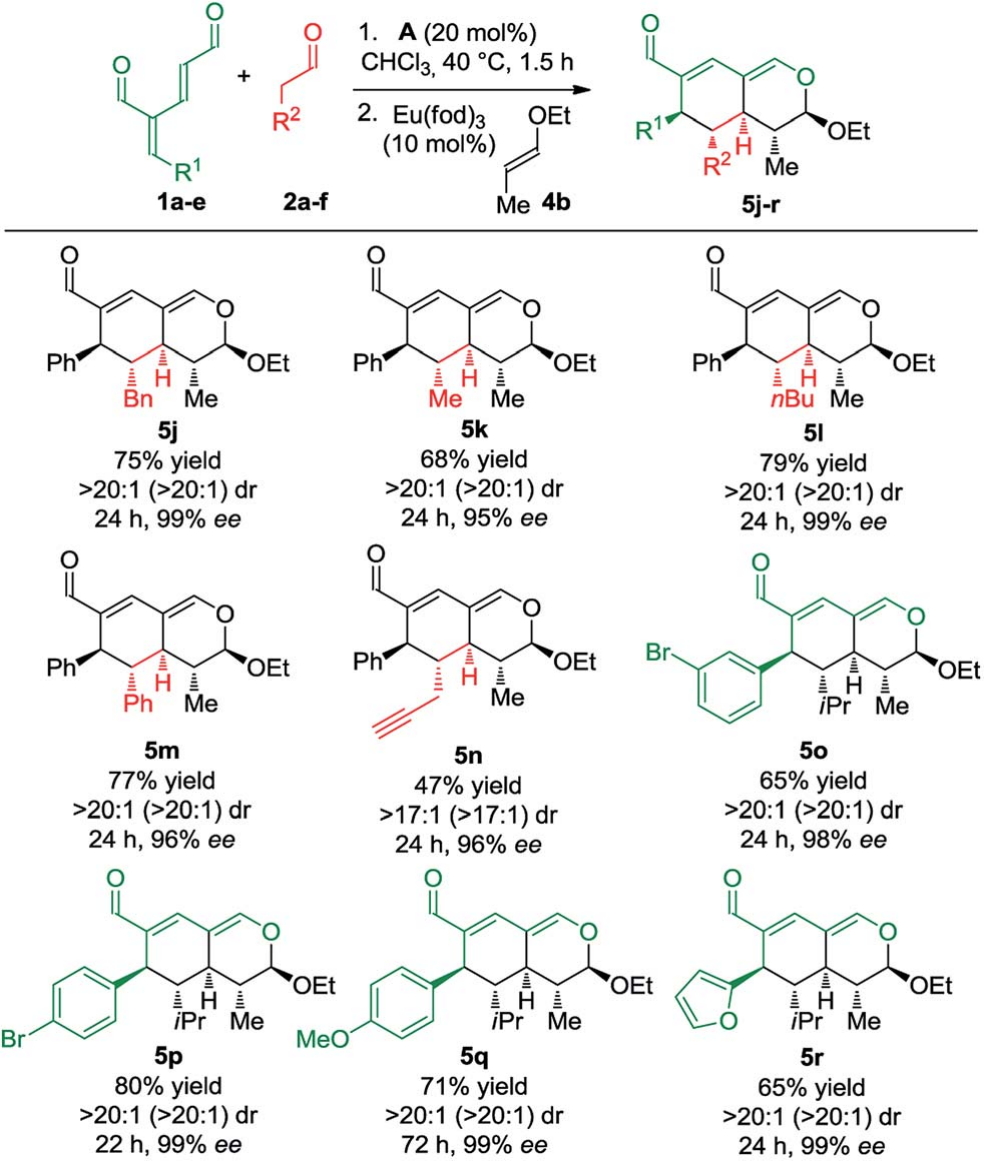
Scope of oxadendralenic dienals **1a–e** and saturated aldehydes **2a–f**.

Next, we focused the investigation towards the oxadendralenic dienals **1** and saturated aldehydes **2**, using the substituted vinyl ether **4b** as the dienophile to ensure better diastereoselectivity and the formation of a fifth stereocenter (Scheme 4).

Various saturated aldehydes were then employed in the reaction outlined in Scheme 4. Both aliphatic and aromatic substituents on the saturated aldehydes afforded the desired products with perfect diastereoselectivity, high enantioselectivity and good yields (**5j–m**). Furthermore, an alkyne substituent was introduced forming **5n** in 47% yield with >17 : 1 dr and 96% ee. The oxadendralenic dienals carrying *meta*- or *para*-substituted bromine underwent the reaction smoothly (**5o** and **p**), and the products displayed results comparable to the other scope entries. An electron-donating group was also well tolerated as the *para*-methoxy substituted oxadendralenic dienal **1d** easily underwent the reaction (**5q**). Furthermore, it was possible to introduce a furan, forming **5r** in 65% yield, >20 : 1 dr and 99% ee.

**Fig. 4:**
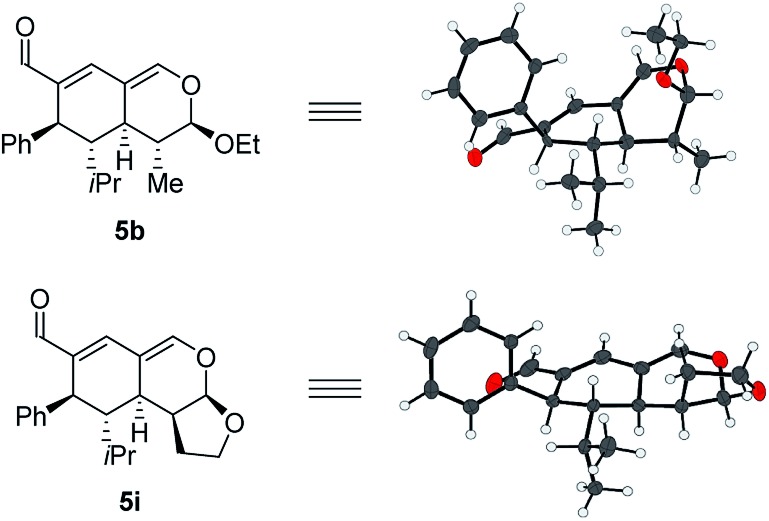
Absolute configuration of tetrahydroisochromenes **5b** and **i** assigned with regards to **3b**.

With a comprehensive scope in hand, we turned our attention towards establishing the absolute configuration of the obtained tetrahydroisochromenes **5**. The relative configuration of tetrahydroisochromenes **5b** and **5i** (Fig. 4) was attained by means of X-ray analysis, from which the absolute configuration was assigned relative to **3b** (Fig. 3). The configuration of the remaining tetrahydroisochromenes **5** was determined analogously.

**Scheme 5:**
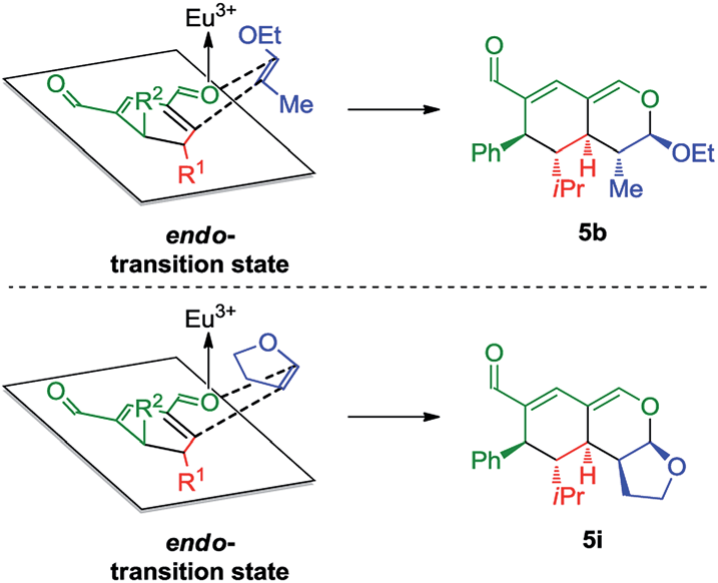
Transition state of the Lewis-acid catalyzed inverse-electron demand hetero-Diels–Alder reaction displaying preferred endo-selectivity.

The stereochemical outcome of the tetrahydroisochromenes **5b** and **5i** can be explained by assuming that the dienophile approaches from the top face due to the steric bulk of the R^1^-substituent (Scheme 5), and proceeds through an *endo*-transition state.

### Sequential organocatalysis

Having established the scope and mechanistic aspects of the organocatalytic cascade featuring a sequential metal catalyzed inverse-electron demand hetero-Diels–Alder reaction, we set out to investigate the viability of utilizing aminocatalytic enamines as alternatives to vinyl ethers (see Scheme 1). We designed a preliminary experiment as a simple procedure, in which an excess of aldehyde **2** would be added to oxadendralenic dienal **1a** with diphenylprolinol-silyl ether **A** in CHCl_3_. Encouragingly, when applying propionaldehyde **2c** with oxadendralenic dienal **1a** in the presence of 20 mol% **A**, the desired tetrahydroisochromene **6a** was procured in 73% yield, a 7 : 1 dr and 99% ee (Table 2, entry 1). Furthermore, the reaction was successfully scaled up, providing 75% yield in a 2.0 mmol reaction. The observed diastereoselectivity is assumed to be controlled by the substituent in the R^1^-position of aldehyde **2**, as the product most likely exists in an equilibrium between the two anomers of **6a**. If isovaleric aldehyde **2a** and hydrocinnamaldehyde **2b** were employed in the envisioned procedure, only the formation of the corresponding cyclic oxadendralenic intermediate **3** would be observed. It is assumed that the lack of reactivity results from steric interactions. Hence, we reasoned that an aldehyde with a smaller substituent was needed for the reaction to proceed. Thus, we explored the hypothesis of incorporating acetaldehyde **2g** as part of the two-step organocatalytic cascade. Following the formation of the cyclic oxadendralenic intermediate **3**, the organocatalytic cycloaddition proceeded well, yielding **6b** in 60% yield, 99% ee and a 2 : 1 dr (Table 2, entry 2).

**Table 2:** Scope of the cascade applying sequential organocatalysis^*a*^

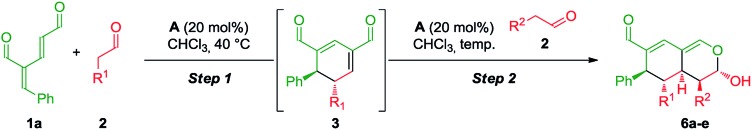										
Entry	R^1^	R^2^	**A** (step 1)	**A** (step 2)	Temp. (step 2)	*t* (h)	Product	Yield^*b*^ (%)	dr^*c*^	ee^*d*^ (%)
1	Me	Me	(*S*)-**A**	—	40 °C	24	**6a**	73	7 : 1	99
2	Me	H	(*S*)-**A**	—	40 °C	24	**6b**	60	2 : 1	99
3	Bn	Bn	(*S*)-**A**	(*R*)-**A**	40 °C	36	**6c**	49	>20 : 1	99
4	*n*Bu	*n*Bu	(*S*)-**A**	(*R*)-**A**	rt	72	**6d**	45	>20 : 1	99
5	Et	Et	(*S*)-**A**	(*R*)-**A**	rt	48	**6e**	47	>20 : 1	99

^*a*^Experiments performed on a 0.2 mmol scale following general procedure C. See ESI Section 5.1 for details.

^*b*^Isolated yield determined after FC.

^*c*^Determined using ^1^H NMR analysis of the isolated compound.

^*d*^Determined using chiral stationary phase UPC^2^.

**Fig. 5:**
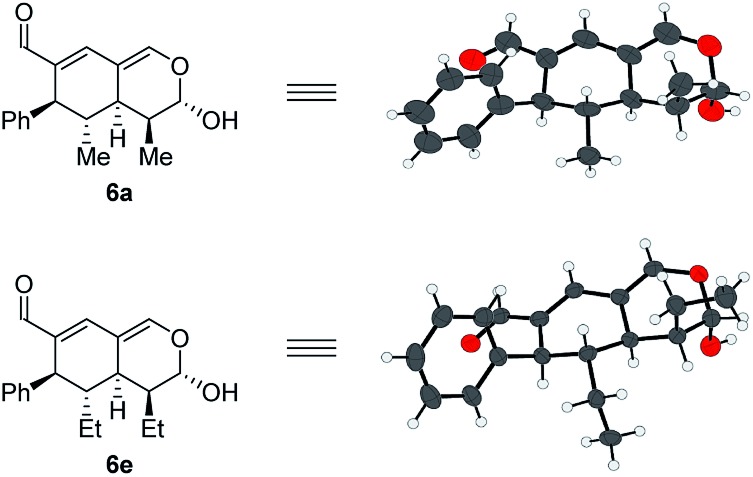
Absolute configuration of tetrahydroisochromenes **6a** and **6e** assigned with regards to **3b**.

**Fig. 6:**
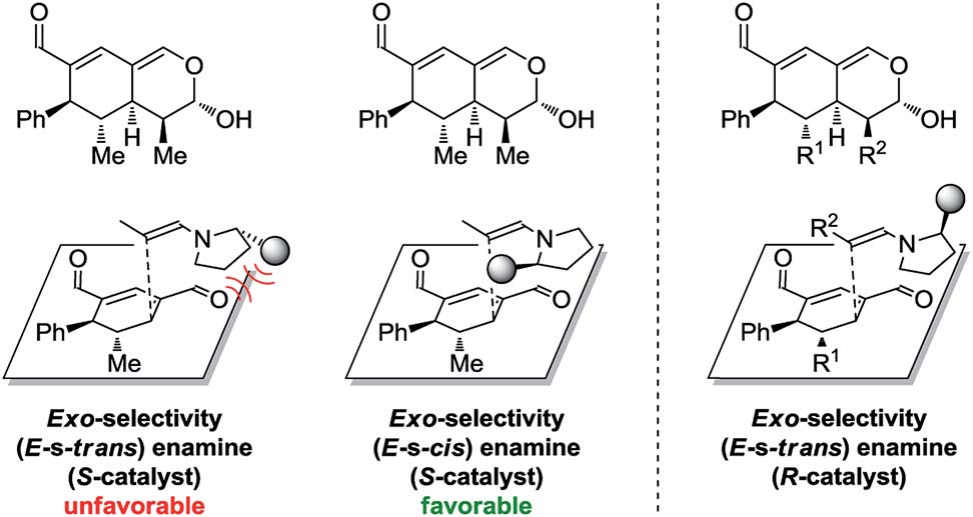
Left: The mechanistic reasoning behind the stereochemical configuration of **6a**. Right: Proposed solution to extend the scope.

In addition to these results, the relative configuration of **6a** (Fig. 5) was obtained using X-ray analysis and the absolute configuration was assigned relative to **3b** (Fig. 3). Interestingly, the second enamine addition provides **6a** with the opposite diastereoselectivity in the dihydropyran ring compared to the formation of tetrahydroisochromene **5b**. As the bottom-face of the cyclic oxadendralenic intermediate **3** is assumed to be sterically blocked, the enamine has to approach from above and proceed through an *exo*-transition state in the inverse-electron demand hetero-Diels–Alder reaction to obtain the observed stereochemistry. This is peculiar as the *exo*-transition state with an enamine intermediate appearing in an (*E*-s-*trans*)-conformation would be unfavorable as a result of the steric clash between **3** and the bulk of the aminocatalyst (*S*)-**A** (Fig. 6, left). However, calculations have shown that a methyl-substituted enamine may just as well exist in the (*E*-s-*cis*)-form, and that this conformation is as favorable as the corresponding (*E*-s-*trans*)-enamine.^20^ Conversely, the (*E*-s-*cis*)-geometry of an enamine substituted with a larger substituent is less favorable. Hence, it is possible that the enamine intermediate of propionaldehyde **2c** reacts through the (*E*-s-*cis*)-conformation, which also explains why isovaleric aldehyde **2a** and hydrocinnamaldehyde **2b** did not work in the reaction as their substituents are too bulky to exist in this form (Fig. 6, left). Based on these considerations, we envisioned adding the other enantiomer of aminocatalyst **A** after the formation of cyclic oxadendralenic intermediate **3**, which should allow for more bulky aldehydes to react in an *exo*-transition state through the (*E*-s-*trans*)-conformation (Fig. 6, right).

We tested this hypothesis by adding 20 mol% (*R*)-**A** to a reaction mixture containing the cyclic oxadendralenic intermediate **3** formed in the reaction between an excess of hydrocinnamaldehyde **2b**, 20 mol% (*S*)-**A** and oxadendralenic dienal **1a**. Full conversion was achieved within 36 h and **6c** was isolated in 49% yield, >20 : 1 dr and 99% ee (Table 2, entry 3). This led us to extend the scope of the reaction, and it was demonstrated that saturated aldehydes bearing linear carbon substituents were tolerated, giving tetrahydroisochromenes **6d** and **e** in >20 : 1 dr, 99% ee and 45–47% yield. The absolute configuration of **6e** (Fig. 5) was determined by analogy with **3b** (Fig. 3), and the observed stereochemistry is consistent with the proposed theory (Fig. 6). To the best of our knowledge this is one of the very rare cases where the enamine intermediate reacts through the (*E*-s-*cis*)-conformer. It should be noted that the implementation of achiral secondary amines such as pyrrolidine in the second step (as a cheap alternative to (*R*)-**A**) was unsuccessful, as their increased basicity resulted in an increased rate of the undesired oxidation of intermediate **3a** into the aromatic 2,4-dicarbaldehyde species.

### Transformations

**Scheme 6:**
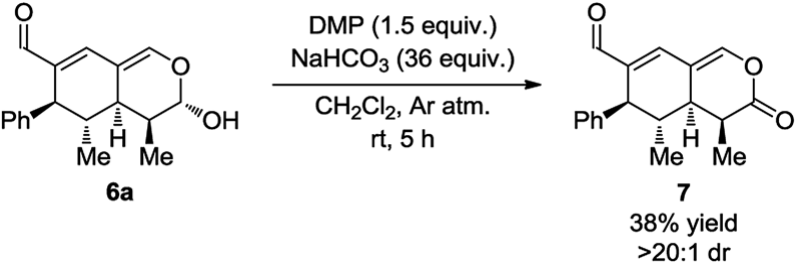
Modified Dess–Martin oxidation of the lactol moiety.

With the intention of strengthening the utility of the developed one-pot cascades even further, transformations of the tetrahydroisochromenes were performed. In particular, functionalization of the lactol moiety in **6a** was explored. First, it was anticipated that the lactol could be oxidized to the corresponding lactone. Using a modified procedure wherein NaHCO_3_ was added to buffer the acidity of the Dess–Martin periodinane, the oxidized product **7** was obtained in 38% yield after only 5 h (Scheme 6).

**Scheme 7:**
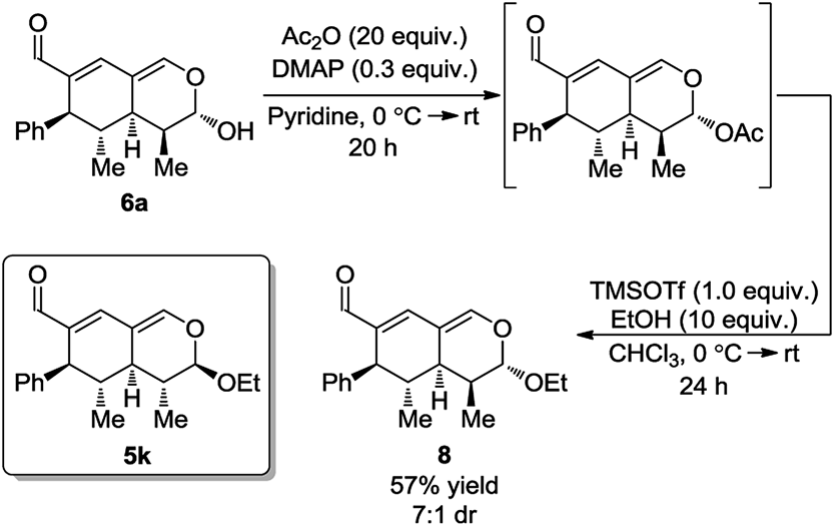
DMAP-catalyzed acetylation and coupling.

Next, it was envisioned that the lactol moiety in **6a** could be converted into an acetal, attaining a product complimentary to tetrahydroisochromene **5k**; however, with the opposite stereochemistry of the substituent positioned next to the acetal. A DMAP-catalyzed acetylation of the hydroxyl group afforded the acetylated intermediate, which subsequently underwent a TMSOTf promoted coupling, yielding the desired product **8** in a 7 : 1 dr and 57% yield (Scheme 7).

## Conclusion

In conclusion, oxadendralenes are introduced in asymmetric catalysis for the construction of chiral bicyclic heterocyclic scaffolds by applying synergistic catalysis. The development is based on the dual activation of the oxadendralene and an aldehyde which forms a vinylogous iminium-ion intermediate and an enamine, respectively. These two intermediates are set-up for the 1,6-addition of the enamine to the vinylogous iminium-ion. This reaction generates a novel cyclic oxadendralenic intermediate, which in a Eu(fod)_3_-catalyzed hetero-Diels–Alder reaction with vinyl ethers forms tetrahydroisochromenes with five continuous stereocenters.

The scope of the reaction is demonstrated for a great variety of oxadendralenes, aldehydes and vinyl ethers giving high substituent diversity of the tetrahydroisochromenes formed in high yields and >20 : 1 dr and up to 99% ee. The dual activation concept is supported by characterization of the vinylogous iminium-ion intermediate formed by reaction of the oxadendralene and the organocatalyst, and the stereochemical outcome of the reaction. The reaction concept has been extended to a double organocatalytic reaction, in which the enamine formed as an intermediate in the first catalytic cycle can be utilized in a second catalytic cycle providing tetrahydroisochromenes with a different stereochemical outcome in moderate to good yields, >20 : 1 dr and up to 99% ee. Finally, the formation of an attractive lactone moiety and diastereoselective acetalization has been demonstrated. The presented novel developments might have the potential to be applied for the formation of novel anti-malaria drug candidates, as the formed tetrahydroisochromene core structure is the central skeleton in artemisinin and arteether. Furthermore, the reaction concept might also be integrated in the synthesis of anti-cancer candidates related to oridonin.

## Supplementary Material

Supplementary information

Crystal structure data

## References

[cit1] Schreiber S. L. (2000). Science.

[cit2] (b) Comprehensive Enantioselective Organocatalysis: Catalysts, Reactions, and Applications, ed. P. I. Dalko, Wiley-VCH, Weinheim, Germany, 2013.

[cit3] Marqués-López E., Herrera R. P., Christmann M. (2010). Nat. Prod. Rep..

[cit4] (c) Sedelmeier G., New Methods, WIPO Patent, WO 2008119804 A1, Novartis AG, 2008.

[cit5] Albrecht Ł., Jiang H., Jørgensen K. A. (2011). Angew. Chem., Int. Ed..

[cit6] Du Z., Shao Z. (2013). Chem. Soc. Rev..

[cit7] Hopf H., Sherburn M. S. (2012). Angew. Chem., Int. Ed..

[cit8] Spino C., Liu G. (1993). J. Org. Chem..

[cit9] Eschenbrenner-Lux V., Kumar K., Waldmann H. (2014). Angew. Chem., Int. Ed..

[cit10] The 2015 Nobel Prize in Physiology or Medicine – Press Release, http://www.nobelprize.org/nobel_prizes/medicine/laureates/2015/press.html, accessed Dec 1, 2015.

[cit11] Zhu C., Cook S. P. (2012). J. Am. Chem. Soc..

[cit12] White N. J. (2008). Science.

[cit13] Zhen T., Wu C.-F., Liu P., Wu H.-Y., Zhou G.-B., Lu Y., Liu J.-X., Liang Y., Li K. K., Wang Y.-Y., Xie Y.-Y., He M.-M., Cao H.-M., Zhang W.-N., Chen L.-M., Petrie K., Chen S.-J., Chen Z. (2012). Sci. Transl. Med..

[cit14] Fuson R. C. (1935). Chem. Rev..

[cit15] (a) Lewis Acids and Selectivity in Organic Synthesis, ed. M. Santelli and J.-M. Pons, CRC Press, BocaBoca Raton, FloridaRaton, Florida, 1996.

[cit16] Juhl K., Jørgensen K. A. (2003). Angew. Chem., Int. Ed..

[cit17] (c) Donslund B. S. Johansen T. K. Poulsen P. H. Halskov K. S. Jørgensen K. A., Angew. Chem., Int. Ed., 2015, 54 , 13860 , . Testing other diarylprolinol-silyl ethers as catalysts gave less satisfactory results . 10.1002/anie.201503920 26423028

[cit18] Murphy J. J., Quintard A., McArdle P., Alexakis A., Stephens J. C. (2011). Angew. Chem., Int. Ed..

[cit19] This is according to the best of our knowledge the first application of europium-catalyzed inverse-electron demand hetero-Diels–Alder reaction in combination with organocatalysis and heterodendralenes

[cit20] Dinér P., Kjærsgaard A., Lie M. A., Jørgensen K. A. (2008). Chem.–Eur. J..

