# Supplementary material for: Oxadendralenes in asymmetric organocatalysis for the construction of tetrahydroisochromenes
Source: Chem Sci. 2016 Feb 17;7(6):3649–57. doi: 10.1039/c6sc00185h (PMC6008721; doi:10.1039/c6sc00185h)
Supplement: Supplementary file 1 [file SC-007-C6SC00185H-s001.pdf]

Electronic Supplementary Material (ESI) for Chemical Science.
This journal is © The Royal Society of Chemistry 2016

Oxadendralenes in Asymmetric Organocatalysis for the Construction of Tetrahydroisochromenes

Niels Hammer, Lars A. Leth, Julian Stiller, Magnus E. Jensen and Karl Anker Jørgensen*

Department of Chemistry, Aarhus University

DK-8000 Aarhus C, Denmark

e-mail: kaj@chem.au.dk

Contents

1. General methods S2
2. Synthesis of starting materials S3
3. Synthesis of the cyclohexadiene dienal **3b** S7
4. Synthesis of the tetrahydroisochromenes **5a-r** S8
5. Synthesis of the tetrahydroisochromenes **6a-e** S14
6. Transformations S16
7. X-Ray structures S18
8. NMR spectra S24
9. UPC^2 traces S61

1. General methods

NMR spectra were acquired on a Varian AS 400 spectrometer or a Bruker AVANCE III HD spectrometer, running at 400 MHz for ^{1}H and 100 MHz for ^{13}C, respectively. Chemical shifts (δ) are reported in ppm relative to residual solvent signals ($CHCl_3$, 7.26 ppm for ^{1}H NMR, $CDCl_3$, 77.0 ppm for ^{13}C NMR, DMSO-d_6, 2.50 ppm for ^{1}H NMR, $CDCl_3$, 39.52 ppm for ^{13}C NMR). The following abbreviations are used to indicate the multiplicity in NMR spectra: s, singlet; d, doublet; t, triplet; q, quartet; m, multiplet; br, broad resonance. ^{13}C NMR spectra were acquired on a broad band decoupled mode. Mass spectra were recorded on a Bruker MicroTOF-Q High-Performance LC-MS system. Analytical thin layer chromatography (TLC) was performed using pre-coated aluminium-backed plates (Merck Kieselgel 60 F254) and visualized by ultraviolet irradiation, $KMnO_4$ or *p*-anisaldehyde dip. Optical rotations were measured on a Bellingham+Stanley ADP440+ polarimeter. The enantiomeric excess (ee) of the products was determined by Ultraperformance Convergence Chromatography (ACQUITY UPC) using Daicel Chiralpak IA, IB, IC and ID columns as chiral stationary phases (columns were kept at 40 °C during measuring). Unless otherwise noted, analytical grade solvents and commercially available reagents were used without further purification. Racemic samples of the tetrahydroisochromenes **5** were prepared using a racemic mixture of enantiomers of TMS-diphenyl prolinol catalyst **A** (20 mol%) in $CHCl_3$, while racemic samples of the tetrahydroisochromenes **6** were prepared by mixing the two enantiomers. For NMR characterization * denotes the minor isomer, $^{+}$ denotes overlap of signals of both isomers, whereas no sign denotes major isomer.

2. Synthesis of starting materials

Overview:

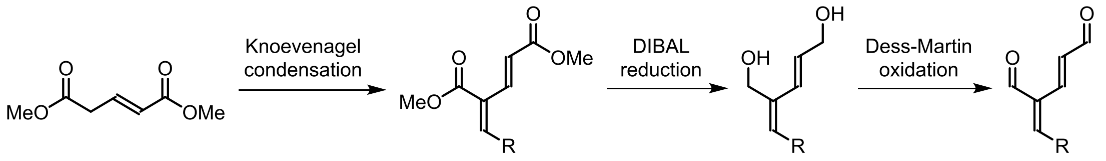

I **II** **III** **1**

2.1. Pent-2-ene-1,5-diols IIIa-e

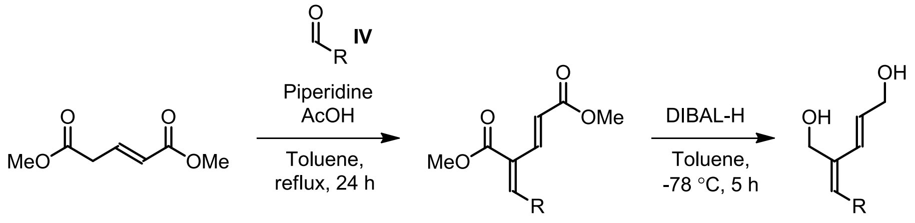

I **II** **III**

General procedure:

A flask was charged with 4Å activated molecular sieves (14 g) and dry toluene (87 mL) were added. Subsequently dimethyl glutaconate **I** (7.0 mmol, 1.0 equiv.), aldehyde **IV** (8.4 mmol, 1.2 equiv.), piperidine (3.5 mmol, 0.5 equiv.) and AcOH (3.5 mmol, 0.5 equiv.) were added accordingly. The reaction mixture was refluxed for 24 h until full conversion. The reaction was quenched by addition of sat. aq. NH_4Cl (excess). After filtration, the filtrate was extracted with EtOAc and washed with sat. $NaHCO_3$. Finally, the organic phase was dried over $MgSO_4$ and the solvent was removed to yield the crude of **II** as a yellow oil. The crude product **II** was used for the next step without further purification.

Under inert atmosphere **II** was solved in dry toluene (20 mL). The mixture was cooled to -78 °C and DIBAL-H (1.0 M in toluene, 35 mmol, 5.0 equiv.) was added dropwise. The mixture was stirred at -78 °C for 5 h to achieve full conversion (TLC). The reaction was quenched by adding EtOAc (5 mL) followed by Rochelles Salt (50 mL). The mixture was then allowed to warm to rt and stirred vigorously overnight. The biphasic mixture was separated and the aqueous layer was extracted with EtOAc (2 x 25 mL) and dried over $MgSO_4$. The solvent was removed and the crude product was purified by FC (Et_2O:CH_2Cl_2 1:1), yielding **III** as a bright yellow solid.

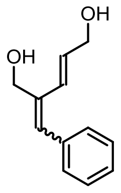

(*E*)-4-((*E*)-Benzylidene)pent-2-ene-1,5-diol IIIa was synthesized following the general procedure and obtained in 67% yield, E/Z 3/1. **^{1}H NMR** (400 MHz, $CDCl_3$) δ 7.40 – 7.24 (m, 5H^{+}), 6.76 – 6.64 (m, 2H, 1H*), 6.40 (d, *J* = 15.9 Hz, 1H*), 6.14 (m, *J* = 16.1, 5.8 Hz, 1H^{+}), 4.49 – 4.44 (m, 2H^{+}), 4.30 (dd, *J* = 5.8, 1.5 Hz, 2H*), 4.24 (dd, *J* = 5.8, 1.6 Hz, 2H), 1.69 (s, 2H^{+}). **^{13}C NMR** (100 MHz, $CDCl_3$) δ 137.0*, 136.6, 136.4^{+}, 134.2*, 133.4*, 130.6, 129.9, 129.3 (2C), 128.9 (2C*), 128.8*, 128.3 (2C*), 128.2 (2C), 127.4*, 127.1, 126.6, 64.7, 63.7, 63.5*, 57.6*. **HRMS** (ESI+) *m/z* calcd. for $C_{12}H_{14}O_2Na$ [M+Na]$^{+}$: 213.0886; found: 213.0886.

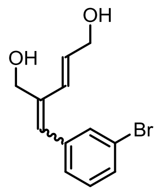

(2*E*)-4-(3-Bromobenzylidene)pent-2-ene-1,5-diol IIIb was synthesized following the general procedure and obtained in 68% yield, E/Z 3/1. **^{1}H NMR** (400 MHz, $CDCl_3$) δ 7.50 (dd, *J* = 1.8 Hz, 1H*), 7.43 – 7.33 (m, 2H, 1H*), 7.31 – 7.28 (m, 1H*), 7.24 – 7.16 (m, 2H, 1H*), 6.67 – 6.53 (m, 2H, 1H*), 6.34 (d, *J* = 15.9 Hz, 1H*), 6.13 (m, 1H^{+}), 4.43 (s, 2H), 4.41 (s, 2H*), 4.27 (dd, *J* = 5.7, 1.4 Hz, 2H*), 4.22 (dd, *J* = 5.9, 1.5 Hz, 2H), 2.30 (s, 2H^{+}). **^{13}C NMR** (100 MHz, $CDCl_3$) δ 138.7, 138.4*, 138.2*, 137.6, 132.9*, 132.4*, 132.0, 131.7*, 131.4, 130.3*, 130.0, 129.8*, 129.7, 129.6*, 127.9, 127.9, 127.5*, 125.9, 122.3*, 122.2, 64.4, 63.6, 63.4*, 57.4*. **HRMS** (ESI+) *m/z* calcd. for $C_{12}H_{13}BrO_2Na$ $[M+Na]^+$: 290.9991; found: 290.9993.

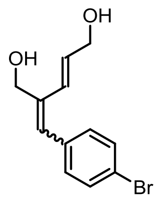

(2*E*)-4-(4-Bromobenzylidene)pent-2-ene-1,5-diol IIIc was synthesized following the general procedure and obtained in 77% yield, E/Z 4/1. **^{1}H NMR** (400 MHz, DMSO-d_6) δ 7.55 (d, *J* = 8.0 Hz, 2H^{+}), 7.39 (d, *J* = 8.2 Hz, 2H*), 7.23 (d, *J* = 8.1 Hz, 2H), 6.62 – 6.50 (m, 2H, 1H*), 6.29 (d, *J* = 15.9 Hz, 1H*), 6.12 (dt, *J* = 15.9, 5.3 Hz, 1H*), 6.02 (dt, *J* = 16.1, 4.9 Hz, 1H), 5.05 (t, *J* = 5.6 Hz, 1H^{+}), 4.79 (m, *J* = 5.6 Hz, 1H^{+}), 4.22 (d, *J* = 5.5 Hz, 2H), 4.16 (d, *J* = 4.8 Hz, 2H*), 4.12 – 4.05 (m, 2H*), 4.03 (t, *J* = 5.1 Hz, 2H). **^{13}C NMR** (100 MHz, DMSO-d_6) δ 139.3*, 138.4, 136.3, 136.0*, 132.6, 131.5*, 131.3*, 131.2 (2C), 131.1 (2C^{+}), 131.0 (2C*), 130.4*, 124.4, 123.4, 120.3*, 119.7, 62.1, 61.6*, 61.5, 56.1*. **HRMS** (ESI+) *m/z* calcd. for $C_{12}H_{13}BrO_2Na$ $[M+Na]^+$: 290.9991; found: 290.9991.

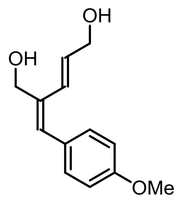

(2*E*)-4-(4-Methoxybenzylidene)pent-2-ene-1,5-diol IIId was synthesized following the general procedure and obtained in 58% yield as a pure (*E*)-isomer. **^{1}H NMR** (400 MHz, $CDCl_3$) δ 7.29 – 7.21 (m, 2H), 6.95 – 6.87 (m, 2H), 6.72 – 6.66 (m, 1H), 6.66 (s, 1H), 6.10 (dt, *J* = 16.1, 6.0 Hz, 1H), 4.42 (d, *J* = 1.1 Hz, 2H), 4.22 (dd, *J* = 6.0, 1.4 Hz, 2H), 3.84 (s, 3H), 3.10 (s, 2H). **^{13}C NMR** (100 MHz, $CDCl_3$) δ 158.7, 135.0, 130.6 (2C), 130.2, 129.6, 129.2, 126.8, 113.6 (2C), 64.9, 63.7, 55.2. **HRMS** (ESI+) *m/z* calcd. for $C_{13}H_{16}O_3Na$ $[M+Na]^+$: 243.0992; found: 243.0994.

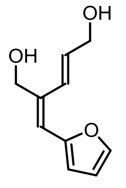

(2*E*,4*E*)-4-(Furan-2-ylmethylene)pent-2-ene-1,5-diol IIIe was synthesized following the general procedure and obtained in 67% yield as a pure (*E*)-isomer. **^{1}H NMR** (400 MHz, $CDCl_3$) δ 7.43 (d, *J* = 1.8 Hz, 1H), 7.22 (m, 1H), 6.40 (dt, *J* = 3.5, 1.8 Hz, 1H), 6.35 (m, 2H), 6.06 (dt, *J* = 16.1, 5.9 Hz, 1H), 4.38 (d, *J* = 1.2 Hz, 2H), 4.27 (dd, *J* = 6.0, 1.6 Hz, 2H), 2.36 (m, 2H). **^{13}C NMR** (100 MHz, $CDCl_3$) δ 152.6, 142.7, 133.8, 130.6, 127.1, 116.7, 111.7, 111.4, 64.5, 63.9. **HRMS** (ESI+) *m/z* calcd. for $C_{10}H_{12}O_3Na$ $[M+Na]^+$: 203.0679; found: 203.0679.

2.2. Oxadendralenic dienals 1a-e

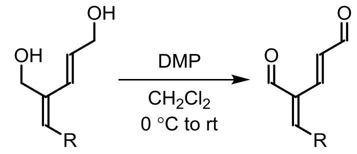

III **1**

General procedure:

A solution of **III** (1.5 mmol, 1 equiv.) in CH_2Cl_2 (16.5 mL) was cooled to 0 °C and Dess-Martin Periodinane (4.5 mmol, 3 equiv.) was added. The reaction mixture was allowed to warm to rt and stirred overnight to achieve full conversion. After that, the mixture was cooled to 0 °C again and diluted with Et_2O (16.5 mL). Subsequently a 1:1:1 mixture of sat. aq. $Na_2S_2O_3$ (5.0 mL), sat. aq. $NaHCO_3$ (5.0 mL) and H_2O (5.0 mL) were added slowly. The reaction mixture was warmed to rt and stirring vigorously for 2 h. The phases were separated and the aqueous layer was extracted with Et_2O (4 x 25 mL). The combined organic layers were dried over Na_2SO_4 and the solvent was removed. The resulting orange solid was purified by FC (pentane:EtOAc 1:10 to 1:5) yielding **1** as a bright yellow solid.

The major isomer of the oxadendralenic dienals is *E* according to the relative configuration obtained via X-ray analysis (Supporting Information Section 7.1.).

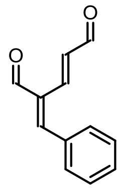

(2*E*)-4-Benzylidenepent-2-enedial 1a was synthesized following the general procedure and obtained in 62% yield as a pure (*E*)-isomer. **^{1}H NMR** (400 MHz, $CDCl_3$) δ 9.77 (d, *J* = 2.1 Hz, 1H), 9.65 (d, *J* = 7.5 Hz, 1H), 7.62 (s, 1H), 7.51 (m, *J* = 1.5 Hz, 5H), 7.36 (ddd, *J* = 16.1, 2.1, 1.0 Hz, 1H), 7.23 (dd, *J* = 16.3, 7.5 Hz, 1H). **^{13}C NMR** (100 MHz, $CDCl_3$) δ 194.4, 191.8, 155.3, 141.5, 134.7, 133.9, 133.7, 131.2, 130.5 (2C), 129.1 (2C). **HRMS** (ESI+) *m/z* calcd. for $C_{12}H_{11}O_2$[M+H]$^+$: 187.0754; found: 187.0752.

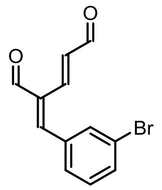

(*E*)-4-((*E*)-3-Bromobenzylidene)pent-2-enedial 1b was synthesized following the general procedure and obtained in 67% yield as a pure (*E*)-isomer. **^{1}H NMR** (400 MHz, $CDCl_3$) δ 9.76 (d, *J* = 2.0 Hz, 1H), 9.65 (d, *J* = 7.1 Hz, 1H), 7.64 (m, 2H), 7.53 (s, 1H), 7.45 – 7.36 (m, 2H), 7.32 – 7.26 (m, 1H), 7.21 (dd, *J* = 16.2, 7.1 Hz, 1H). **^{13}C NMR** (100 MHz, $CDCl_3$) δ 194.1, 191.4, 152.8, 140.5, 135.6, 135.3, 135.0, 133.9, 133.0, 130.6, 128.7, 123.2. **HRMS** (ESI+) *m/z* calcd. for $C_{12}H_{10}BrO_2$ [M+H]$^+$: 264.9859; found: 264.9857.

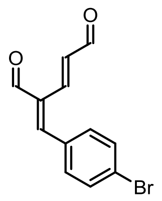

(*E*)-4-((*E*)-4-Bromobenzylidene)pent-2-enedial 1c was synthesized following the general procedure and obtained in 40% yield as a pure (*E*)-isomer. **^{1}H NMR** (400 MHz, $CDCl_3$) δ 9.75 (d, *J* = 2.0 Hz, 1H), 9.65 (d, *J* = 7.0 Hz, 1H), 7.69 – 7.63 (m, 2H), 7.53 (s, 1H), 7.39 – 7.34 (m, 2H), 7.32 – 7.26 (m, 1H), 7.21 (dd, *J* = 16.2, 7.0 Hz, 1H). **^{13}C NMR** (100 MHz, $CDCl_3$) δ 193.9, 191.4, 153.2, 140.7, 135.1, 134.4, 132.6, 132.5 (2C), 131.7 (2C), 125.9. **HRMS** (ESI+) *m/z* calcd. for $C_{12}H_{10}BrO_2$ [M+H]$^+$: 264.9859; found: 264.9855.

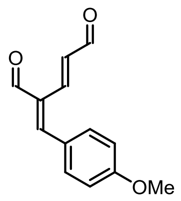

(*E*)-4-((*E*)-4-Methoxybenzylidene)pent-2-enedial 1d was synthesized following the general procedure and obtained in 53% yield as a pure (*E*)-isomer. **^{1}H NMR** (400 MHz, $CDCl_3$) δ 9.71 (d, *J* = 2.1 Hz, 1H), 9.67 (d, *J* = 7.6 Hz, 1H), 7.55 – 7.47 (m, 3H), 7.38 (ddd, *J* = 16.1, 2.2, 1.1 Hz, 1H), 7.22 (dd, *J* = 16.1, 7.5 Hz, 1H), 7.06 – 6.99 (m, 2H), 3.90 (s, 3H). **^{13}C NMR** (100 MHz, $CDCl_3$) δ 194.3, 191.7, 162.4, 155.0, 142.0, 134.2, 132.9 (2C), 132.1, 126.5, 114.8 (2C), 55.6. **HRMS** (ESI+) *m*/*z* calcd. for $C_{13}H_{12}O_3Na$ $[M+Na]^+$: 239.0684; found: 239.0682.

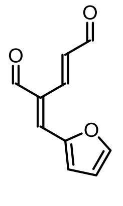

(2*E*,4*E*)-4-(Furan-2-ylmethylene)pent-2-enedial 1e was synthesized following the general procedure and obtained in 56% yield as a pure (*E*)-isomer. **^{1}H NMR** (400 MHz, $CDCl_3$) δ 9.71 (d, *J* = 7.7 Hz, 1H), 9.69 (d, *J* = 2.3 Hz, 1H), 8.01 (ddd, *J* = 16.1, 2.3, 0.9 Hz, 1H), 7.80 (d, *J* = 1.7 Hz, 1H), 7.32 (dd, *J* = 16.1, 7.7 Hz, 1H), 7.12 (s, 1H), 7.02 (d, *J* = 3.5 Hz, 1H), 6.67 (dd, *J* = 3.6, 1.8 Hz, 1H). **^{13}C NMR** (100 MHz, $CDCl_3$) δ 194.7, 191.4, 151.0, 148.3, 142.4, 137.5, 133.7, 129.0, 122.3, 113.5. **HRMS** (ESI+) *m*/*z* calcd. for $C_{10}H_9O_3$ $[M+H]^+$: 177.0546; found: 177.0546.

2.3. Pent-4-ynal 2f

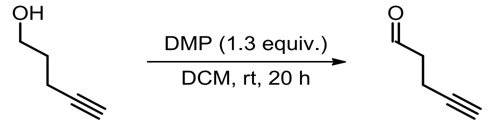

V **2f**

Procedure: To a 250 mL round bottomed flask fitted with a magnetic stir bar, **23** (10.0 mmol, 1 equiv.) was added to CH_2Cl_2 (55 mL) followed by Dess-Martin Periodinane (13.0 mmol, 1.3 equiv.). The reaction mixture was stirred at rt. After 20 h, the reaction was complete, as determined by TLC (pentane:Et_2O 7:1). The mixture was diluted with Et_2O (50 mL) and subsequently quenched by addition of a 1:1:1 mixture of sat. aq. $Na_2S_2O_3$ (30 mL), sat. aq. $NaHCO_3$ (30 mL) and H_2O (30 mL), and additional H_2O (50 mL) were added. The phases were separated, and the aqueous phase was extracted with Et_2O (2 x 15 mL). The organic phases were combined and dried over $MgSO_4$. The crude was concentrated at 50 °C, to remove excess solvents. **2f** was procured in 70% yield as part of a mixture containing 80% compound and 20% CH_2Cl_2 and Et_2O.
The characterization of **2f** is known in literature, and corresponds to the obtained product.[1]

[1] Y. Yan, J. Chen, L. Zhang, Q. Zheng, Y. Han, H. Zhang, D. Zhang, T. Awakawa, I. Abe and W. Liu, *Angew. Chem. Int. Ed.* **2013**, *52*, 12308.

3. Synthesis of the cyclic oxadendralenic intermediates 3a,b

3.1. Procedure

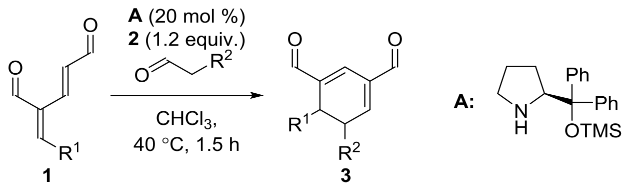

In a 4 mL glass vial catalyst (*S*)-**A** (0.06 mmol, 0.2 equiv.), aldehyde **2** (0.9 mmol, 1.2 equiv.) and oxadendralenic dienal **1** (0.2 mmol, 1.0 equiv.) were mixed in $CHCl_3$ (0.4 mL). The reaction mixture was heated to 40 °C until completion. Full conversion was determined by ^{1}H NMR analysis. The crude was loaded directly onto a column and FC (pentane:EtOAc) afforded **3**.

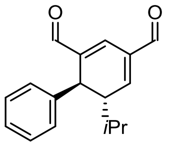

(1*S*,6*S*)-6-Methyl-1,6-dihydro-[1,1'-biphenyl]-2,4-dicarbaldehyde 3a was obtained after 1.5 h following the procedure. FC (pentane:EtOAc 40:1 to 30:1) yielded **3a** as a white solid in 79% yield, >20:1 dr. **^{1}H NMR** (400 MHz, $CDCl_3$) δ 9.62 (s, 1H), 9.61 (s, 1H), 7.46 – 7.42 (m, 1H), 7.25 – 7.16 (m, 3H), 7.12 – 7.08 (m, 2H), 7.06 (dt, *J* = 5.8, 0.9 Hz, 1H), 4.14 – 4.07 (m, 1H), 2.72 (td, *J* = 5.8, 1.6 Hz, 1H), 2.02 – 1.85 (m, 1H), 1.02 (d, *J* = 6.8 Hz, 3H), 0.98 (d, *J* = 6.8 Hz, 3H). **^{13}C NMR** (100 MHz, $CDCl_3$) δ 192.4, 189.7, 155.3, 142.6, 140.7, 136.5, 134.6, 128.8 (2C), 127.0, 126.7 (2C), 50.6, 37.8, 33.9, 19.4, 19.3. **HRMS** (ESI+) *m/z* calcd. for $C_{17}H_{18}NaO_2$ $[M+Na]^+$: 277.1119; found: 277.1165.

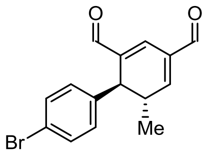

(1*S*,6*S*)-4'-Bromo-6-methyl-1,6-dihydro-[1,1'-biphenyl]-2,4-dicarbaldehyde 3b was obtained after 2 h following the procedure on 0.3 mmol scale with 3 equivalents of **2**. FC (pentane:EtOAc 8:1) yielded **3b** as a yellow oil in 18% yield, >20:1 dr. **^{1}H NMR** (400 MHz, $CDCl_3$) δ 9.67 (s, 1H), 9.59 (s, 1H), 7.49 (d, *J* = 1.5 Hz, 1H), 7.38 – 7.32 (m, 2H), 7.12 – 7.07 (m, 1H), 7.02 – 6.95 (m, 2H), 3.90 (d, *J* = 2.2 Hz, 1H), 2.92 (qdd, *J* = 7.5, 5.9, 1.5 Hz, 1H), 1.22 (d, *J* = 7.3 Hz, 3H). **^{13}C NMR** (100 MHz, $CDCl_3$) δ 192.6, 189.7, 156.9, 140.3, 139.0, 135.0, 134.5, 131.8 (2C), 128.6 (2C), 121.1, 41.7, 38.3, 19.2. **HRMS** (ESI+) *m/z* calcd. for $C_{15}H_{14}BrO_2$ $[M+H]^+$: 305.0172; found: 305.0165.

4. Synthesis of the tetrahydroisochromenes 5a-r

4.1. General procedure A

In a 4 mL glass vial catalyst **A** (0.04 mmol, 0.2 equiv.) and pent-2-enedial **1** (0.3 mmol, 1.5 equiv.) were dissolved in $CHCl_3$ (0.2 mL). Aldehyde **2** (0.2 mmol, 1.0 equiv.) in $CHCl_3$ (0.2 mL) was added as a stock solution. The reaction mixture was heated to 40 °C for 1.5 h. Afterwards dienophile **4** (1.0 mmol, 5.0 equiv.) and $Eu(fod)_3$ (0.02 mmol, 0.1 equiv.) were added and the reaction was stirred at 40 °C for the time indicated. Full conversion was determined by a control experiment performed at 0.05 mmol scale of aldehyde from which the reaction progress was monitored by NMR. After full conversion the crude reaction mixture was loaded on a column and purified by FC (pentane:EtOAc) yielding the corresponding product **5**.

4.2. General procedure B

In a 4 mL glass vial catalyst **A** (0.04 mmol, 0.2 equiv.), aldehyde **2** (0.6 mmol, 3.0 equiv.) and pent-2-enedial **1** (0.2 mmol, 1.0 equiv.) were dissolved in $CHCl_3$ (0.4 mL). The reaction mixture was heated to 40 °C for 1.5 h. Afterwards dienophile **4** (1.0 mmol, 5.0 equiv.) and $Eu(fod)_3$ (0.02 mmol, 0.1 equiv.) were added and the reaction was stirred at 40 °C for the time indicated. Full conversion was determined by a control experiment performed at 0.05 mmol scale of aldehyde from which the reaction progress was monitored by NMR. After full conversion the crude reaction mixture was loaded on a column and purified by FC (pentane:EtOAc) yielding the corresponding product **5**.

4.3. Results and characterization

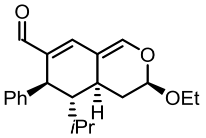

Product **5a** was obtained after 24 h following general procedure A. FC (pentane:EtOAc 40:1) afforded the pure compound in 88% yield, >20:1 dr and 99% ee. Yellow oil. $[\alpha]_D^{20}$ = –95.8 (*c*=1.0, CH_2Cl_2). **^{1}H NMR** (400 MHz, $CDCl_3$) δ 9.16 (s, 1H), 7.25 – 7.13 (m, 5H), 7.02 – 6.97 (m, 1H), 6.87 (d, *J* = 1.9 Hz, 1H), 5.03 (dd, *J* = 9.9, 2.0 Hz, 1H), 4.03 (dq, *J* = 9.5, 7.1 Hz, 1H), 3.67 (dq, *J* = 9.5, 7.1 Hz, 1H), 3.55 – 3.50 (m, 1H), 2.54 – 2.42 (m, 1H), 2.37 (ddd, *J* = 13.1, 5.4, 2.0 Hz, 1H), 1.91 (pd, *J* = 7.2, 1.5 Hz, 1H), 1.70 – 1.54 (m, 2H), 1.30 (t, *J* = 7.1 Hz, 3H), 0.91 (d, *J* = 7.3 Hz, 3H), 0.81 (d, *J* = 7.2 Hz, 3H). **^{13}C NMR** (100 MHz, $CDCl_3$) δ 191.4, 147.1, 144.4, 142.8, 138.7, 128.8 (2C), 128.0 (2C), 126.2, 115.0, 101.1, 65.1, 51.4, 43.9, 34.2, 31.9, 27.5, 21.7, 17.1, 15.1. **HRMS** (ESI+) *m/z* calcd. for $C_{21}H_{27}O_3$ $[M+H]^+$: 327.1955; found: 327.1955. **UPC^2**: IC, CO_2/*i*PrOH 80/20, 3.0 mL·min^{-1}; t_{minor} = 4.0 min; t_{major} = 5.6 min.

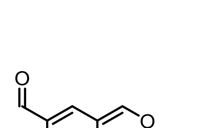

Product **5b** was obtained after 24 h following general procedure A. FC (pentane:EtOAc 30:1) afforded the pure compound in 90% yield, >20:1 dr and 99% ee. Yellow oil. $[\alpha]_D^{20}$ = +17.0 (*c*=1, CH_2Cl_2). **^{1}H NMR** (400 MHz, $CDCl_3$) δ 9.42 (s, 1H), 7.23 – 7.07 (m, 6H), 6.85 (d, J = 2.1 Hz, 1H), 4.83 (d, J = 5.8 Hz, 1H), 3.98 (d, J = 4.2 Hz, 1H), 3.88 (dq, J = 9.6, 7.1 Hz, 1H), 3.55 (dq, J = 9.6, 7.0 Hz, 1H), 2.22 (dt, J = 6.4, 4.0 Hz, 1H), 2.02 – 1.95 (m, 1H), 1.80 (dtd, J = 11.8, 6.8, 6.2, 2.9 Hz, 2H), 1.21 (t, J = 7.1 Hz, 3H), 0.95 (dd, J = 6.9, 2.8 Hz, 6H), 0.89 (d, J = 6.9 Hz, 3H). **^{13}C NMR** (100 MHz, $CDCl_3$) δ 191.4, 145.4, 145.3, 144.6, 139.1, 128.2 (4C), 125.9, 116.0, 105.2, 64.6, 48.5, 40.2, 38.3, 35.8, 32.3, 20.4, 19.1, 15.9, 15.0. **HRMS** (ESI+) *m/z* calcd. for $C_{22}H_{28}O_3K$ $[M+K]^+$: 379.1670; found: 379.1671. **UPC^2**: IC, CO_2/*i*PrOH 90/10, 3.0 mL·min^{-1}; t_{minor} = 5.7 min; t_{major} = 6.7 min.

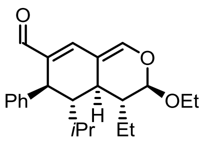

Product **5c** was obtained after 120 h following general procedure A. FC (pentane:EtOAc 50:1) afforded the pure compound in 74% yield, >20:1 dr and 99% ee. Yellow oil. $[\alpha]_D^{20}$ = +30.1 (*c*=0.4, CH_2Cl_2). **^{1}H NMR** (400 MHz, $CDCl_3$) δ 9.28 (s, 1H), 7.22 – 7.17 (m, 4H), 7.11 (d, J = 6.0 Hz, 2H), 6.77 (d, J = 2.0 Hz, 1H), 4.99 (d, J = 3.0 Hz, 1H), 3.88 – 3.77 (m, 2H), 3.52 (dq, J = 9.5, 7.0 Hz, 1H), 2.35 (ddd, J = 9.7, 6.9, 2.6 Hz, 1H), 1.96 (dtd, J = 13.6, 6.8, 2.9 Hz, 2H), 1.88 (dt, J = 10.1, 2.6 Hz, 1H), 1.52 – 1.34 (m, 2H), 1.20 (t, J = 7.0 Hz, 3H), 0.97 – 0.90 (m, 7H), 0.84 (d, J = 7.1 Hz, 3H). **^{13}C NMR** (100 MHz, $CDCl_3$) δ 191.8, 146.0, 145.2, 142.8, 140.1, 128.9 (2C), 127.9 (2C), 125.8, 117.0, 101.8, 64.1, 49.7, 42.0, 40.3, 36.8, 29.1, 24.8, 20.2, 18.9, 15.1, 11.7. **HRMS** (ESI+) *m/z* calcd. for $C_{23}H_{31}O_3$ $[M+H]^+$: 355.2268; found: 355.2266. **UPC^2**: IC, CO_2/*i*PrOH 80/20, 3.0 mL·min^{-1}; t_{minor} = 2.4 min; t_{major} = 2.8 min.

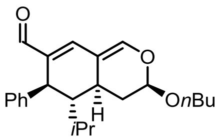

Product **5d** was obtained after 24 h following general procedure A. FC (pentane:EtOAc 20:1) afforded the pure compound in 82% yield, >20:1 dr and 99% ee. Yellow oil. $[\alpha]_D^{20}$ = +19.0 (*c*=1.0, CH_2Cl_2). **^{1}H NMR** (400 MHz, $CDCl_3$) δ 9.16 (s, 1H), 7.22 (d, *J* = 7.1 Hz, 2H), 7.18 – 7.11 (m, 3H), 6.98 (s, 1H), 6.86 (d, *J* = 1.4 Hz, 1H), 5.01 (dd, *J* = 9.8, 1.9 Hz, 1H), 3.96 (dt, *J* = 9.5, 6.7 Hz, 1H), 3.58 (dt, *J* = 9.5, 6.7 Hz, 1H), 3.52 (d, *J* = 9.7, 1H), 2.51 – 2.41 (m, 1H), 2.35 (ddd, *J* = 13.1, 5.4, 1.9 Hz, 1H), 1.91 (dsept, *J* = 7.2 Hz, 1.0 Hz, 1H), 1.69 – 1.56 (m, 4H), 1.47 – 1.37 (m, 2H), 0.95 (d, *J* = 7.4 Hz, 3H), 0.90 (d, *J* = 7.3 Hz, 3H), 0.79 (d, *J* = 7.2 Hz, 3H). **^{13}C NMR** (100 MHz, $CDCl_3$) δ 191.6, 147.3, 144.5, 142.9, 138.6, 128.9 (2C), 128.1 (2C), 126.2, 115.0, 101.3, 69.5, 51.5, 43.9, 34.2, 32.0, 31.7, 27.5, 21.6, 19.2, 17.2, 13.9. **HRMS** (ESI+) *m/z* calcd. for $C_{23}H_{31}O_3$ $[M+H]^+$: 355.2268; found: 355.2271. **UPC^2**: IC, CO_2/*i*PrOH 80/20, 3.0 mL·min^{-1}; t_{minor} = 4.0 min; t_{major} = 5.8 min.

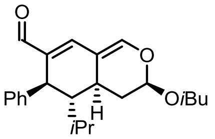

Product **5e** was obtained after 22 h following general procedure A. FC (pentane:EtOAc 20:1) afforded the pure compound in 90% yield, >20:1 dr and 99% ee. Yellow oil. $[\alpha]_D^{20}$ = +2.3 (*c*=1.0, CH_2Cl_2). **^{1}H NMR** (400 MHz, $CDCl_3$) δ 9.16 (s, 1H), 7.31 – 7.19 (m, 2H), 7.19 – 7.11 (m, 3H), 6.98 (s, 1H), 6.86 (d, *J* = 1.1 Hz, 1H), 5.00 (dd, *J* = 9.7, 1.9 Hz, 1H), 3.73 (dd, *J* = 9.2, 6.6 Hz, 1H), 3.53 (d, *J* = 9.7 Hz, 1H), 3.33 (d, *J* = 9.2, 6.8 Hz, 1H), 2.47 (td, *J* = 11.5, 4.9 Hz, 1H), 2.35 (ddd, *J* = 13.0, 5.4, 1.8 Hz, 1H), 1.92 (m, 2H), 1.72 – 1.55 (m, 2H), 0.95 (dd, *J* = 6.7, 1.1 Hz, 6H), 0.91 (d, *J* = 7.2 Hz, 3H), 0.79 (d, *J* = 7.2 Hz, 3H). **^{13}C NMR** (100 MHz, $CDCl_3$) δ 191.4, 147.2, 144.5, 142.8, 138.7, 128.8 (2C), 128.0 (2C), 126.2, 115.0, 101.5, 76.3, 51.5, 43.8, 34.0, 32.0, 28.5, 27.5, 21.5, 19.3, 19.2, 17.3. **HRMS** (ESI+) *m/z* calcd. for $C_{23}H_{30}O_3Na$ $[M+Na]^+$: 377.2087; found: 377.2085. **UPC^2**: IC, CO_2/*i*PrOH 80/20, 3.0 mL·min^{-1}; t_{minor} = 3.6 min; t_{major} = 5.4 min.

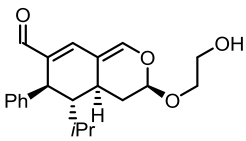

Product **5f** was obtained after 24 h following general procedure A. FC (pentane:EtOAc 4:1 to 3:1) afforded the pure compound in 85% yield, >20:1 dr and 99% ee. Yellow oil. $[\alpha]_D^{20}$ = –76.8 (*c*=1.0, CH_2Cl_2). **^{1}H NMR** (400 MHz, $CDCl_3$) δ 9.16 (s, 1H), 7.26 – 7.11 (m, 5H), 7.00 – 6.95 (m, 1H), 6.88 – 6.82 (m, 1H), 5.06 (dd, *J* = 9.8, 2.0 Hz, 1H), 4.03 (qd, *J* = 7.1, 6.2, 4.3 Hz, 1H), 3.81 (q, *J* = 3.4 Hz, 3H), 3.58 – 3.48 (m, 1H), 2.55 – 2.37 (m, 2H), 2.13 (s, 1H), 1.98 – 1.86 (m, 1H), 1.73 – 1.54 (m, 2H), 0.90 (d, *J* = 7.3 Hz, 3H), 0.80 (d, *J* = 7.1 Hz, 3H). **^{13}C NMR** (100 MHz, $CDCl_3$) δ 191.4, 146.6, 144.2, 142.3, 138.9, 128.8 (2C), 128.0 (2C), 126.2, 115.1, 101.5, 71.2, 61.8, 51.4, 43.9, 34.0, 31.8, 27.5, 21.7, 17.1. **HRMS** (ESI+) *m/z* calcd. for $C_{21}H_{27}O_4$ $[M+H]^+$: 343.1904; found: 343.1908. **UPC^2**: IC, CO_2/*i*PrOH 60/40, 3.0 mL·min^{-1}; t_{minor} = 3.1 min; t_{major} = 3.9 min.

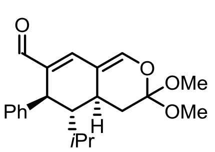

Product **5g** was obtained after 4 h following general procedure A without using Eu(fod)$_3$. FC (pentane:EtOAc 20:1) afforded the pure compound in 84% yield, >20:1 dr and 99% ee. Yellow oil. $[\alpha]_D^{20}$ = +100.0 (*c*=1.0, CH_2Cl_2). **^{1}H NMR** (400 MHz, $CDCl_3$) δ 9.18 (s, 1H), 7.25 – 7.21 (m, 2H), 7.18 – 7.13 (m, 3H), 6.98 (m, 1H), 6.79 (d, *J* = 1.9 Hz, 1H), 3.55 (d, *J* = 9.8 Hz, 1H), 3.43 (s, 3H), 3.34 (s, 3H), 2.58 – 2.48 (m, 1H), 2.42 (dd, *J* = 13.0, 5.6 Hz, 1H), 1.91 (m, 1H), 1.66 – 1.56 (m, 2H), 0.90 (d, *J* = 7.3 Hz, 3H), 0.82 (d, *J* = 7.2 Hz, 3H). **^{13}C NMR** (100 MHz, $CDCl_3$) δ 191.4, 145.2, 144.4, 142.2, 139.3, 128.8 (2C), 128.0 (2C), 126.2, 116.1, 113.5, 50.8, 50.5, 48.9, 43.8, 32.8, 30.6, 27.5, 21.7, 17.2. **HRMS** (ESI+) *m/z* calcd. for $C_{21}H_{27}O_4$ $[M+H]^+$: 343.1904; found: 343.1903. **UPC^2**: IB, CO_2/MeCN 95/5, 3.0 mL·min^{-1}; t_{minor} = 5.4 min; t_{major} = 6.8 min.

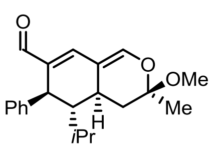

Product **5h** was obtained after 40 h following general procedure A. FC (pentane:EtOAc 25:1) afforded the pure compound in 55% yield, >20:1 dr and 99% ee. Yellow oil. $[\alpha]_D^{20}$ = -35.6 (*c*=1.0, CH_2Cl_2). **^{1}H NMR** (400 MHz, $CDCl_3$) δ 9.16 (s, 1H), 7.23 (d, *J* = 7.1 Hz, 2H), 7.20 – 7.12 (m, 3H), 6.99 (s, 1H), 6.86 (s, 1H), 3.54 (d, *J* = 9.8 Hz, 1H), 3.43 (s, 3H), 2.41 (dt, *J* = 11.5, 5.1 Hz, 1H), 2.09 (dd, *J* = 13.0, 5.4 Hz, 1H), 1.92 (p, *J* = 7.4 Hz, 1H), 1.77 (t, *J* = 12.4 Hz, 1H), 1.62 (ddd, *J* = 11.5, 9.9, 1.5 Hz, 1H), 1.45 (s, 3H), 0.90 (d, *J* = 7.3 Hz, 3H), 0.82 (d, *J* = 7.2 Hz, 3H). **^{13}C NMR** (100 MHz, $CDCl_3$) δ 191.3, 147.6, 144.5, 143.0, 138.4, 128.8 (2C), 128.0 (2C), 126.2, 113.7, 102.8, 51.3, 49.6, 44.0, 36.0, 31.3, 27.5, 22.5, 21.8, 17.0. **HRMS** (ESI+) *m/z* calcd. for $C_{21}H_{27}O_3$ $[M+H]^+$: 327.1955; found: 327.1959. **UPC^2**: IC, CO_2/*i*PrOH 80/20, 3.0 mL·min^{-1}; t_{minor} = 4.4 min; t_{major} = 6.7 min.

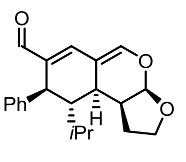

Product **5i** was obtained after 72 h following general procedure A performing the DA reaction at 80 °C. FC (pentane:EtOAc 20:1) afforded the pure compound in 42% yield, >20:1 dr and 99% ee. Yellow oil. $[\alpha]_D^{20}$ = –142.6 (*c*=0.8, CH_2Cl_2). **^{1}H NMR** (400 MHz, $CDCl_3$) δ 9.15 (s, 1H), 7.25 – 7.21 (m, 2H), 7.21 – 7.13 (m, 3H), 7.01 (s, 1H), 6.84 (d, *J* = 2.1 Hz, 1H), 5.59 (d, *J* = 3.8 Hz, 1H), 4.26 – 4.18 (m, 1H), 4.10 – 4.01 (m, 1H), 3.65 (d, *J* = 9.3 Hz, 1H), 2.85 – 2.74 (m, 1H), 2.69 – 2.57 (m, 1H), 2.10 – 1.92 (m, 2H), 1.91 – 1.69 (m, 2H), 0.98 (d, *J* = 7.2 Hz, 3H), 0.64 (d, *J* = 7.2 Hz, 3H). **^{13}C NMR** (100 MHz, $CDCl_3$) δ 191.2, 147.0, 144.8, 142.9, 137.9, 128.9 (2C), 128.1 (2C), 126.3, 110.5, 102.0, 68.5, 48.3, 42.2, 41.1, 30.9, 27.8, 22.7, 20.1, 19.1. **HRMS** (ESI+) *m/z* calcd. for $C_{21}H_{25}O_3$ $[M+H]^+$: 325.1798; found: 325.1806. **UPC^2**: IC, CO_2/*i*PrOH 60/40, 3.0 mL·min^{-1}; t_{major} = 4.5 min; t_{minor} = 5.4 min.

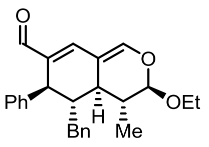

Product **5j** was obtained after 24 h following general procedure B. FC (pentane:EtOAc 80:1 to 40:1) afforded the pure compound in 75% yield, >20:1 dr and 99% ee.
Yellow oil. $[\alpha]_D^{20}$ = –36.8 (*c*=1.0, CH_2Cl_2). **^{1}H NMR** (400 MHz, $CDCl_3$) δ 9.36 (s, 1H), 7.25 – 7.04 (m, 11H), 6.83 (d, *J* = 2.0 Hz, 1H), 4.71 (d, *J* = 4.2 Hz, 1H), 3.76 (ddd, *J* = 16.6, 14.2, 6.7 Hz, 2H), 3.42 (dq, *J* = 9.6, 7.0 Hz, 1H), 2.91 – 2.81 (m, 1H), 2.66 – 2.55 (m, 2H), 1.94 (dtd, *J* = 11.4, 7.0, 3.4 Hz, 1H), 1.85 (ddd, *J* = 7.4, 4.8, 2.0 Hz, 1H), 1.12 (t, *J* = 7.1 Hz, 3H), 0.86 (d, *J* = 6.9 Hz, 3H). **^{13}C NMR** (100 MHz, $CDCl_3$) δ 191.6, 145.2, 144.4, 143.5, 140.1, 138.0, 129.4 (2C), 128.4 (2C), 128.2 (2C), 128.1 (2C), 126.1, 125.9, 116.0, 104.2, 64.3, 45.2, 45.0, 39.7, 39.0, 33.8, 16.8, 15.0. **HRMS** (ESI+) *m/z* calcd. for $C_{26}H_{29}O_3$ $[M+H]^+$: 389.2111; found: 389.2115. **UPC^2**: IC, CO_2/*i*PrOH 80/20, 3.0 mL·min^{-1}; t_{minor} = 4.9 min; t_{major} = 5.9 min.

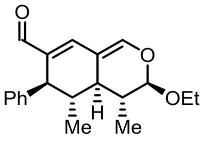

Product **5k** was obtained after 24 h following general procedure A. FC (pentane:EtOAc 40:1) afforded the pure compound in 68% yield, >20:1 dr and 95% ee. Yellow oil. $[\alpha]_D^{20}$ = -192.0 (*c*=1.0, CH_2Cl_2). **^{1}H NMR** (400 MHz, $CDCl_3$) δ 9.34 (s, 1H), 7.37 – 7.28 (m, 2H), 7.28 – 7.17 (m, 4H), 6.86 (s, 1H), 4.80 (d, *J* = 4.6 Hz, 1H), 3.91 (dq, *J* = 9.6, 7.0 Hz, 1H), 3.61 (dq, *J* = 9.6, 7.0 Hz, 1H), 3.38 (d, *J* = 9.6, 1H), 2.15 (qt, *J* = 6.9, 4.4 Hz, 1H), 2.00 – 1.86 (m, 1H), 1.77 (dd, *J* = 11.6, 4.3 Hz, 1H), 1.26 (t, *J* = 7.1 Hz, 3H), 1.19 (d, *J* = 6.9 Hz, 3H), 1.02 (d, *J* = 6.5 Hz, 3H). **^{13}C NMR** (100 MHz, $CDCl_3$) δ 191.9, 145.0, 144.0, 143.9, 138.7, 128.5 (2C), 128.1 (2C), 126.1, 116.3, 103.1, 64.4, 50.1, 42.4, 42.3, 33.7, 18.6, 16.8, 15.1. **HRMS** (ESI+) *m/z* calcd. for $C_{20}H_{25}O_3$ $[M+H]^+$: 313.1798; found: 313.1799. **UPC^2**: IC, CO_2/*i*PrOH 80/20, 3.0 mL·min^{-1}; t_{major} = 3.6 min; t_{minor} = 5.0 min.

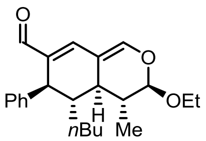

Product **5l** was obtained after 24 h following general procedure A. FC (pentane:EtOAc 80:1) afforded the pure compound in 79% yield, >20:1 dr and 99% ee. Yellow oil. $[\alpha]_D^{20}$ = −138.0 (*c*=1.0, CH_2Cl_2). **^{1}H NMR** (400 MHz, $CDCl_3$) δ 9.29 (s, 1H), 7.25 – 7.12 (m, 6H), 6.80 – 6.75 (m, 1H), 4.82 (d, *J* = 3.1 Hz, 1H), 3.77 (dq, *J* = 9.6, 7.1 Hz, 1H), 3.66 (d, *J* = 9.2 Hz, 1H), 3.49 (dq, *J* = 9.6, 7.0 Hz, 1H), 2.10 (dtt, *J* = 16.5, 8.4, 4.1 Hz, 2H), 1.80 (dt, *J* = 11.1, 2.4 Hz, 1H), 1.54 – 1.44 (m, 1H), 1.44 – 1.33 (m, 1H), 1.33 – 1.26 (m, 1H), 1.22 (ddt, *J* = 13.6, 6.6, 3.3 Hz, 3H), 1.15 (t, *J* = 7.1 Hz, 3H), 1.05 (d, *J* = 7.1 Hz, 3H), 0.85 (t, *J* = 7.2 Hz, 3H). **^{13}C NMR** (100 MHz, $CDCl_3$) δ 192.1, 145.3, 144.3, 143.2, 139.1, 128.3 (2C), 128.0 (2C), 126.0, 116.7, 102.9, 64.0, 45.6, 44.9, 39.0, 32.6, 28.5, 26.6, 23.0, 18.1, 15.1, 14.0.**HRMS** (ESI+) *m/z* calcd. for $C_{23}H_{30}O_3Na$ $[M+Na]^+$: 377.2087; found: 377.2081. **UPC^2**: IC, CO_2/*i*PrOH 80/20, 3.0 mL·min^{-1}; t_{minor} = 3.0 min; t_{major} = 3.8 min.

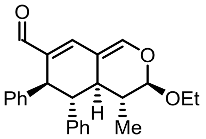

Product **5m** was obtained after 24 h following general procedure B, performing the first step at rt. FC (pentane:EtOAc 40:1 to 30:1) afforded the pure compound in 77% yield, >20:1 dr and 96% ee. Yellow oil. $[\alpha]_D^{20}$ = −52.2 (*c*=1.0, CH_2Cl_2). **^{1}H NMR** (400 MHz, $CDCl_3$) δ 9.33 (s, 1H), 7.25 – 7.23 (m, 1H), 7.21 – 7.13 (m, 3H), 7.08 – 7.01 (m, 3H), 6.94 – 6.85 (m, 3H), 6.79 – 6.73 (m, 2H), 4.68 (d, *J* = 3.9 Hz, 1H), 3.86 (d, *J* = 9.5 Hz, 1H), 3.81 (dq, *J* = 9.4, 7.4 Hz, 1H), 3.47 (dq, *J* = 9.5, 7.0 Hz, 1H), 2.91 (dd, *J* = 12.2, 10.2 Hz, 1H), 2.28 (d, *J* = 12.1, 1H), 1.69 (qt, *J* = 7.1, 3.8 Hz, 1H), 1.19 (t, *J* = 7.0 Hz, 3H), 0.68 (d, *J* = 7.0 Hz, 3H). **^{13}C NMR** (100 MHz, $CDCl_3$) δ 191.5, 145.0, 144.4, 142.9, 141.8, 138.7, 128.7 (2C), 128.2 (2C), 127.8 (2C), 127.7 (2C), 126.5, 125.8, 116.0, 103.1, 64.3, 55.6, 50.0, 40.9, 34.1, 17.7, 15.1. **HRMS** (ESI+) *m/z* calcd. for $C_{25}H_{27}O_3$ $[M+H]^+$: 375.1955; found: 375.1958. **UPC^2**: IC, CO_2/*i*PrOH 80/20, 3.0 mL·min^{-1}; t_{major} = 4.3 min; t_{minor} = 6.4 min.

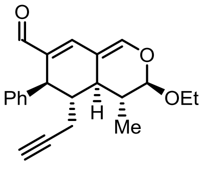

Product **5n** was obtained after 24 h following general procedure B. FC (pentane:EtOAc 50:1) afforded the pure compound in 47% yield, 17:1 dr and 96% ee. Yellow oil. $[\alpha]_D^{20}$ = −155.6 (*c*=1.0, CH_2Cl_2). **^{1}H NMR** (400 MHz, $CDCl_3$) δ 9.28 (s, 1H), 7.30 – 7.13 (m, 6H), 6.80 (s, 1H), 4.82 (d, *J* = 2.9 Hz, 1H), 3.84 (d, *J* = 9.9 Hz, 1H), 3.77 (m, 1H), 3.56 – 3.45 (m, 1H), 2.40 (dt, *J* = 17.6, 3.4 Hz, 1H), 2.21 – 1.95 (m, 5H), 1.15 (m, 6H).**^{13}C NMR** (100 MHz, $CDCl_3$) δ 191.7, 145.1, 143.6, 143.3, 138.7, 128.3 (2C), 128.2 (2C), 126.3, 116.0, 102.4, 81.0, 71.1, 64.1, 45.7, 44.9, 38.0, 32.7, 18.3, 18.1, 15.1. **HRMS** (ESI+) *m/z* calcd. for $C_{22}H_{25}O_3$ $[M+H]^+$: 337.1798; found: 337.1803. **UPC^2**: IC, CO_2/*i*PrOH 80/20, 3.0 mL·min^{-1}; t_{major} = 2.9 min; t_{minor} = 3.3 min.

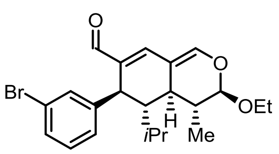

Product **5o** was obtained after 24 h following general procedure A. FC (pentane:EtOAc 50:1) afforded the pure compound in 65% yield, >20:1 dr and 98% ee. Yellow oil. $[\alpha]_D^{20}$ = 63.2 (*c*=1.0, CH_2Cl_2). **^{1}H NMR** (400 MHz, $CDCl_3$) δ 9.34 (s, 1H), 7.26 (s, 1H), 7.23 (dt, *J* = 6.7, 2.0 Hz, 1H), 7.17 (s, 1H), 7.10 – 7.02 (m, 2H), 6.85 (d, *J* = 2.0 Hz, 1H), 4.83 (d, *J* = 5.1 Hz, 1H), 3.91 – 3.81 (m, 2H), 3.54 (dq, *J* = 9.6, 7.1 Hz, 1H), 2.20 – 2.16 (m, 1H), 1.93 (td, *J* = 6.7, 2.0 Hz, 1H), 1.88 – 1.79 (m,2H), 1.21 (t, *J* = 7.1 Hz, 3H), 0.96 (d, *J* = 6.8 Hz, 3H), 0.92 (d, *J* = 7.0 Hz, 3H), 0.88 (d, *J* = 7.0 Hz, 3H). **^{13}C NMR** (100 MHz, $CDCl_3$) δ 191.1, 148.0, 145.8, 144.7, 138.5, 131.2, 129.6, 129.1, 127.2, 122.2, 115.7, 104.7, 64.5, 48.8, 40.3, 38.1, 35.3, 31.6, 20.3, 19.1, 16.2, 15.1. **HRMS** (ESI+) *m/z* calcd. for $C_{22}H_{28}BrO_3$ $[M+H]^+$: 419.1216; found: 419.1215. **UPC^2**: IC, CO_2/*i*PrOH 80/20, 3.0 mL·min^{-1}; t_{major} = 2.5 min; t_{minor} = 2.9 min.

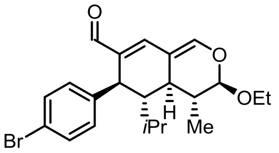

Product **5p** was obtained after 22 h following general procedure B. FC (pentane:EtOAc 60:1) afforded the pure compound in 88% yield, >20:1 dr and 99% ee. Yellow oil. $[\alpha]_D^{20}$ = 89.0 (*c*=1.0, CH_2Cl_2). **^{1}H NMR** (400 MHz, $CDCl_3$) δ 9.36 (s, 1H), 7.32 (s, 1H), 7.30 (s, 1H), 7.16 (s, 1H), 7.03 (s, 1H), 7.02 (s, 1H), 6.84 (d, *J* = 2.1 Hz, 1H), 4.83 (d, *J* = 5.2 Hz, 1H), 3.92 – 3.82 (m, 2H), 3.54 (dq, *J* = 9.5, 7.0 Hz, 1H), 2.18 – 2.15 (m, 1H), 1.97 – 1.75 (m, 3H), 1.21 (t, *J* = 7.1 Hz, 3H), 0.96 (d, *J* = 6.8 Hz, 3H), 0.92 (d, *J* = 6.9 Hz, 3H), 0.87 (d, *J* = 7.0 Hz, 3H). **^{13}C NMR** (100 MHz, $CDCl_3$) δ 191.1, 145.7, 144.7, 144.6, 138.9, 131.2 (2C), 130.1 (2C), 119.7, 115.8, 104.8, 64.6, 48.8, 40.1, 38.2, 35.4, 31.8, 20.3, 19.1, 16.2, 15.1. **HRMS** (ESI+) *m/z* calcd. for $C_{22}H_{28}BrO_3$ $[M+H]^+$: 419.1216; found: 419.1216. **UPC^2**: IC, CO_2/*i*PrOH 80/20, 3.0 mL·min^{-1}; t_{major} = 2.9 min; t_{minor} = 3.5 min.

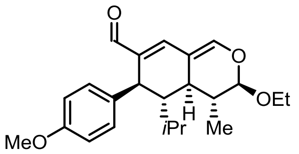

Product **5q** was obtained after 72 h following general procedure B. FC (pentane:EtOAc 30:1) afforded the pure compound in 71% yield, >20:1 dr and 99% ee. Yellow oil. $[\alpha]_D^{20}$ = 45.6 (*c*=1.0, CH_2Cl_2). **^{1}H NMR** (400 MHz, $CDCl_3$) δ 9.34 (s, 1H), 7.13 (s, 1H), 7.03 (s, 1H), 7.02 (s, 1H), 6.82 (d, *J* = 2.1 Hz, 1H), 6.74 (s, 1H), 6.72 (s, 1H), 4.81 (d, *J* = 5.8 Hz, 1H), 3.92 – 3.83 (m, 2H), 3.74 (s, 3H), 3.54 (dq, *J* = 9.6, 7.1 Hz, 1H), 2.15 (dt, *J* = 6.4, 4.0 Hz, 1H), 1.95 (td, *J* = 6.9, 2.2 Hz, 1H), 1.84 – 1.73 (m, 2H), 1.21 (t, *J* = 7.1 Hz, 3H), 0.93 (dd, *J* = 6.9, 4.9 Hz, 6H), 0.86 (d, *J* = 6.9 Hz, 3H). **^{13}C NMR** (100 MHz, $CDCl_3$) δ 191.4, 157.7, 145.2, 144.5, 139.4, 137.4, 129.0 (2C), 116.0, 113.6 (2C), 105.2, 64.7, 55.1, 48.5, 39.3, 38.3, 35.7, 32.3, 20.4, 19.1, 15.9, 15.1. **HRMS** (ESI+) *m/z* calcd. for $C_{23}H_{31}O_4$ $[M+H]^+$: 371.2217; found: 371.2220. **UPC^2**: IC, CO_2/*i*PrOH 80/20, 3.0 mL·min^{-1}; t_{minor} = 3.7 min; t_{major} = 5.0 min.

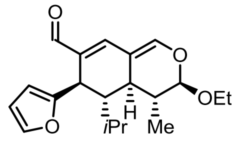

Product **5r** was obtained after 24 h following general procedure B. FC (pentane:EtOAc 40:1) afforded the pure compound in 65% yield, >20:1 dr and 99% ee. Yellow oil. $[\alpha]_D^{20}$ = -146.6 (*c*=1.0, CH_2Cl_2). **^{1}H NMR** (400 MHz, $CDCl_3$) δ 9.48 (s, 1H), 7.24 (dd, *J* = 1.9, 0.9 Hz, 1H), 7.16 (s, 1H), 6.82 (d, *J* = 2.2 Hz, 1H), 6.17 (dd, *J* = 3.2, 1.9 Hz, 1H), 5.74 (dt, *J* = 3.2, 0.9 Hz, 1H), 4.80 (d, *J* = 7.3 Hz, 1H), 4.15 (d, *J* = 3.3 Hz, 1H), 3.88 (dq, *J* = 9.6, 7.1 Hz, 1H), 3.55 (dq, *J* = 9.6, 7.1 Hz, 1H), 2.32 (dt, *J* = 5.3, 3.5 Hz, 1H), 2.05 (td, *J* = 9.5, 2.6 Hz, 1H), 1.69 –1.61 (m, 1H), 1.44 (dq, *J* = 9.8, 6.8 Hz, 1H), 1.21 (t, *J* = 7.1 Hz, 3H), 0.97 (d, *J* = 6.7 Hz, 3H), 0.93 (d, *J* = 6.8 Hz, 3H), 0.88 (d, *J* = 6.9 Hz, 3H). **^{13}C NMR** (100 MHz, $CDCl_3$) δ 190.9, 156.2, 146.5, 146.4, 140.9, 135.5, 115.5, 110.3, 106.8, 105.6, 64.9, 43.4, 37.8, 36.2, 33.2, 32.6, 20.6, 19.6, 15.0 (2C). **HRMS** (ESI+) *m*/*z* calcd. for $C_{20}H_{26}O_4Na$ $[M+Na]^+$: 353.1723; found: 353.1722. **UPC^2**: IA, Gradient CO_2/*i*PrOH, first 99/1 in 0.5 min, then 99/1 → 60/40 (10%/min), 3.0 mL·min^{-1}; t_{major} = 2.7 min; t_{minor} = 2.8 min.

5. Synthesis of the tetrahydroisochromenes 6a-e

5.1. General Procedure C

In a 4 mL glass vial catalyst (*S*)-**A** (0.04 mmol, 0.2 equiv.) and (2*E*)-4-benzylidenepent-2-enedial **1a** (0.2 mmol, 1.0 equiv.) were dissolved in $CHCl_3$ (0.4 mL), followed by the addition of aldehyde **2** (0.6 mmol, 5.0 equiv.). The reaction mixture was heated to 40 °C until full conversion of **1a** into intermediate. Full conversion was determined by a control experiment performed at 0.05 mmol scale, from which the reaction progress was monitored by NMR. Next, catalyst (*R*)-**A** (0.04 mmol, 0.2 equiv.) was added and the reaction was set at rt until full conversion into the desired product was reached. Then the crude reaction mixture was loaded on a column and purified by FC (EtOAc/pentane) yielding the corresponding product **6**.

5.2. Results and characterization

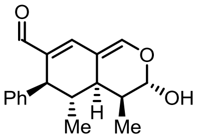

Product **6a** was obtained after 24 h following procedure C, employing 3.0 equiv. of aldehyde **2**, without adding the other enantiomer of the catalyst and running the second step at 40 °C. FC (pentane:EtOAc 5:1) afforded the pure compound in 73% yield, 7:1 dr and 99% ee. Yellow solid. **^{1}H NMR** (400 MHz, $CDCl_3$) δ 9.22 (s, $1H^{+}$), 7.31 – 7.05 (m, $6H^{+}$), 6.82 (d, *J* = 2.1 Hz, 1H*), 6.75 (d, *J* = 2.2 Hz, 1H), 5.36 (d, *J* = 1.8 Hz, 1H*), 5.32 (s, 1H), 3.32 – 3.25(m, 1H), 3.25 – 3.21 (m, 1H*), 2.50 (ddd, *J* = 12.3, 5.4, 2.2 Hz, 1H), 2.40 (ddd, *J* = 11.7, 4.7 Hz, 1.8 Hz, 1H*), 2.37 – 2.28 (m, 1H*), 2.27 – 2.19 (m, 1H), 1.64 – 1.51 (m, $1H^{+}$), 0.90 – 0.81 (m, $6H^{+}$). **^{13}C NMR** (100 MHz, $CDCl_3$) δ 191.8^{+}, 145.2^{+}, 145.1^{+}, 143.7, 143.5*, 137.3^{+}, 128.5*, 128.4*, 128.2, 128.1 ($2C^{+}$), 128.1, 126.2*, 126.1, 113.1, 112.5*, 98.3*, 97.3, 48.3, 48.0*, 40.4*, 37.5*, 36.7, 33.1, 32.5*, 30.9, 15.4*, 15.3, 9.9, 3.8*.**HRMS** (ESI+) *m/z* calcd. for $C_{18}H_{20}O_3Na$ $[M+Na]^+$: 307.1305; found: 307.1307. **UPC^2**: IA, Gradient CO_2/MeOH, first 99/1 in 0.5 min, then 99/1 → 60/40 (10%/min), 3.0 mL·min^{-1}; t_{major} = 4.6 min; t_{minor} = 4.1 min.

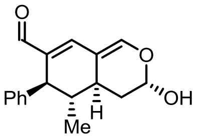

Product **6b** was obtained after 24 h following general procedure C with some modifications. In the first step, 1.2 equiv. of **1a** was used along with 1 equiv. of propionaldehyde **2c** (added by weight). After 3 h, acetaldehyde **2g** (5.0 equiv.) was added as part of the second step, and the reaction was stirred at 40 °C. Furthermore, there were no addition of the other enantiomer of the catalyst. FC (pentane:EtOAc 6:1) afforded the pure compound in 60% yield, 2:1 dr and 99% ee. Yellow solid. **^{1}H NMR** (400 MHz, $CDCl_3$) δ 9.21 (s, $1H^{+}$), 7.30 – 7.03 (m, $6H^{+}$), 6.84 (d, J = 1.0 Hz, 1H*), 6.79 (d, *J* = 2.0 Hz, 1H), 5.61 (s, 1H), 5.33 (dd, *J* = 10.2, 3.7 Hz, 1H*), 3.83 – 3.73 (m, 1H*), 3.58 – 3.50 (m, 1H), 3.28 (d, *J* = 10.0 Hz, 1H), 3.23 (d, *J* = 10.1 Hz, 1H*), 2.43 – 2.18 (m, $2H^{+}$), 1.50 – 1.34 (m, $2H^{+}$), 0.92 – 0.85 (m, $3H^{+}$). **^{13}C NMR** (100 MHz, $CDCl_3$) δ 191.8, 191.6*, 147.2*, 145.6, 144.2, 143.8*, 143.7, 143.4*, 137.8^{+}, 128.3 ($2C^{+}$), 128.2 (2C*), 128.1 (2C), 126.2^{+}, 115.3, 114.3*, 96.0*, 92.4, 48.3, 48.2*, 42.3*, 41.7, 35.8*, 34.4*, 31.1, 29.6, 16.0*, 15.9. **HRMS** (ESI+) *m/z* calcd. for $C_{17}H_{19}O_3$ $[M+H]^+$: 271.1329; found: 271.1326. **UPC^2**: IA, Gradient CO_2/MeOH, first 99/1 in 0.5 min, then 99/1 → 60/40 (10%/min), 3.0 mL·min^{-1}; t_{major} = 4.5 min; t_{minor} = 4.2 min.

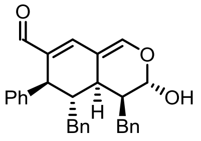

Product **6c** was obtained after 36 h following general procedure C. FC (pentane:EtOAc 6:1) afforded the pure compound in 49% yield, >20:1 dr and 99% ee. Yellow solid. $[\alpha]_D^{20}$ = +86.6 (*c*=1.0, CH_2Cl_2). **^{1}H NMR** (400 MHz, $CDCl_3$) δ 9.21 (s, 1H), 7.32 (ddd, *J* = 14.9, 8.3, 6.7 Hz, 4H), 7.25 – 7.10 (m, 7H), 7.09 – 7.00 (m, 3H), 6.84 (d, *J* = 2.3 Hz, 1H), 6.73 – 6.65 (m, 2H), 4.99 (s, 1H), 3.53 (d, *J* = 10.2, 1H), 3.15 – 3.05 (m, 2H), 2.97 (ddd, *J* = 12.4, 4.4, 2.2 Hz, 1H), 2.56 (dd, *J* = 14.7, 6.6 Hz, 1H), 2.48 – 2.38 (m, 2H), 2.33 – 2.22 (m, 1H), 2.17 (t, *J* = 13.8 Hz, 1H). **^{13}C NMR** (100 MHz, $CDCl_3$) δ 191.5, 145.7, 143.8 (2C), 143.6, 140.3, 138.5, 137.2, 129.3 (2C), 129.2 (3C), 128.5 (2C), 128.4 (2C), 128.2 (2C), 126.5, 126.2, 126.2, 113.4, 93.6, 45.8, 42.0, 37.6, 37.2, 33.0, 30.6. **HRMS** (ESI+) *m/z* calcd. for $C_{30}H_{28}O_3Na$ $[M+Na]^+$: 359.1931; found: 359.1934. **UPC^2**: IA, Gradient CO_2/MeOH, first 99/1 in 0.5 min, then 99/1 → 60/40 (10%/min), 3.0 mL·min^{-1}; t_{major} = 6.3 min; t_{minor} = 5.5 min.

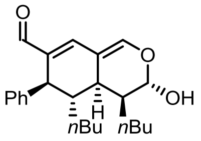

Product **6d** was obtained after 72 h following general procedure C. FC (pentane:EtOAc 5:1) afforded the pure compound in 45% yield, >20:1 dr and 99% ee. Yellow solid. $[\alpha]_D^{20}$ = +32.8 (*c*=1.0, CH_2Cl_2). **^{1}H NMR** (400 MHz, $CDCl_3$) δ 9.21 (s, 1H), 7.33 – 7.08 (m, 5H), 7.02 (d, *J* = 2.0 Hz, 1H), 6.74 (d, *J* = 2.3 Hz, 1H), 5.57 – 5.53 (d, 1.5 Hz, 1H), 3.62 (d, *J* = 10.3 Hz, 1H), 3.34 (bs, 1H), 2.78 (ddd, *J* = 12.8, 5.3, 2.3 Hz, 1H), 2.02 – 1.91 (m, 1H), 1.79 – 1.67 (m, 1H), 1.49 – 1.10 (m, 12H), 0.88 (dt, *J* = 10.3, 6.8 Hz, 6H). **^{13}C NMR** (100 MHz, $CDCl_3$) δ 191.6, 145.0, 144.3, 143.9, 138.0, 128.2 (4C), 126.1, 114.4, 94.2, 43.7, 40.0, 35.5, 29.4, 28.9, 25.9, 24.9, 23.6, 23.3, 22.9, 14.0, 14.0. **HRMS** (ESI+) *m/z* calcd. for $C_{24}H_{32}O_3Na$ $[M+Na]^+$: 391.2244; found: 391.2244. **UPC^2**: IA, Gradient CO_2/MeOH, first 99/1 in 0.5 min, then 99/1 → 60/40 (10%/min), 3.0 mL·min^{-1}; t_{major} = 4.3 min; t_{minor} = 4.1 min.

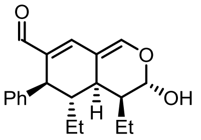

Product **6e** was obtained after 48 h following general procedure C. FC (pentane:EtOAc 5:1) afforded the pure compound in 47% yield, >20:1 dr and 99% ee. Yellow solid. $[\alpha]_D^{20}$ = +78.2 (*c*=1.0, CH_2Cl_2). **^{1}H NMR** (400 MHz, $CDCl_3$) δ 9.22 (s, 1H), 7.28 (d, *J* = 7.4 Hz, 2H), 7.22 – 7.10 (m, 3H), 7.05 – 6.98 (m, 1H), 6.75 (d, *J* = 2.3 Hz, 1H), 5.60 – 5.54 (m, 1H), 3.62 (d, *J* = 10.8, 1H), 3.16 (d, *J* = 3.9 Hz, 1H), 2.85 – 2.74 (m, 1H), 1.94 – 1.85 (m, 1H), 1.74 (tt, *J* = 10.5, 3.7 Hz, 1H), 1.48 – 1.38 (m, 1H), 1.37 – 1.09 (m, 3H), 0.97 (t, *J* = 7.5 Hz, 3H), 0.88 (t, *J* = 7.5 Hz, 3H). **^{13}C NMR** (100 MHz, $CDCl_3$) δ 191.7, 145.2, 144.3, 143.8, 137.9, 128.2 (4C), 126.1, 114.3, 93.9, 42.8, 40.2, 37.1, 28.3, 18.4, 16.8, 11.7, 7.0. **HRMS** (ESI+) *m/z* calcd. for $C_{20}H_{24}O_3Na$ $[M+Na]^+$: 335.1618; found: 335.1618. **UPC^2**: IA, Gradient CO_2/MeOH, first 99/1 in 0.5 min, then 99/1 → 60/40 (10%/min), 3.0 mL·min^{-1}; t_{major} = 4.1 min; t_{minor} = 3.9 min.

6. Transformations

6.1. Synthesis of (*R*)-4,5-Dimethyl-3-oxo-6-phenyl-4,4a,5,6-tetrahydro-3H-$4\lambda^3,5\lambda^3,6\lambda^3$-isochromene-7-carbaldehyde 7

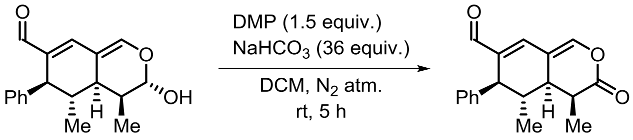

6a **7**

Procedure: A flame dried 25 mL flask was charged with **6a** (0.2 mmol, 1.0 equiv.) and a magnetic stir bar, followed by the addition of $NaHCO_3$ (7.2 mmol, 36.0 equiv.) and CH_2Cl_2 (10 mL). Next, DMP was added and the flask was fitted with a septum and flushed with N_2. The reaction was set to react at rt. Upon full conversion (TLC, pentane:EtOAc 4:1), Et_2O (5 mL) was added to the reaction and the mixture was filtered and concentrated *in vacuo*. The crude was purified by FC (pentane:EtOAc 5:1) yielding the oxidized product **7** in 38% yield and >20:1 dr.

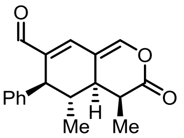

^{1}H NMR (400 MHz, $CDCl_3$) δ 9.27 (s, 1H), 7.31 (td, *J* = 7.4, 7.0, 1.2 Hz, 2H), 7.26 – 7.21 (m, 1H), 7.17 – 7.07 (m, 3H), 7.0 (d, *J* = 2.9 Hz, 1H), 3.30 – 3.22 (m, 1H), 3.09 (qd, *J* = 7.2, 5.3 Hz, 1H), 2.66 (ddd, *J* = 11.9, 5.5, 2.8 Hz, 1H), 1.73 (ddq, *J* = 12.9, 10.1, 6.5 Hz, 1H), 1.20 (d, *J* = 7.3 Hz, 3H), 0.86 (d, *J* = 6.4 Hz, 3H). **^{13}C NMR** (100 MHz, $CDCl_3$) δ 191.1, 170.6, 142.5, 142.2, 140.8, 138.9, 128.5 (4C), 126.8, 115.5, 47.6, 38.1, 36.5, 35.2, 15.1, 10.1. **HRMS** (ESI+) *m/z* calcd. for $C_{18}H_{19}O_3Na$ $[M+H]^+$: 283.1329; found: 283.1329.

6.2. Synthesis of (*R*)-3-Ethoxy-4,5-dimethyl-6-phenyl-4,4a,5,6-tetrahydro-3H-$3\lambda^3,4\lambda^3,5\lambda^3,6\lambda^3$-isochromene-7-carbaldehyde 8

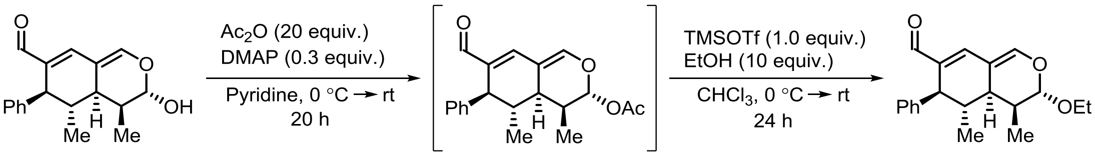

6a **8**

Procedure: In a 12 mL glass vial, **6a** (0.91 mmol, 1.0 equiv.) was dissolved in pyridine (1.8 mL). The solution was cooled to 0 °C after which Ac_2O (18.2 mmol, 20 equiv.) was added. Following the addition of DMAP (0.27 mmol, 0.3 equiv), the reaction mixture was set at rt and left to react overnight. By means of a TLC analysis (pentane:EtOAc 4:1), full conversion into the acetylated reaction intermediate was achieved after 20 h. The crude was plugged through silica (pentane:EtOAc 9:1 → 3:1), affording the intermediate nearly clean as a yellow oil.

Next, the intermediate (0.6 mmol, 1.0 equiv.) was dissolved in $CHCl_3$ (1.4 mL) followed by the addition of EtOH (6.0 mmol, 10 equiv.). The reaction mixture was cooled to 0 °C, and then TMSOTf (0.6 mmol, 1.0 equiv.) was added. After 5 h at 0 °C, the reaction was allowed to heat to rt and stirred overnight. Full conversion was achieved after 24 h, as found by TLC analysis (pentane:EtOAc 4:1). The crude was loaded

directly on a column, and purified by FC (pentane:EtOAc 20:1 → 10:1) yielding **8** as a yellow oil, in 7:1 dr and 57% yield over two steps.

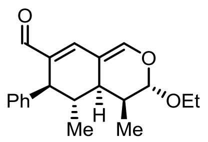

Only signals of the major diastereoisomer is reported: **^{1}H NMR** (400 MHz, $CDCl_3$) δ 9.21 (s, 1H), 7.31 – 7.09 (m, 5H), 7.06 (d, *J* = 1.9 Hz, 1H), 6.73 (d, *J* = 2.3 Hz, 1H), 4.94 (d, *J* = 1.8 Hz, 1H), 3.85 (dq, *J* = 9.8, 7.1 Hz, 1H), 3.64 (dq, *J* = 9.8, 7.1 Hz, 1H), 3.30 – 3.24 (m, 1H), 2.47 (ddd, *J* = 12.2, 5.4, 2.2 Hz, 1H), 2.20 (qdd, *J* = 7.2, 5.3, 1.9 Hz, 1H), 1.55 (ddq, *J* = 12.9, 9.9, 6.5 Hz, 1H), 1.23 (t, *J* = 7.1 Hz, 3H), 0.85 (d, *J* = 1.0 Hz, 3H), 0.84 (d, *J* = 1.7 Hz, 3H). **^{13}C NMR** (100 MHz, $CDCl_3$) δ 191.5, 145.1, 145.0, 143.9, 137.3, 128.1 (4C), 126.1, 113.6, 102.9, 64.2, 48.3, 36.8, 33.9, 30.9, 15.3, 15.1, 10.0. **HRMS** (ESI+) *m/z* calcd. for $C_{20}H_{24}O_3Na$ $[M+Na]^+$: 335.1618; found: 335.1622.

7. X-Ray structures

7.1. Oxadendralenic dienal 1a

Item	Value
Molecular formula	$C_{12}H_{10}O_2$
Formula weight	186.2
Crystal system	orthorhombic
Space Group	P 2_1 2_1 2_1
a (Å)	7.0175
b (Å)	11.1406
c (Å)	11.9874
α (°)	90
β (°)	90
γ (°)	90
Volume (Å^3)	937.18
Z	4
T (K)	110
ρ (g cm^{-1})	1.32
λ (Å)	0.71073
μ (mm^{-1})	0.089
# measured refl	7396
# unique refl	2232
R_{int}	0.0635
# parameters	127
R(F^2), all refl	0.076
$R_w(F^2)$, all refl	0.1312
Goodness of fit	1.067

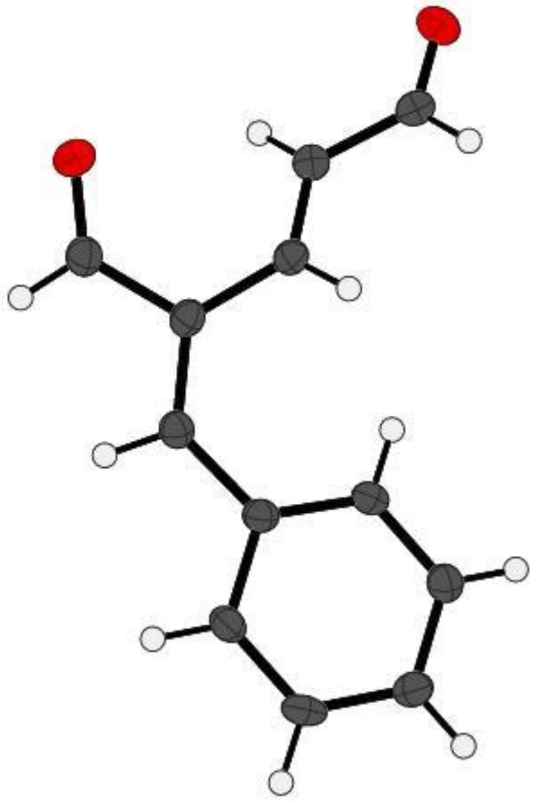

Crystal data for [**1a**]: $C_{12}H_{10}O_2$, *M* = 186.2, orthorhombic, space group P $2_12_12_1$ (no. 115), *a* = 7.0175(4) Å, *b* = 11.1406(6) Å, *c* = 11.9874(6) Å, Flack parameter = -0.5, *V* = 937.18(8) Å^3, *T* = 110 K, *Z* = 4, d_c = 1.32 g cm^{-3}, *μ*(Mo Kα, λ = 0.71073 Å) = 0.089 mm^{-1}, 7396 reflections collected, 2232 unique [R_{int} = 0.0635], which were used in all calculations. Refinement on F^2, final R(F) = 0.076, $R_w(F^2)$ = 0.1312. CCDC number 1419567.

7.2. Intermediate 3b

Item	Value
Molecular formula	$C_{15}H_{13}BrO_2$
Formula weight	305.16
Crystal system	orthorhombic
Space Group	$P\ 2_1\ 2_1\ 2_1$
a (Å)	6.1506
b (Å)	7.564
c (Å)	28.362
α (°)	90
β (°)	90
γ (°)	90
Volume (Å^3)	1319.5
Z	4
T (K)	100
ρ (g cm^{-1})	1.536
λ (Å)	0.56086
μ (mm^{-1})	1.669
# measured refl	5932
# unique refl	1902
R_{int}	0.071
# parameters	164
R(F^2), all refl	0.047
$R_w(F^2)$, all refl	0.0739
Goodness of fit	1.003

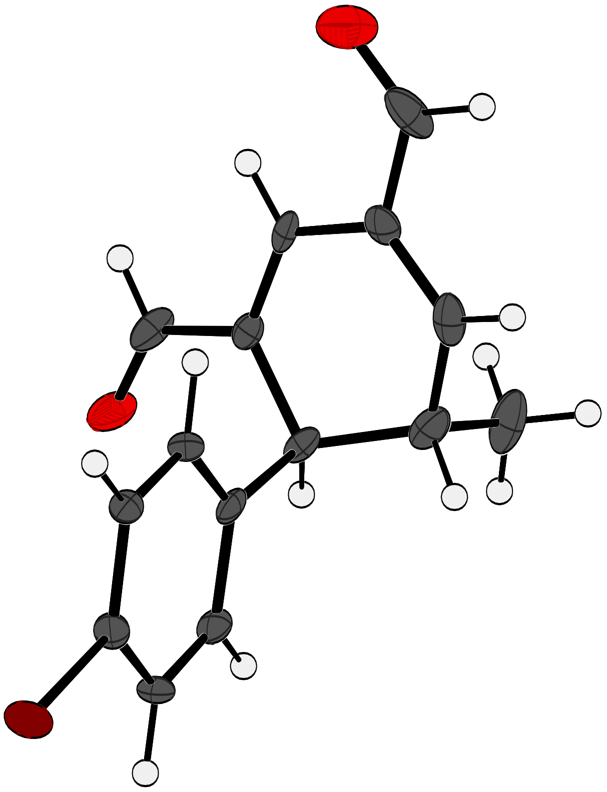

Crystal data for [**3b**]: $C_{15}H_{13}BrO_2$, M = 305.16, orthorhombic, space group P $2_12_12_1$ (no. 115), a = 6.1506(18) Å, b = 7.564(2) Å, c = 28.362(8) Å, Flack parameter = 0.01, V = 1319.5(6) Å^3, T = 100 K, Z = 4, d_c = 1.536 g cm^{-3}, μ(Mo Kα, λ = 0.56086 Å) = 1.669 mm^{-1}, 5932 reflections collected, 1902 unique [R_{int} = 0.071], which were used in all calculations. Refinement on F^2, final R(F) = 0.047, $R_w(F^2)$ = 0.0739. CCDC number 1408759.

7.3. Tetrahydroisochromene 5b

Item	Value
Molecular formula	$C_{22}H_{28}O_3$
Formula weight	340.47
Crystal system	orthorhombic
Space Group	$P\ 2_1\ 2_1\ 2_1$
a (Å)	7.5376
b (Å)	10.5784
c (Å)	23.8774
α (°)	90
β (°)	90
γ (°)	90
Volume (Å^3)	1903.9
Z	4
T (K)	100
ρ (g cm^{-1})	1.1877
λ (Å)	0.71073
μ (mm^{-1})	0.077
# measured refl	7273
# unique refl	3091
R_{int}	0.0793
# parameters	229
R(F^2), all refl	0.1427
R_w(F^2), all refl	0.13
Goodness of fit	1.0032

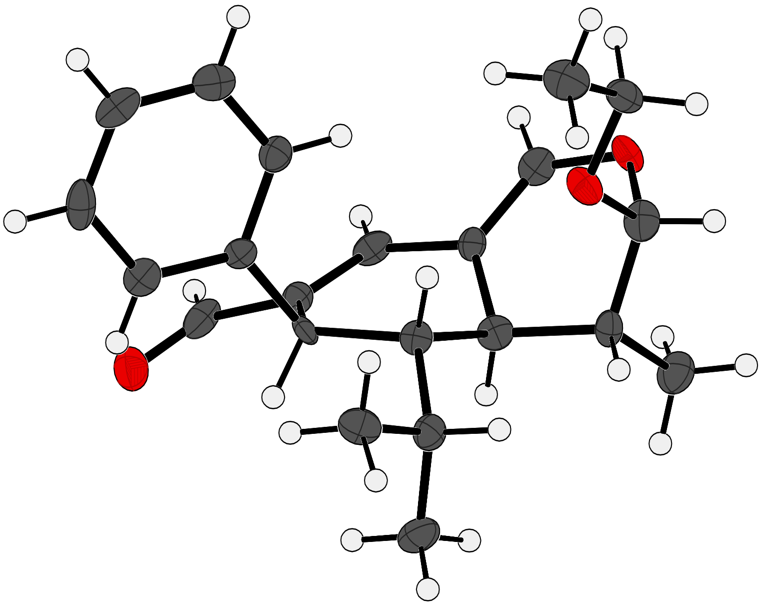

Crystal data for [**5b**]: $C_{22}H_{28}O_3$, *M* = 340.47, orthorhombic, space group $P\ 2_1\ 2_1\ 2_1$ (no. 115), *a* = 7.5376(6) Å, *b* = 10.5784(10) Å, *c* = 23.8774(19) Å, *V* = 1903.9(3) Å^3, *T*= 100 K, *Z* = 4, d_c = 1.1877 g cm^{-3}, *μ*(Mo Kα, λ = 0.71073 Å) = 0.077 mm^{-1}, 7273 reflections collected, 3091 unique [R_{int} = 0.0793], which were used in all calculations. Refinement on F^2, final R(F) = 0.1427, R_w(F^2) = 0.13. CCDC number 1405309.

7.4. Tetrahydroisochromene 5i

Item	Value
Molecular formula	$C_{21}H_{24}O_3$
Formula weight	324.4
Crystal system	monoclinic
Space Group	$P\ 2_1$
a (Å)	8.8945
b (Å)	21.16
c (Å)	9.0481
α (°)	90
β (°)	91.63
γ (°)	90
Volume ($Å^3$)	1702.24
Z	4
T (K)	100
ρ (g cm^{-1})	1.266
λ (Å)	0.71073
μ (mm^{-1})	0.083
# measured refl	11096
# unique refl	5072
R_{int}	0.0268
# parameters	437
$R(F^2)$, all refl	0.0451
$R_w(F^2)$, all refl	0.0847
Goodness of fit	1.074

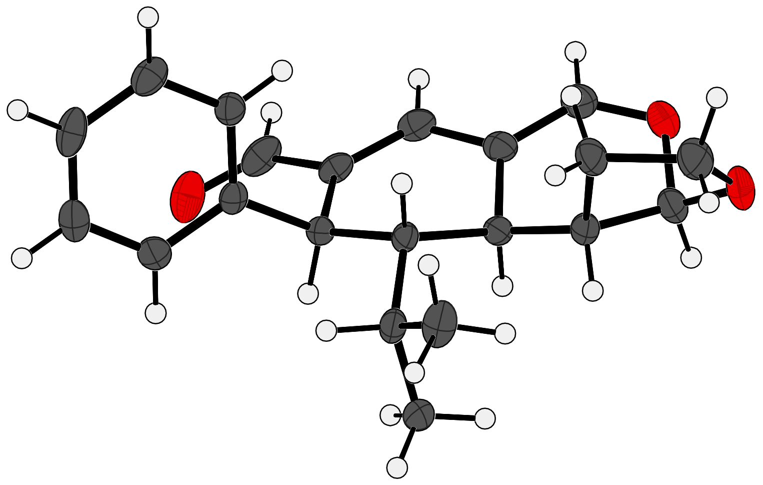

Crystal data for [**5i**]: $C_{21}H_{24}O_3$, M = 324.4, monoclinic, space group P 2_1 (no. 6), a = 8.8945(2) Å, b = 21.16(5) Å, c = 9.0481(2) Å, β = 91.63(19)°, V = 1702.24(7) $Å^3$, T= 100 K, Z = 4, d_c = 1.266 g cm^{-3}, μ(Mo Kα, λ = 0.71073 Å) = 0.083 mm^{-1}, 11096 reflections collected, 5072 unique [R_{int} = 0.0268], which were used in all calculations. Refinement on F^2, final R(F) = 0.0451, $R_w(F^2)$ = 0.0847. CCDC number 1405275.

7.5. Tetrahydroisochromene 6a

Item	Value
Molecular formula	$C_{18}H_{20}O_3$
Formula weight	284.36
Crystal system	monoclinic
Space Group	P 2_1
a (Å)	7.503
b (Å)	10.304
c (Å)	10.253
α (°)	90
β (°)	92.82
γ (°)	90
Volume (Å^3)	791.7
Z	2
T (K)	293
ρ (g cm^{-1})	1.1928
λ (Å)	0.71073
μ (mm^{-1})	0.08
# measured refl	5304
# unique refl	2910
R_{int}	0.1847
# parameters	192
R(F^2), all refl	0.2768
R_w(F^2), all refl	0.3441
Goodness of fit	0.959

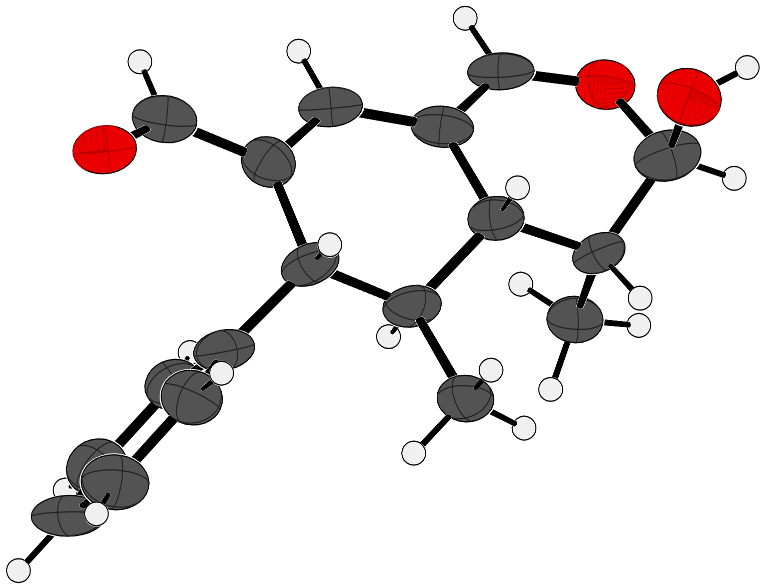

Crystal data for [**6a**]: $C_{18}H_{20}O_3$, M = 284.36, monoclinic, space group P 2_1 (no. 6), a = 7.503(3) Å, b = 10.304(5) Å, c = 10.253(5) Å, β = 92.82(4)°, Flack parameter = -3.6, V = 791.7(6) Å^3, T = 293 K, Z = 2, d_c = 1.1928 g cm^{-3}, μ(Mo Kα, λ = 0.71073 Å) = 0.08 mm^{-1}, 5304 reflections collected, 2910 unique [R_{int} = 0.1847], which were used in all calculations. Refinement on F^2, final R(F) = 0.2768, R_w(F^2) = 0.3441. CCDC number 1405277.

7.6. Tetrahydroisochromene 6e

Item	Value
Molecular formula	$C_{20}H_{24}O_3$
Formula weight	312.41
Crystal system	orthorhombic
Space Group	P 2_1 2_1 2_1
a (Å)	8.6911
b (Å)	9.1112
c (Å)	21.5901
α (°)	90
β (°)	90
γ (°)	90
Volume (Å^3)	1709.64
Z	4
T (K)	100
ρ (g cm^{-1})	1.2137
λ (Å)	0.71073
μ (mm^{-1})	0.08
# measured refl	32900
# unique refl	5923
R_{int}	0.1353
# parameters	210
R(F^2), all refl	0.1907
R_w(F^2), all refl	0.2308
Goodness of fit	0.9793

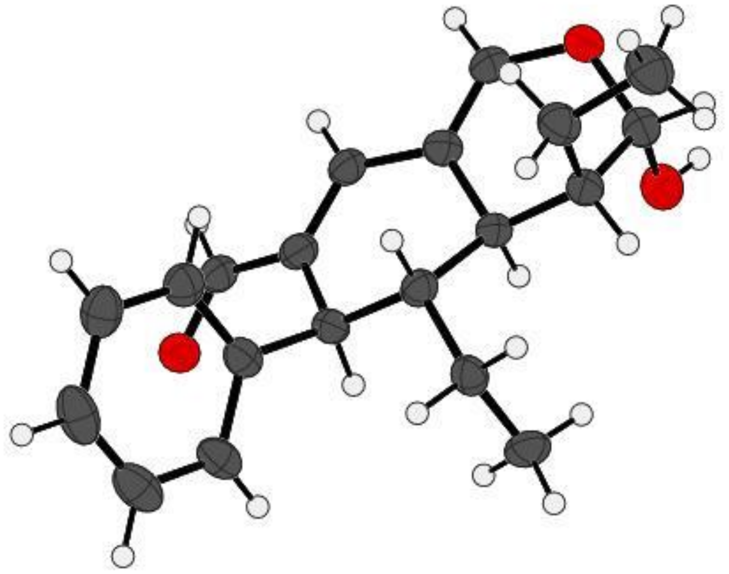

Crystal data for [**6e**]: $C_{20}H_{24}O_3$, *M* = 312.41, orthorhombic, space group P 2_1 2_1 2_1 (no. 115), *a* = 8.6911(5) Å, *b* = 9.1112(4) Å, *c* = 21.5901(10) Å, Flack parameter = -1.7,*V* = 1709.64(14) Å^3, *T* = 100 K, *Z* = 4, d_c = 1.2137 g cm^{-3}, μ(Mo Kα, λ = 0.71073 Å) = 0.08 mm^{-1}, 32900 reflections collected, 5923 unique [R_{int} = 0.1353], which were used in all calculations. Refinement on F^2, final R(F) = 0.1907, R_w(F^2) = 0.2308. CCDC number 1419566.

8. NMR spectra of novel compounds

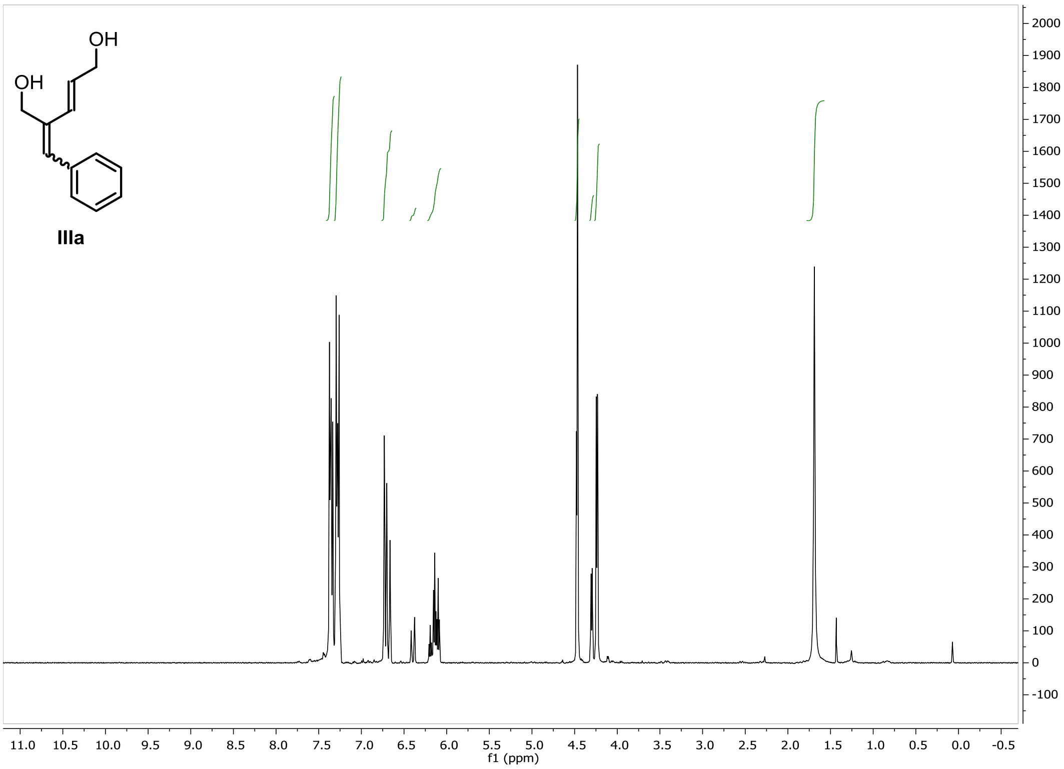

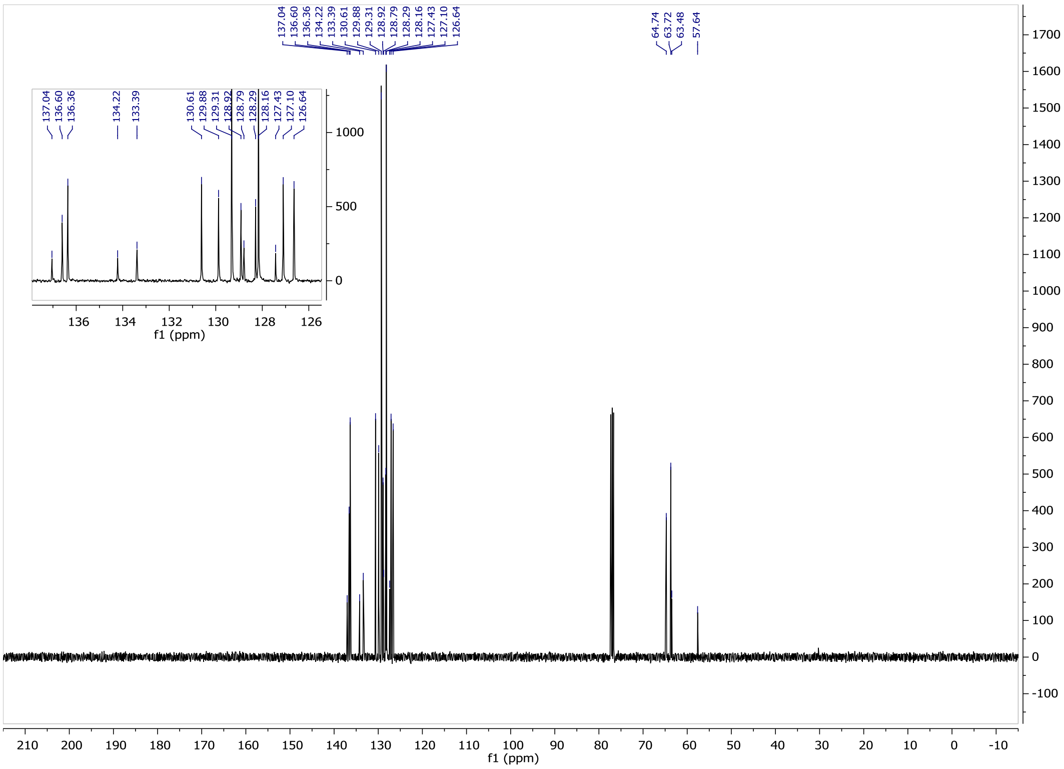

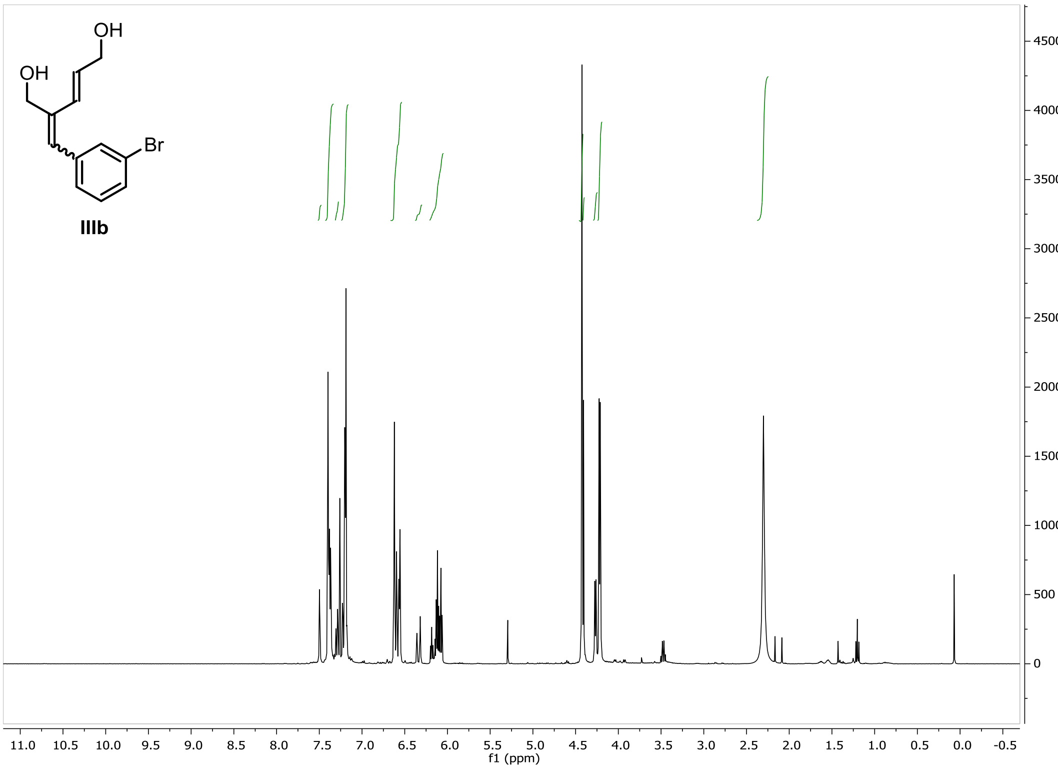
OH
OH
Br
IIIb
f1 (ppm)

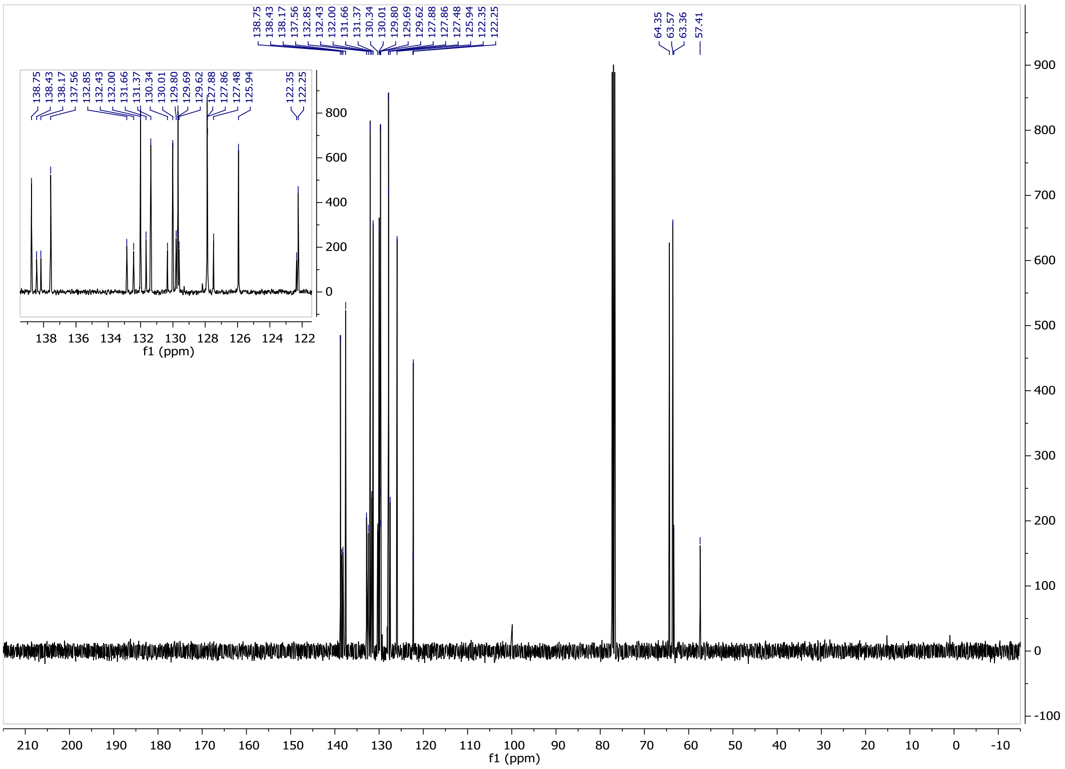
138.75
138.43
138.17
137.56
132.85
132.43
132.00
131.66
131.37
130.34
130.01
129.80
129.69
129.62
127.88
127.86
127.48
125.94
122.35
122.25
64.35
63.57
63.36
57.41
f1 (ppm)

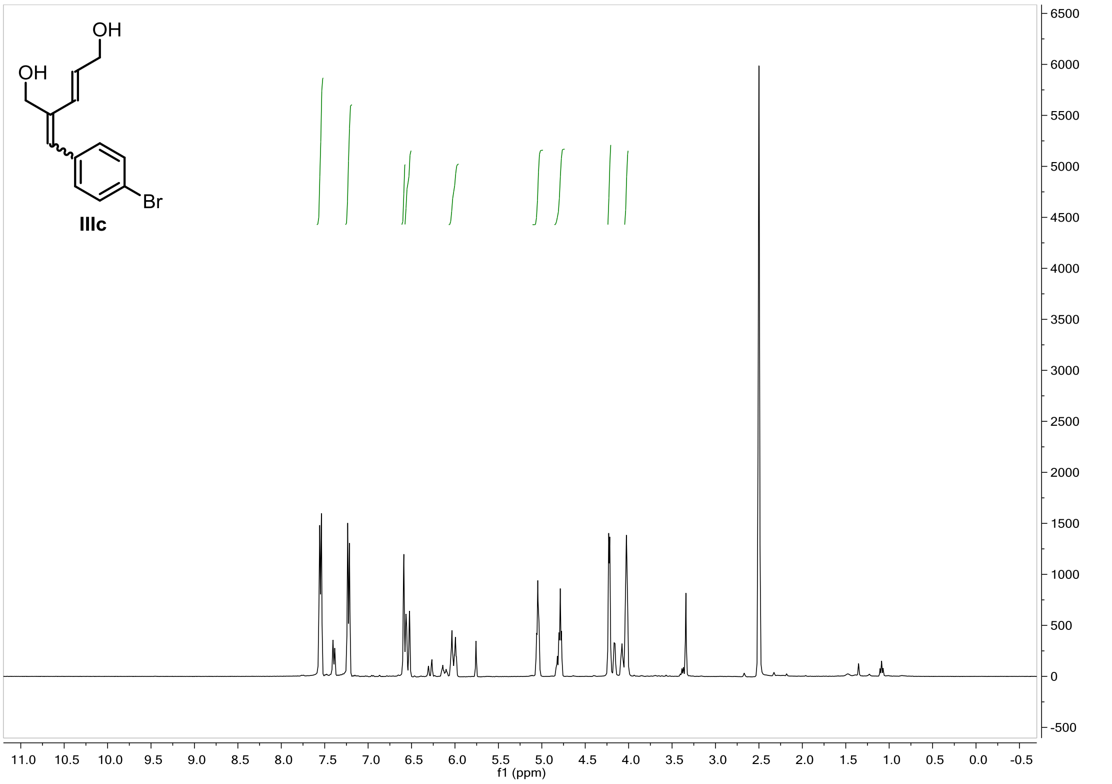
OH
OH
Br
IIIc
f1 (ppm)

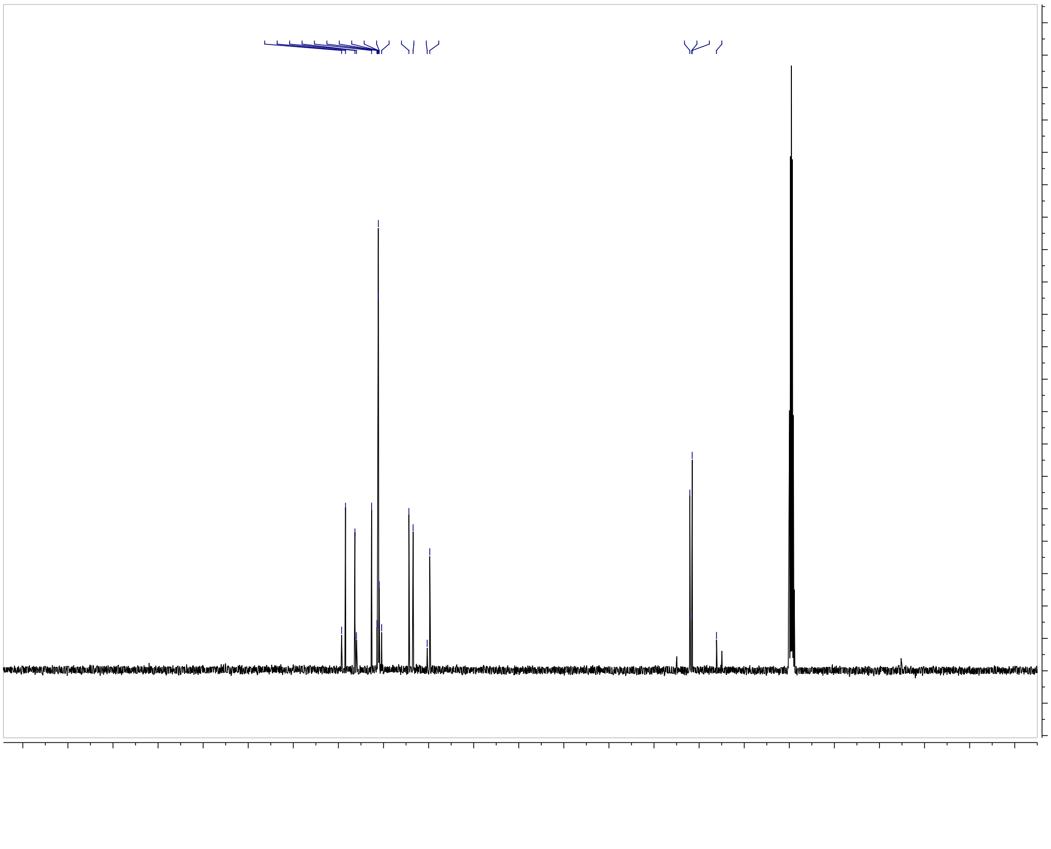

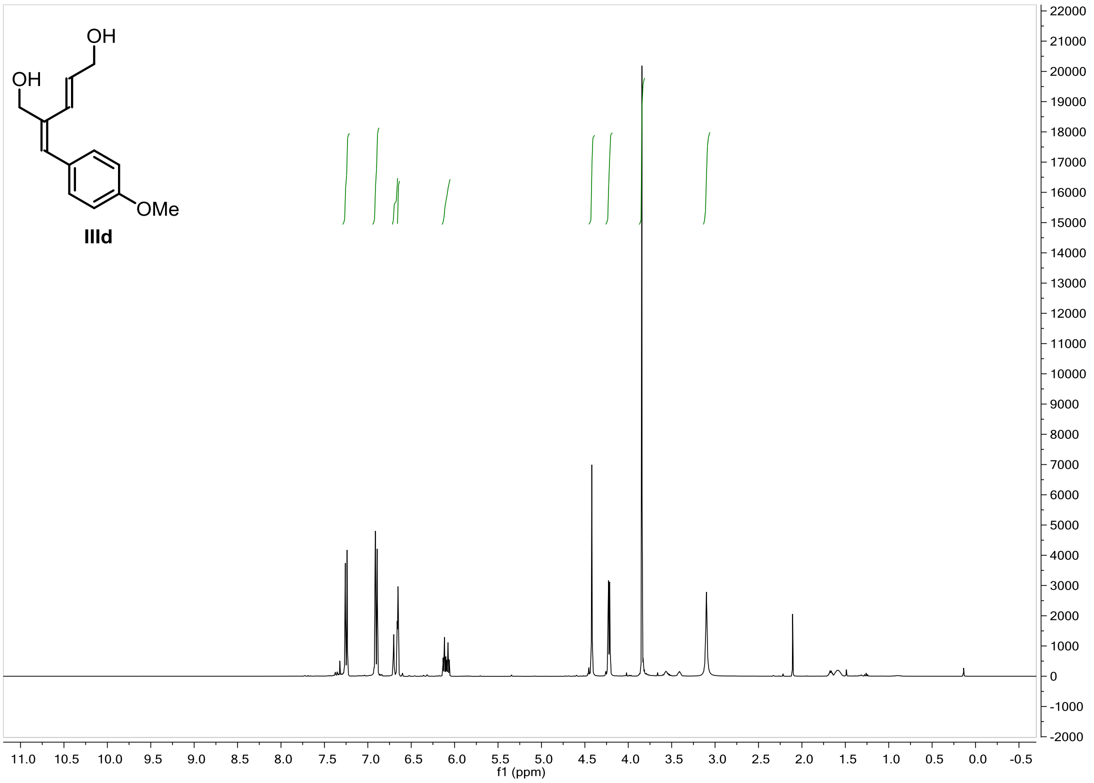
OH
OH
OMe
IIId
f1 (ppm)

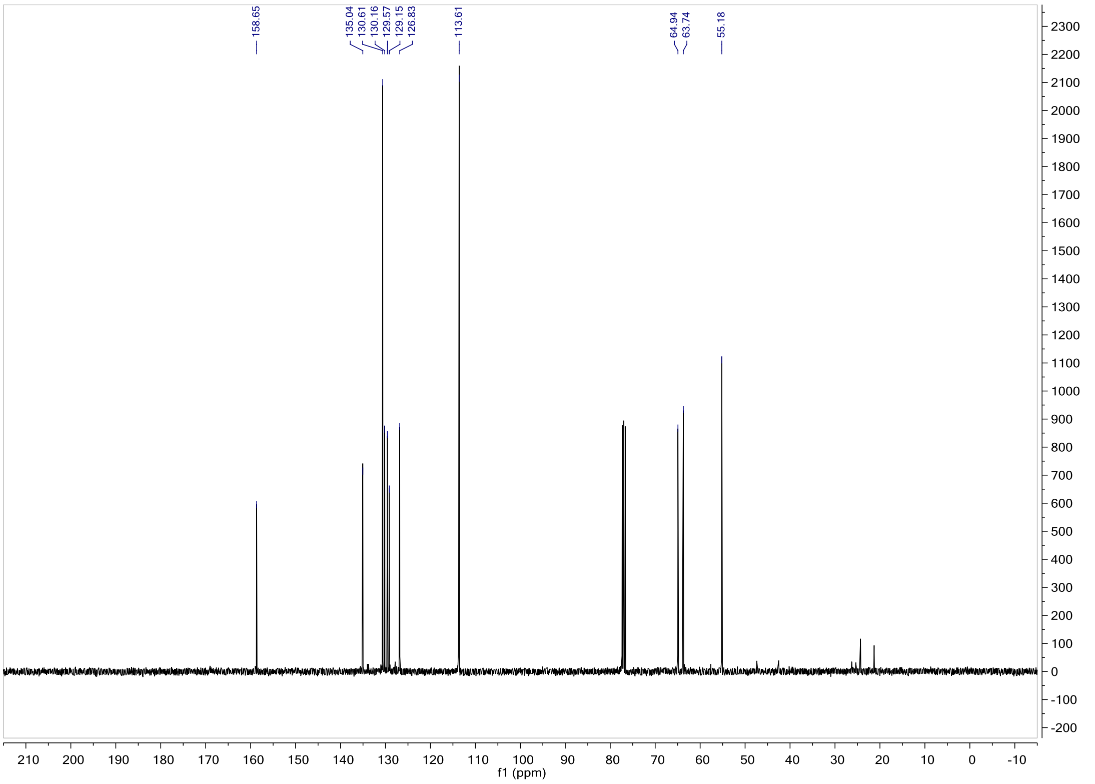
158.65
135.04
130.61
130.16
129.57
129.15
126.83
113.61
64.94
63.74
55.18
f1 (ppm)

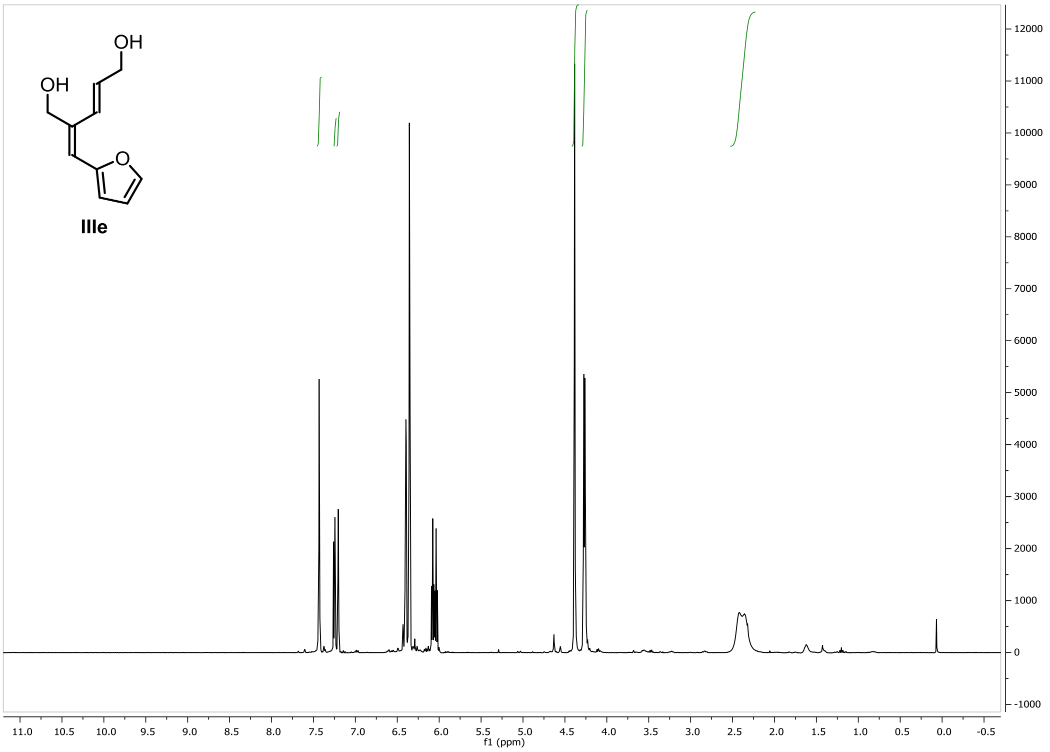
IIIe
f1 (ppm)

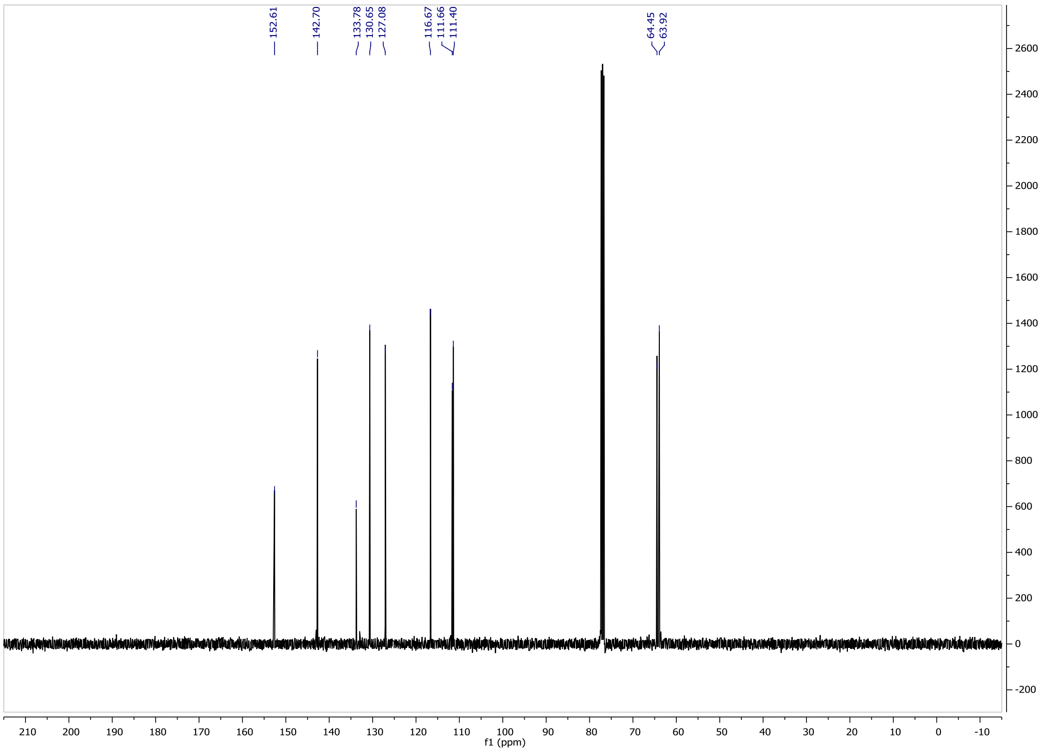
152.61
142.70
133.78
130.65
127.08
116.67
111.66
111.40
64.45
63.92
f1 (ppm)

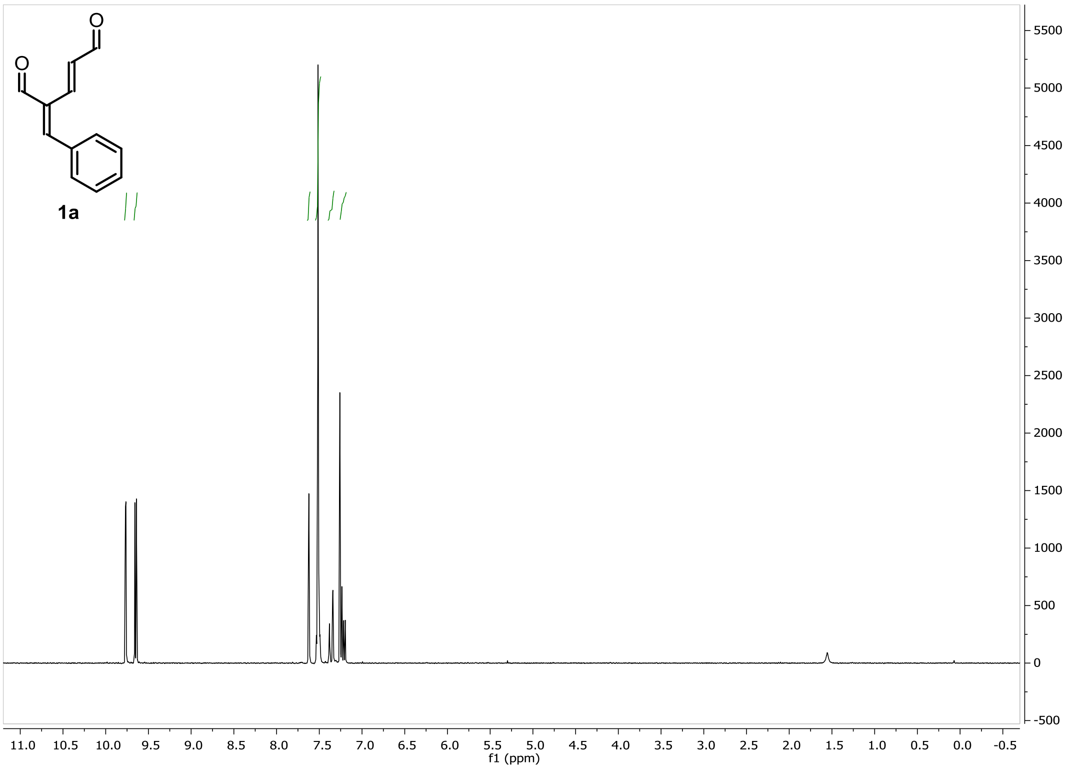
1a
f1 (ppm)

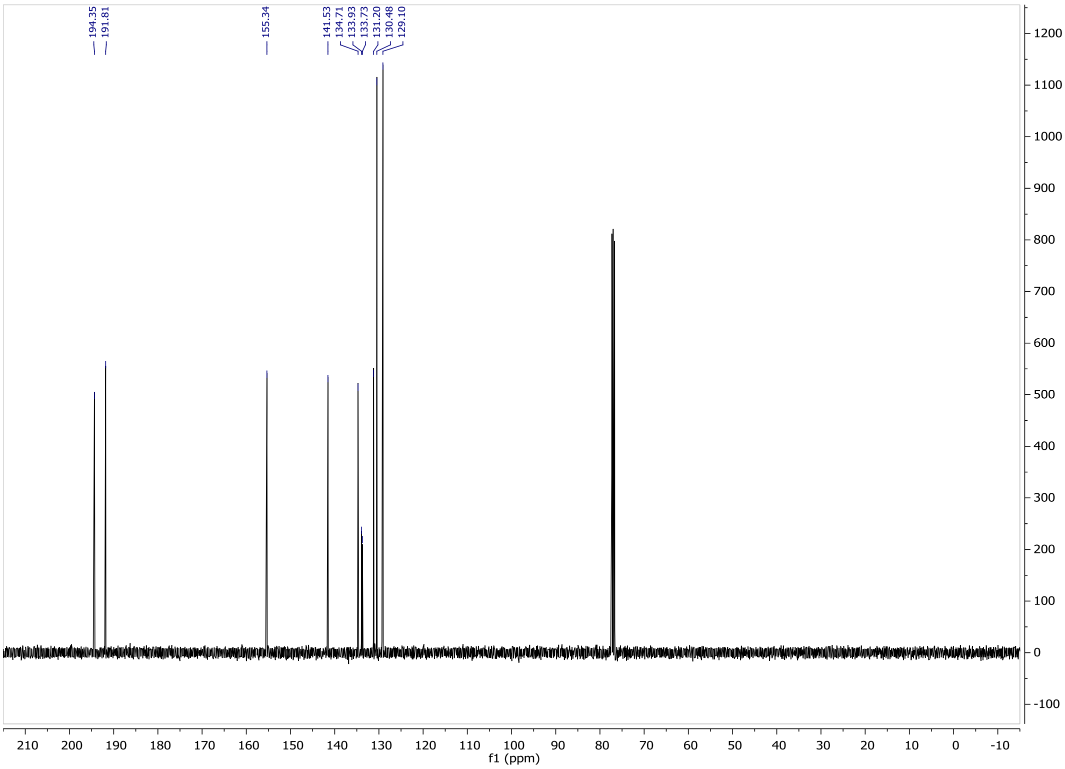
194.35
191.81
155.34
141.53
134.71
133.93
133.73
131.20
130.48
129.10
f1 (ppm)

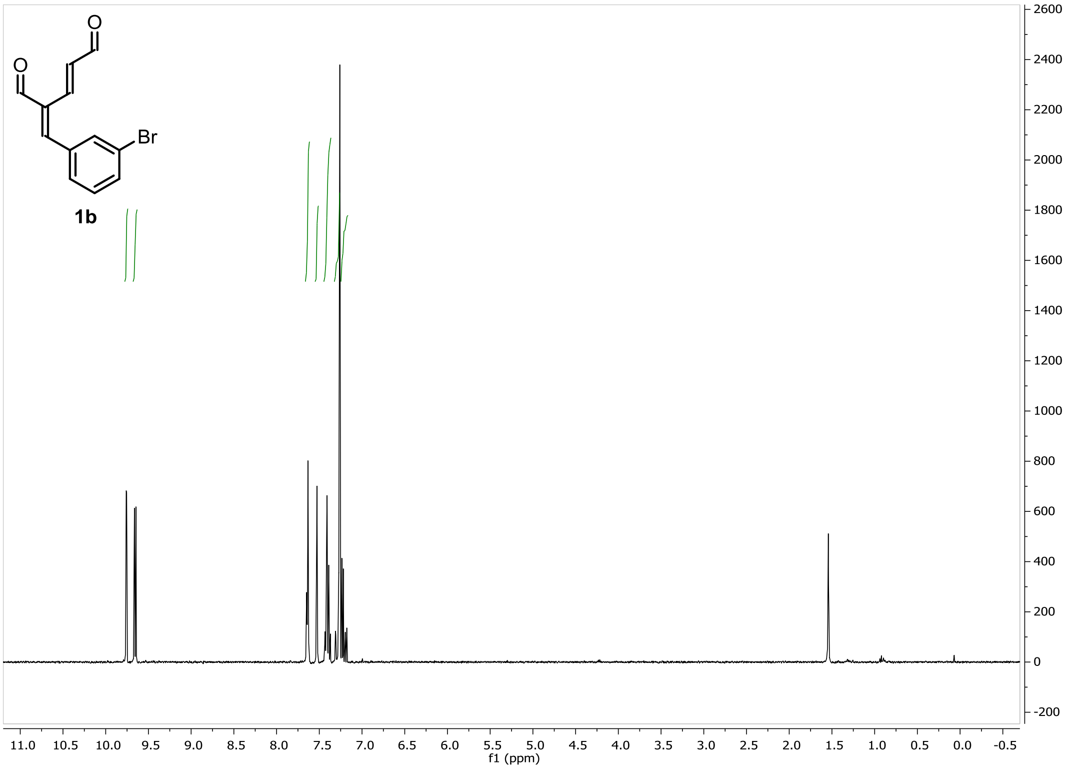
Br
1b
f1 (ppm)

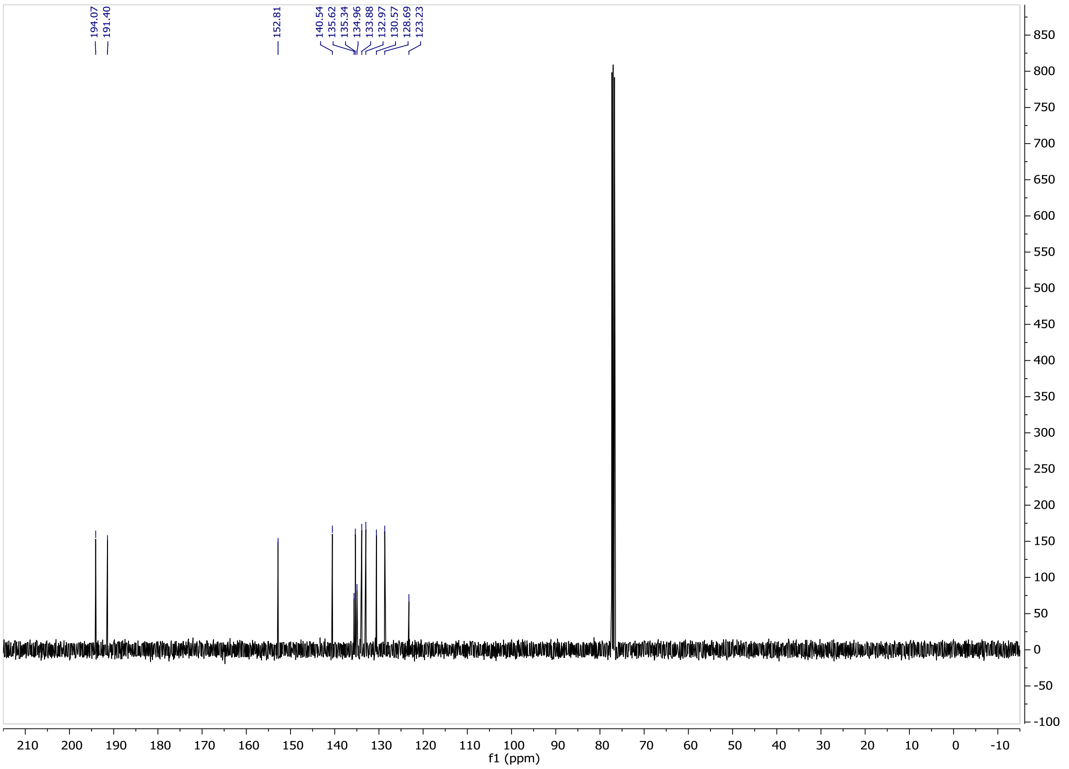
194.07
191.40
152.81
140.54
135.62
135.34
134.96
133.88
132.97
130.57
128.69
123.23
f1 (ppm)

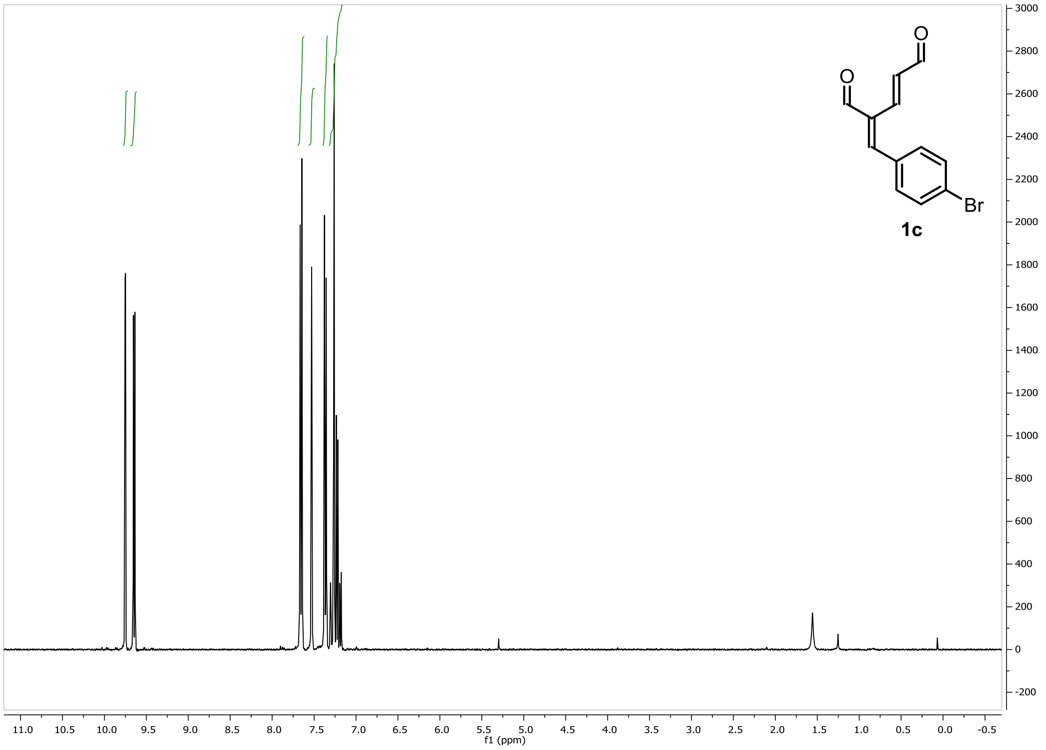
O
O
Br
1c
f1 (ppm)

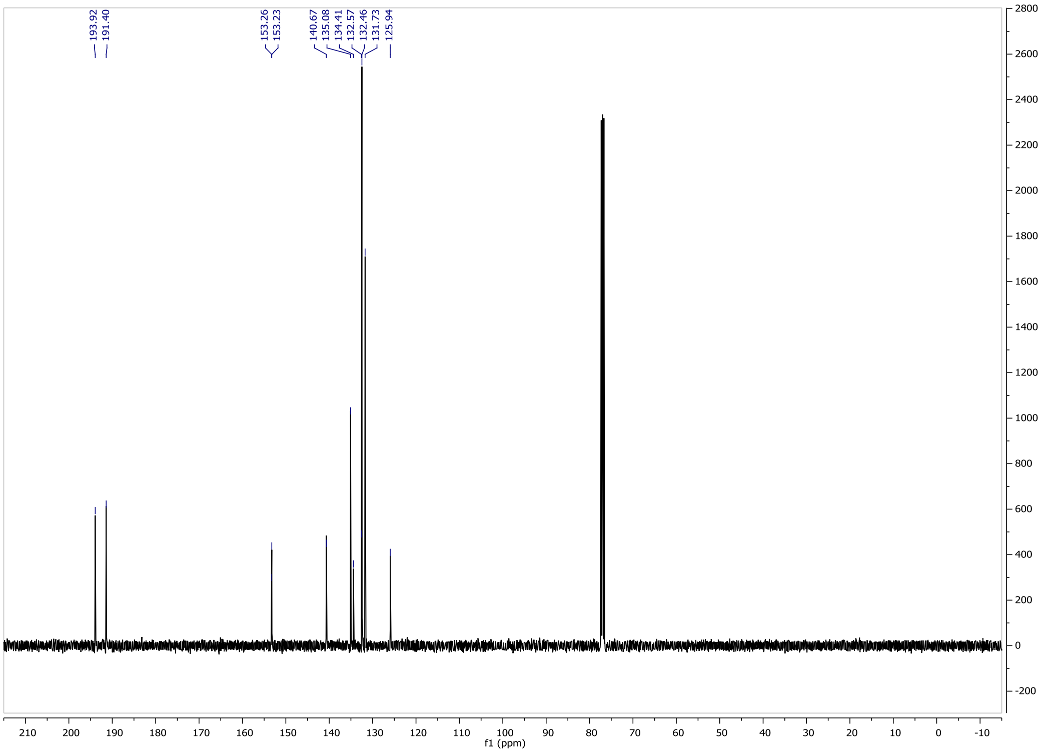
193.92
191.40
153.26
153.23
140.67
135.08
134.41
132.57
132.46
131.73
125.94
f1 (ppm)

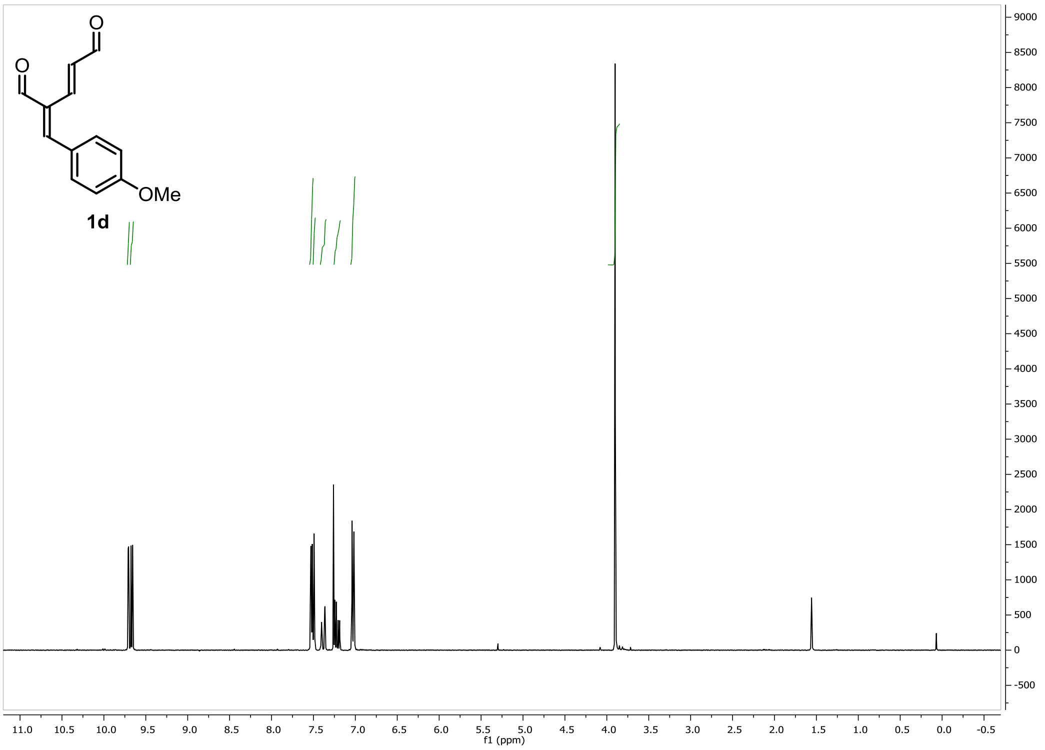
O
O
OMe
1d
f1 (ppm)

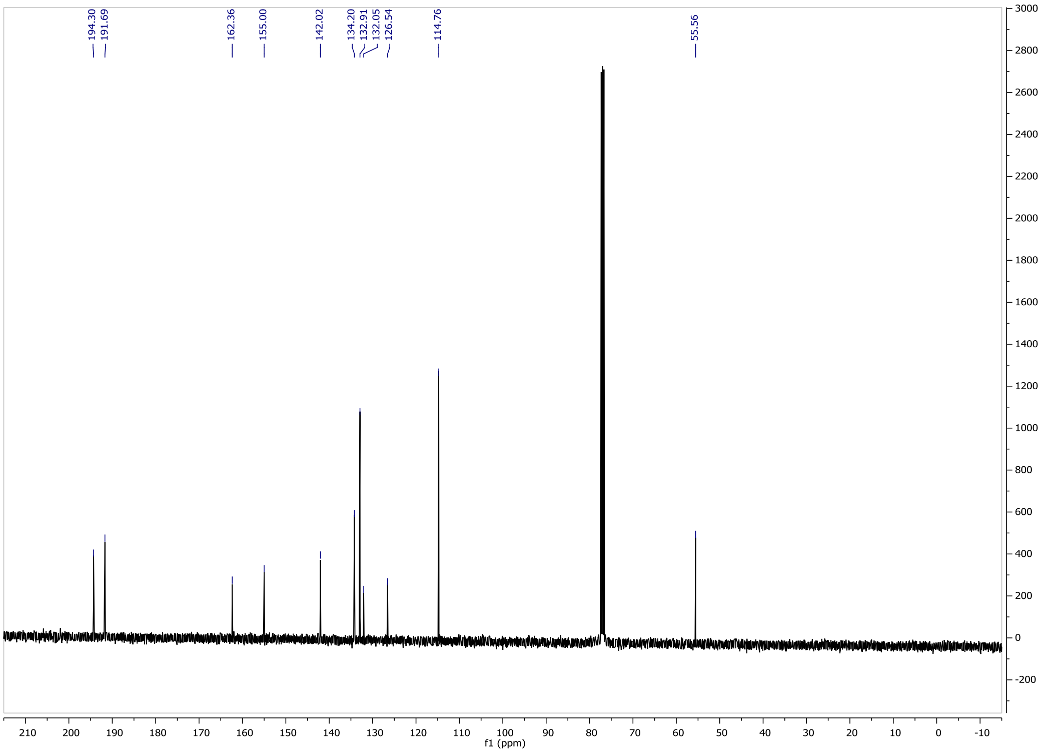
194.30
191.69
162.36
155.00
142.02
134.20
132.91
132.05
126.54
114.76
55.56
f1 (ppm)

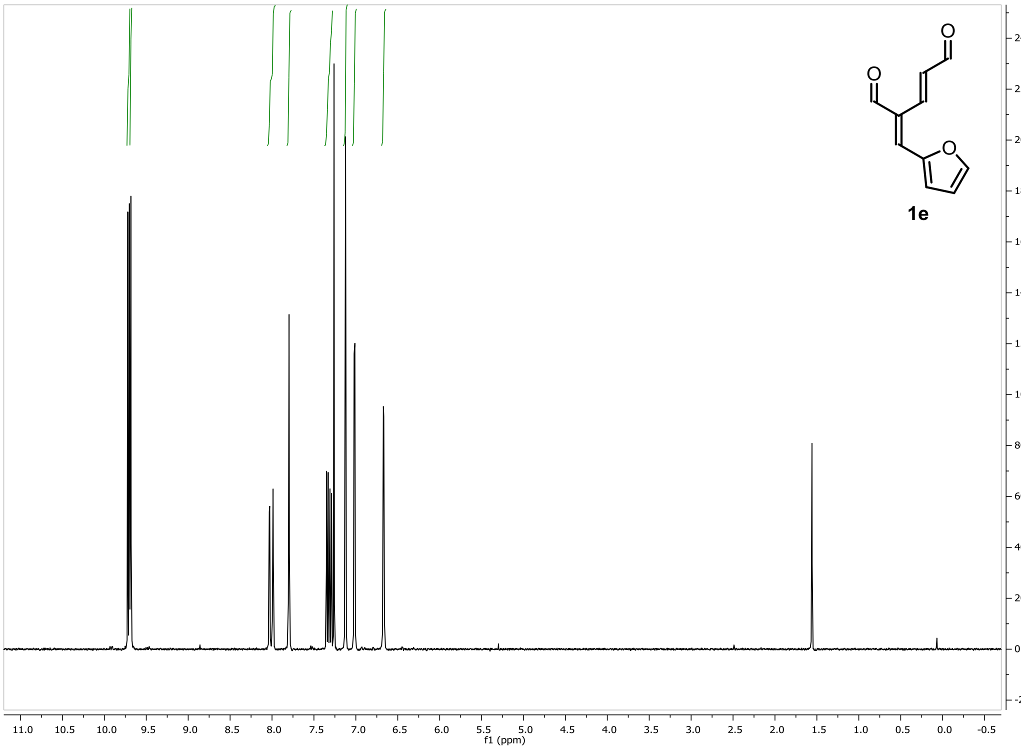
1e
f1 (ppm)

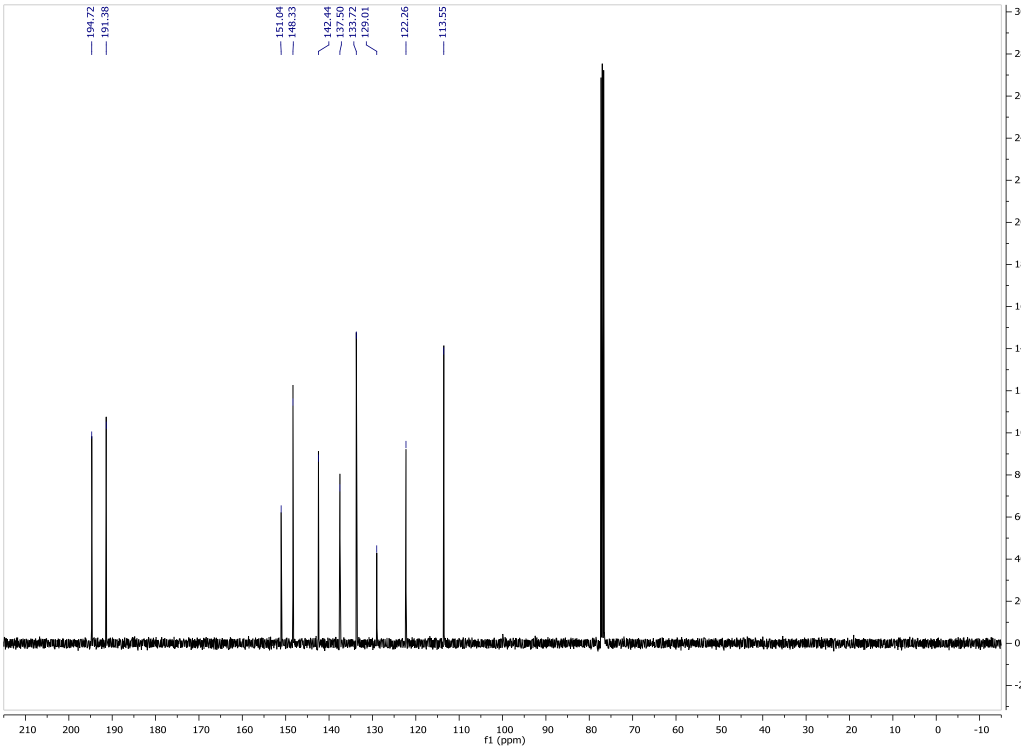
194.72
191.38
151.04
148.33
142.44
137.50
133.72
129.01
122.26
113.55
f1 (ppm)

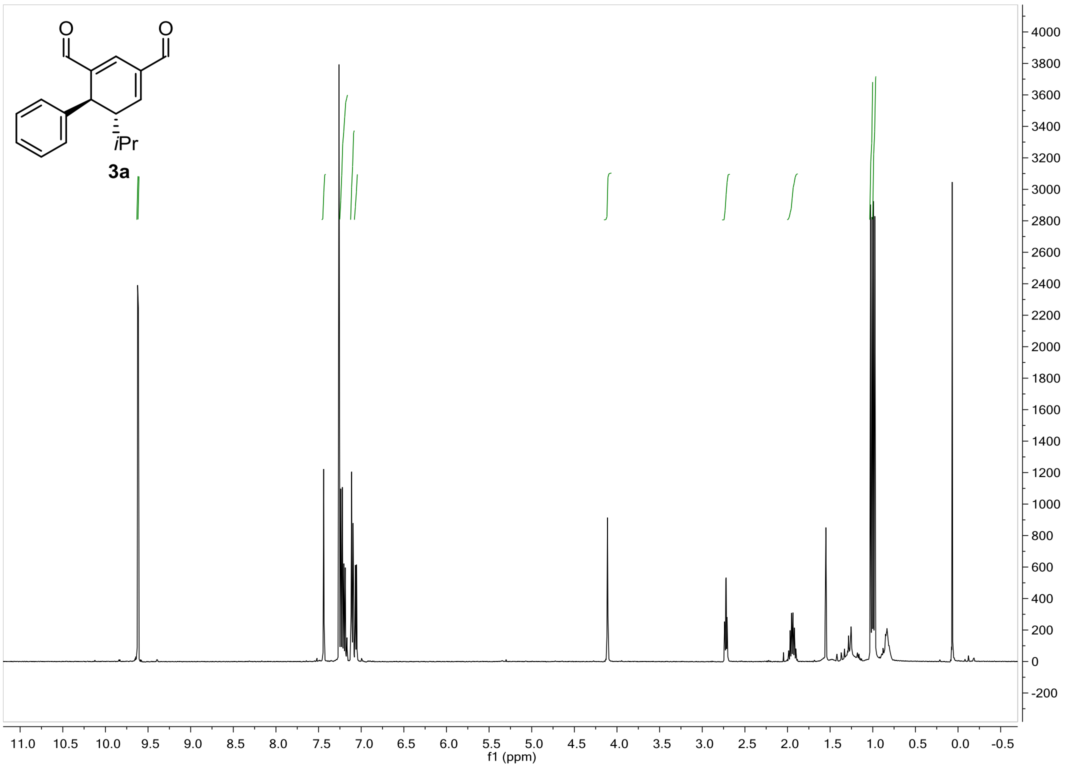

O
O
iPr
3a
f1 (ppm)

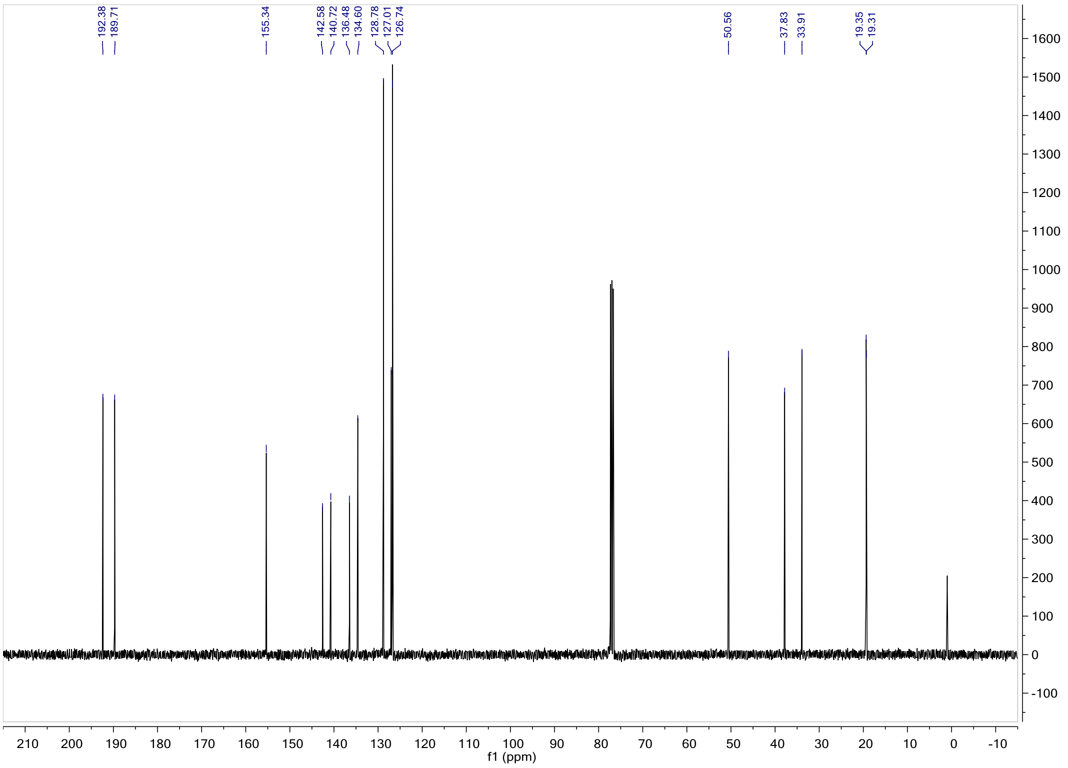

192.38
189.71
155.34
142.58
140.72
136.48
134.60
128.78
127.01
126.74
50.56
37.83
33.91
19.35
19.31
f1 (ppm)

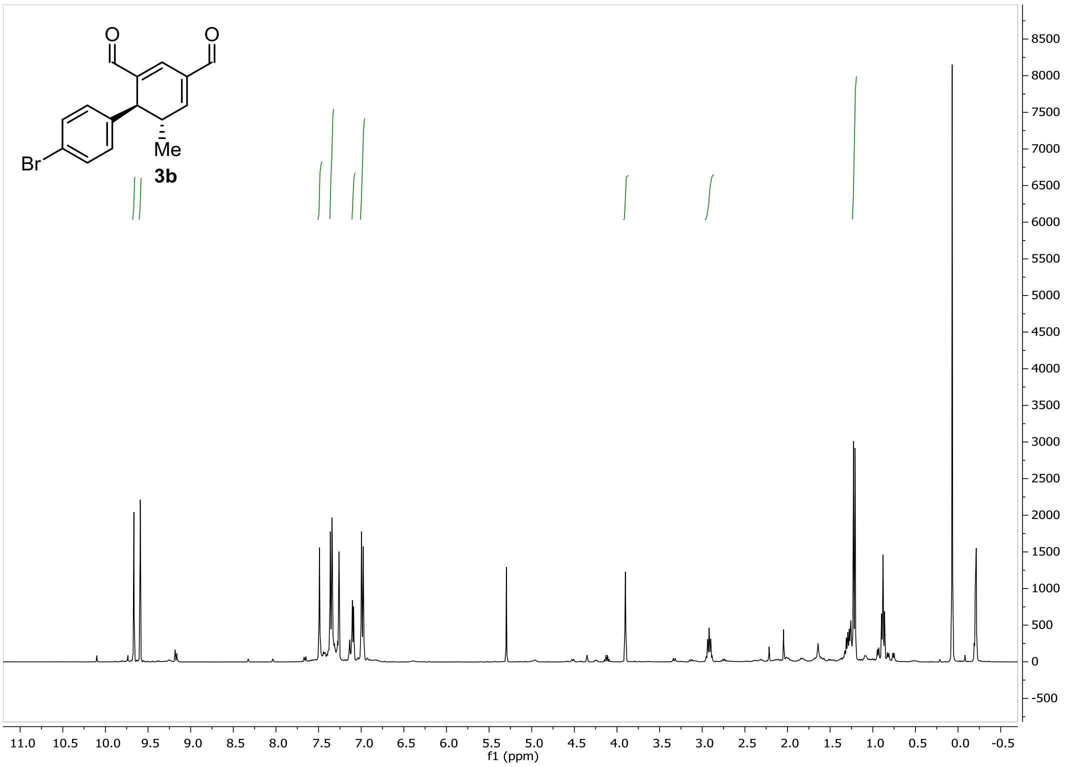
O
O
Me
Br
3b
f1 (ppm)

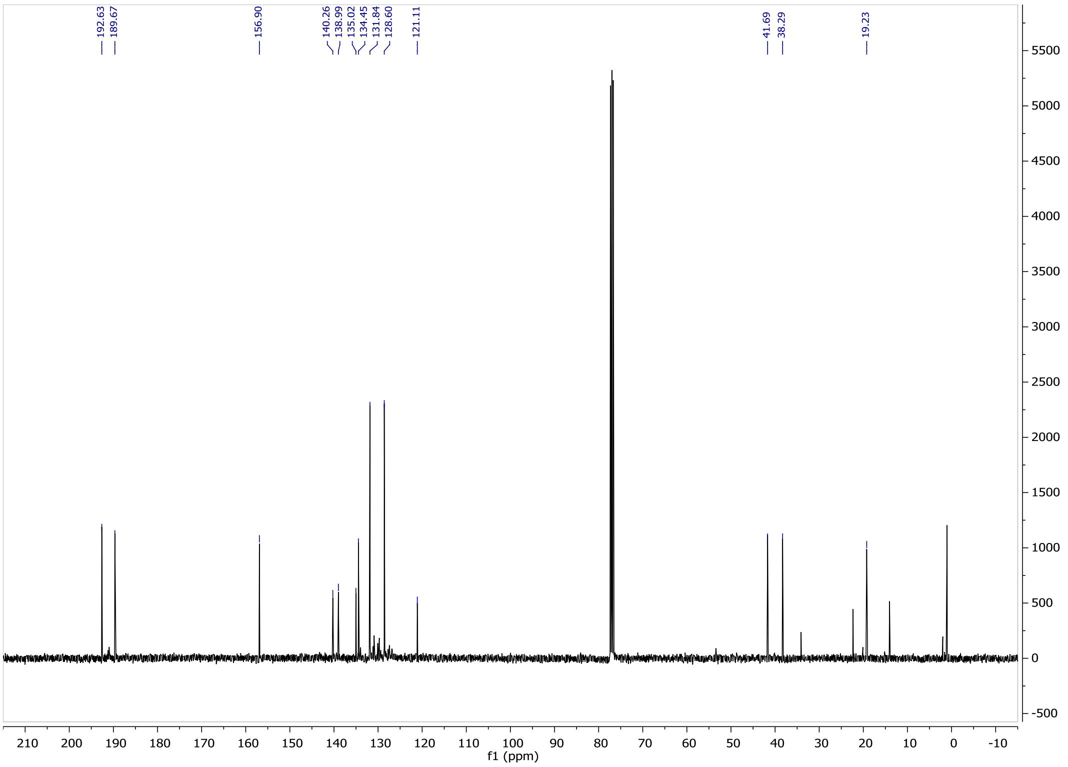
192.63
189.67
156.90
140.26
138.99
135.02
134.45
131.84
128.60
121.11
41.69
38.29
19.23
f1 (ppm)

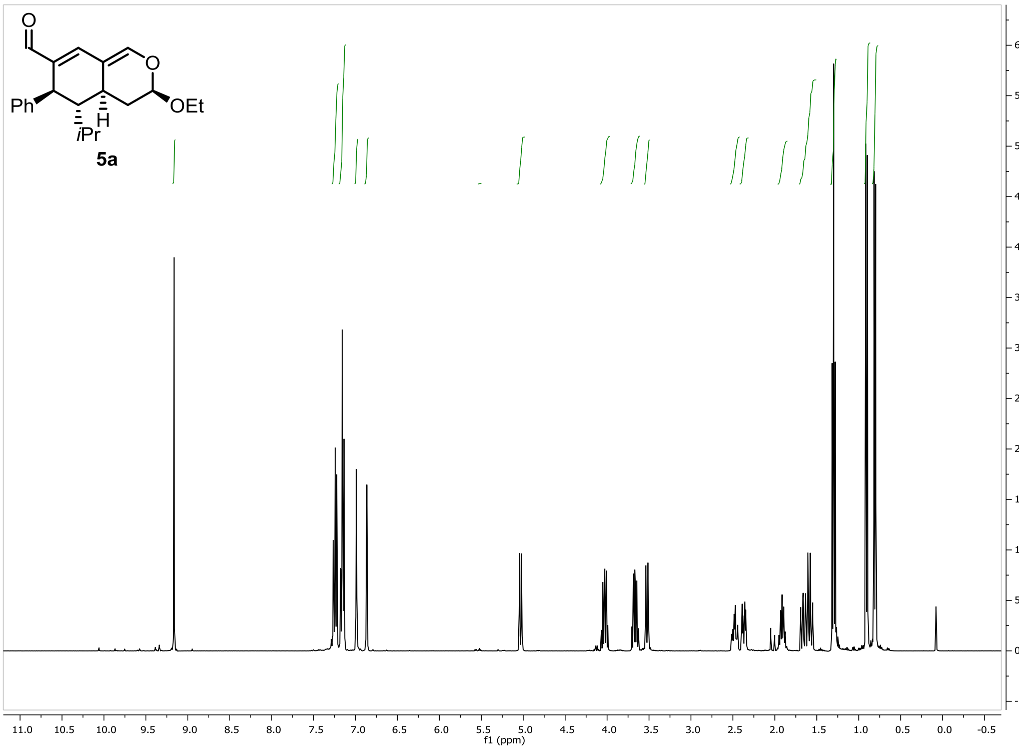
O
Ph
OEt
H
iPr
5a
f1 (ppm)

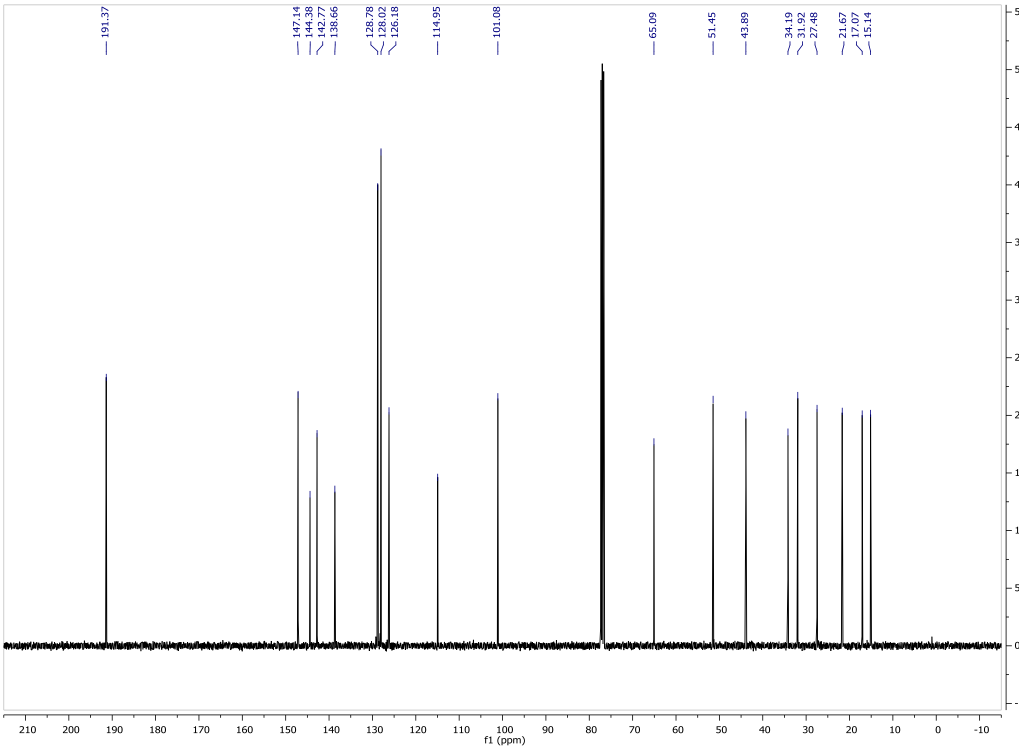
191.37
147.14
144.38
142.77
138.66
128.78
128.02
126.18
114.95
101.08
65.09
51.45
43.89
34.19
31.92
27.48
21.67
17.07
15.14
f1 (ppm)

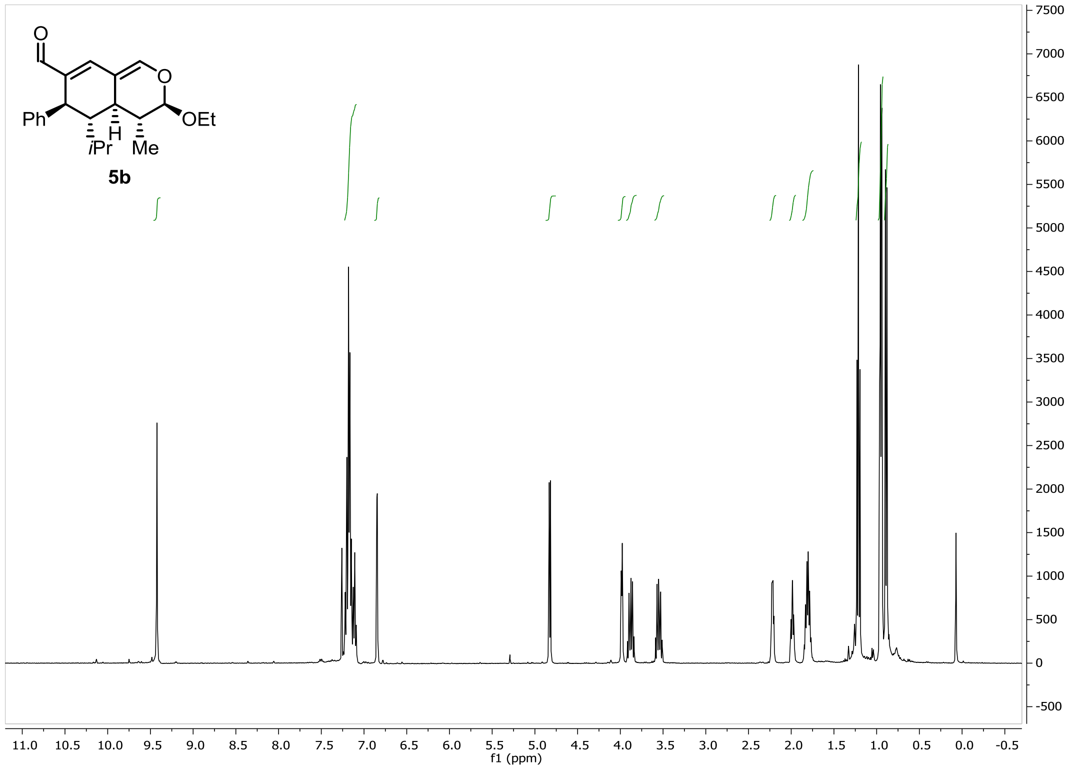
Ph
OEt
iPr
H
Me
O
5b
f1 (ppm)

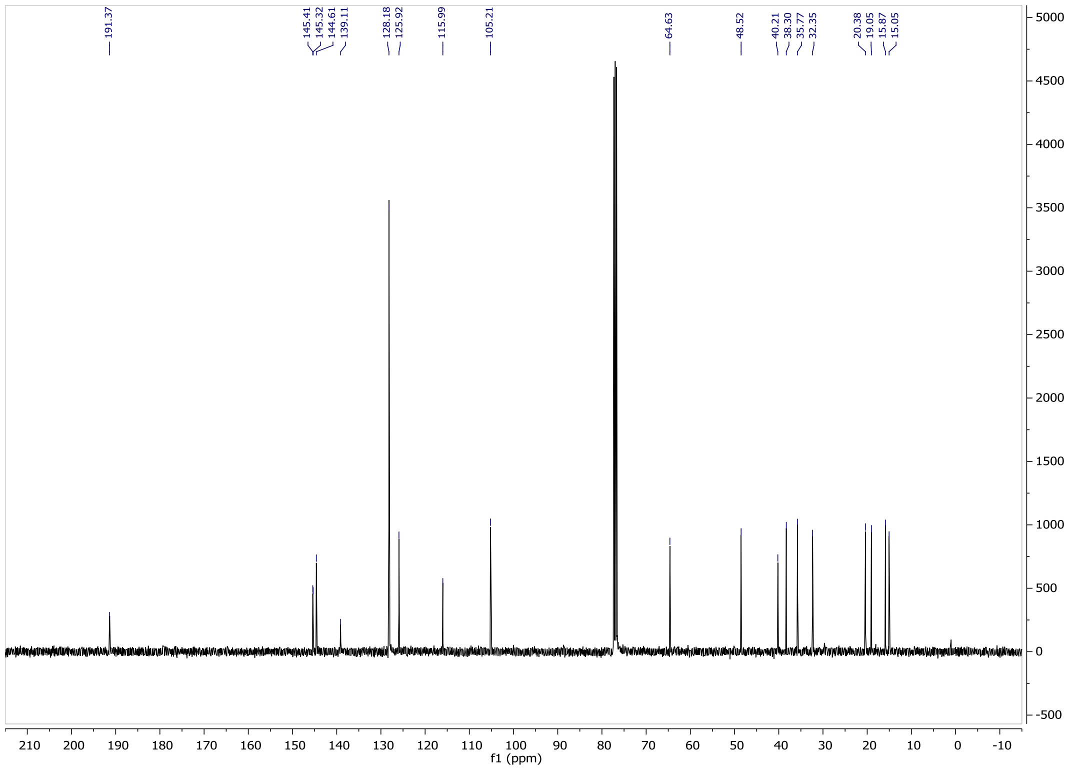
191.37
145.41
145.32
144.61
139.11
128.18
125.92
115.99
105.21
64.63
48.52
40.21
38.30
35.77
32.35
20.38
19.05
15.87
15.05
f1 (ppm)

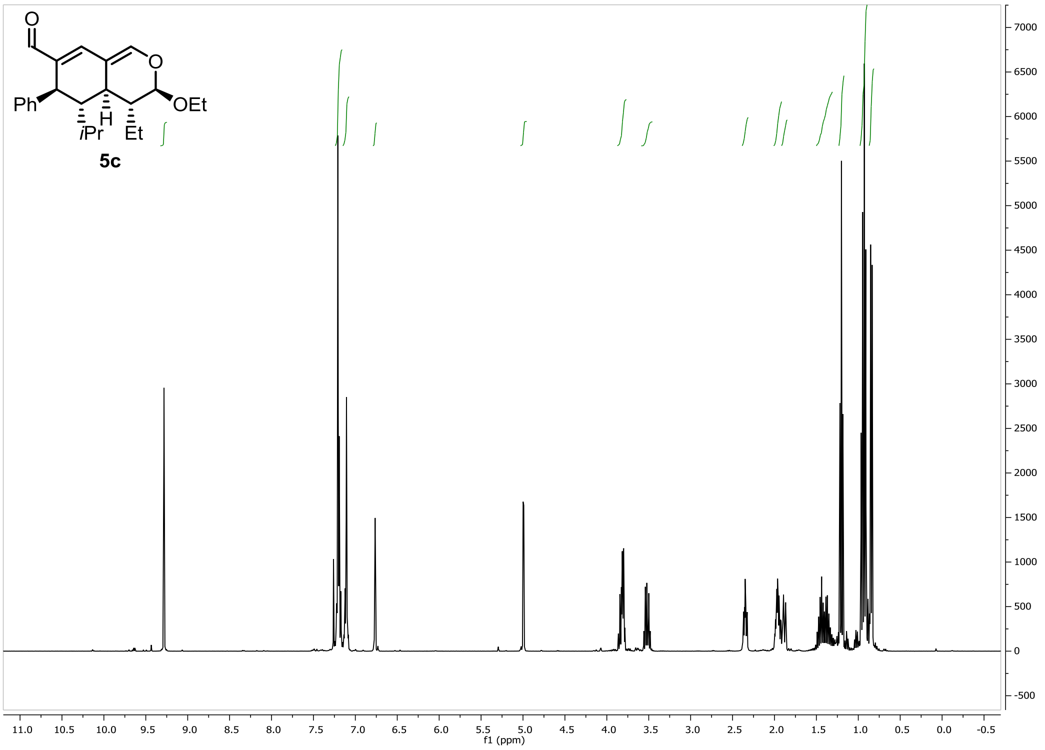
Ph
OEt
H
iPr
Et
5c
f1 (ppm)

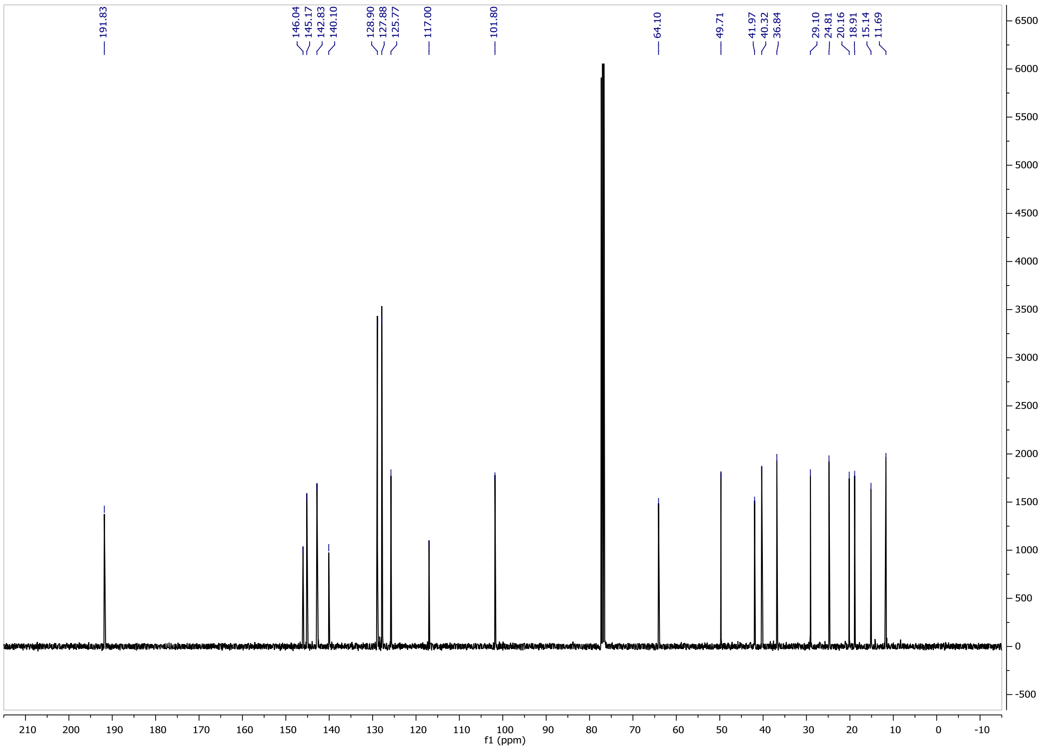
191.83
146.04
145.17
142.83
140.10
128.90
127.88
125.77
117.00
101.80
64.10
49.71
41.97
40.32
36.84
29.10
24.81
20.16
18.91
15.14
11.69
f1 (ppm)

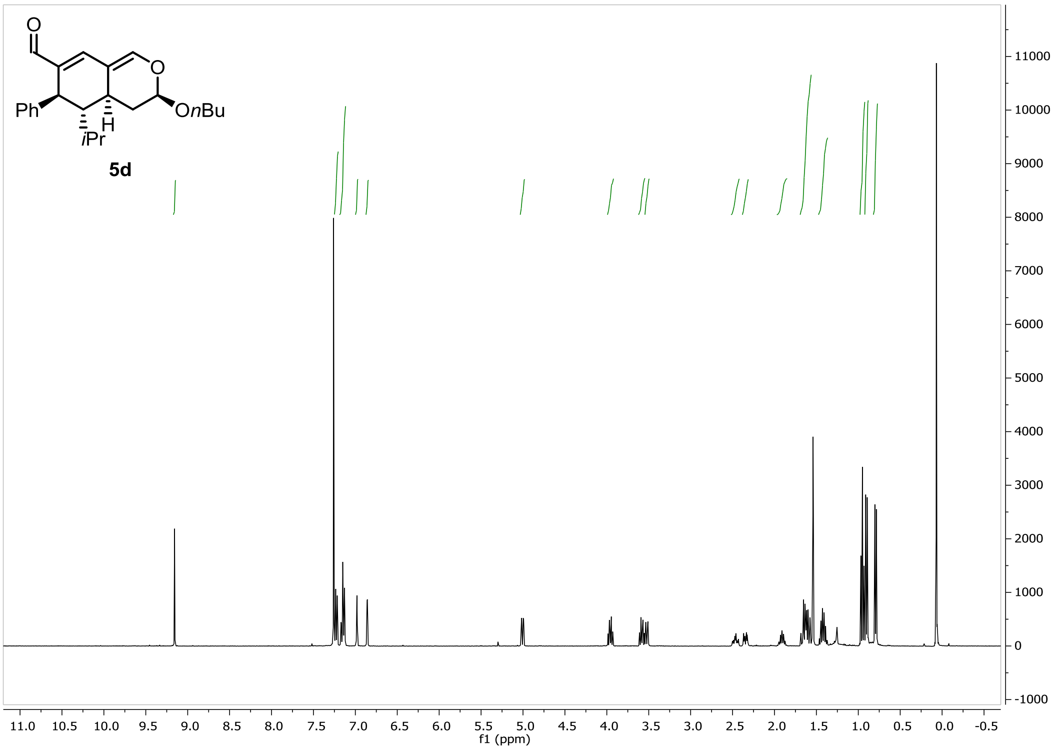
O
Ph
OnBu
H
iPr
5d
f1 (ppm)

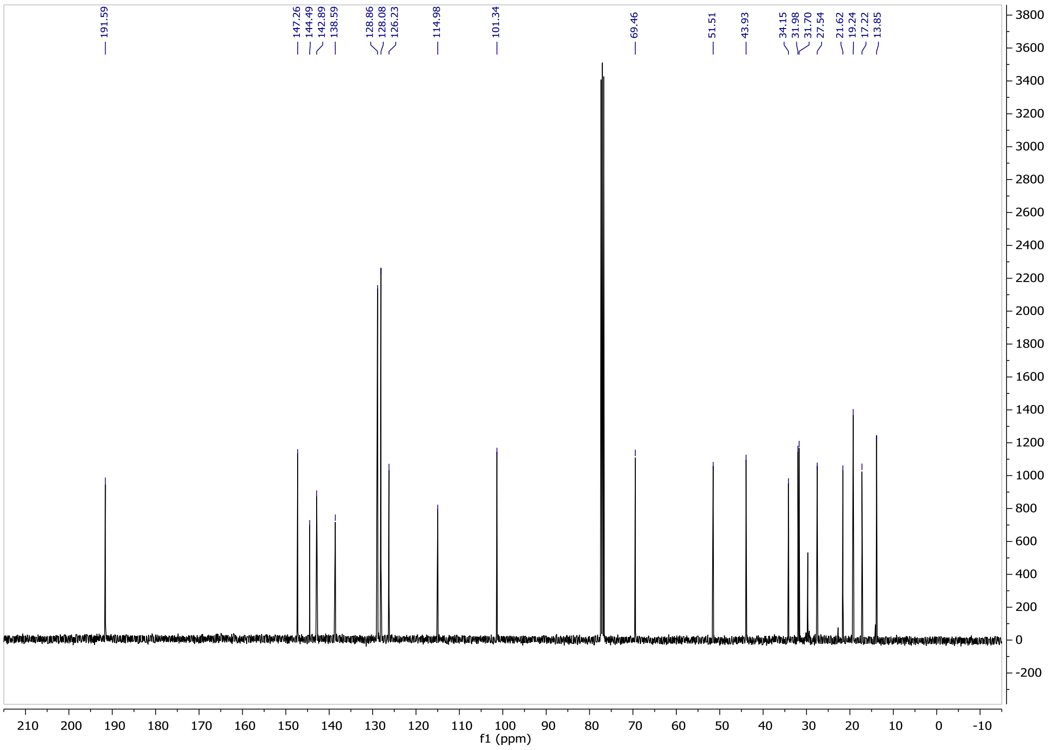
191.59
147.26
144.49
142.89
138.59
128.86
128.08
126.23
114.98
101.34
69.46
51.51
43.93
34.15
31.98
31.70
27.54
21.62
19.24
17.22
13.85
f1 (ppm)

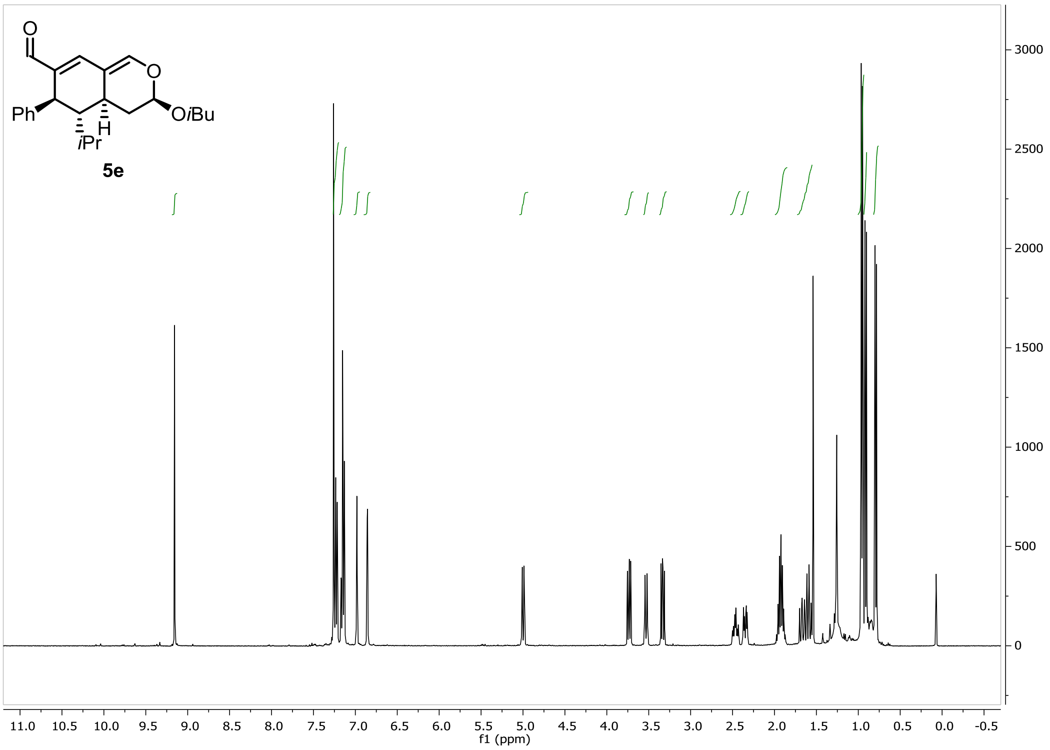
O
Ph
O
H
OiBu
iPr
5e
f1 (ppm)

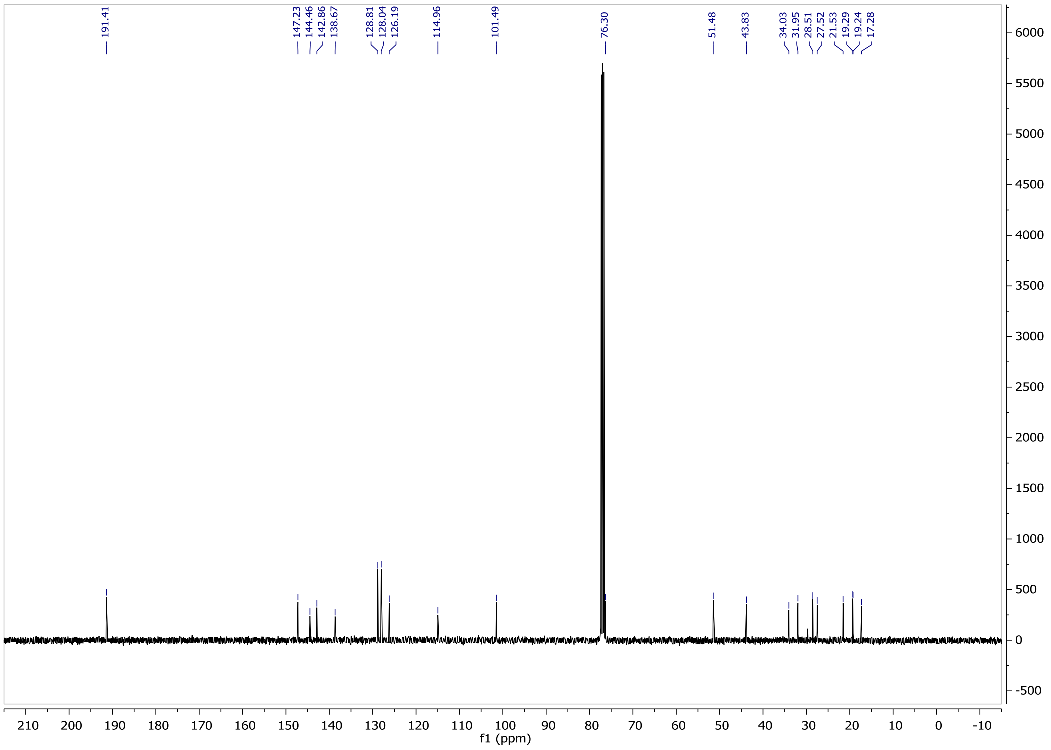
191.41
147.23
144.46
142.86
138.67
128.81
128.04
126.19
114.96
101.49
76.30
51.48
43.83
34.03
31.95
28.51
27.52
21.53
19.29
19.24
17.28
f1 (ppm)

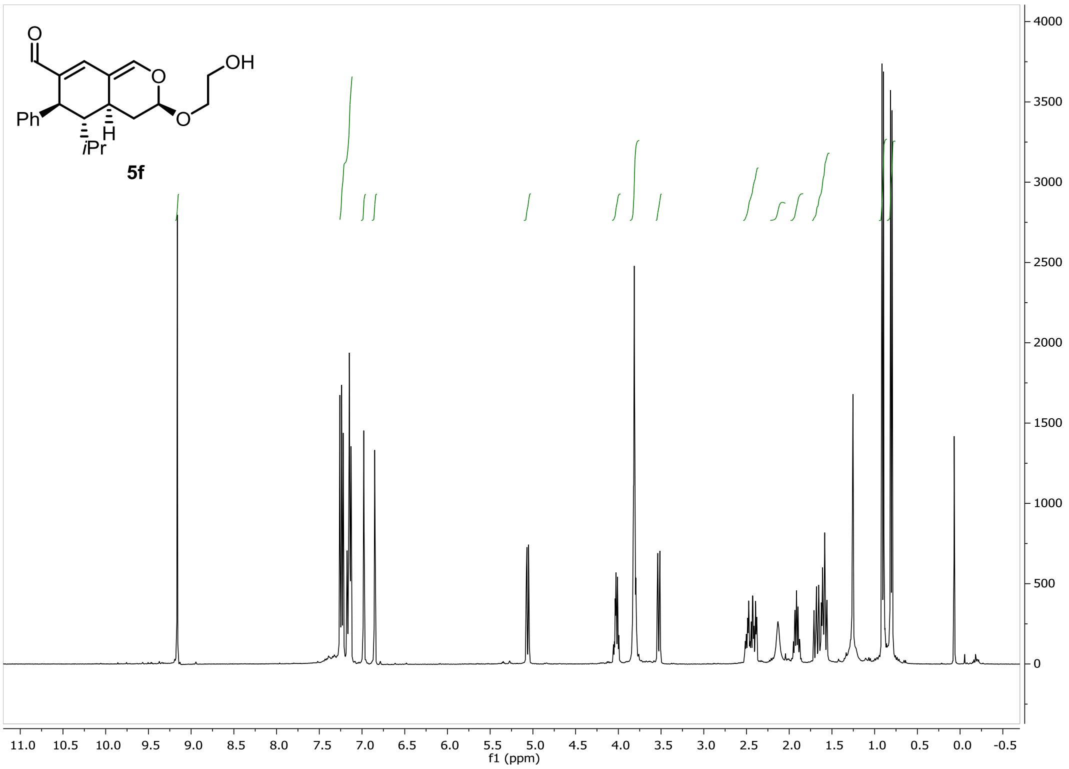
OH
Ph
iPr
H
O
O
O
5f
f1 (ppm)

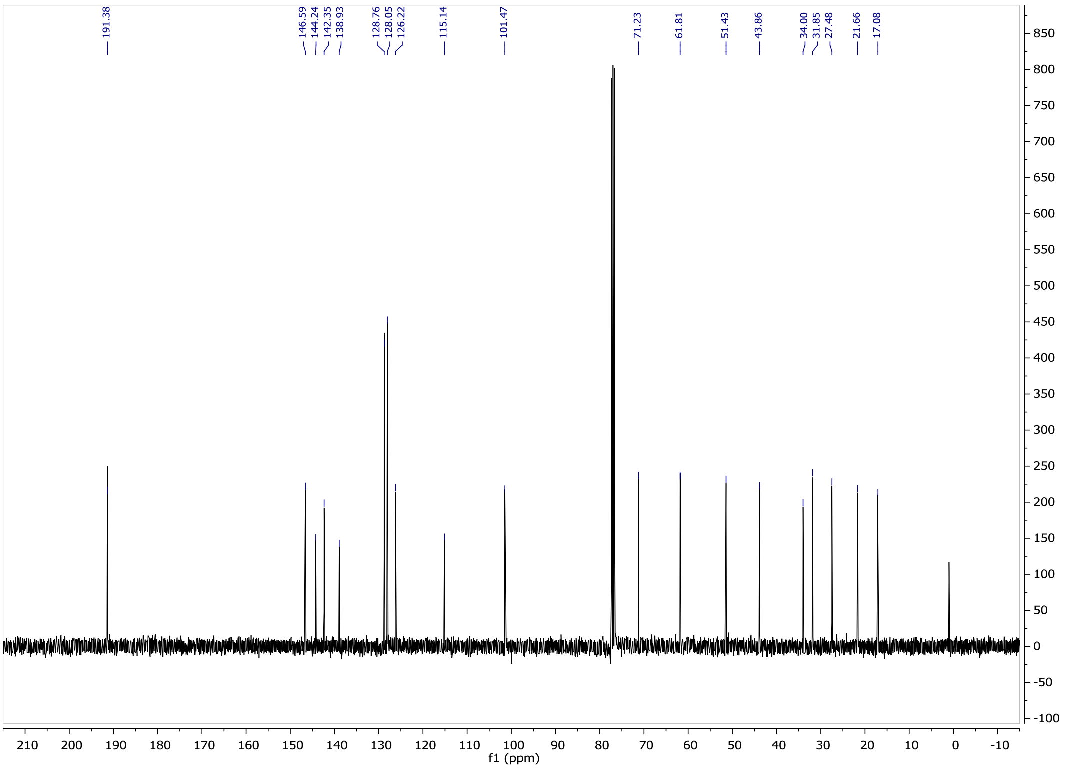
191.38
146.59
144.24
142.35
138.93
128.76
128.05
126.22
115.14
101.47
71.23
61.81
51.43
43.86
34.00
31.85
27.48
21.66
17.08
f1 (ppm)

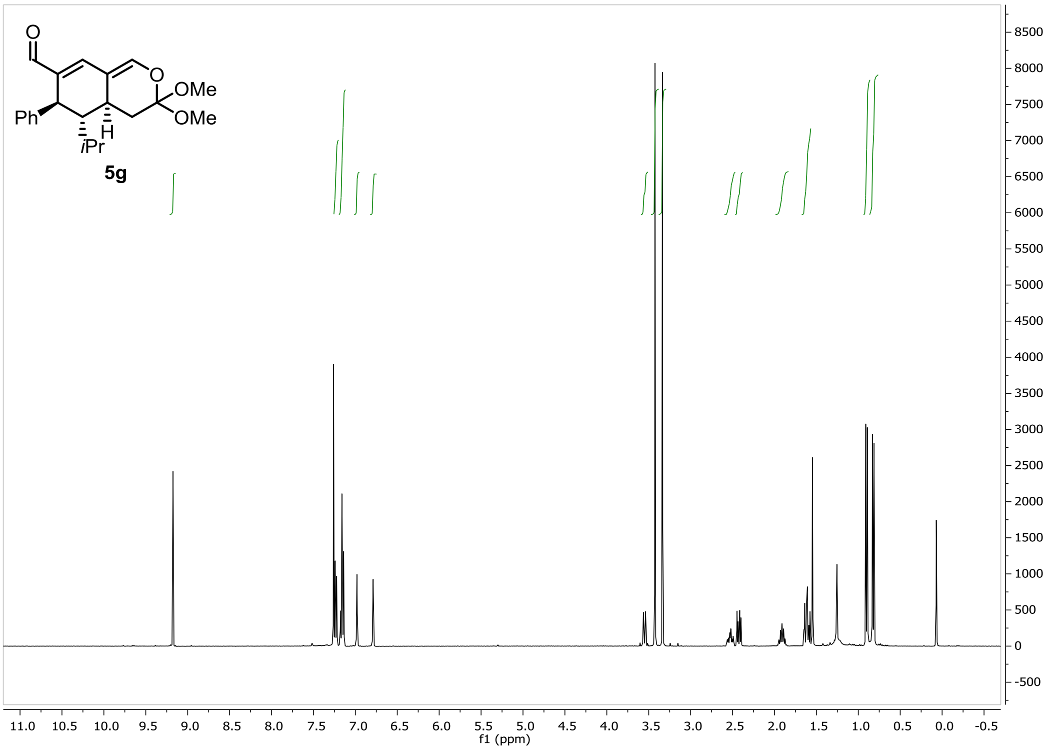

O
OMe
OMe
Ph
H
iPr
5g
f1 (ppm)

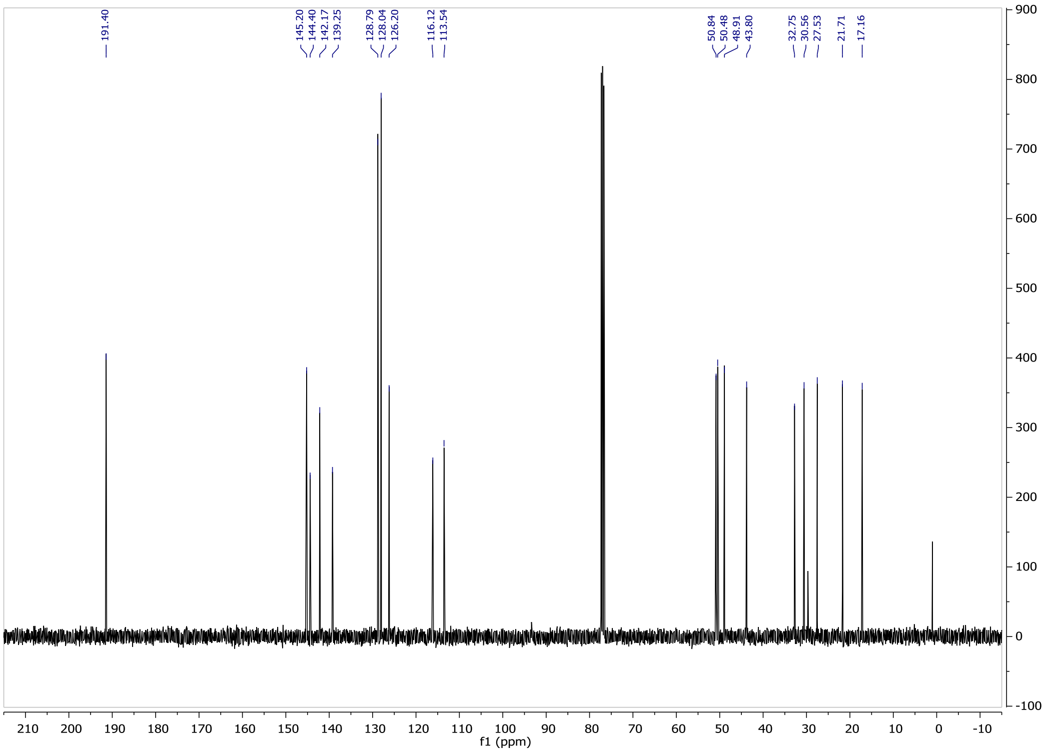

191.40
145.20
144.40
142.17
139.25
128.79
128.04
126.20
116.12
113.54
50.84
50.48
48.91
43.80
32.75
30.56
27.53
21.71
17.16
f1 (ppm)

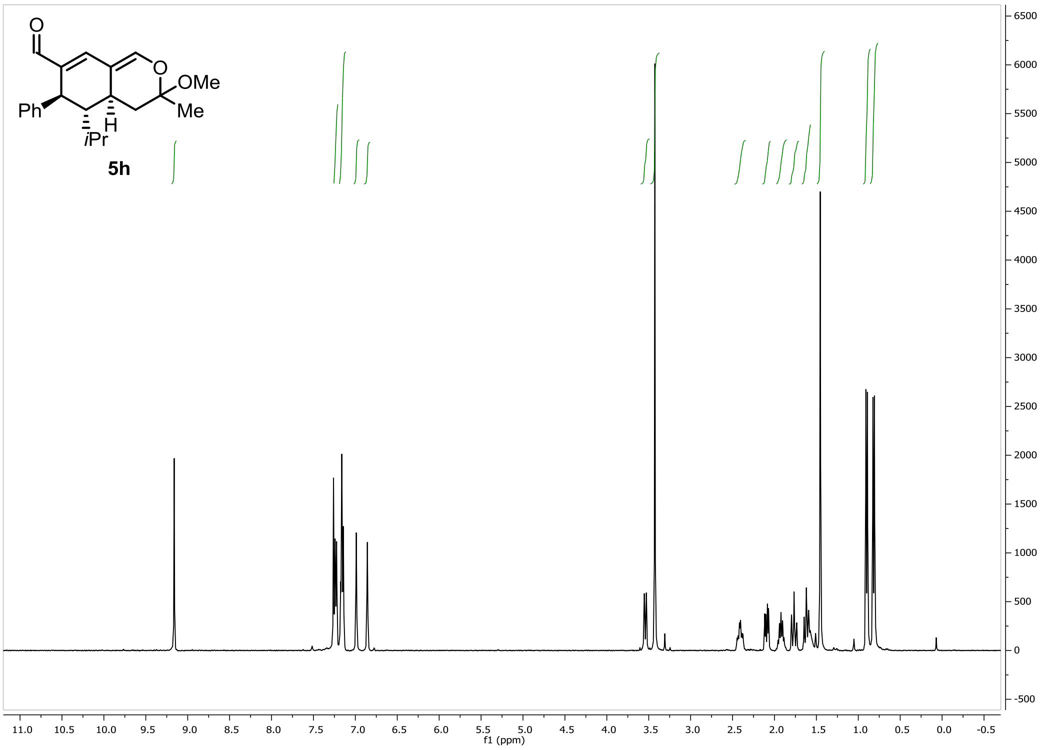
O
OMe
Ph
H
Me
iPr
5h
f1 (ppm)

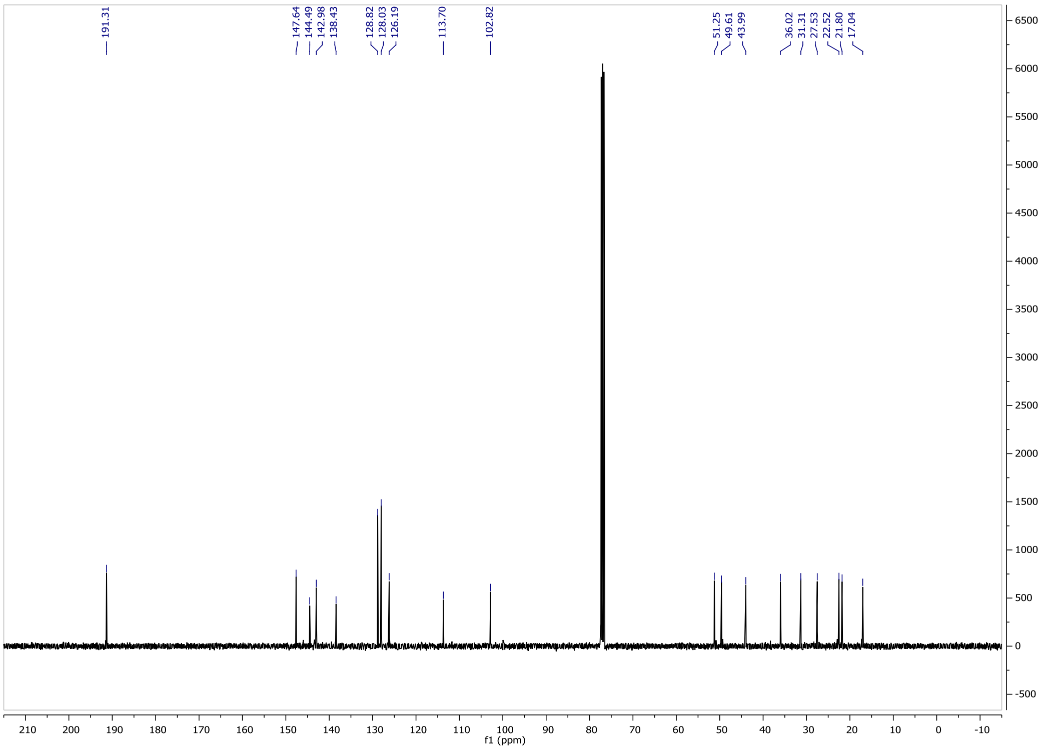
191.31
147.64
144.49
142.98
138.43
128.82
128.03
126.19
113.70
102.82
51.25
49.61
43.99
36.02
31.31
27.53
22.52
21.80
17.04
f1 (ppm)

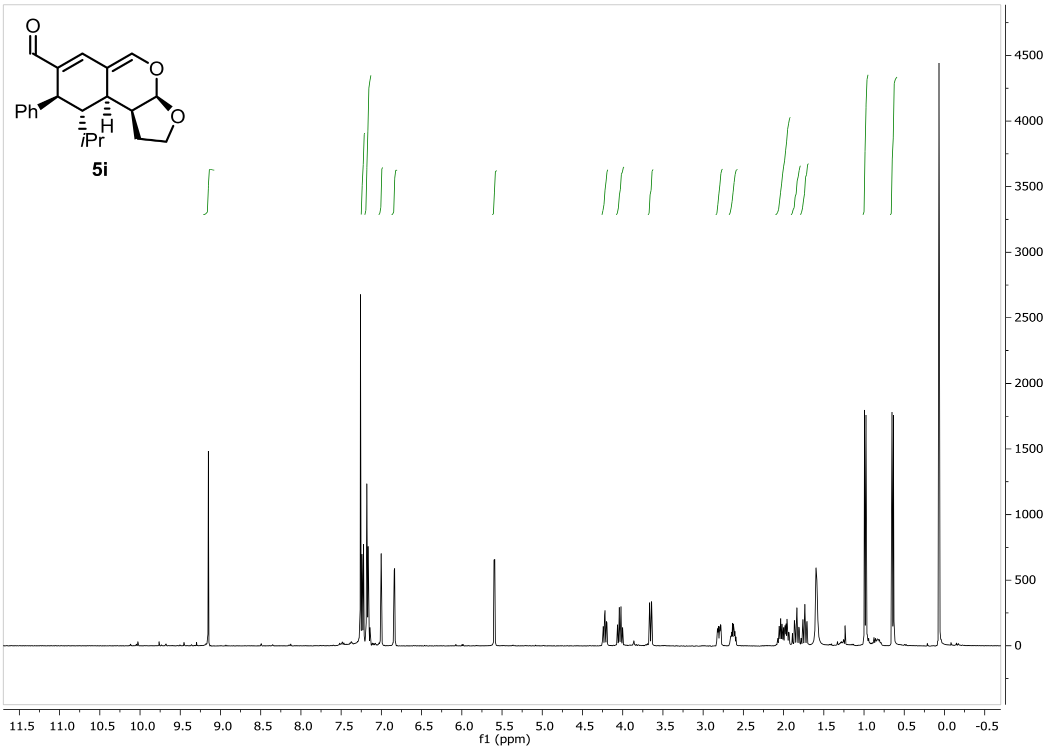
O
O
O
Ph
H
iPr
5i
f1 (ppm)

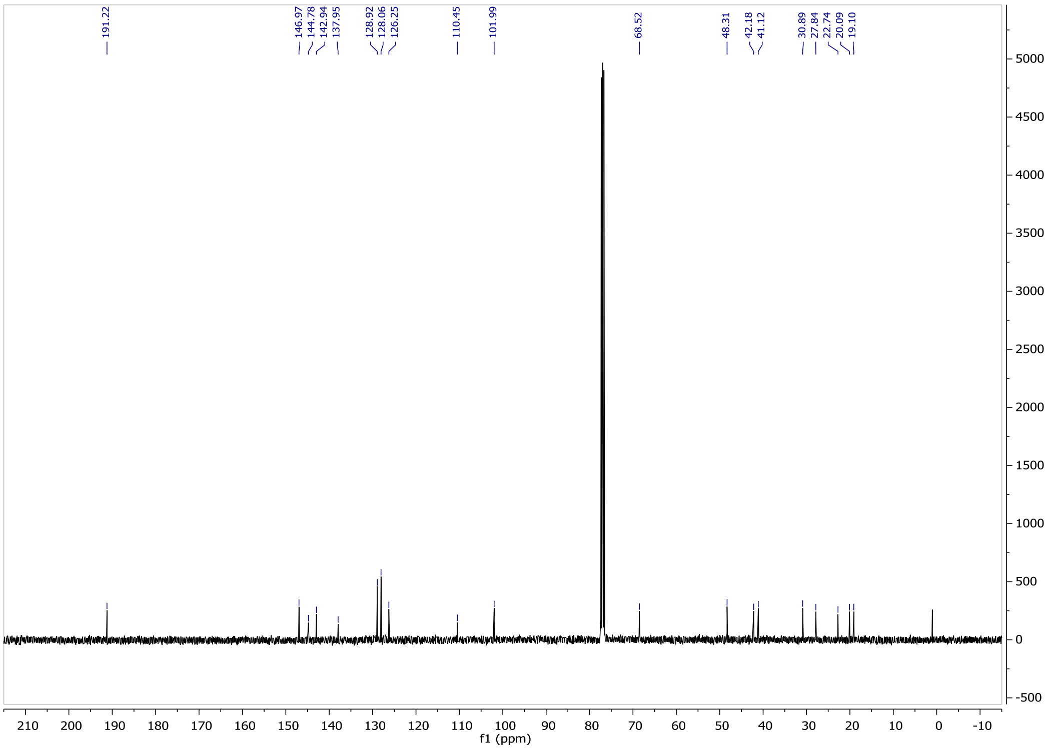
191.22
146.97
144.78
142.94
137.95
128.92
128.06
126.25
110.45
101.99
68.52
48.31
42.18
41.12
30.89
27.84
22.74
20.09
19.10
f1 (ppm)

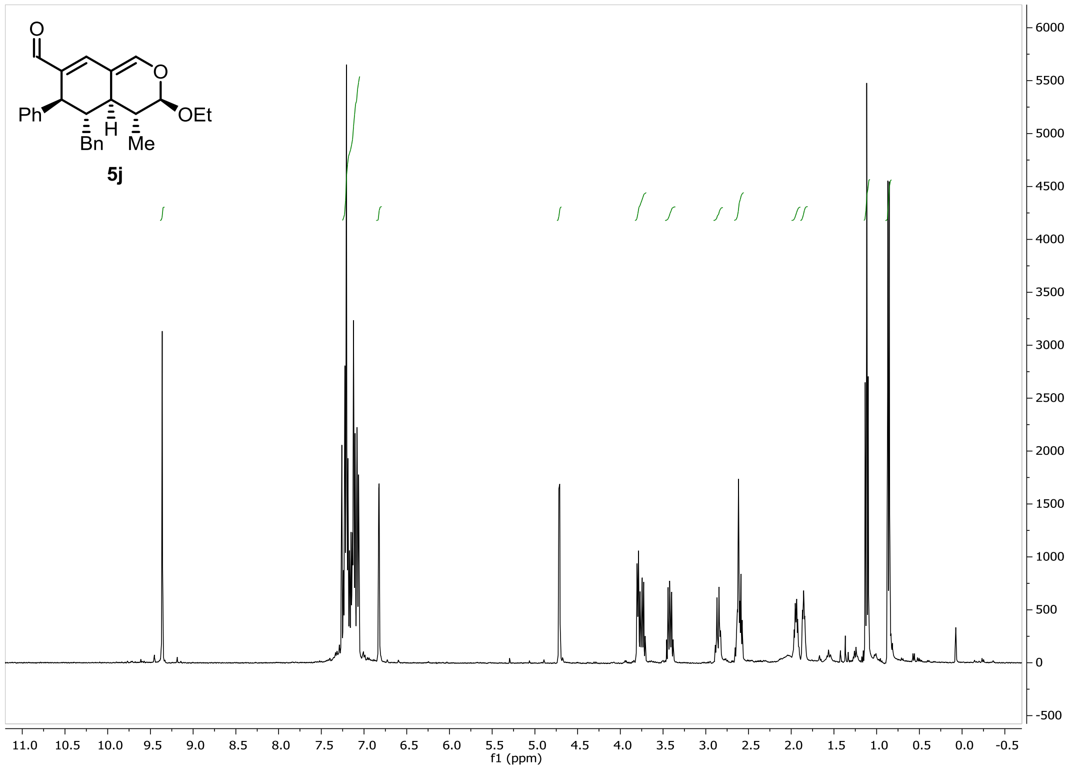
O
Ph
OEt
H
Bn
Me
5j
f1 (ppm)

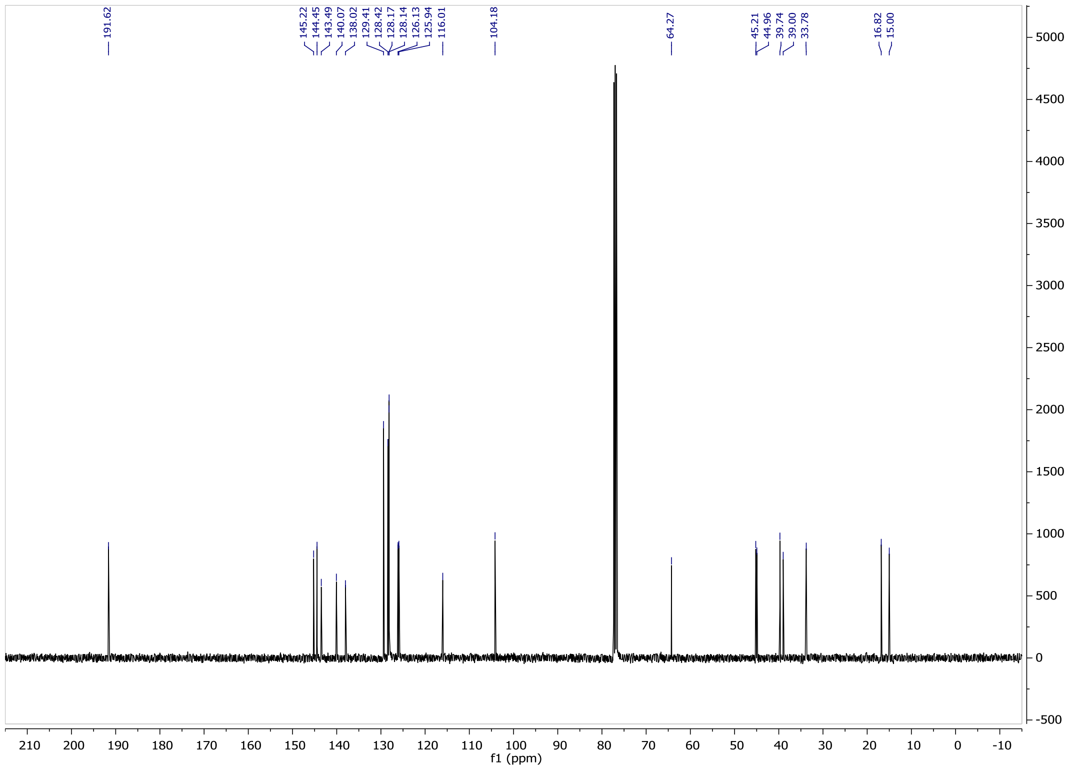
191.62
145.22
144.45
143.49
140.07
138.02
129.41
128.42
128.17
128.14
126.13
125.94
116.01
104.18
64.27
45.21
44.96
39.74
39.00
33.78
16.82
15.00
f1 (ppm)

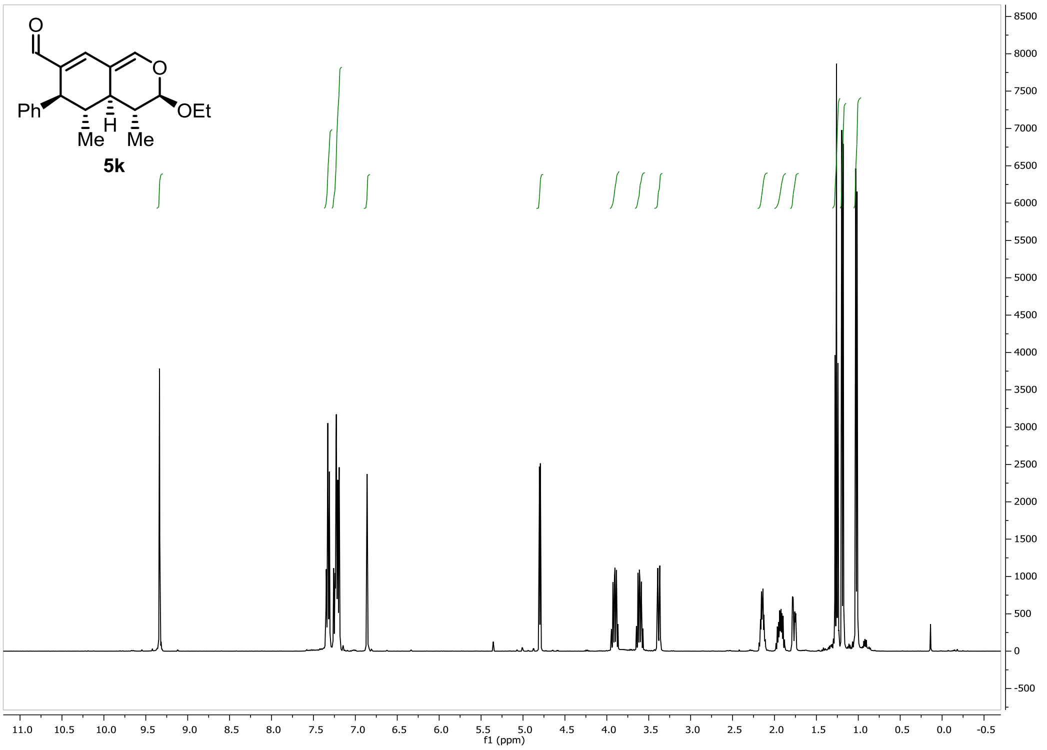
Ph
Me
H
Me
OEt
O
5k
f1 (ppm)

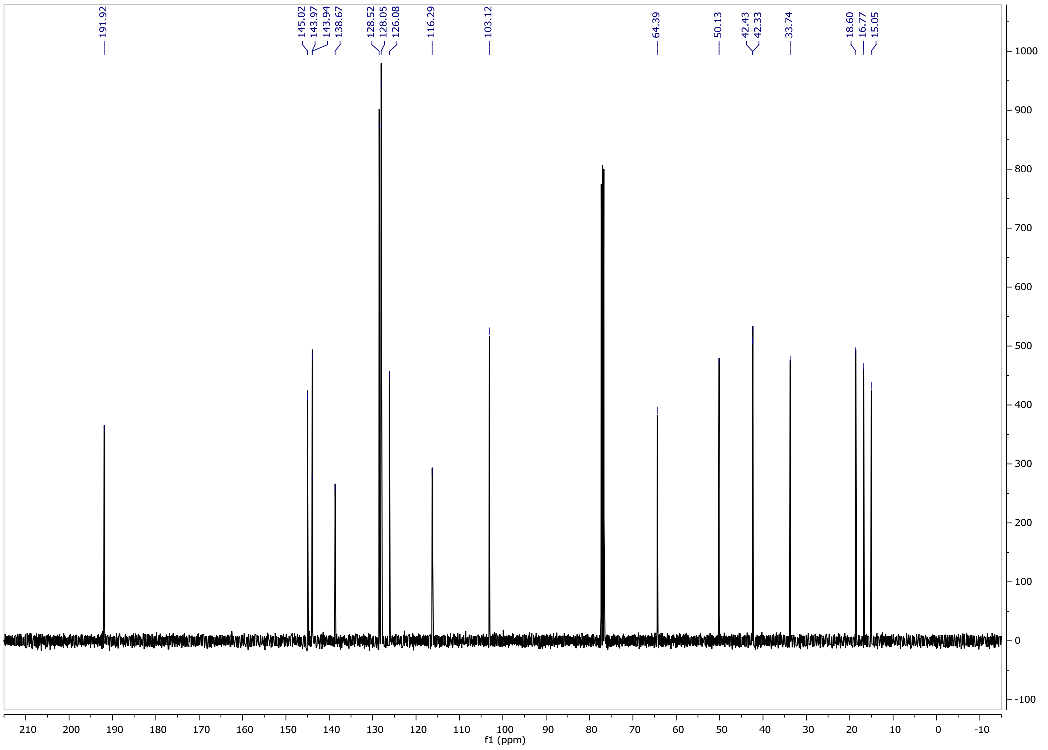
191.92
145.02
143.97
143.94
138.67
128.52
128.05
126.08
116.29
103.12
64.39
50.13
42.43
42.33
33.74
18.60
16.77
15.05
f1 (ppm)

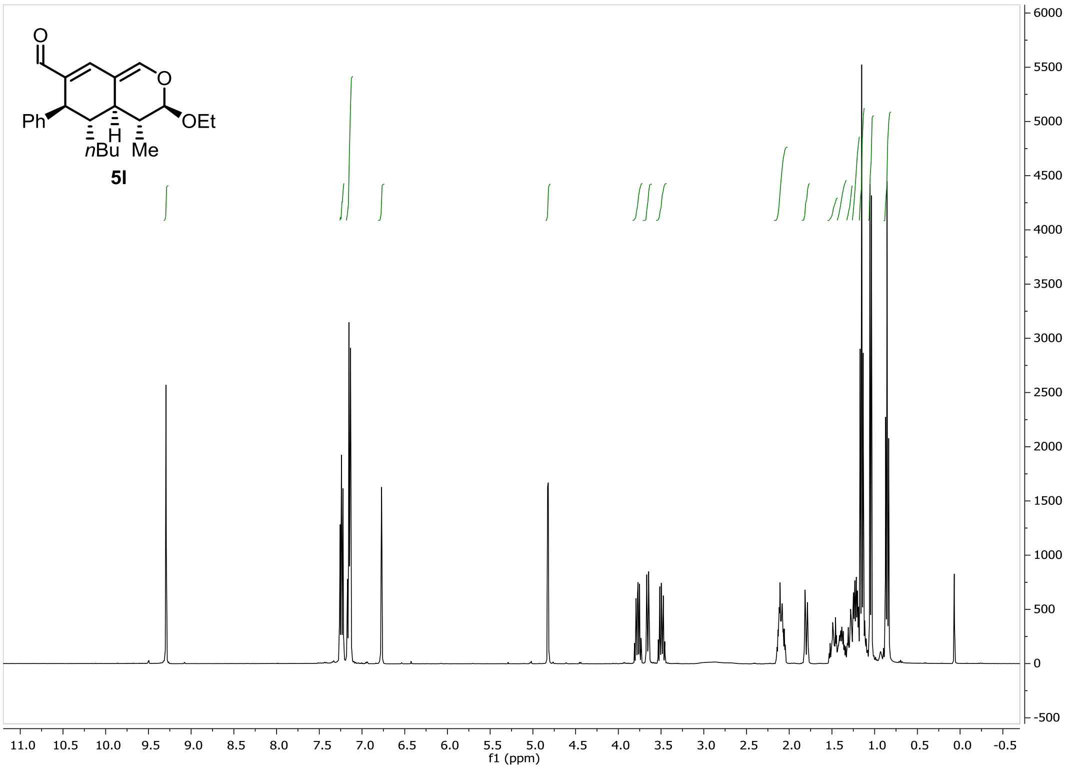
Ph
nBu
H
Me
OEt
O
5l
f1 (ppm)

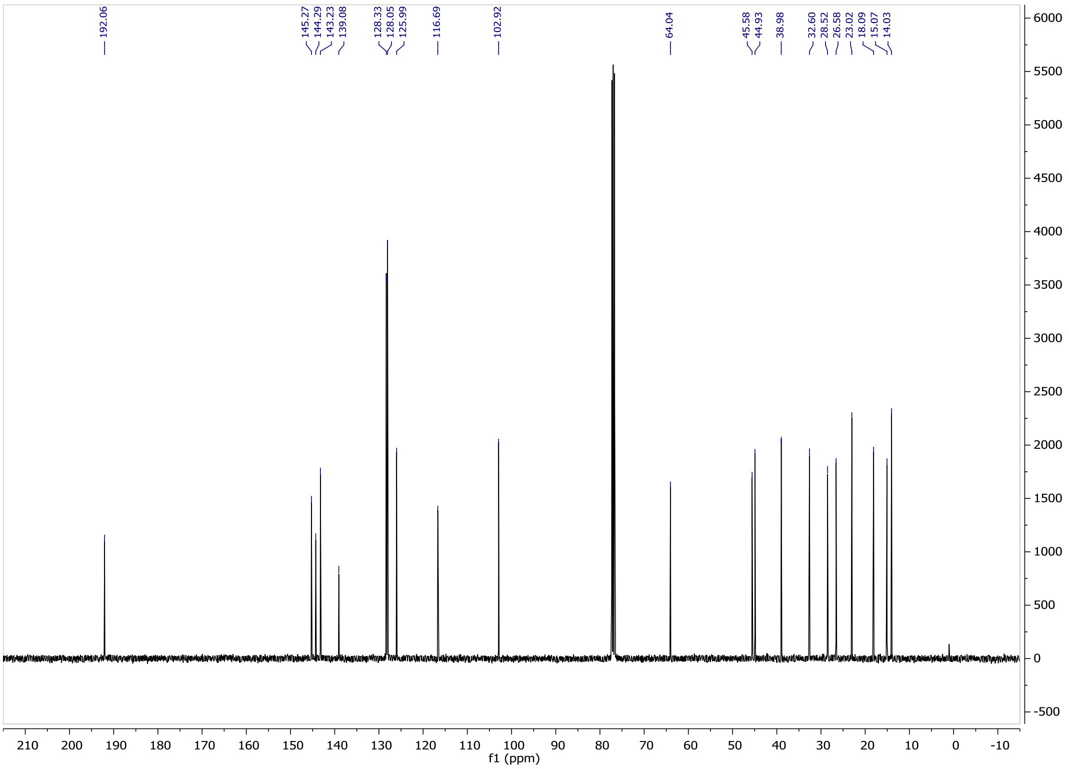
192.06
145.27
144.29
143.23
139.08
128.33
128.05
125.99
116.69
102.92
64.04
45.58
44.93
38.98
32.60
28.52
26.58
23.02
18.09
15.07
14.03
f1 (ppm)

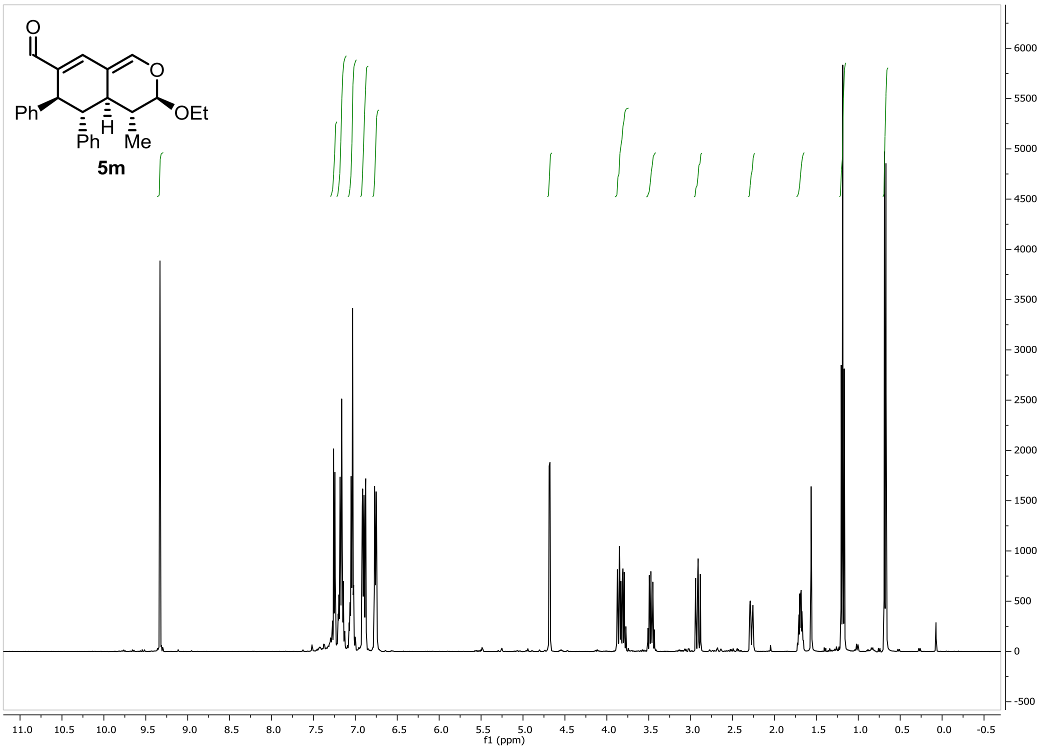
Ph
OEt
Ph
H
Me
5m
f1 (ppm)

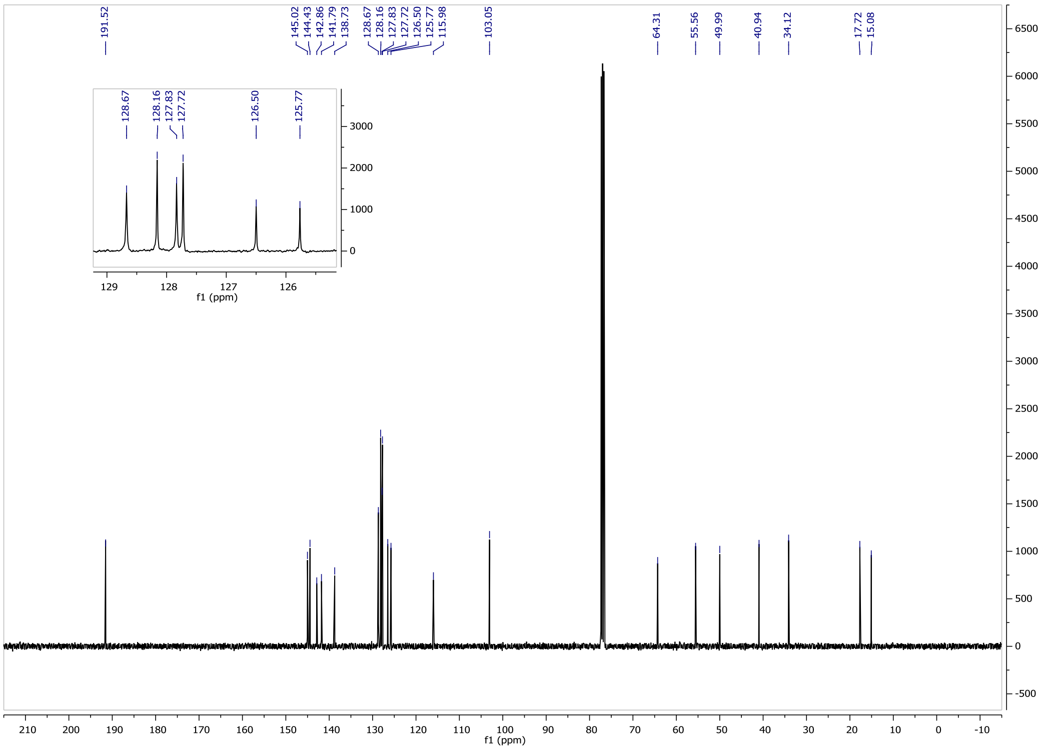
191.52
145.02
144.43
142.86
141.79
138.73
128.67
128.16
127.83
127.72
126.50
125.77
115.98
103.05
64.31
55.56
49.99
40.94
34.12
17.72
15.08
f1 (ppm)

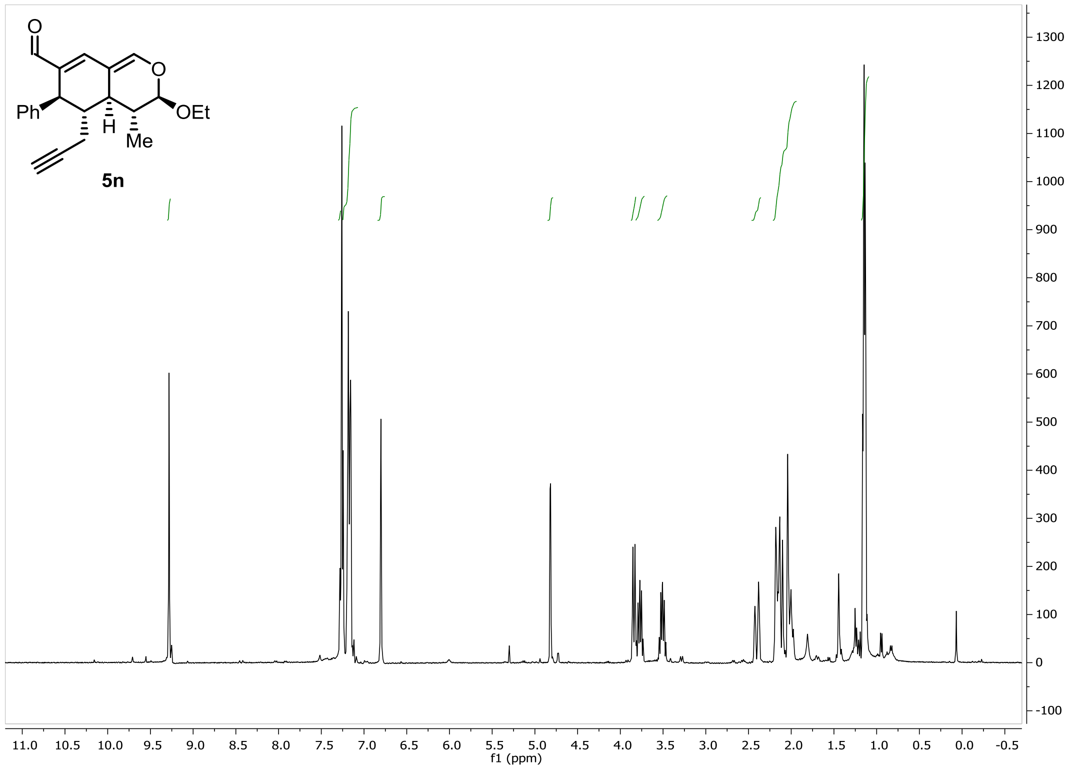
O
O
Ph
H
Me
OEt
5n
f1 (ppm)

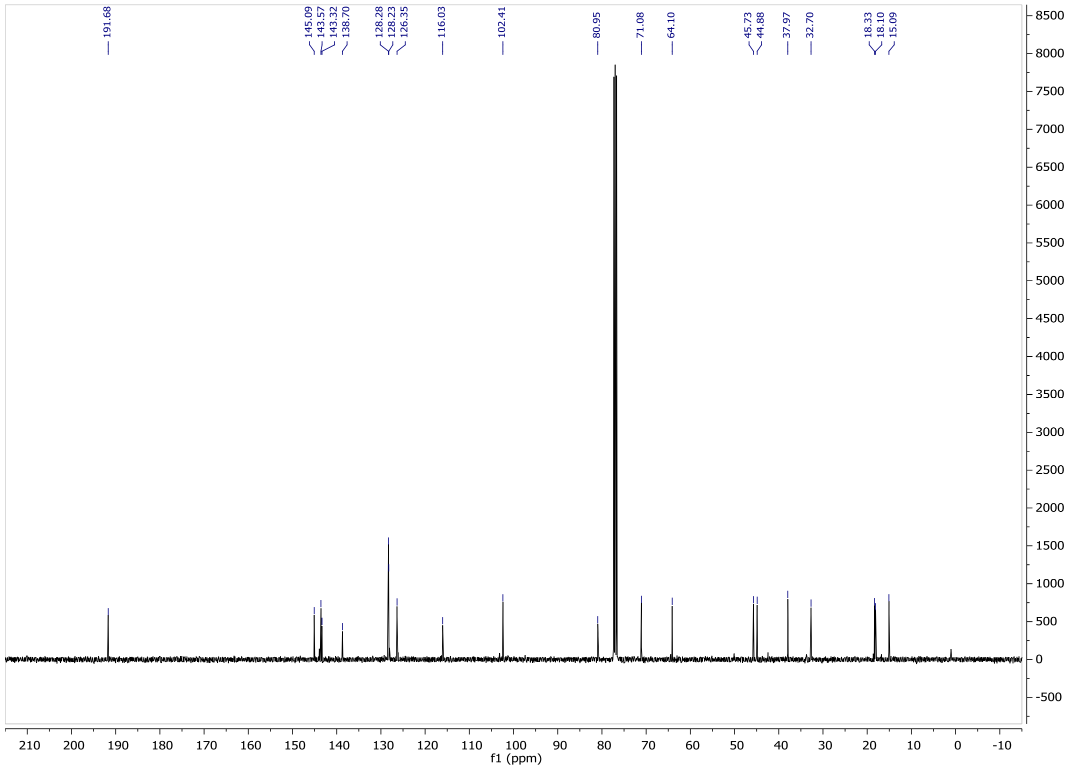
191.68
145.09
143.57
143.32
138.70
128.28
128.23
126.35
116.03
102.41
80.95
71.08
64.10
45.73
44.88
37.97
32.70
18.33
18.10
15.09
f1 (ppm)

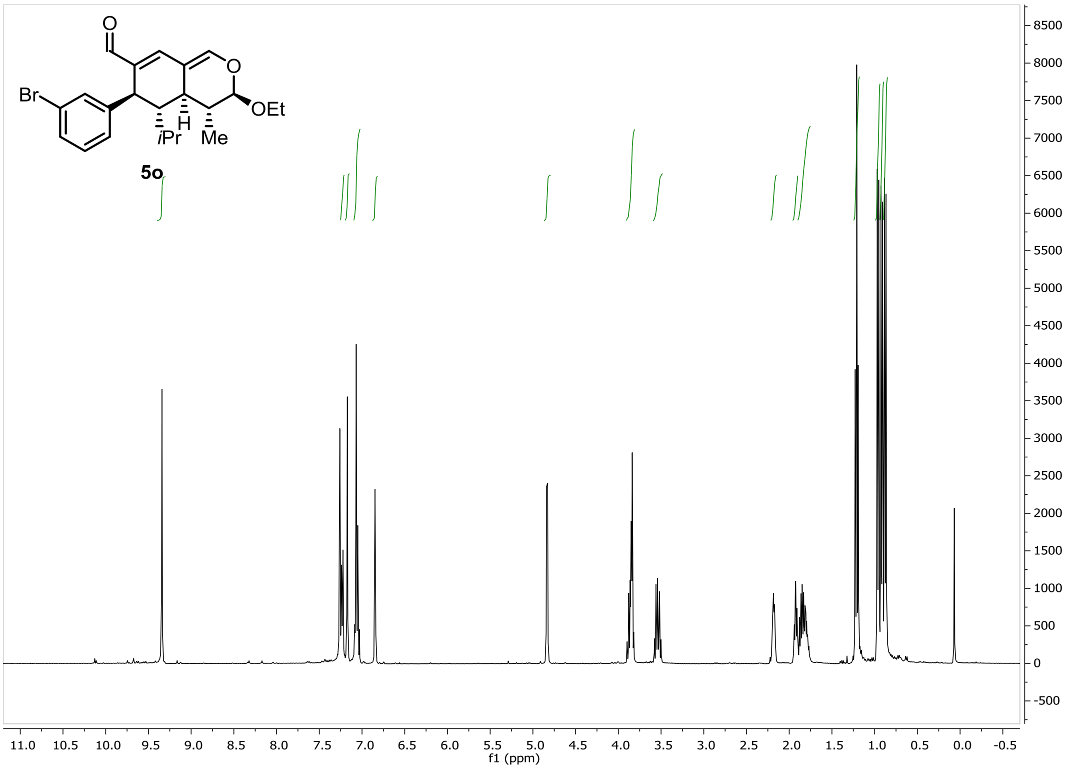

O
O
Br
OEt
H
iPr
Me
5o
f1 (ppm)

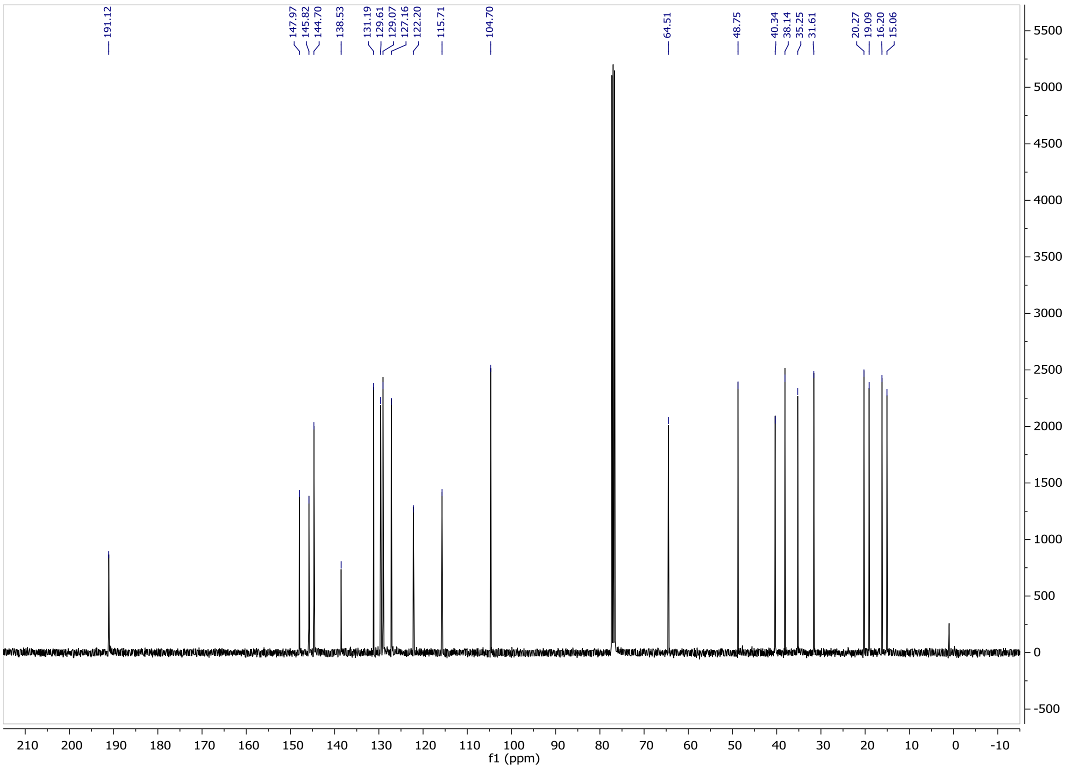

191.12
147.97
145.82
144.70
138.53
131.19
129.61
129.07
127.16
122.20
115.71
104.70
64.51
48.75
40.34
38.14
35.25
31.61
20.27
19.09
16.20
15.06
f1 (ppm)

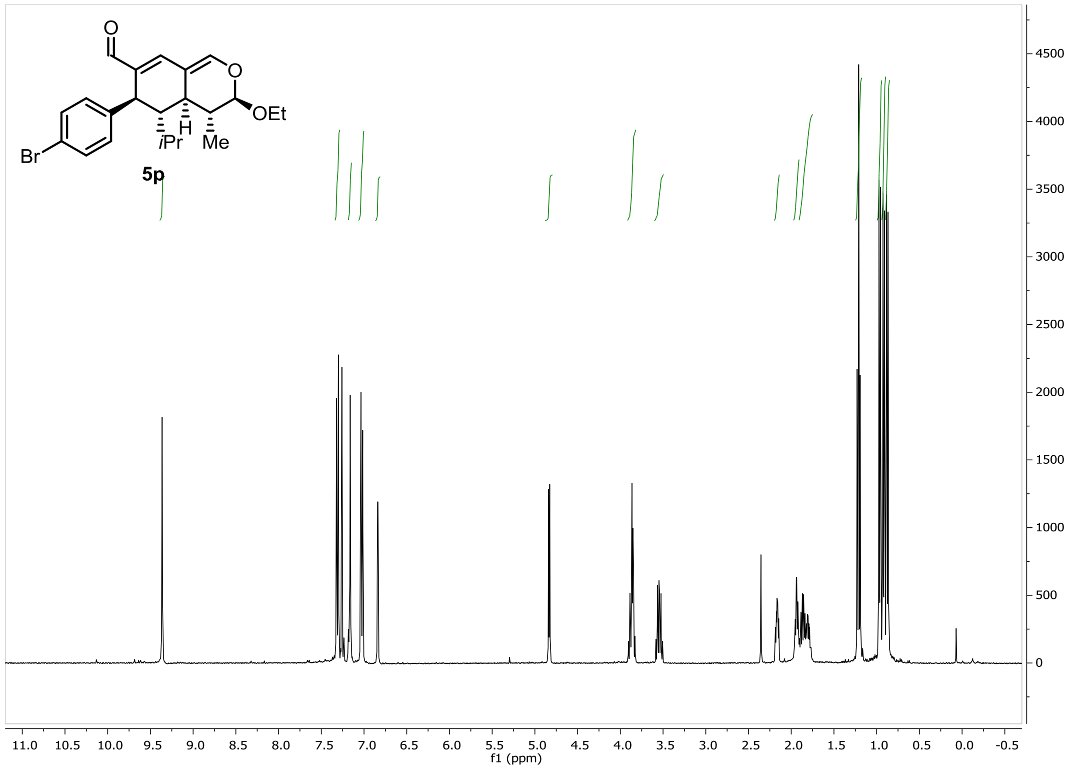
O
OEt
Br
*i*Pr
H
Me
5p
f1 (ppm)

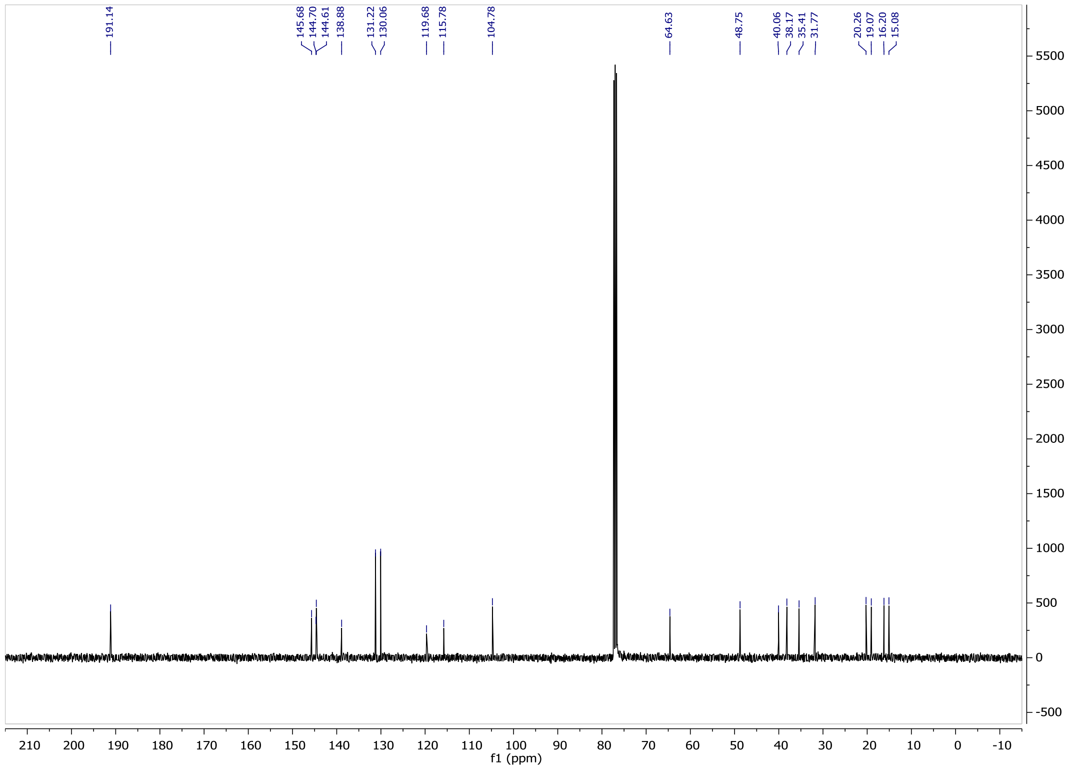
191.14
145.68
144.70
144.61
138.88
131.22
130.06
119.68
115.78
104.78
64.63
48.75
40.06
38.17
35.41
31.77
20.26
19.07
16.20
15.08
f1 (ppm)

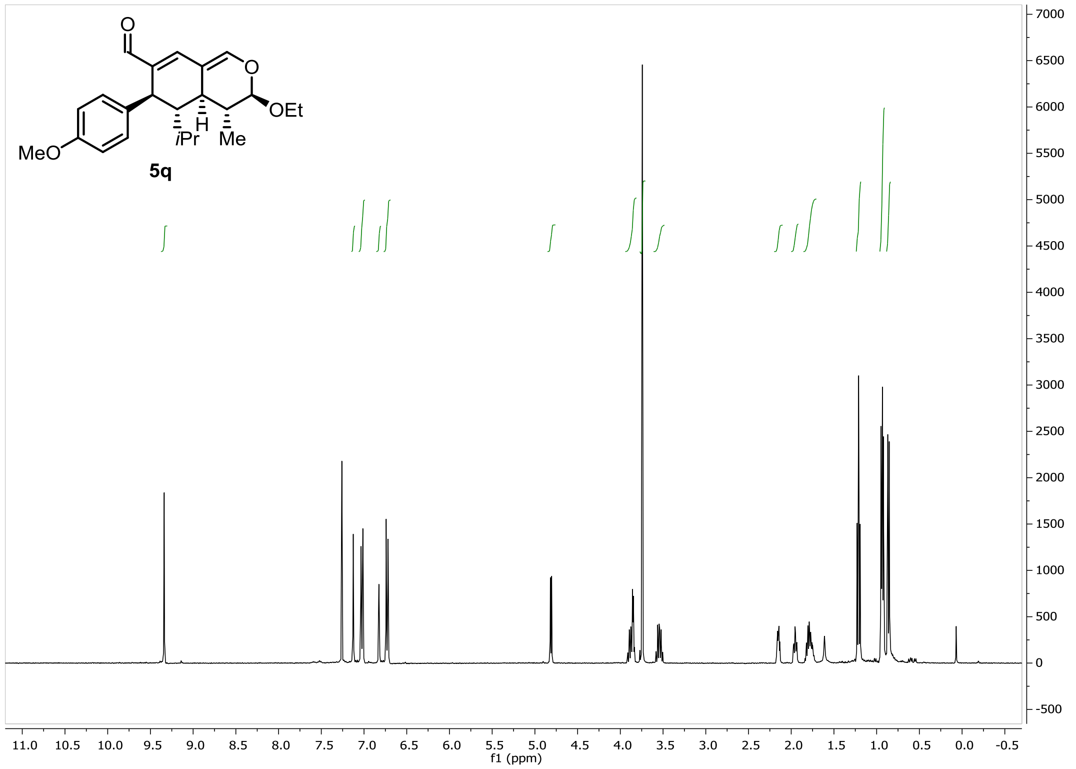
O
O
OEt
H
iPr
Me
MeO
5q
f1 (ppm)

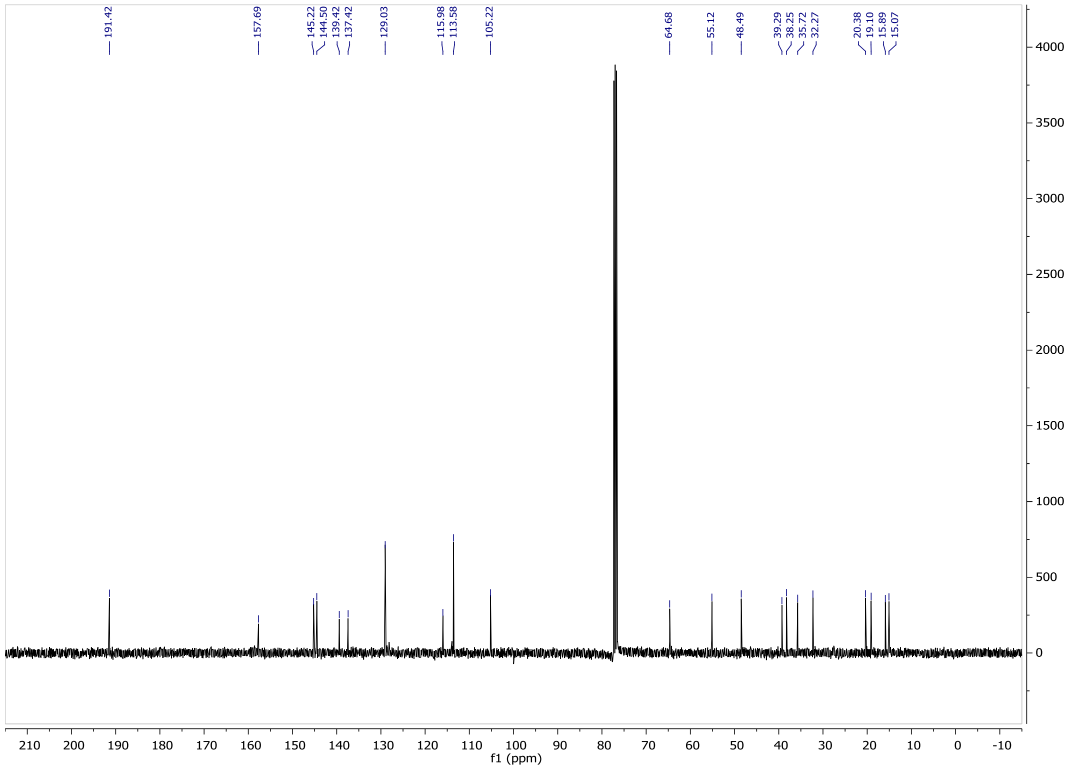
191.42
157.69
145.22
144.50
139.42
137.42
129.03
115.98
113.58
105.22
64.68
55.12
48.49
39.29
38.25
35.72
32.27
20.38
19.10
15.89
15.07
f1 (ppm)

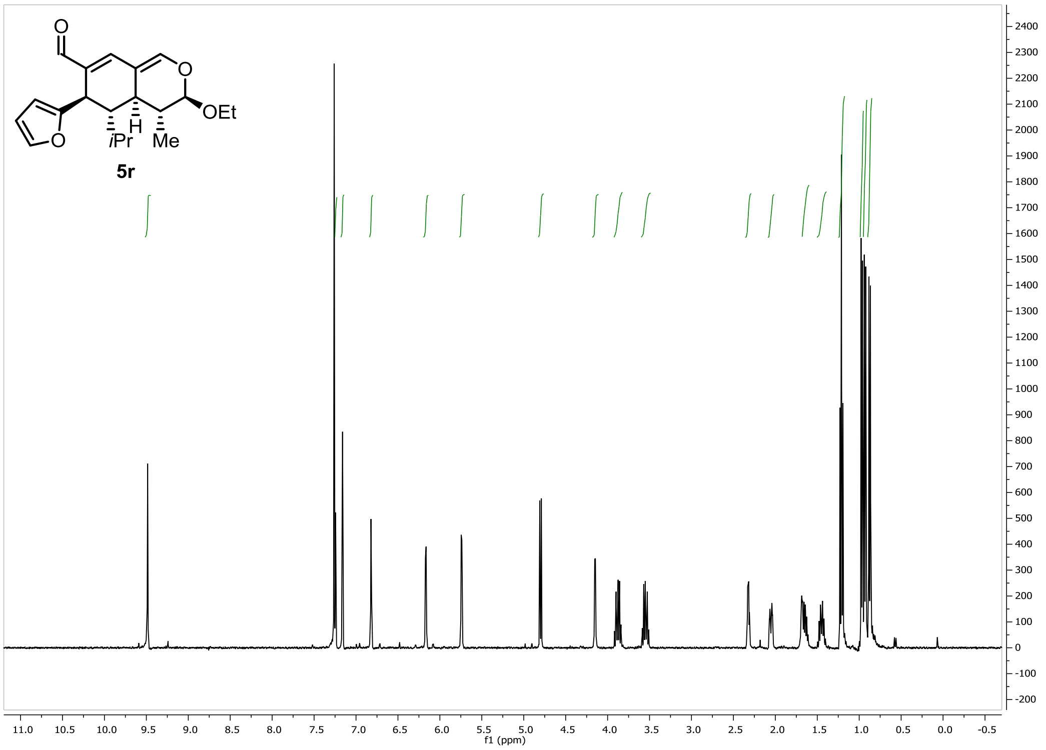

O
O
OEt
O
H
iPr
Me
5r
f1 (ppm)

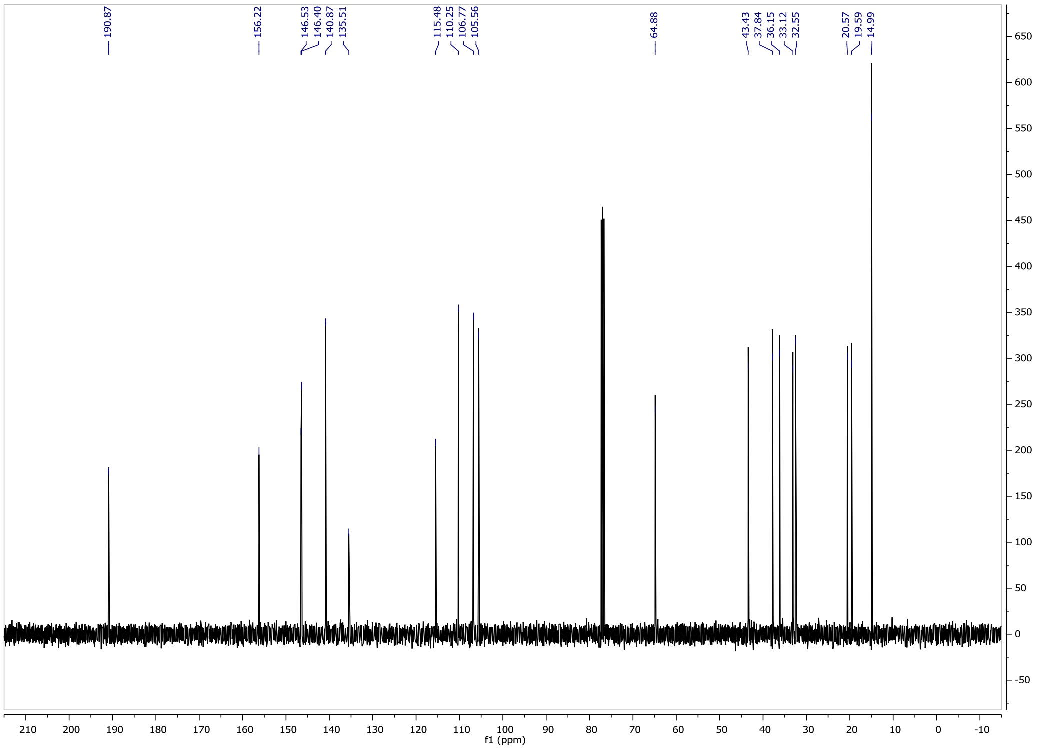

190.87
156.22
146.53
146.40
140.87
135.51
115.48
110.25
106.77
105.56
64.88
43.43
37.84
36.15
33.12
32.55
20.57
19.59
14.99
f1 (ppm)

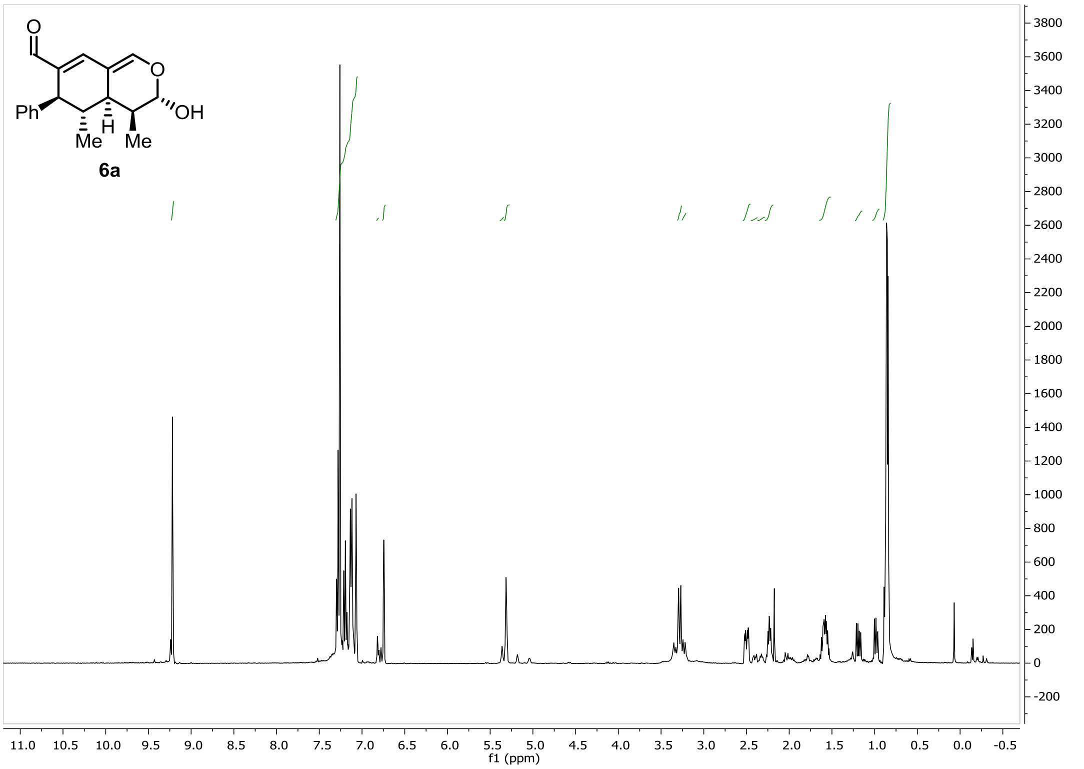
O
Ph
OH
H
Me
Me
6a
f1 (ppm)

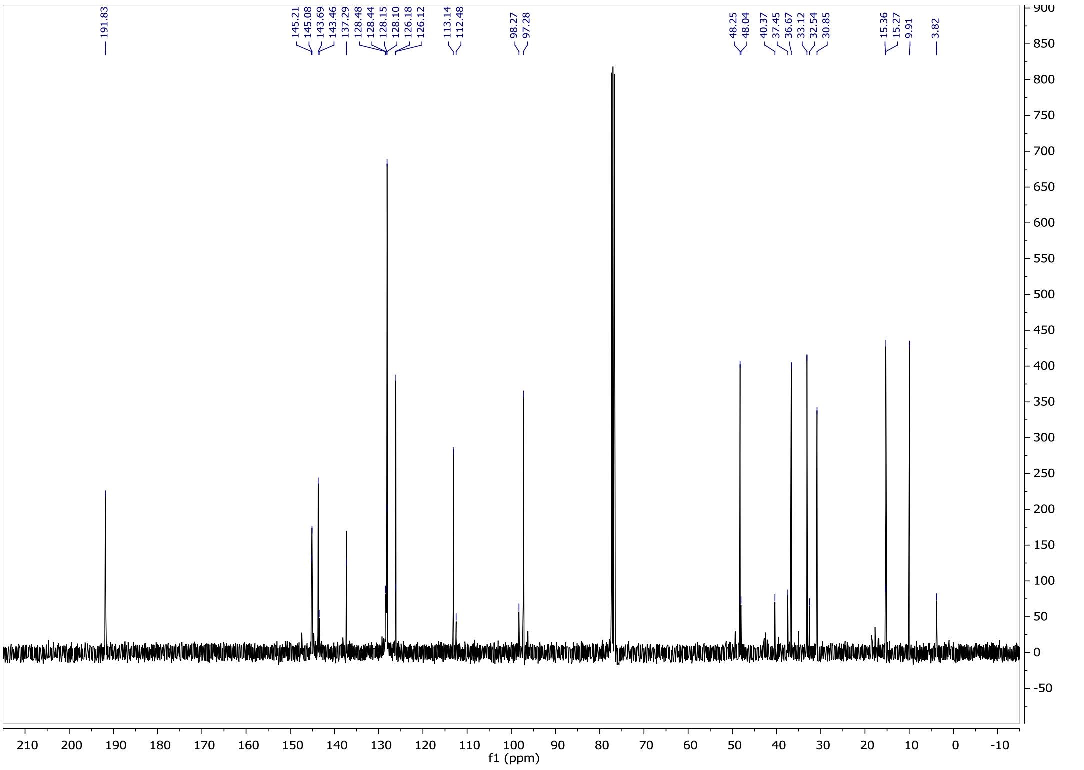
191.83
145.21
145.08
143.69
143.46
137.29
128.48
128.44
128.15
128.10
126.18
126.12
113.14
112.48
98.27
97.28
48.25
48.04
40.37
37.45
36.67
33.12
32.54
30.85
15.36
15.27
9.91
3.82
f1 (ppm)

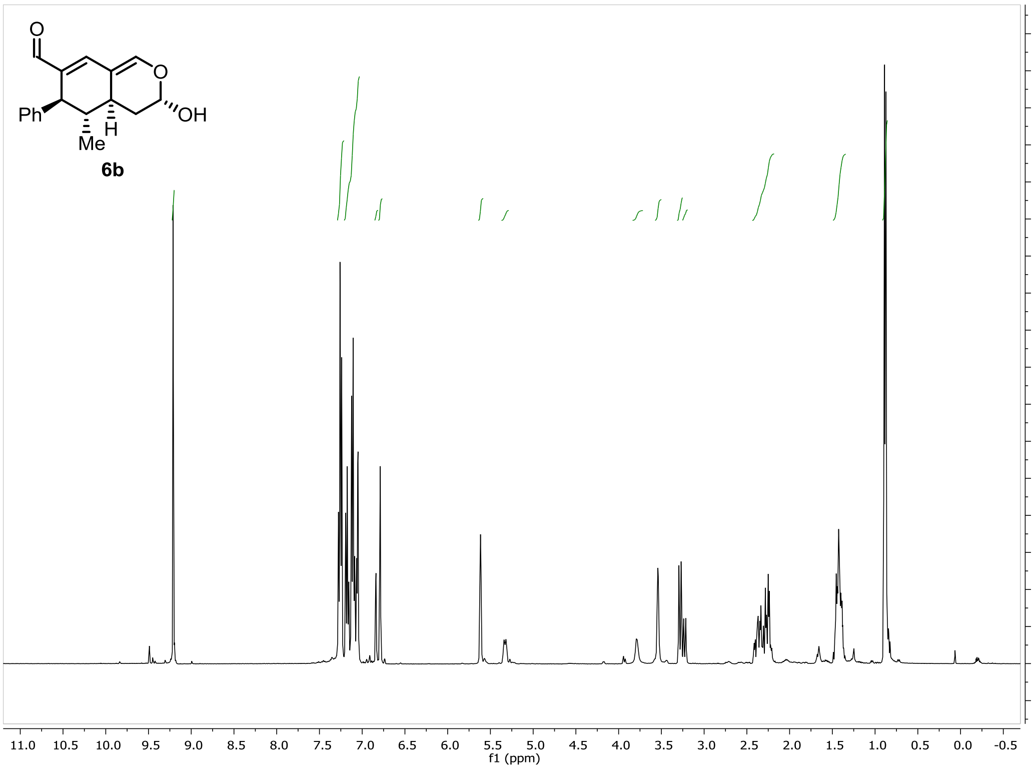

O
Ph
OH
H
Me
6b
f1 (ppm)

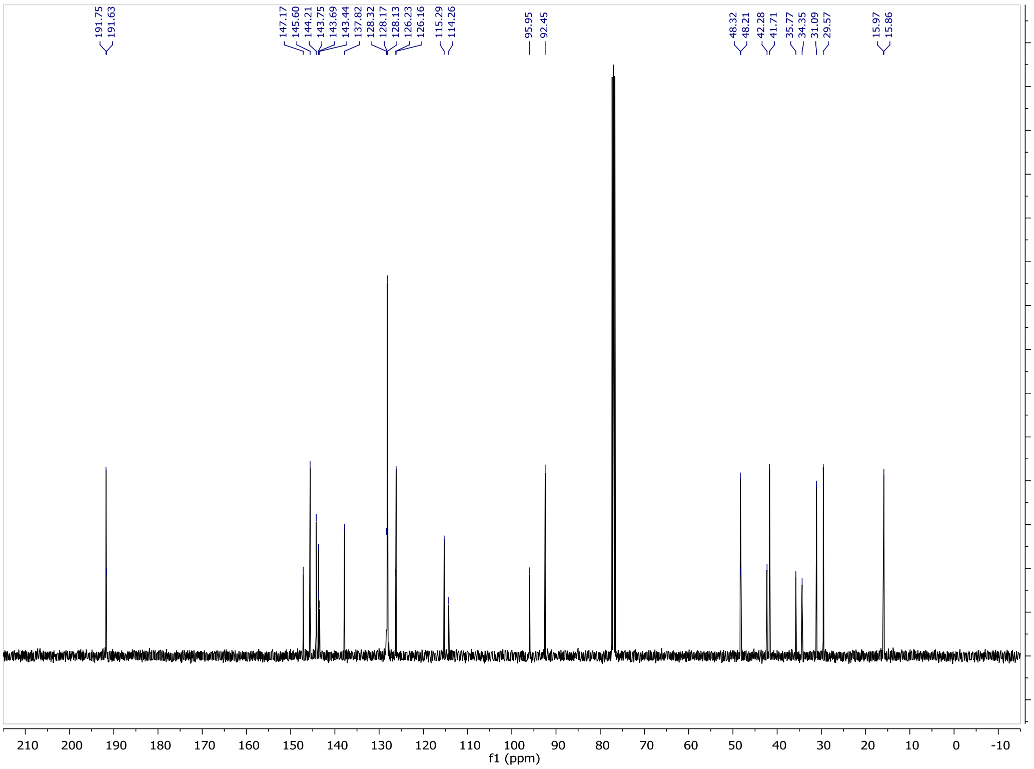

191.75
191.63
147.17
145.60
144.21
143.75
143.69
143.44
137.82
128.32
128.17
128.13
126.23
126.16
115.29
114.26
95.95
92.45
48.32
48.21
42.28
41.71
35.77
34.35
31.09
29.57
15.97
15.86
f1 (ppm)

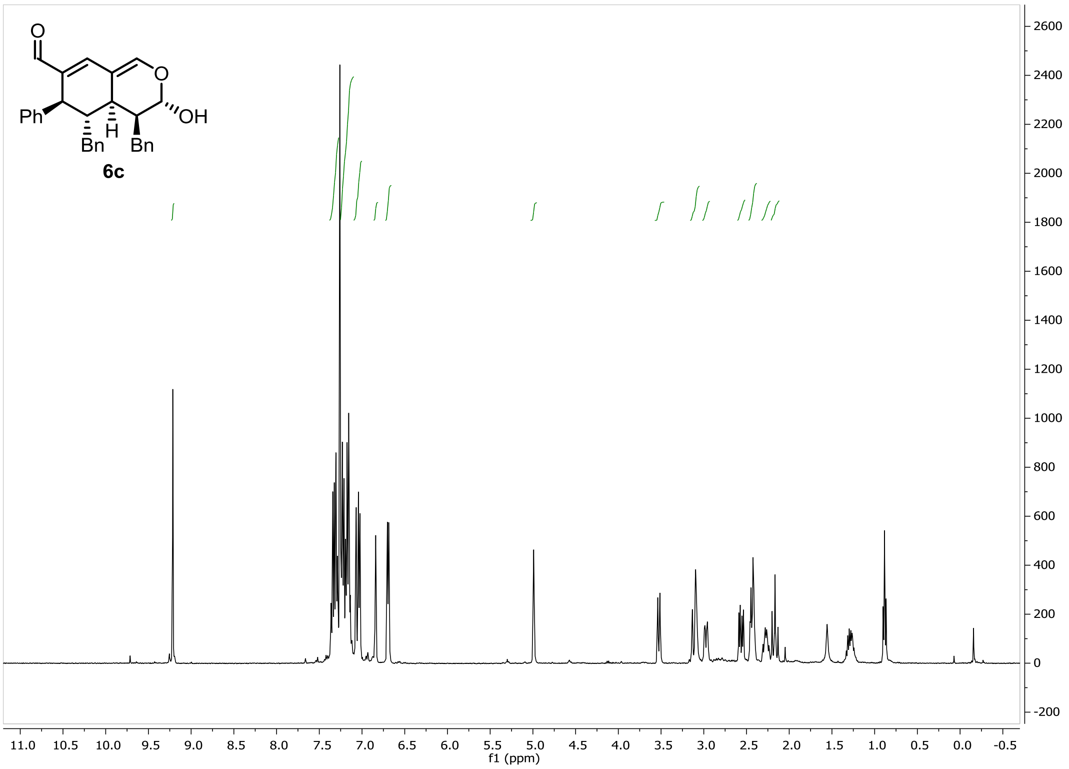
Ph
OH
Bn
H
Bn
6c
f1 (ppm)

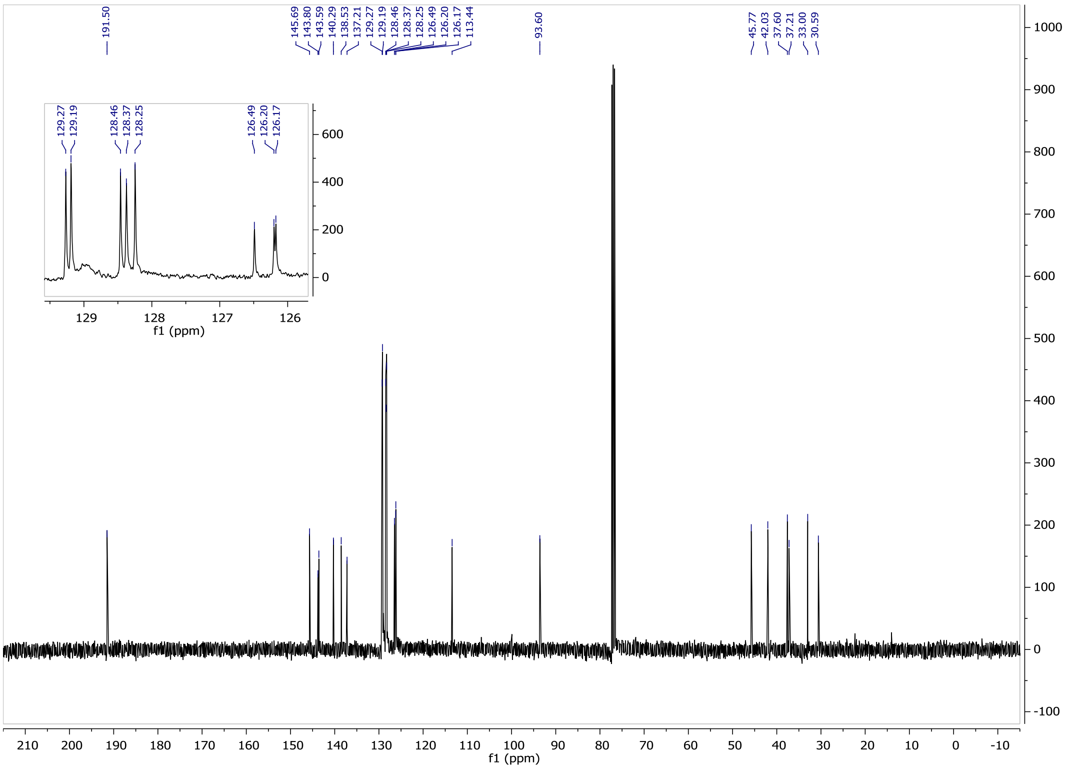
191.50
145.69
143.80
143.59
140.29
138.53
137.21
129.27
129.19
128.46
128.37
128.25
126.49
126.20
126.17
113.44
93.60
45.77
42.03
37.60
37.21
33.00
30.59
f1 (ppm)

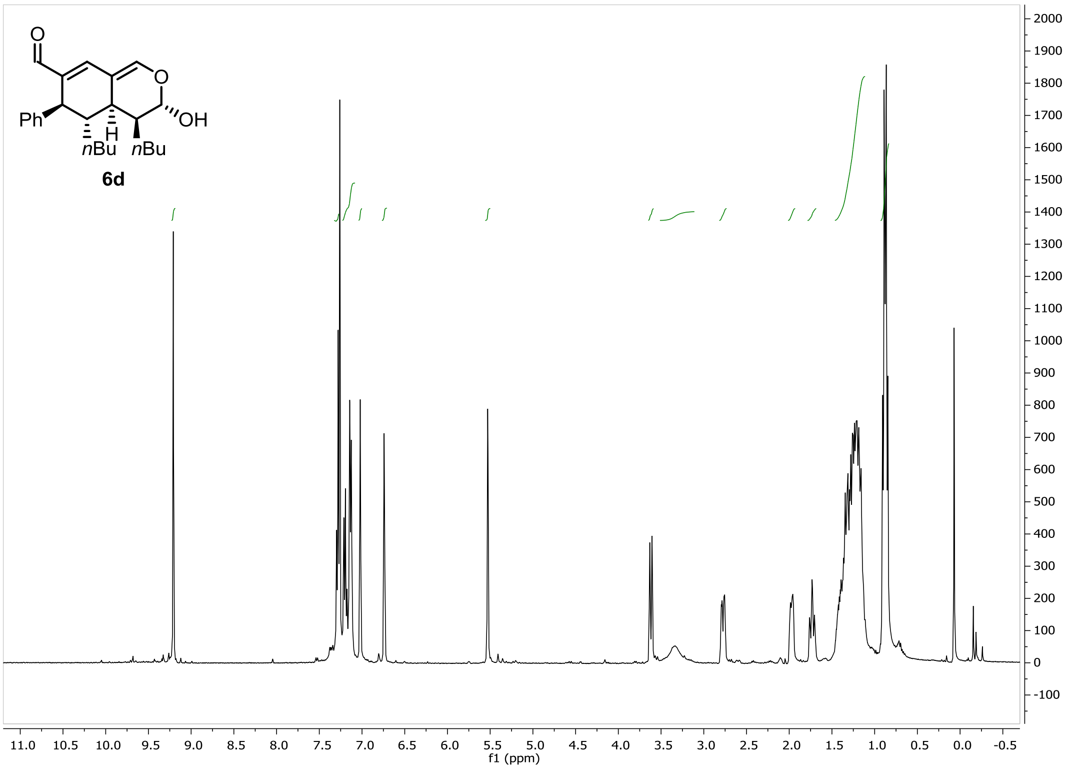
Ph
OH
nBu
nBu
H
O
O
6d
f1 (ppm)

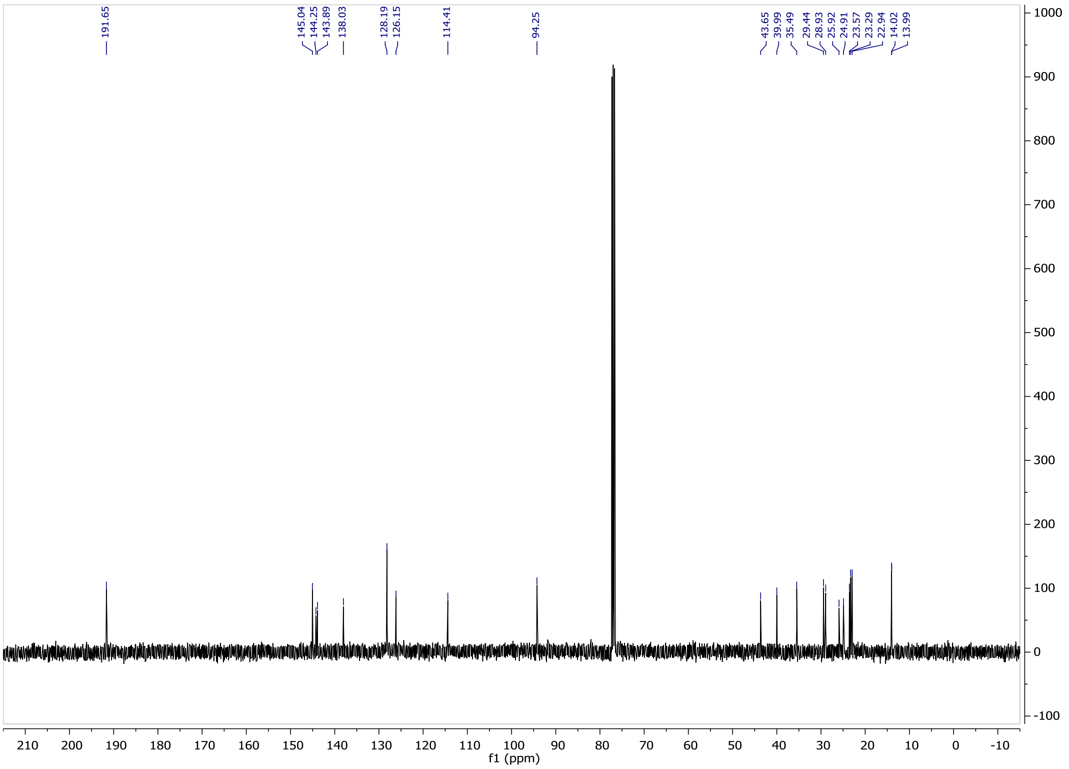
191.65
145.04
144.25
143.89
138.03
128.19
126.15
114.41
94.25
43.65
39.99
35.49
29.44
28.93
25.92
24.91
23.57
23.29
22.94
14.02
13.99
f1 (ppm)

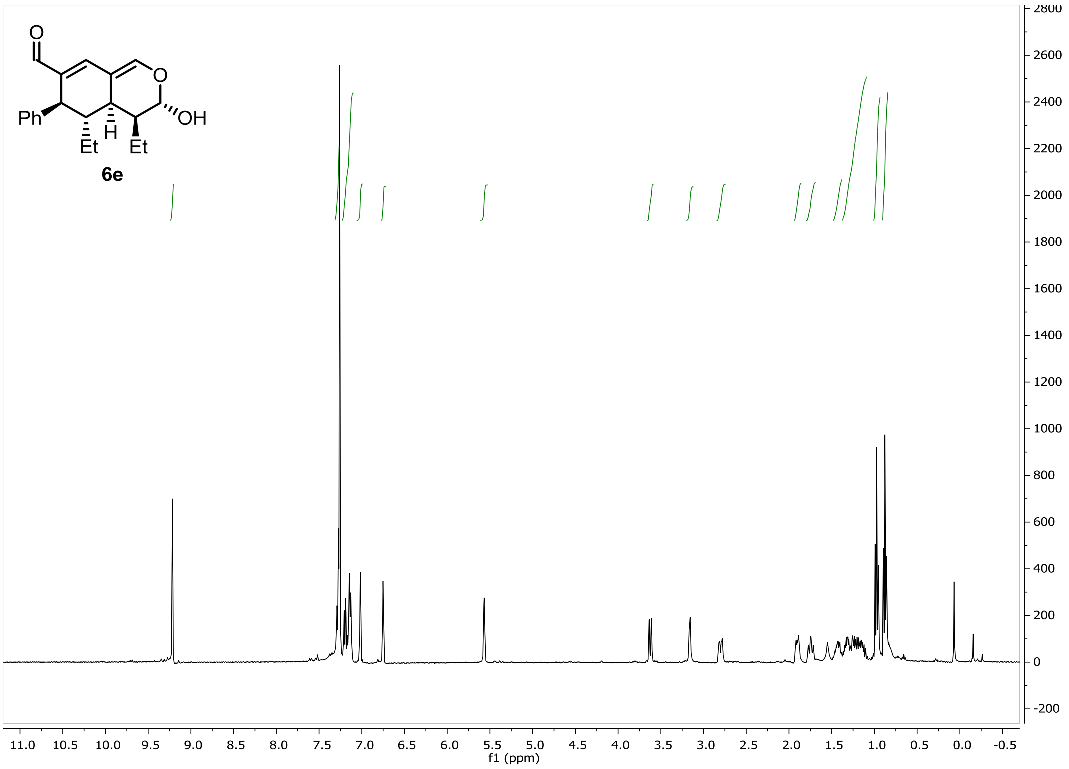
O
Ph
OH
H
Et
Et
6e
f1 (ppm)

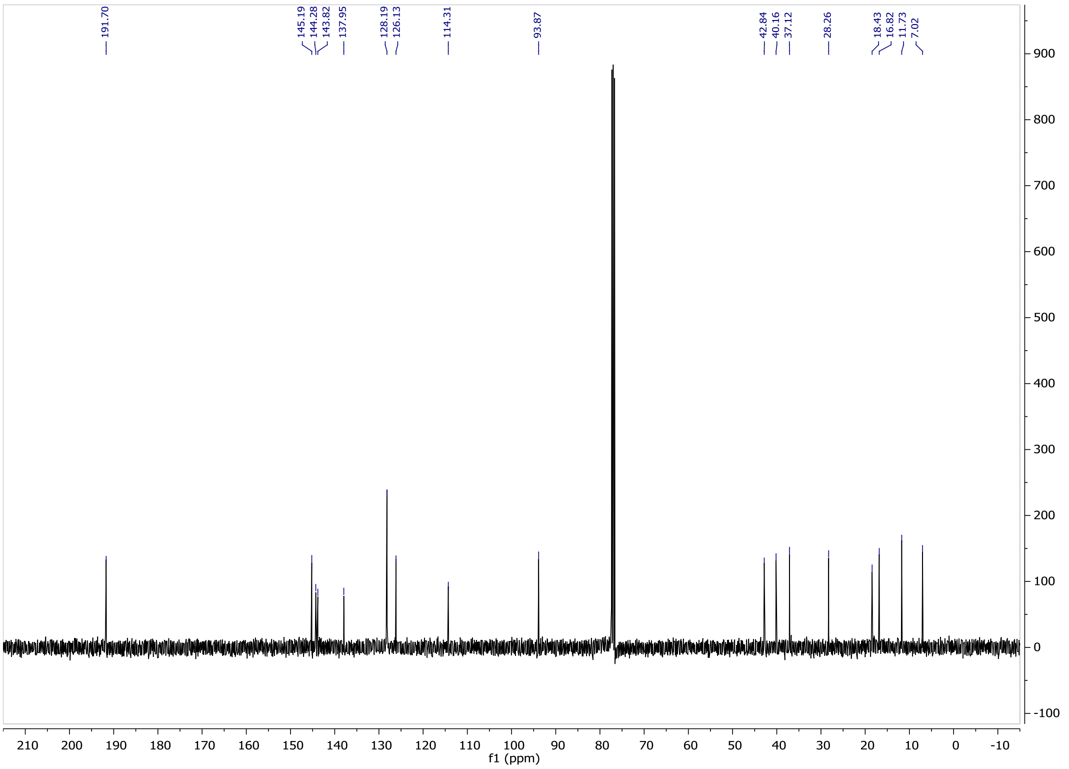
191.70
145.19
144.28
143.82
137.95
128.19
126.13
114.31
93.87
42.84
40.16
37.12
28.26
18.43
16.82
11.73
7.02
f1 (ppm)

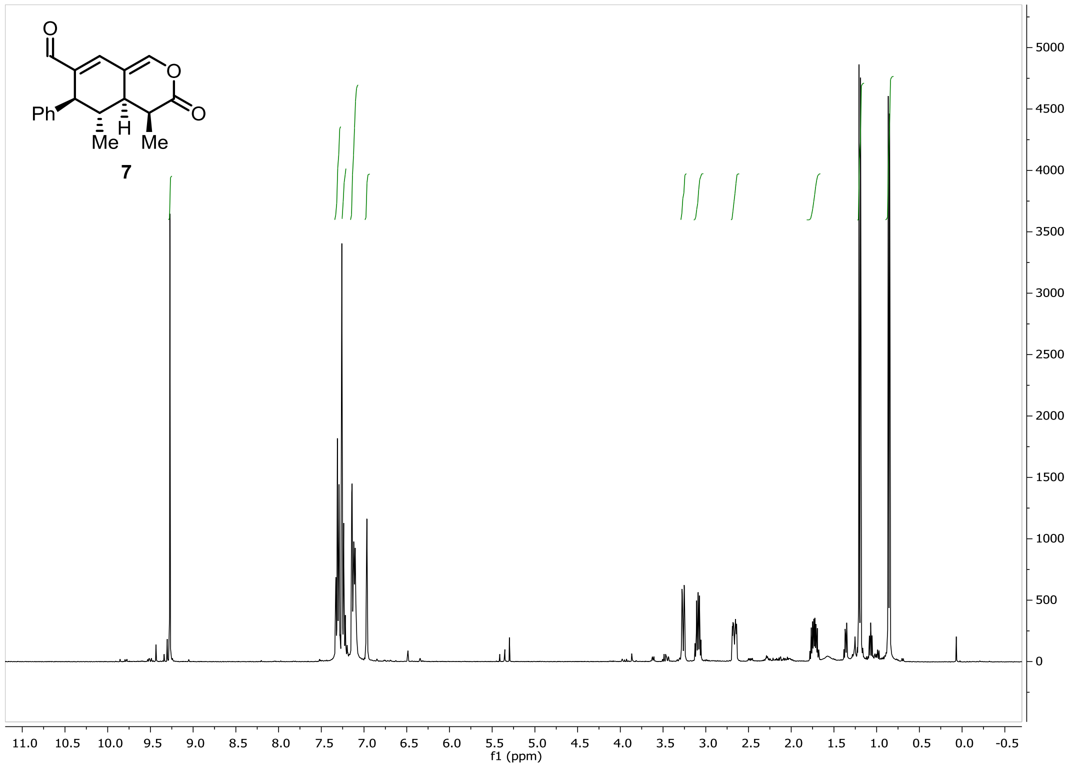

Ph
Me
H
Me
O
O
O
7
f1 (ppm)

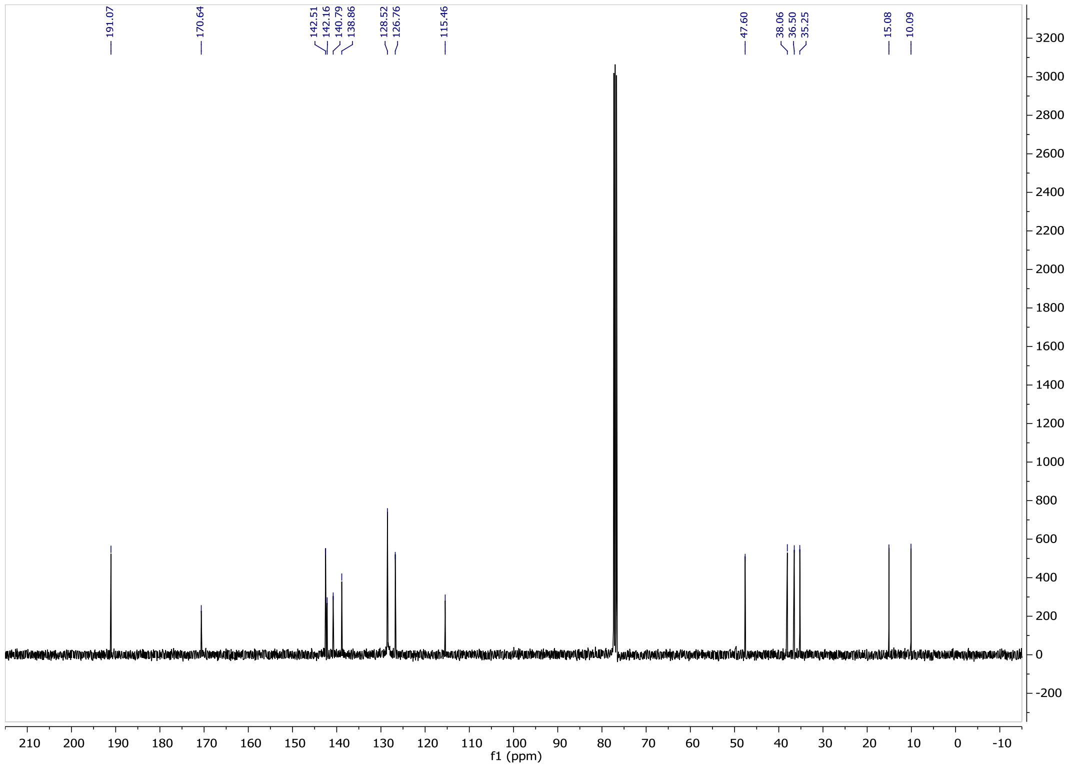

191.07
170.64
142.51
142.16
140.79
138.86
128.52
126.76
115.46
47.60
38.06
36.50
35.25
15.08
10.09
f1 (ppm)

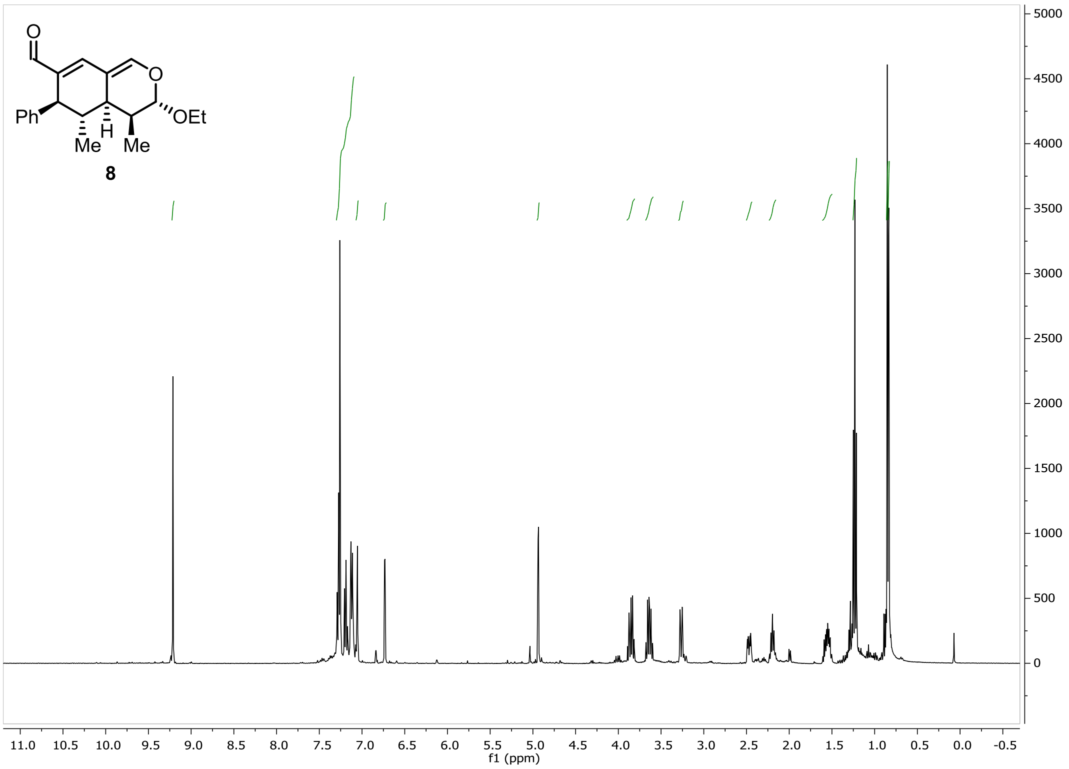
O
Ph
H
OEt
Me
Me
8
f1 (ppm)

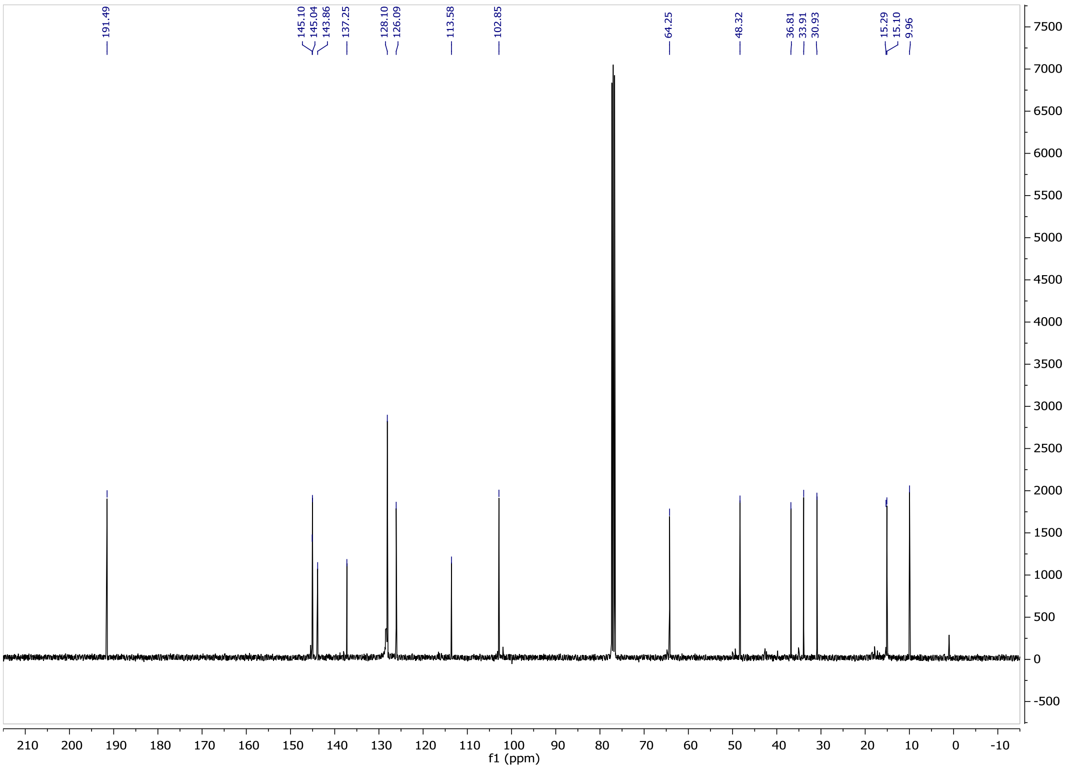
191.49
145.10
145.04
143.86
137.25
128.10
126.09
113.58
102.85
64.25
48.32
36.81
33.91
30.93
15.29
15.10
9.96
f1 (ppm)

9. UPC^2 traces

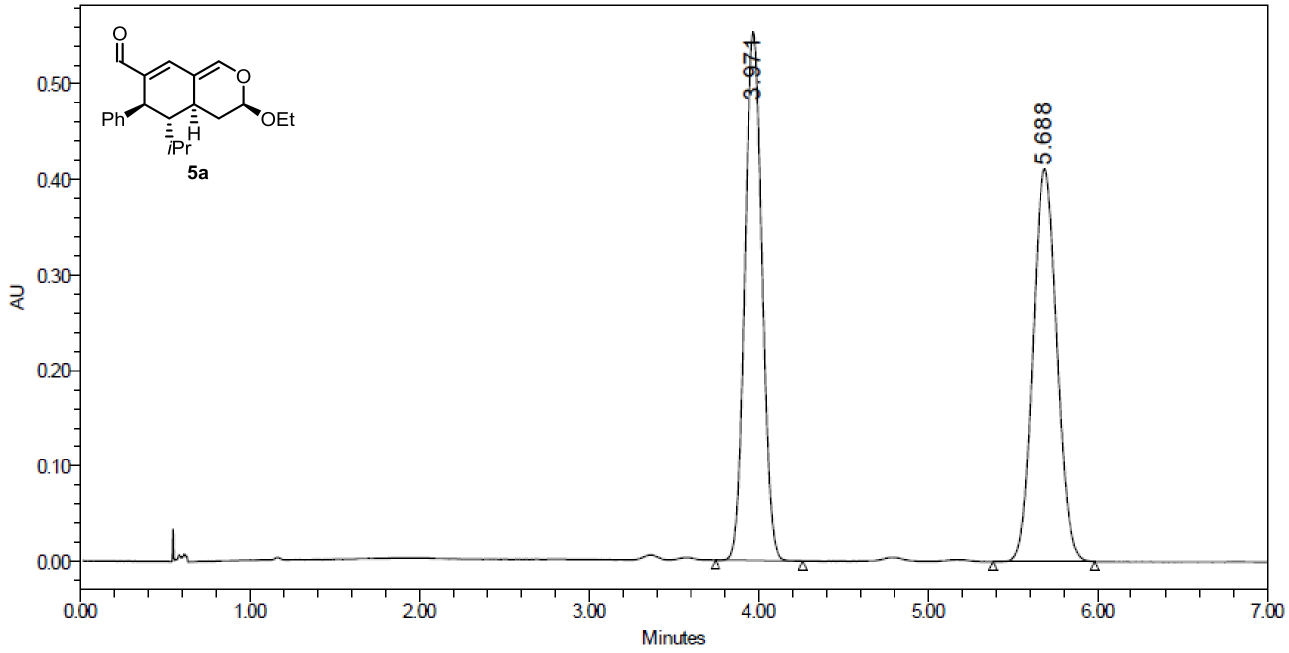

	Retention Time (min)	% Area
1	3.971	49.28
2	5.688	50.72

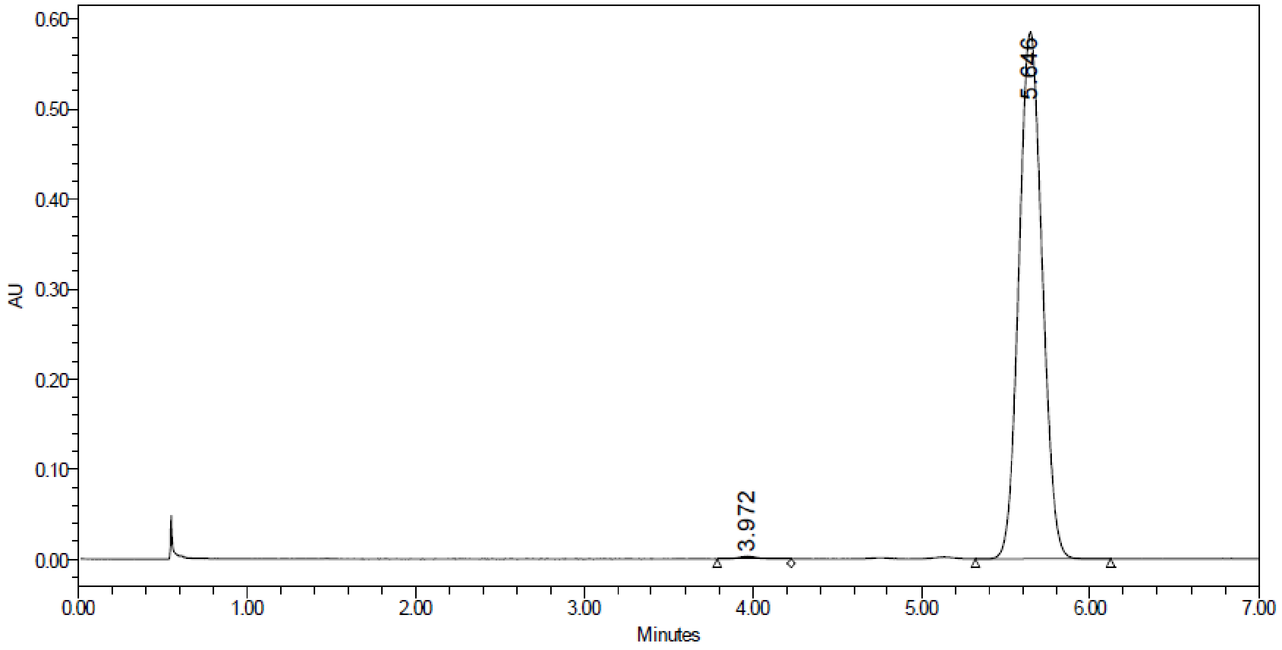

	Retention Time (min)	% Area
1	3.972	0.43
2	5.646	99.57

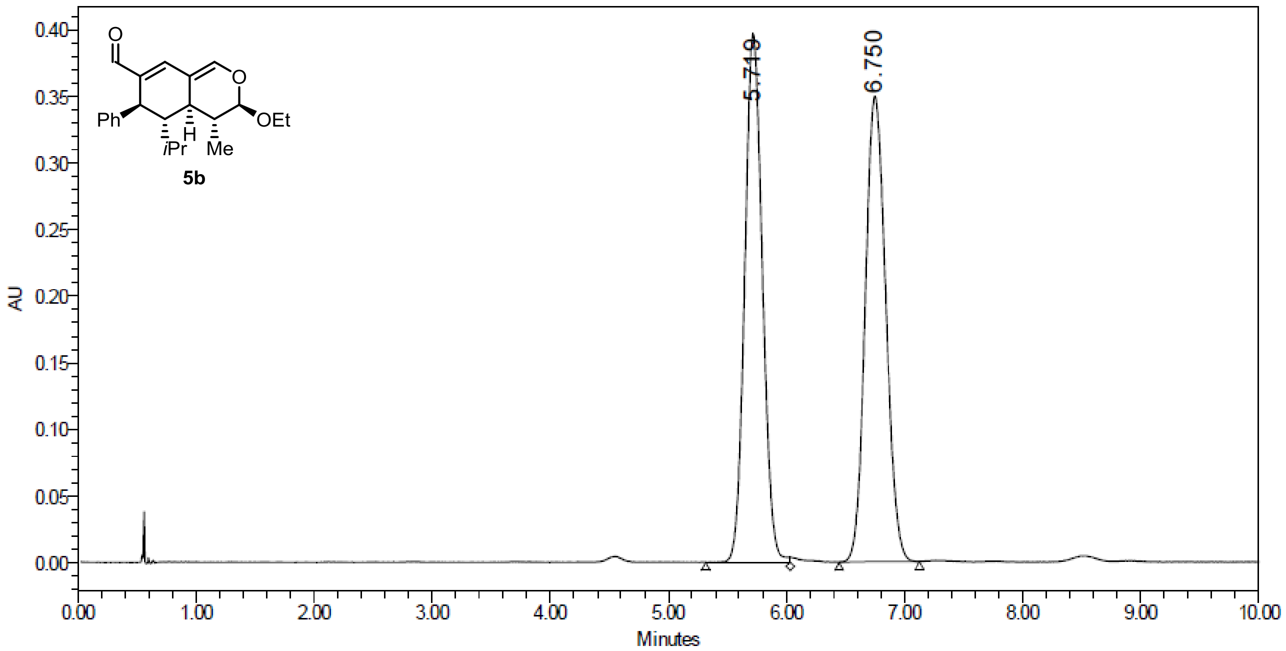

	Retention Time (min)	% Area
1	5.719	49.58
2	6.750	50.42

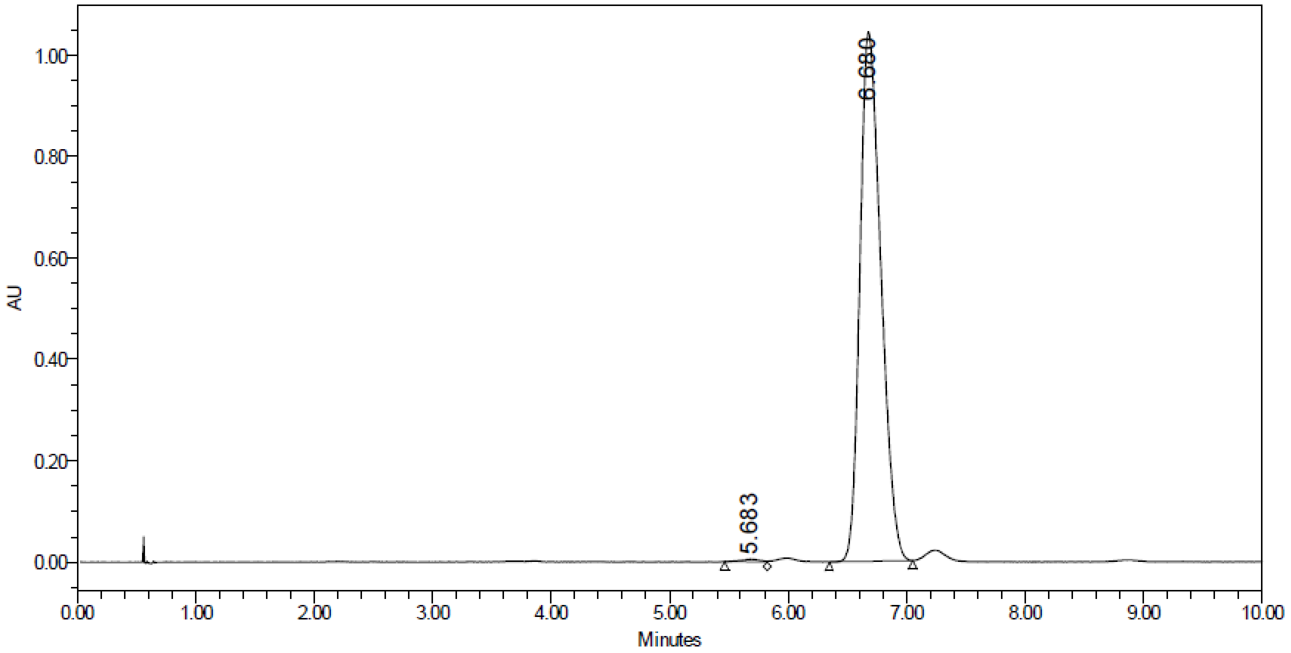

	Retention Time (min)	% Area
1	5.683	0.47
2	6.680	99.53

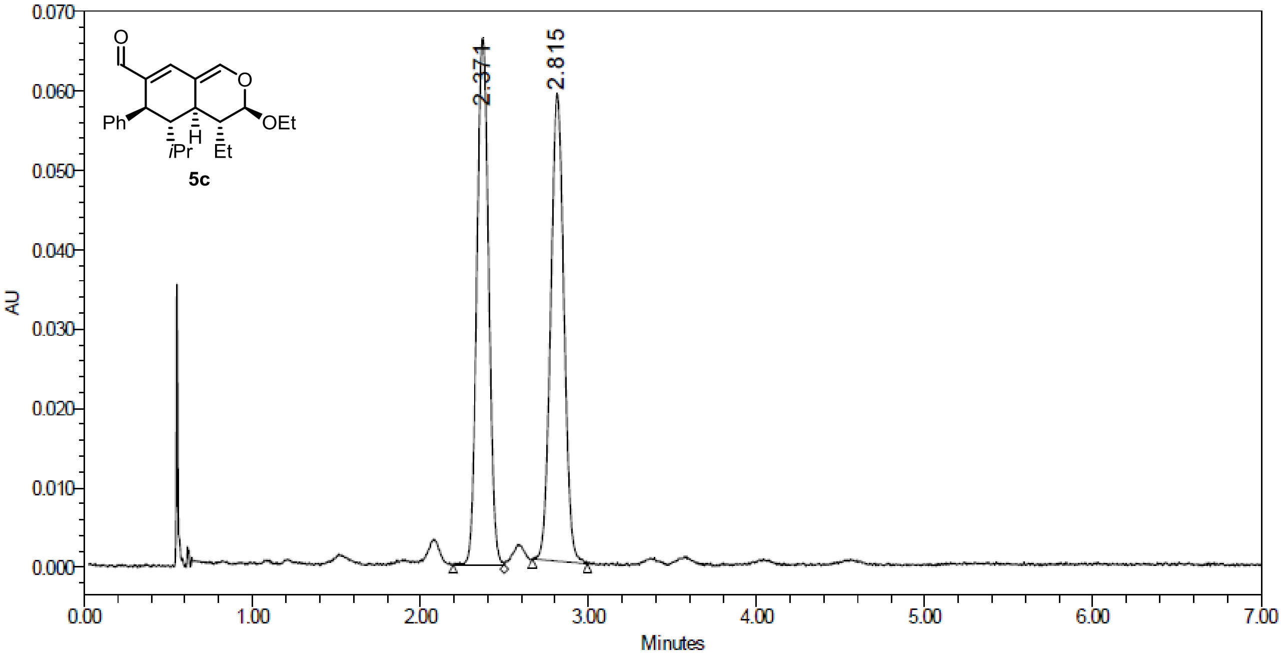

	Retention Time (min)	% Area
1	2.371	49.58
2	2.815	50.42

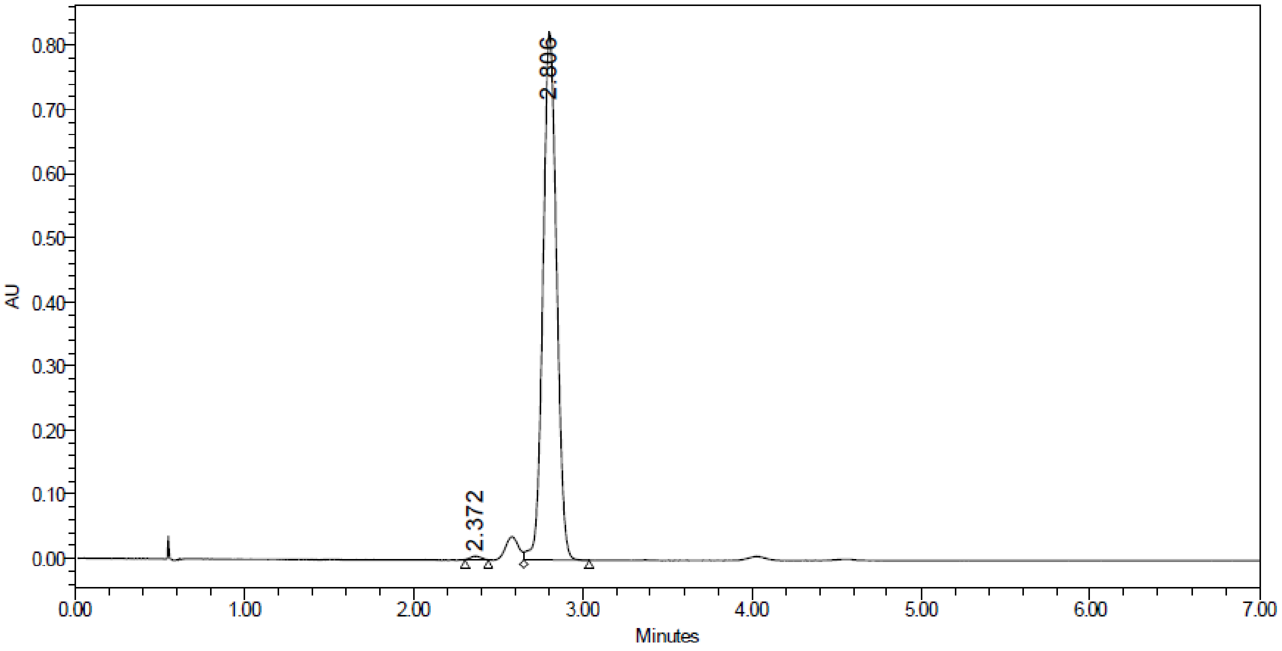

	Retention Time (min)	% Area
1	2.372	0.47
2	2.806	99.53

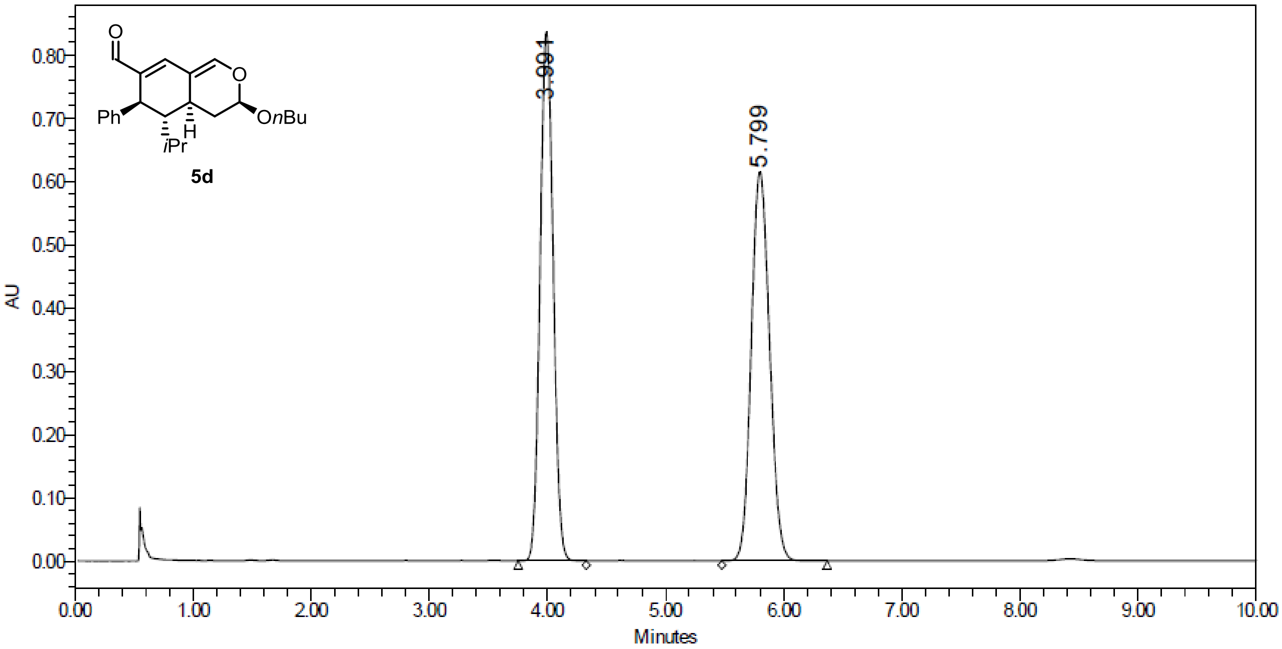

	Retention Time (min)	% Area
1	3.991	49.31
2	5.799	50.69

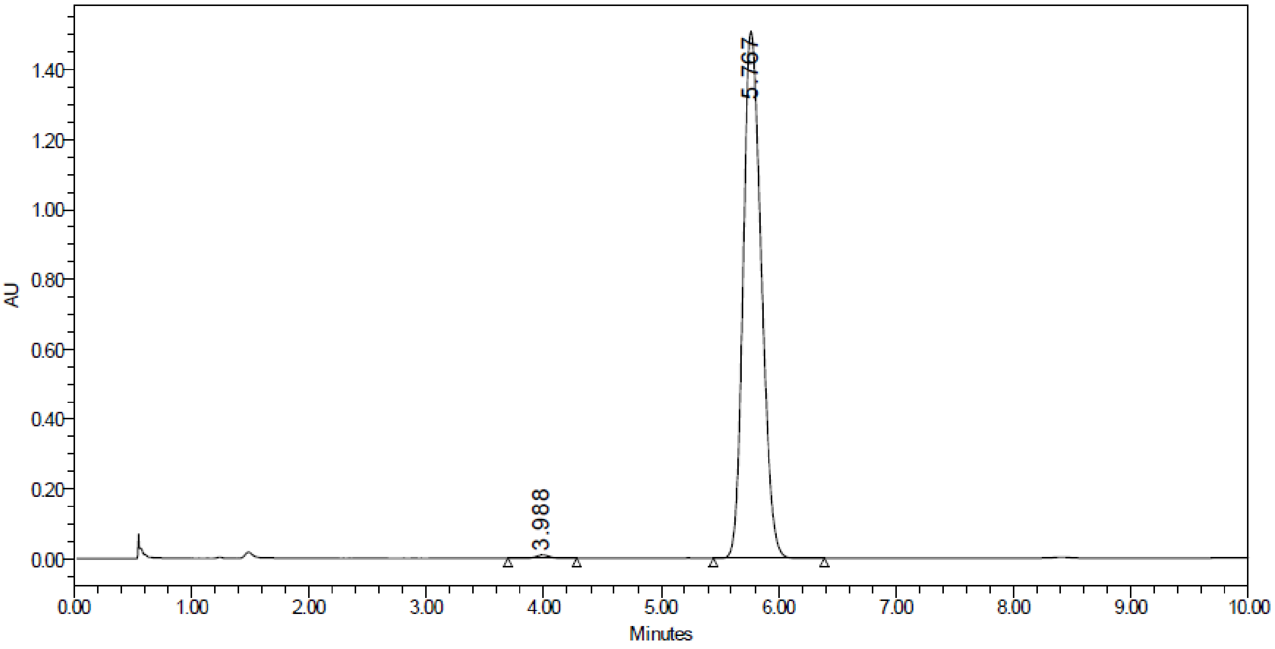

	Retention Time (min)	% Area
1	3.988	0.43
2	5.767	99.57

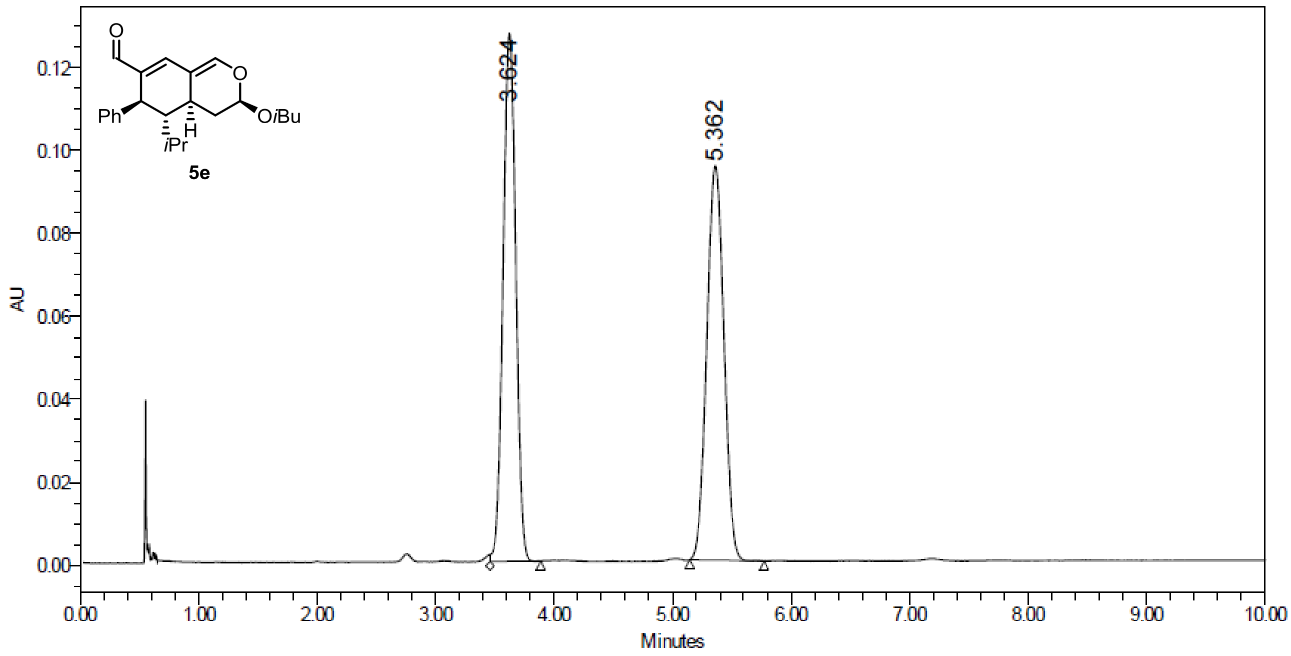

	Retention Time (min)	% Area
1	3.624	49.75
2	5.362	50.25

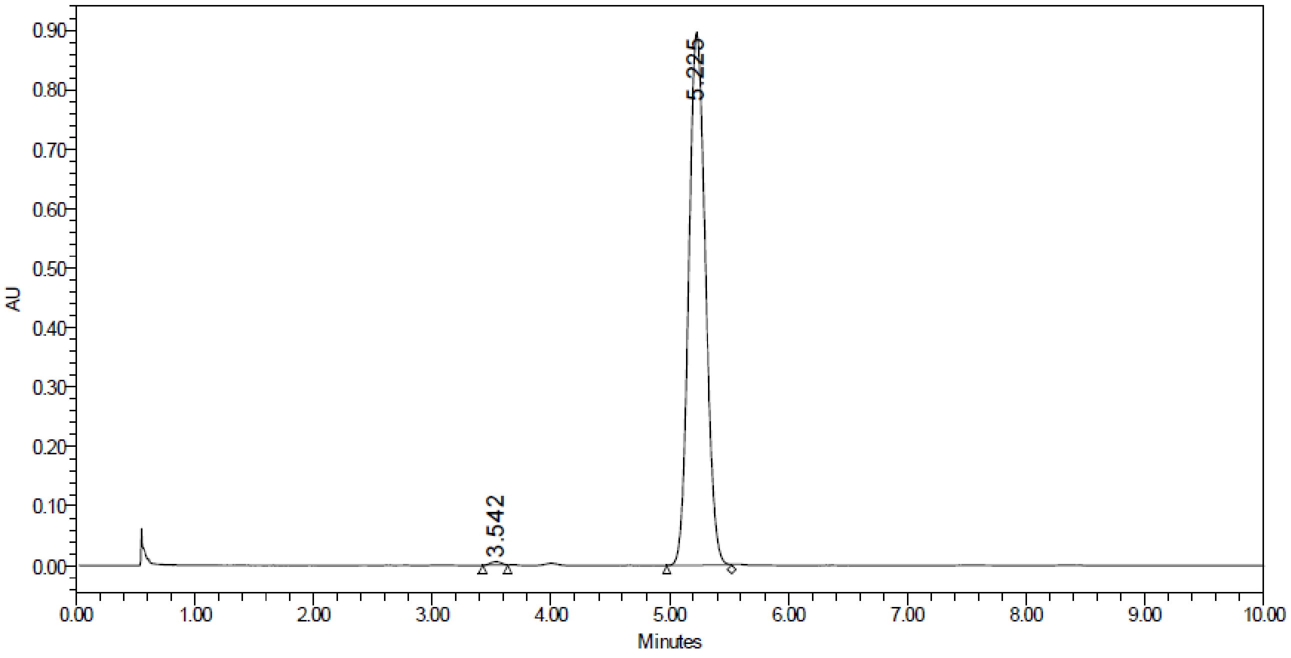

	Retention Time (min)	% Area
1	3.542	0.40
2	5.225	99.60

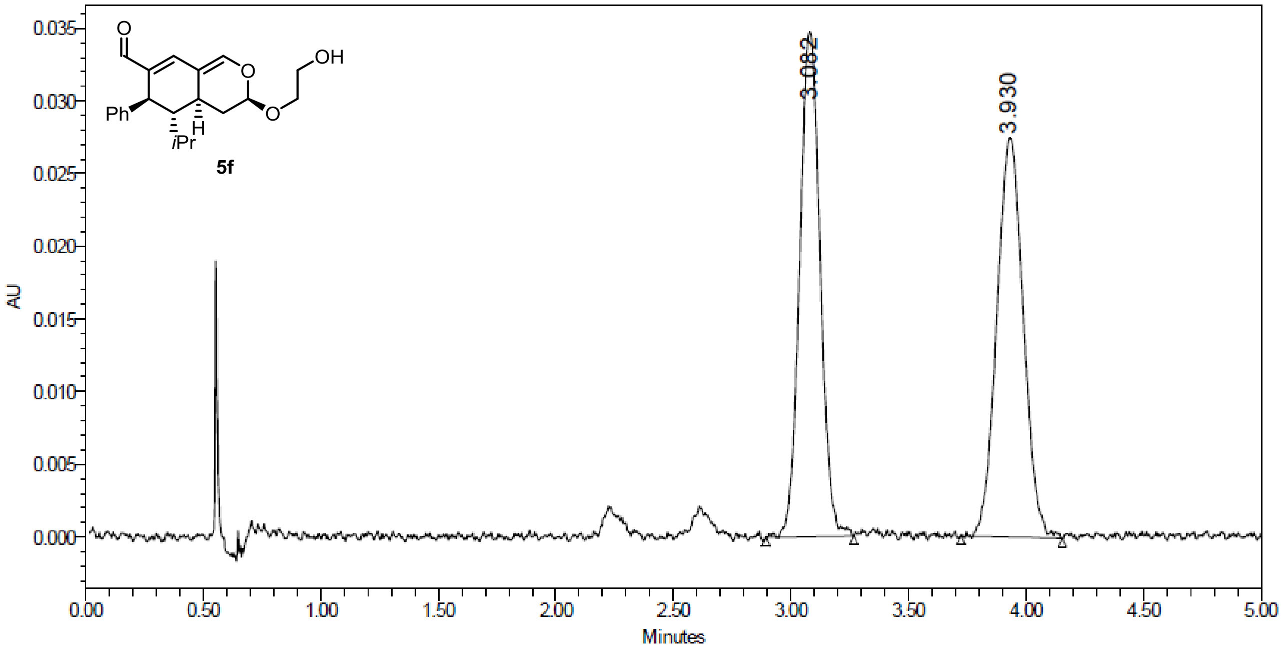

	Retention Time (min)	% Area
1	3.082	49.45
2	3.930	50.55

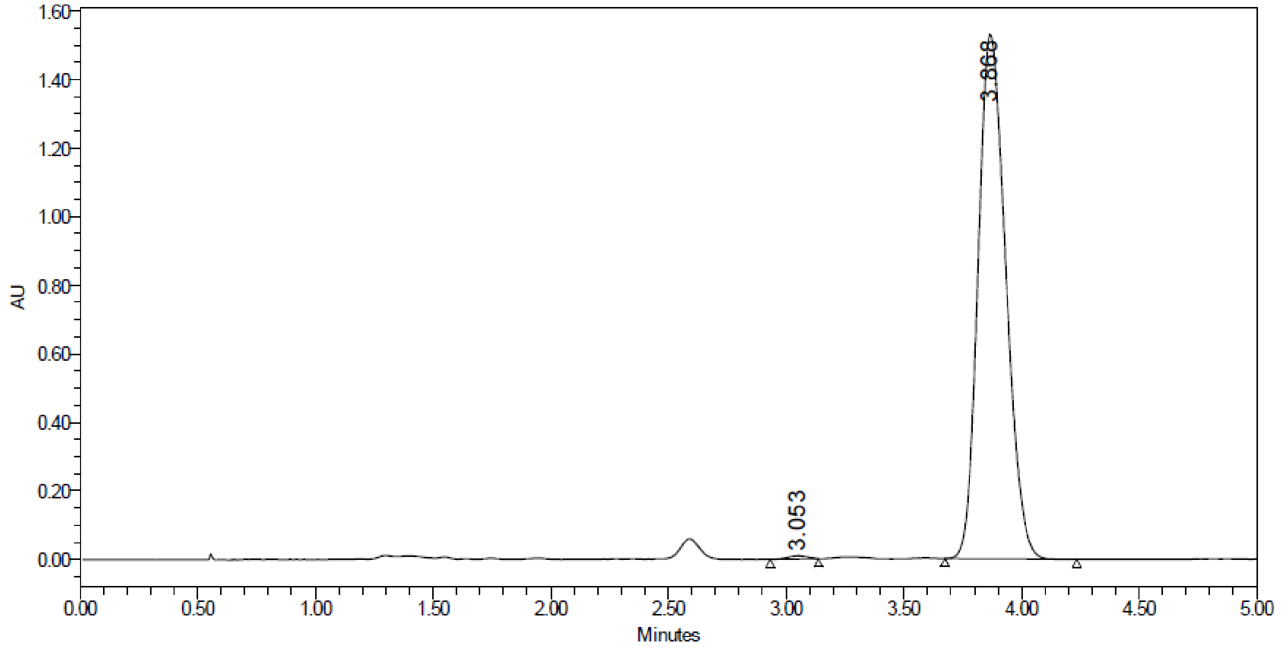

	Retention Time (min)	% Area
1	3.053	0.40
2	3.868	99.60

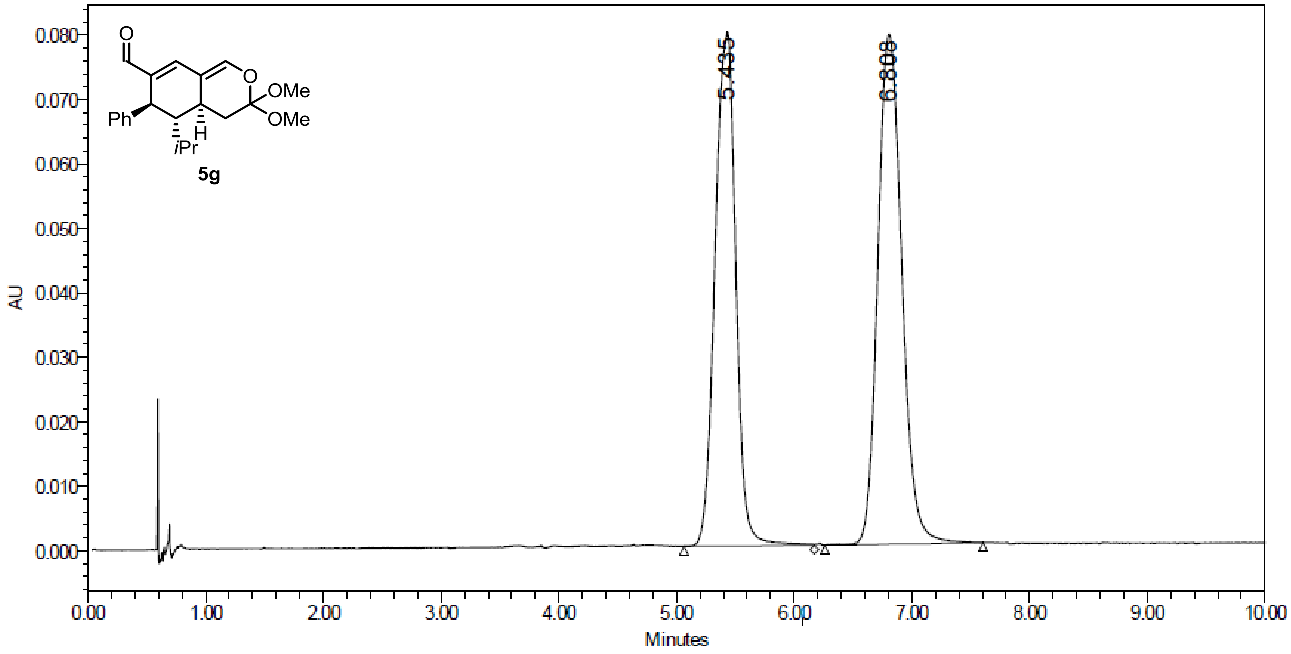

	Retention Time (min)	% Area
1	5.435	46.75
2	6.808	53.25

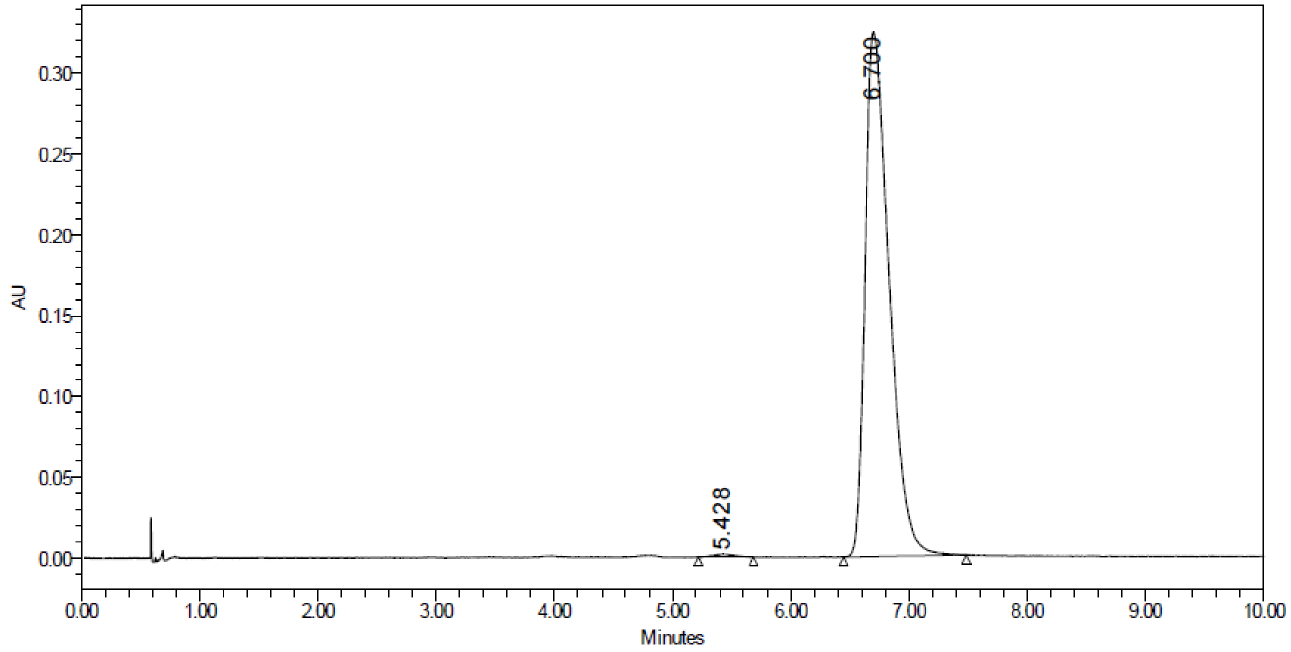

	Retention Time (min)	% Area
1	5.428	0.48
2	6.700	99.52

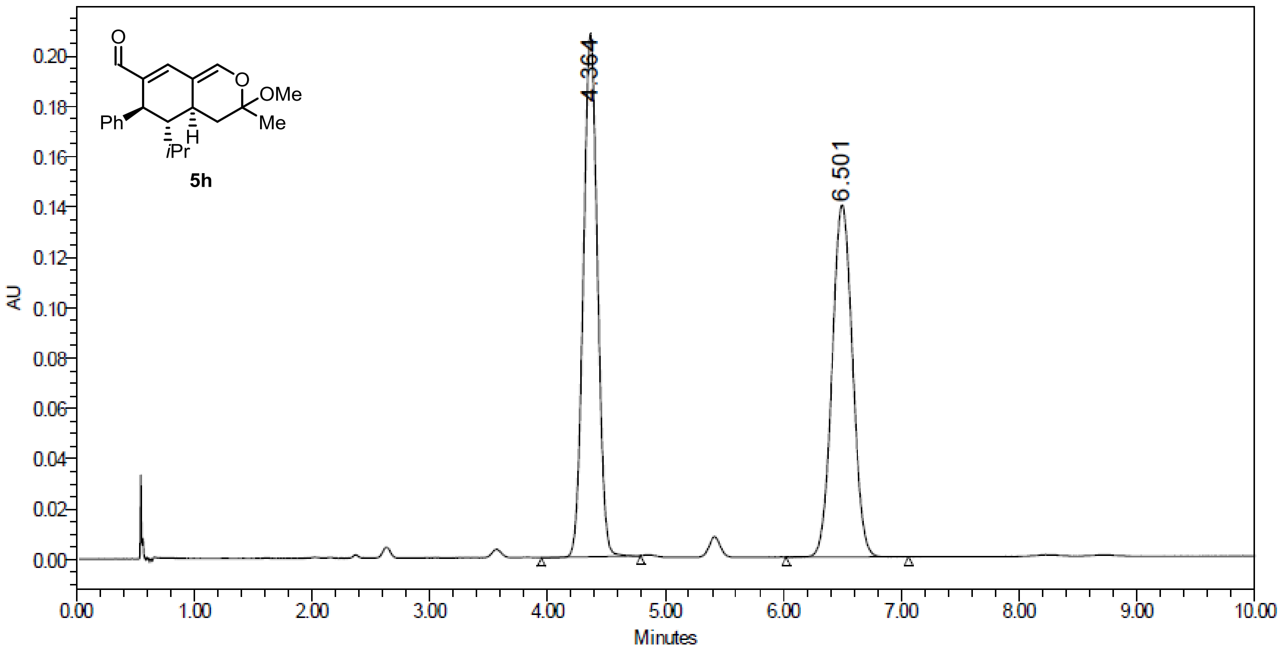

	Retention Time (min)	% Area
1	4.364	50.99
2	6.501	49.01

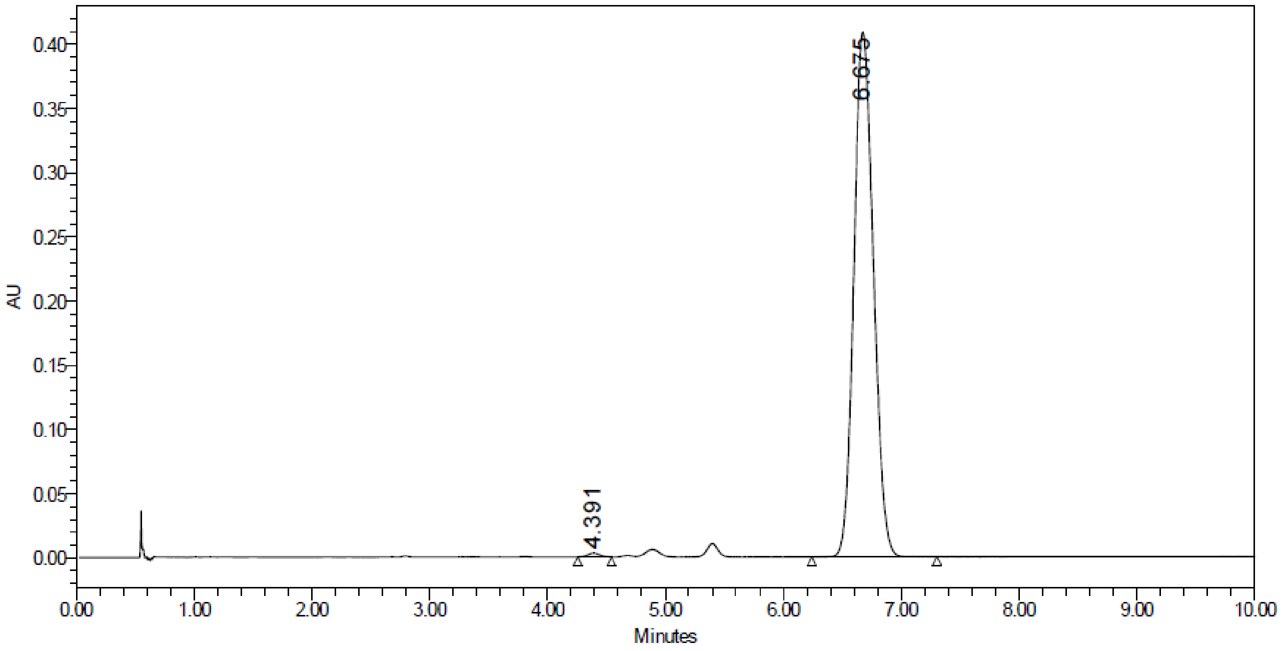

	Retention Time (min)	% Area
1	4.391	0.50
2	6.675	99.50

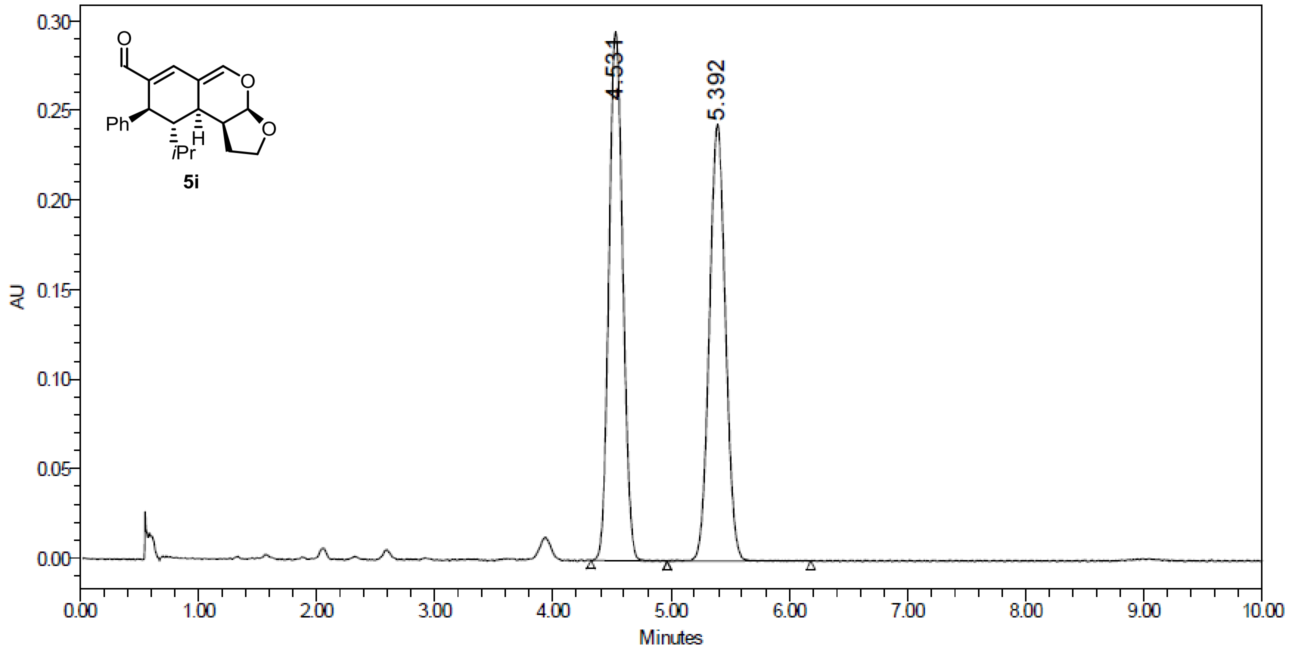

	Retention Time (min)	% Area
1	4.531	50.61
2	5.392	49.39

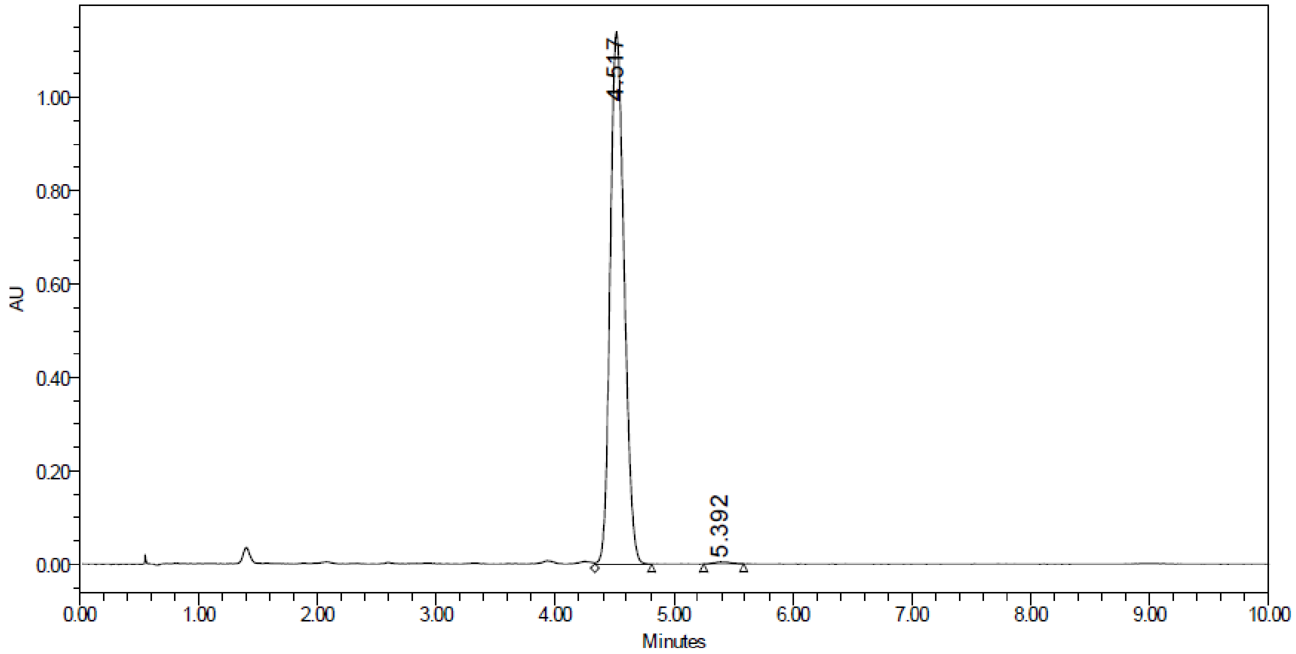

	Retention Time (min)	% Area
1	4.517	99.54
2	5.392	0.46

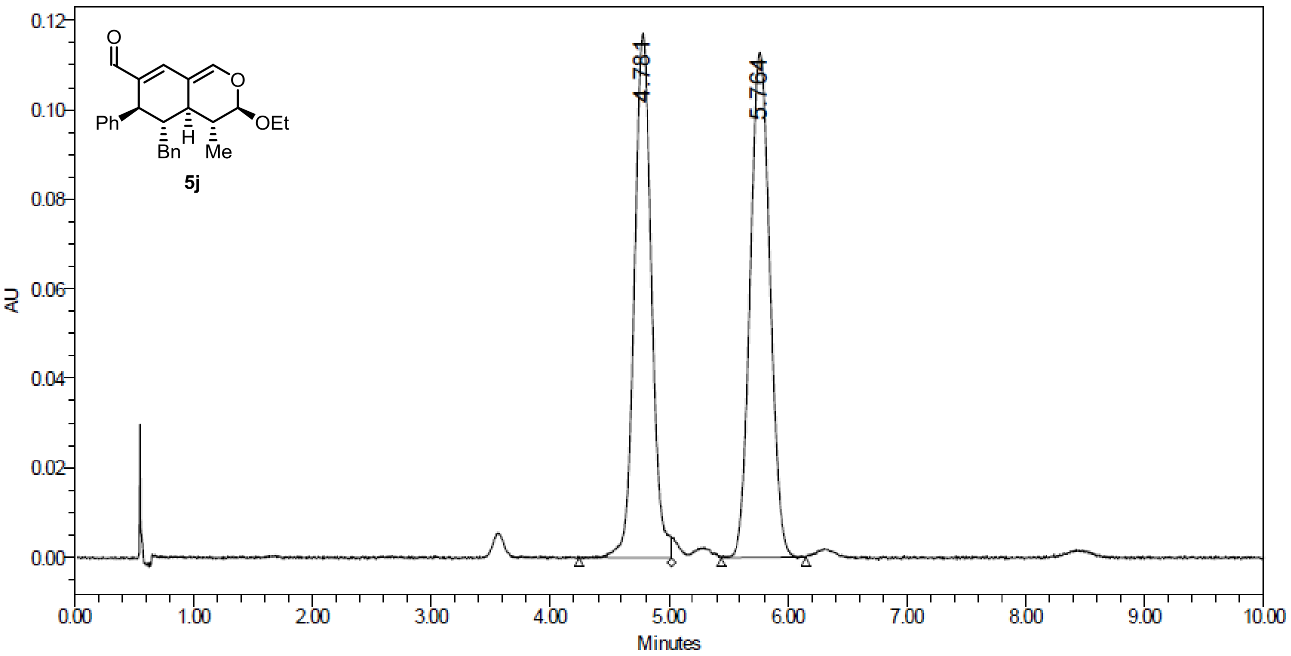

	Retention Time (min)	% Area
1	4.781	47.75
2	5.764	52.25

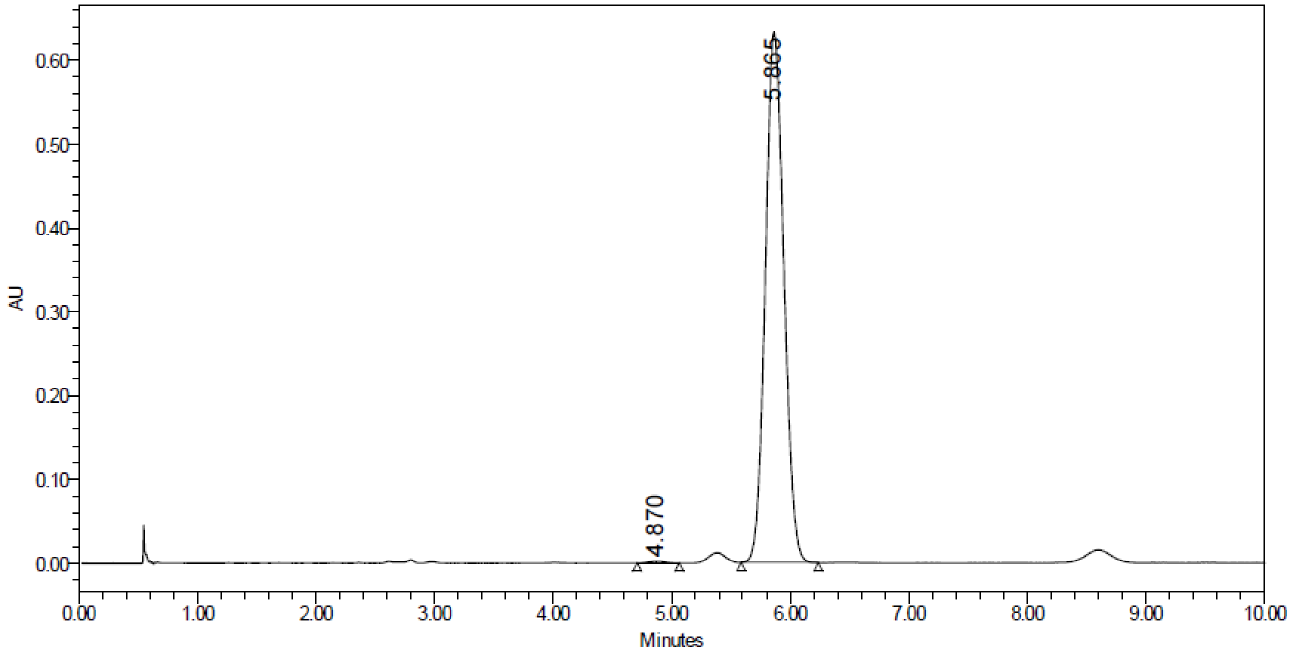

	Retention Time (min)	% Area
1	4.870	0.29
2	5.865	99.71

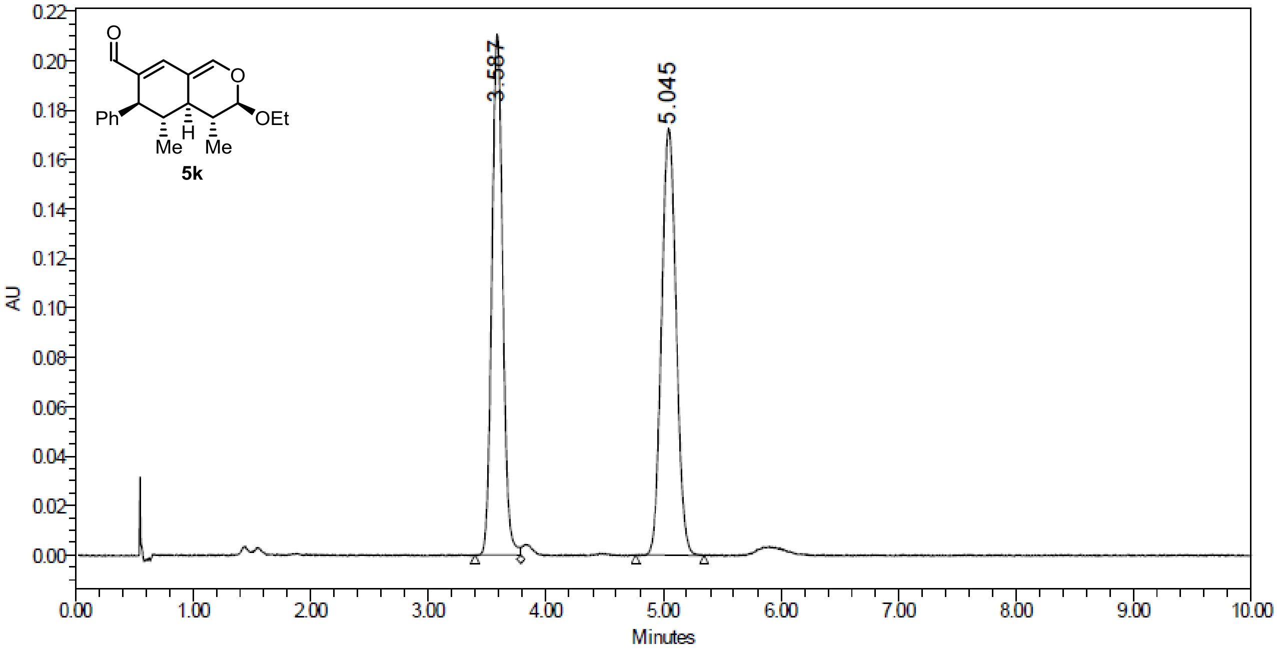

	Retention Time (min)	% Area
1	3.587	47.62
2	5.045	52.38

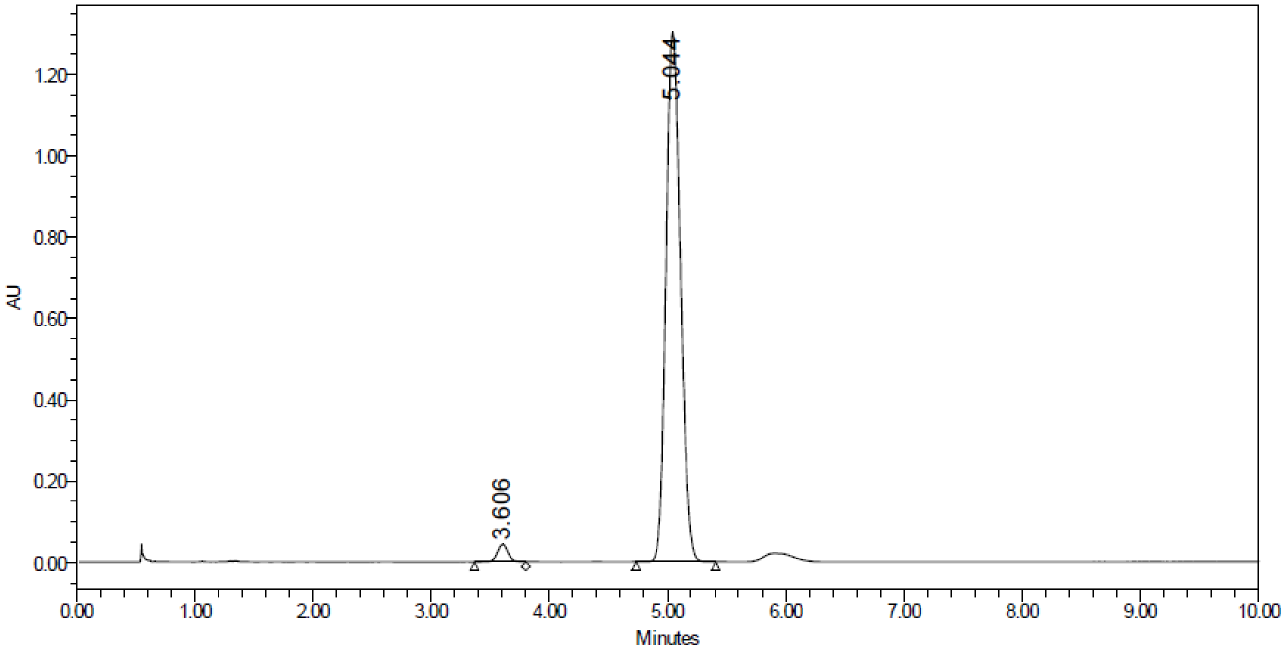

	Retention Time (min)	% Area
1	3.606	2.45
2	5.044	97.55

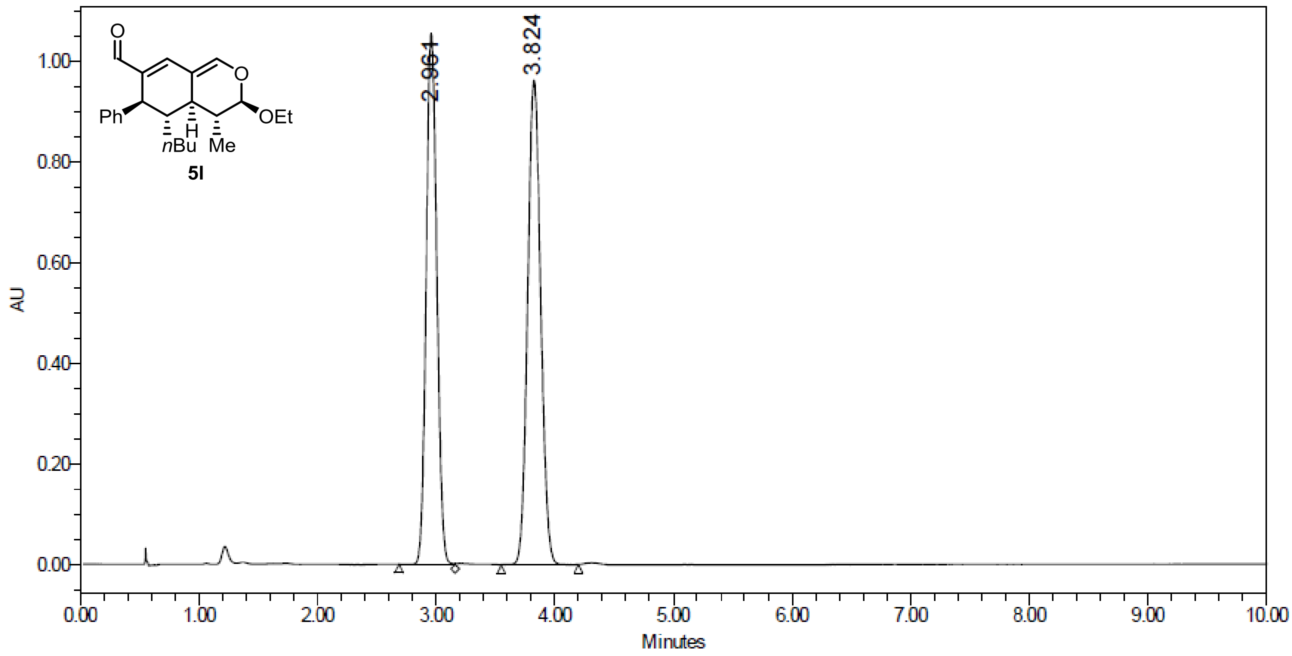

	Retention Time (min)	% Area
1	2.961	47.10
2	3.824	52.90

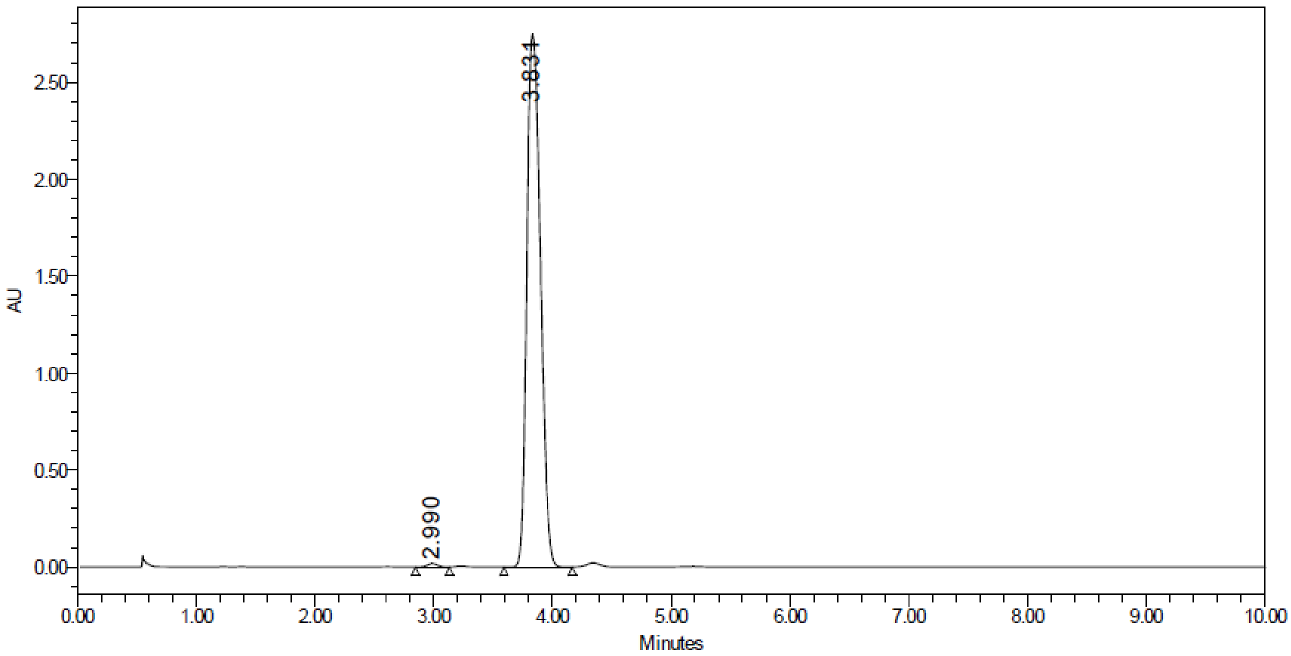

	Retention Time (min)	% Area
1	2.990	0.46
2	3.831	99.54

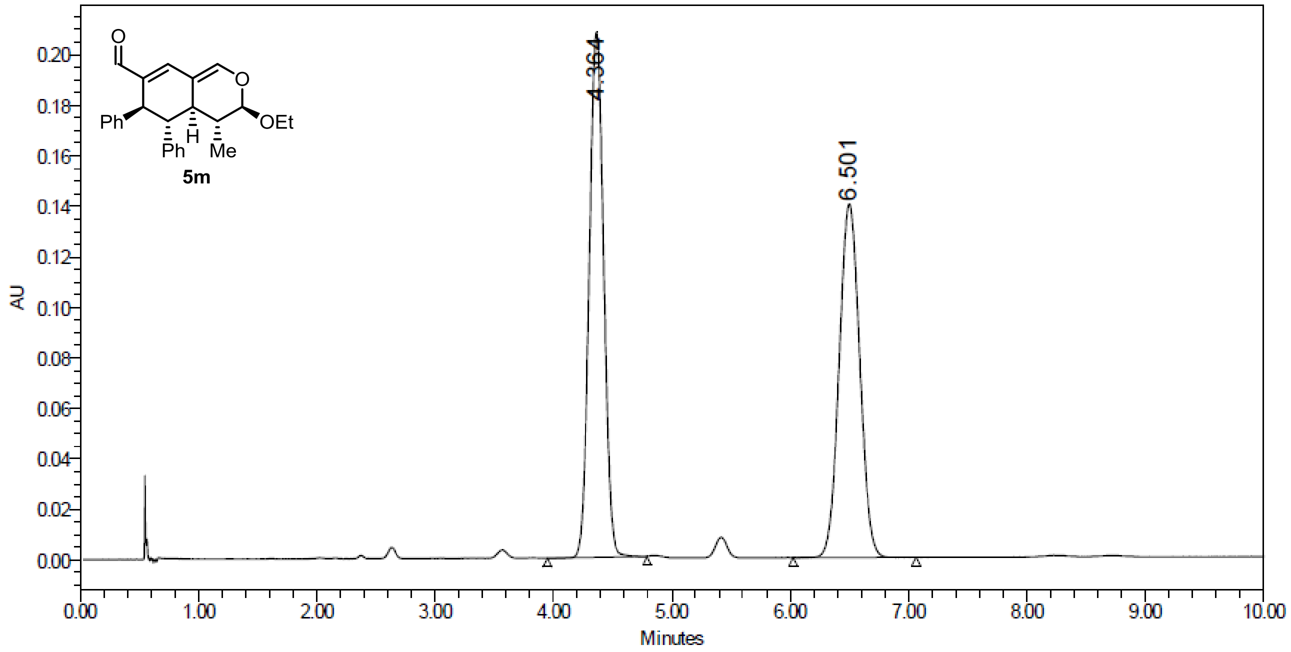

	Retention Time (min)	% Area
1	4.364	50.99
2	6.501	49.01

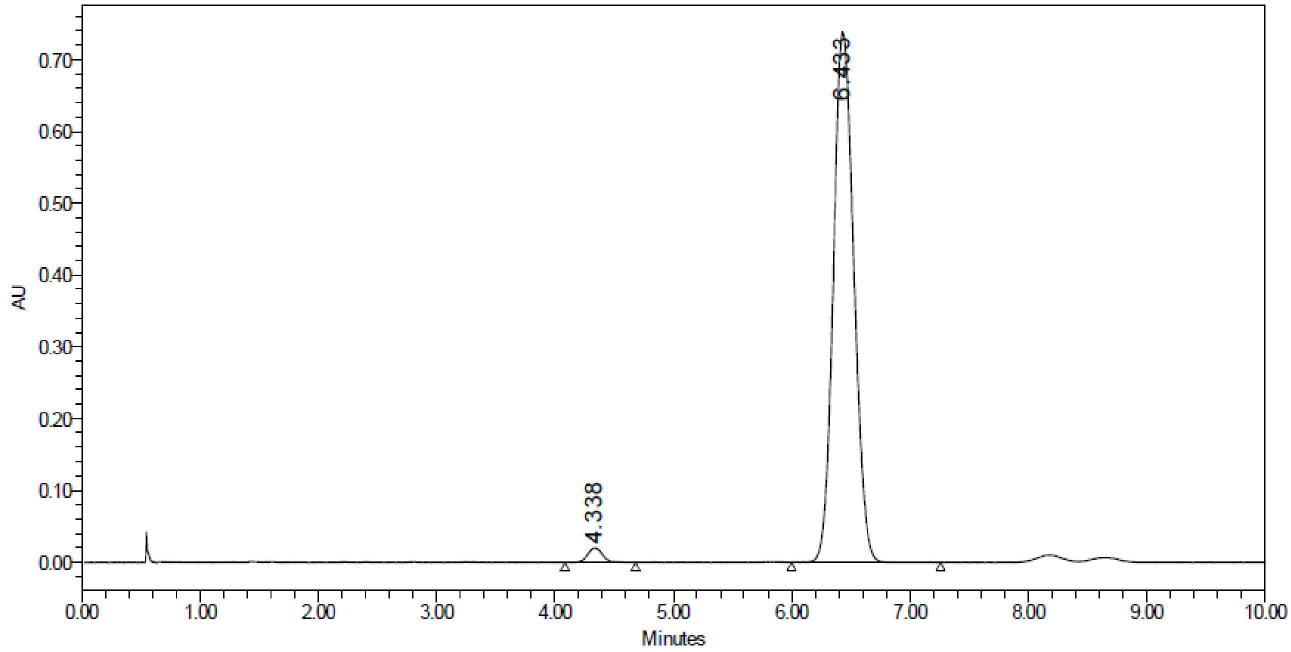

	Retention Time (min)	% Area
1	4.338	1.89
2	6.433	98.11

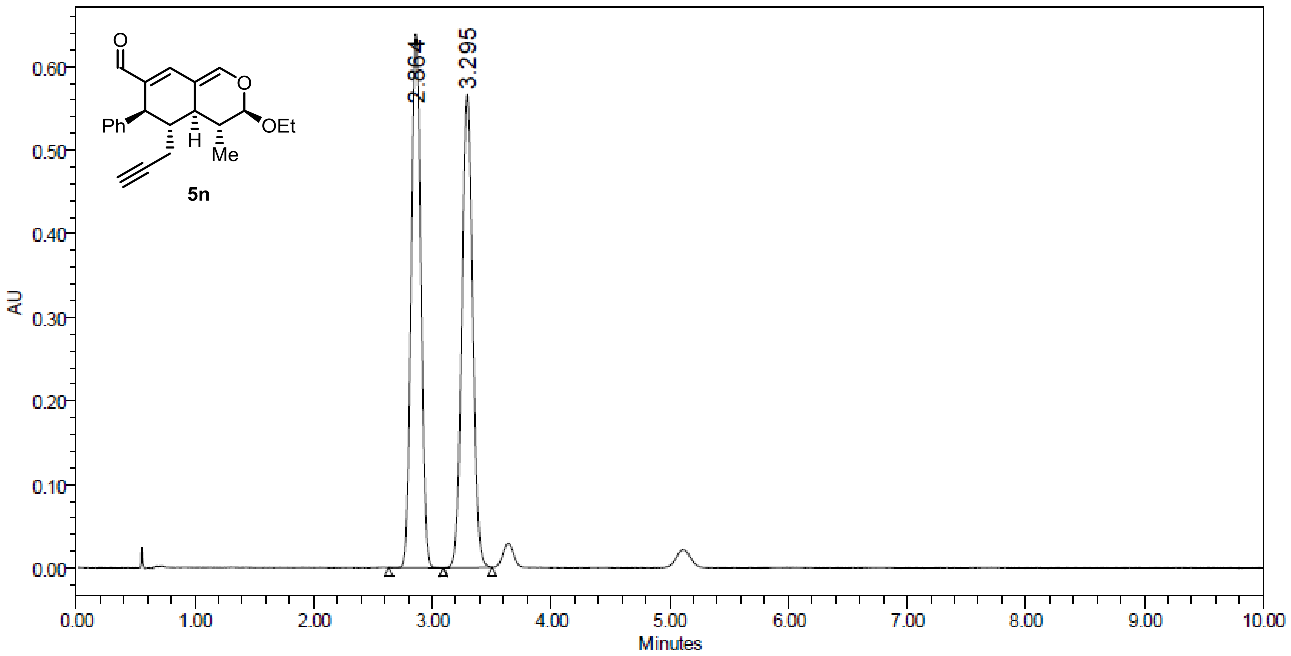

	Retention Time (min)	% Area
1	2.864	49.88
2	3.295	50.12

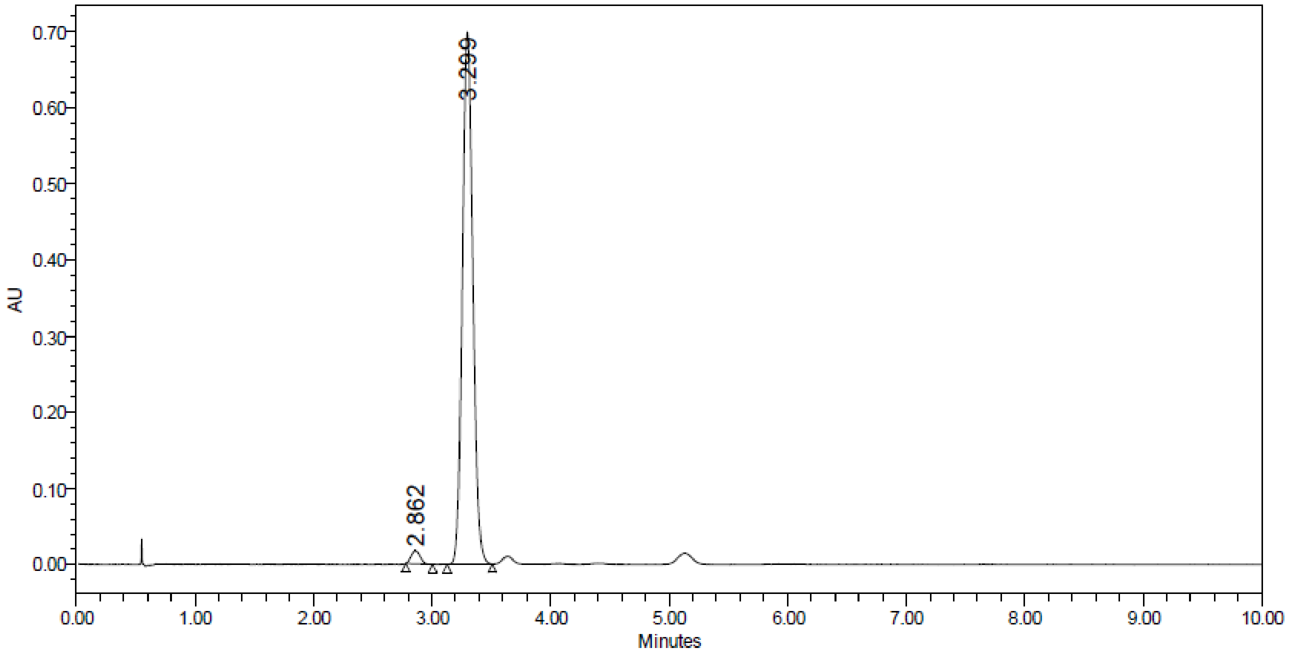

	Retention Time (min)	% Area
1	2.862	1.93
2	3.299	98.07

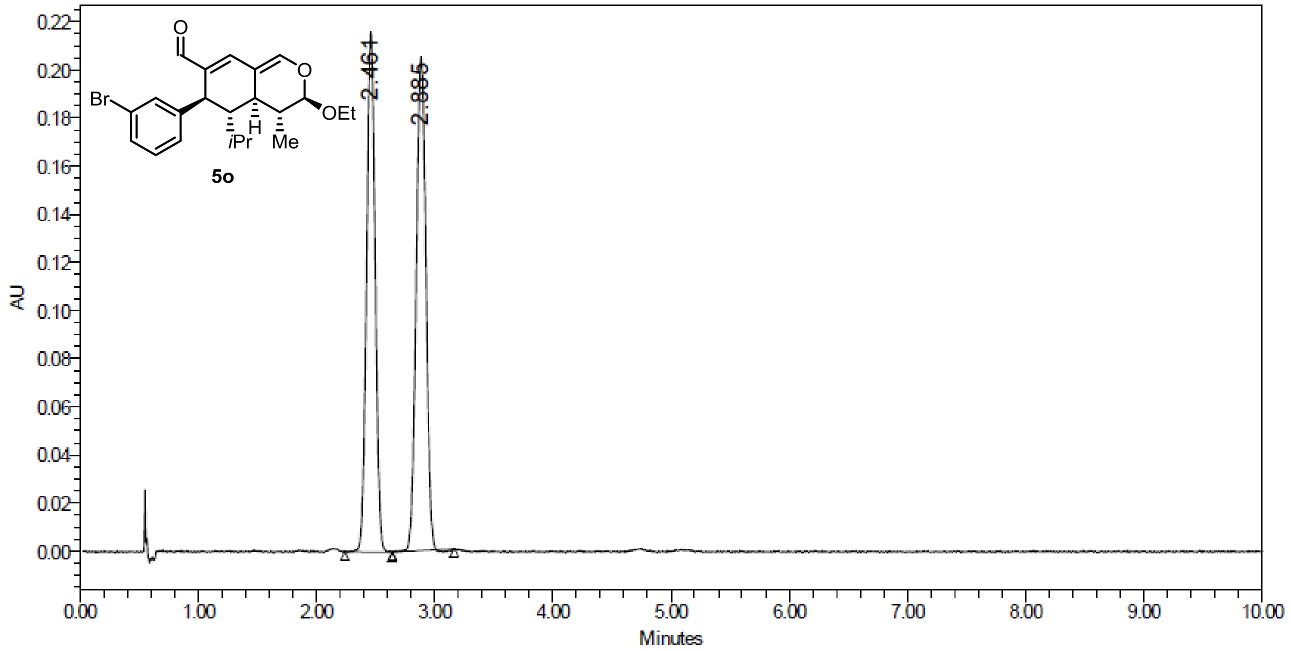

	Retention Time (min)	% Area
1	2.461	48.99
2	2.885	51.01

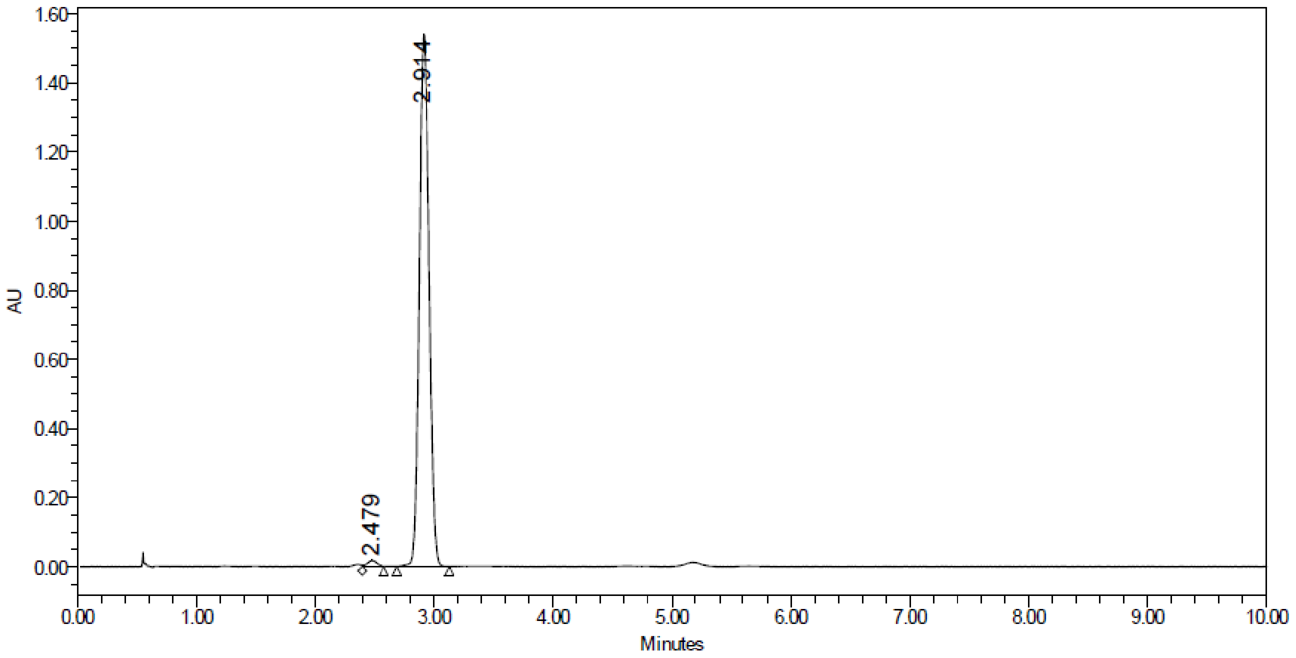

	Retention Time (min)	% Area
1	2.479	1.12
2	2.914	98.88

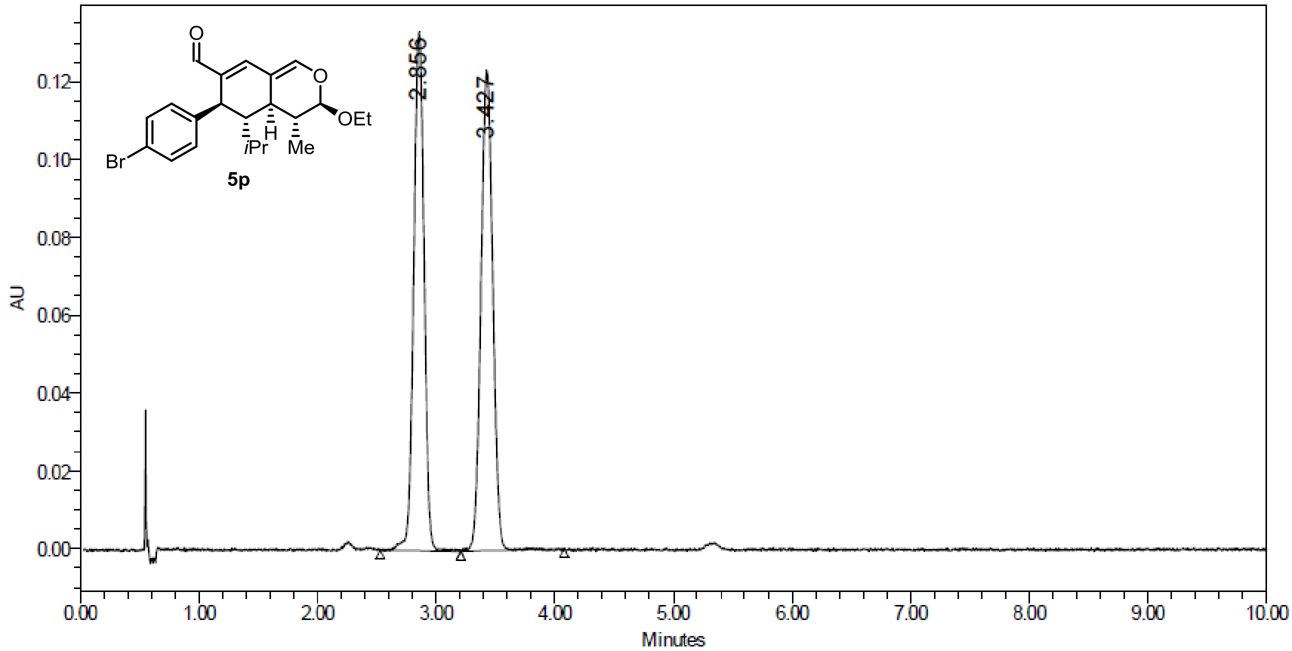

	Retention Time (min)	% Area
1	2.856	48.99
2	3.427	51.01

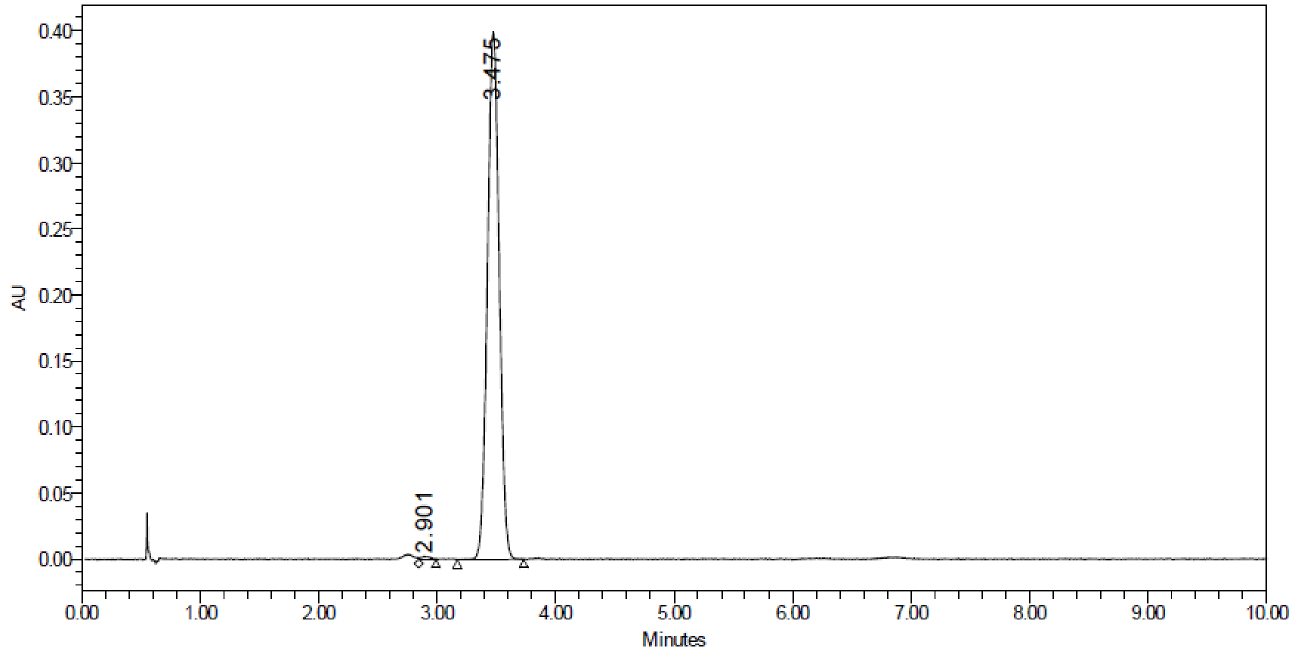

	Retention Time (min)	% Area
1	2.901	0.36
2	3.475	99.64

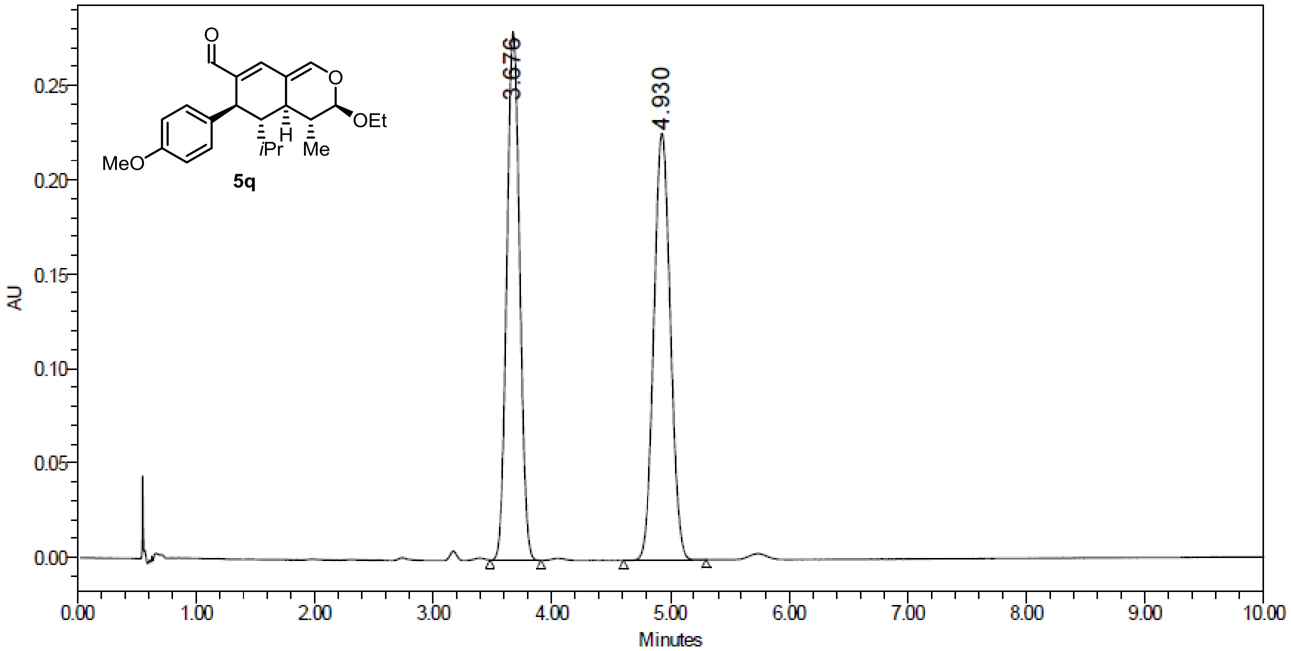

	Retention Time (min)	% Area
1	3.676	49.08
2	4.930	50.92

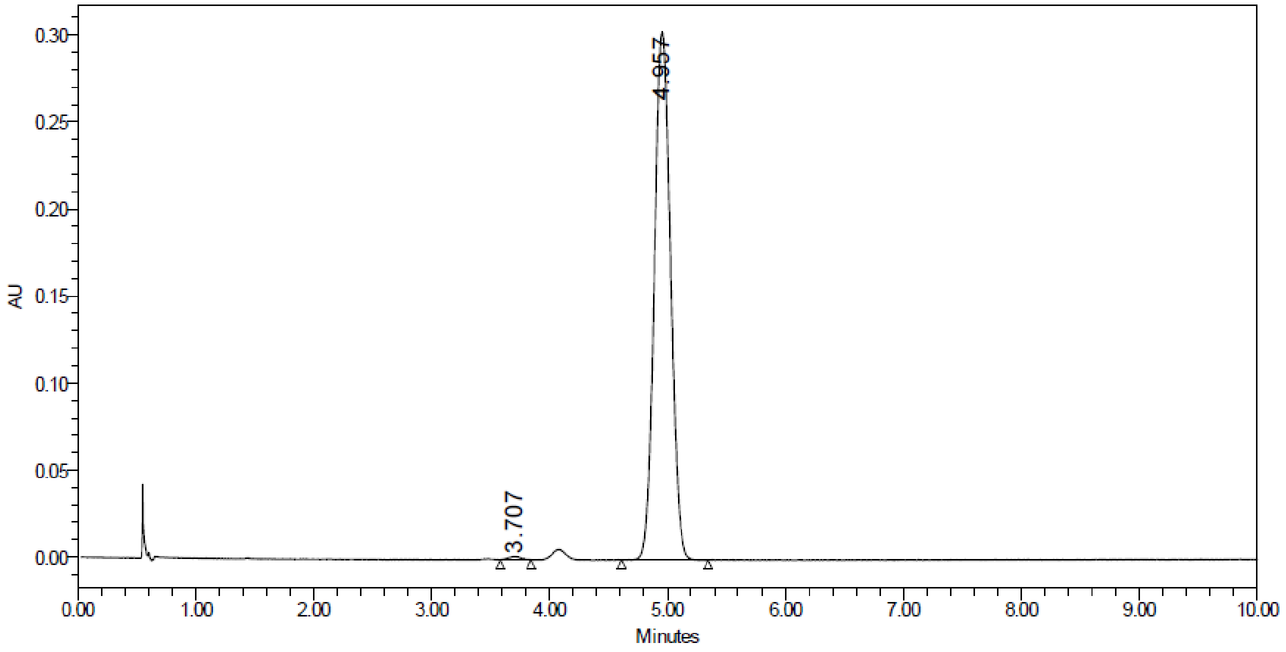

	Retention Time (min)	% Area
1	3.707	0.45
2	4.957	99.55

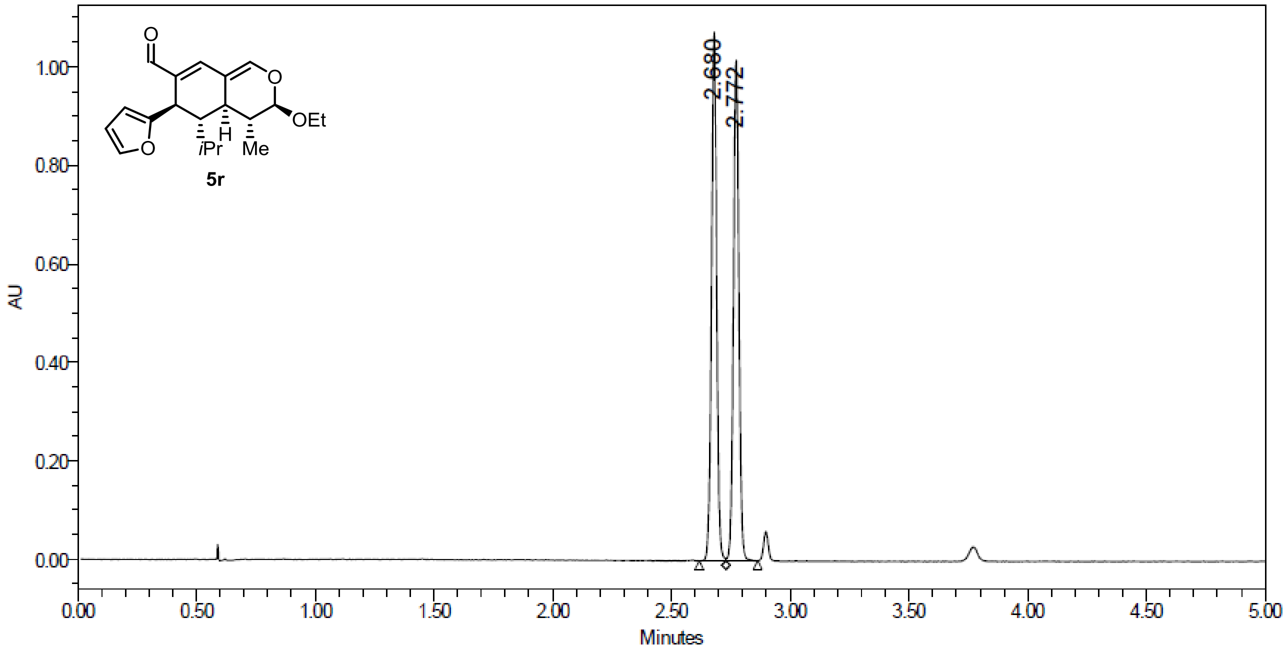

	Retention Time (min)	% Area
1	2.680	51.50
2	2.772	48.50

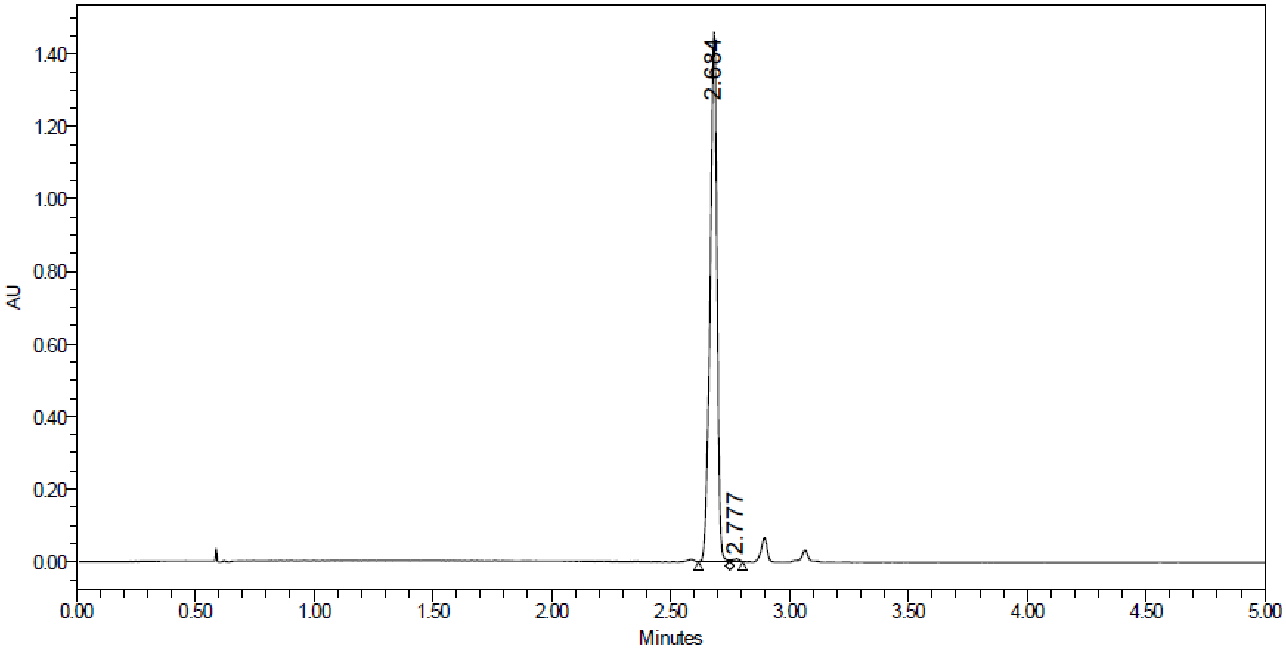

	Retention Time (min)	% Area
1	2.684	99.48
2	2.777	0.52

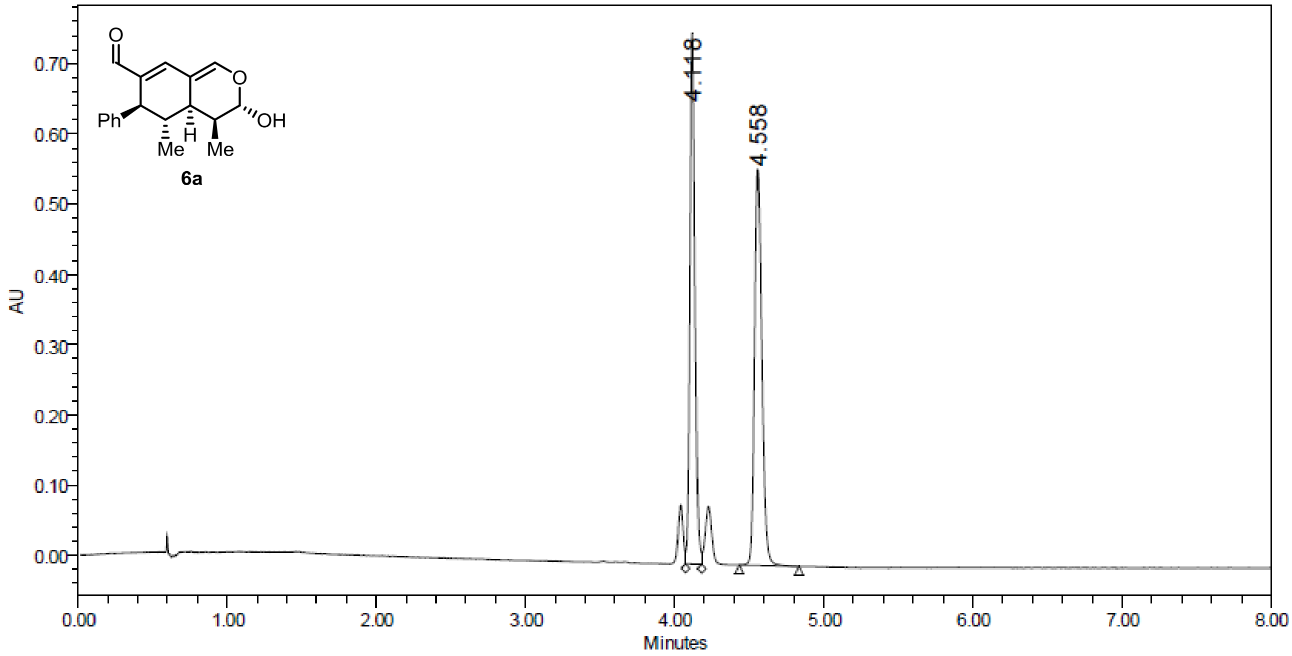

	Retention Time (min)	% Area
1	4.118	48.67
2	4.558	51.33

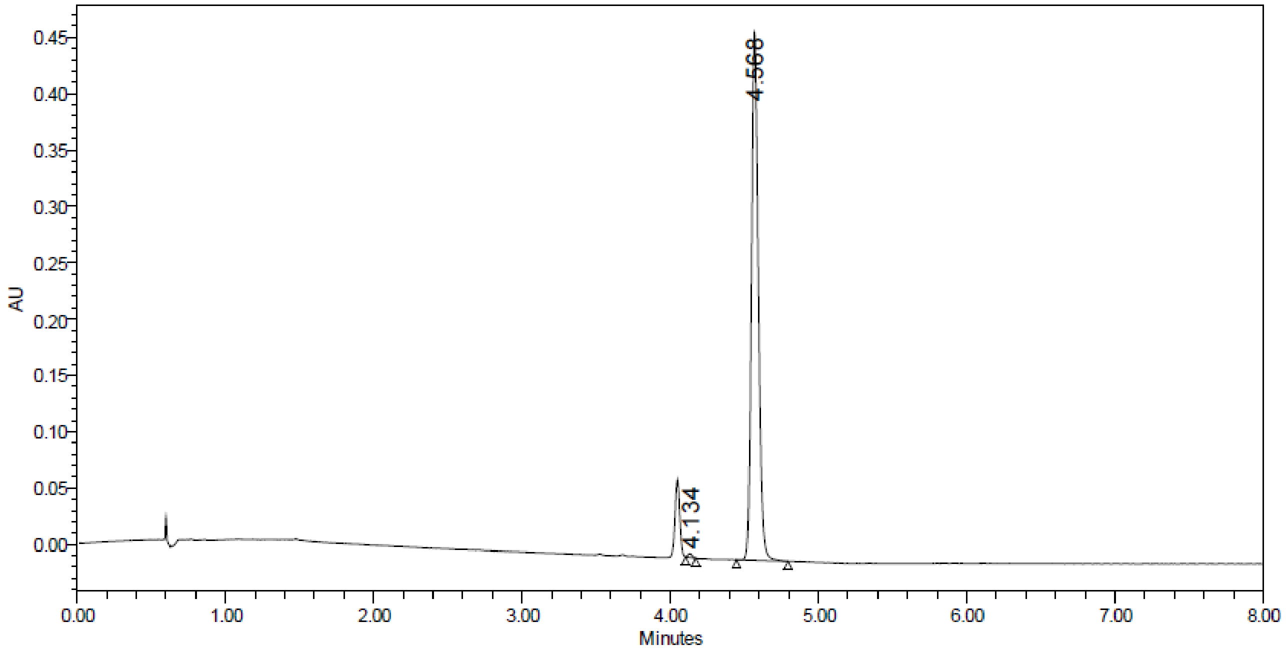

	Retention Time (min)	% Area
1	4.134	0.43
2	4.568	99.57

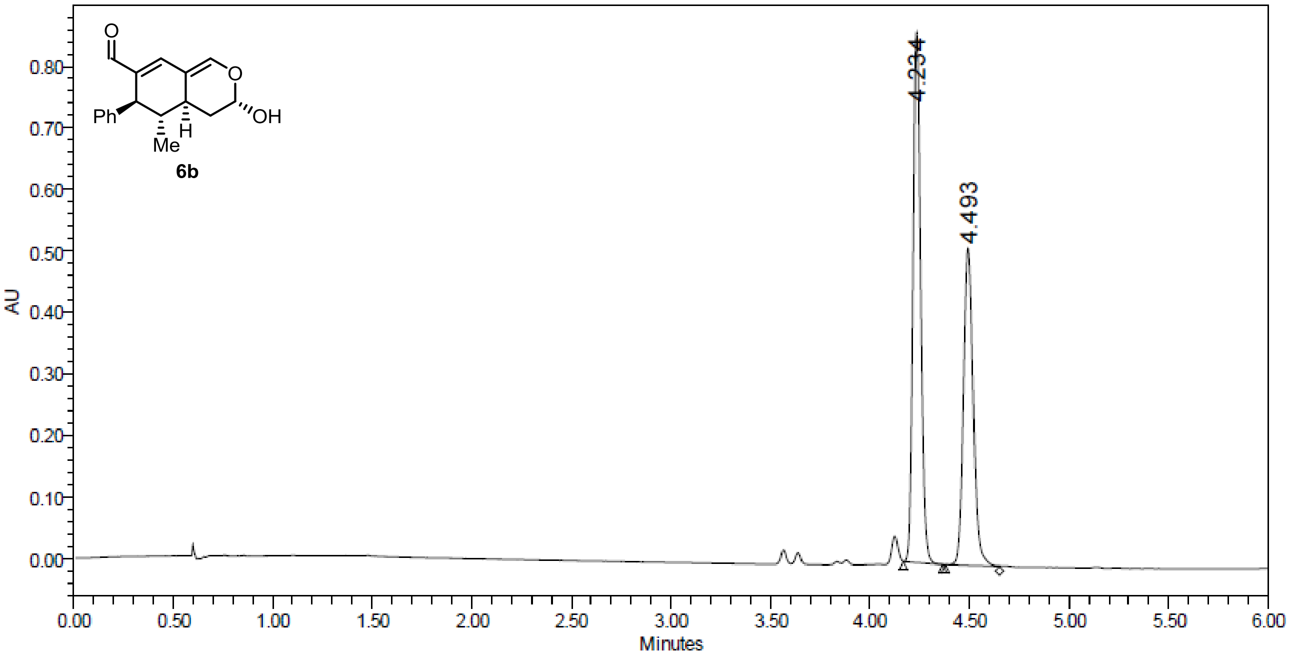

	Retention Time (min)	% Area
1	4.234	55.91
2	4.493	44.09

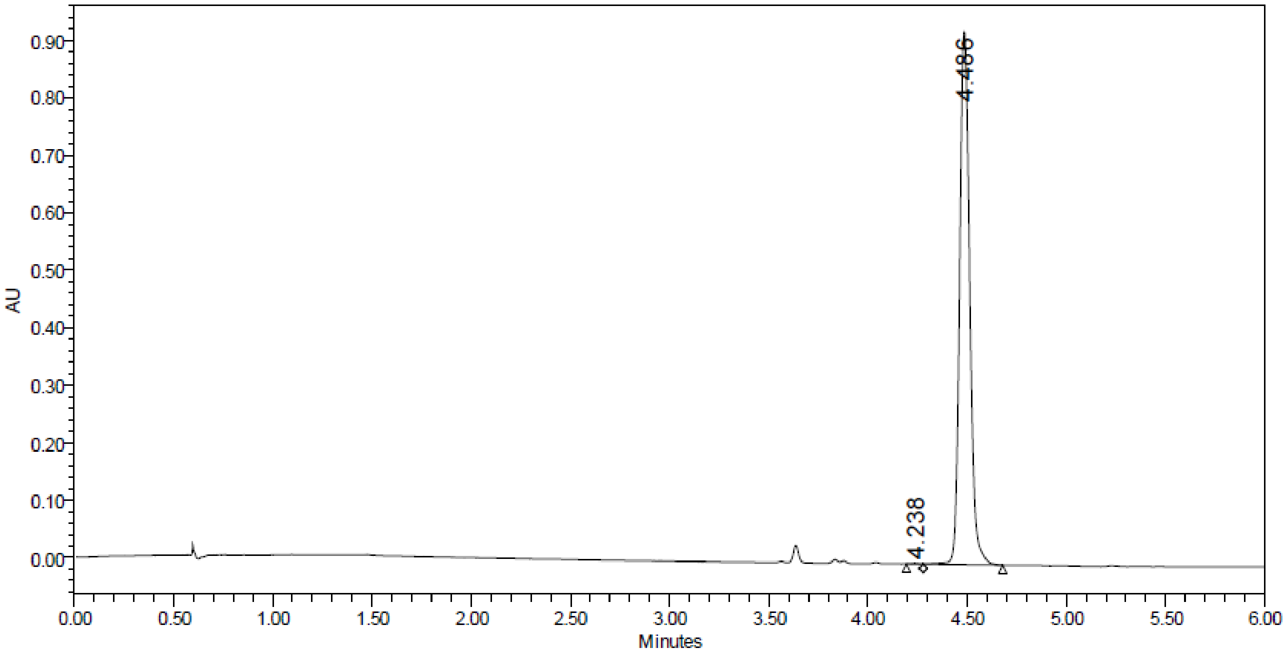

	Retention Time (min)	% Area
1	4.238	0.15
2	4.486	99.85

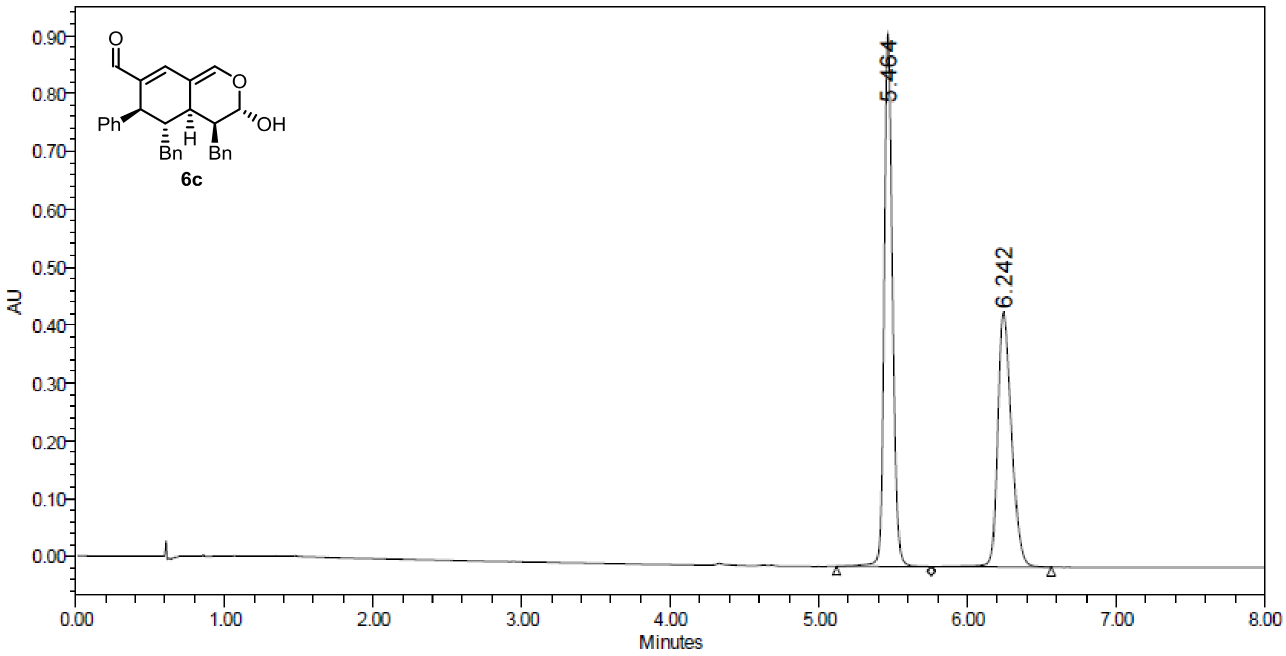

	Retention Time (min)	% Area
1	5.464	56.10
2	6.242	43.90

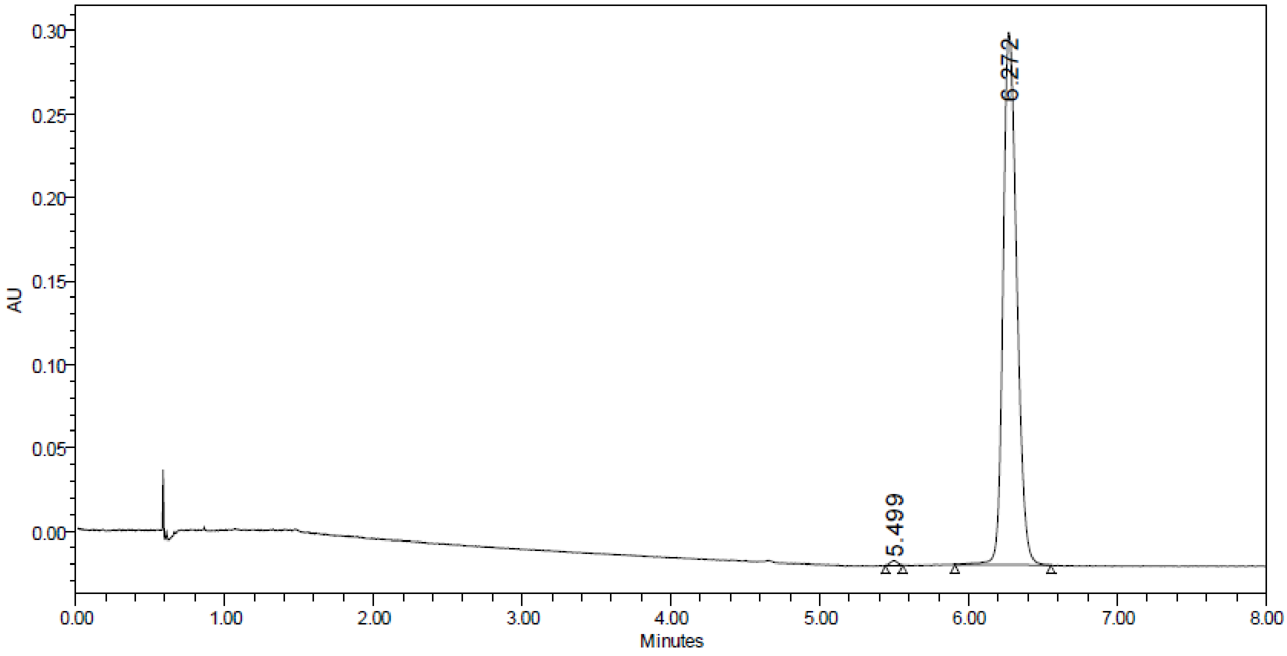

	Retention Time (min)	% Area
1	5.499	0.42
2	6.272	99.58

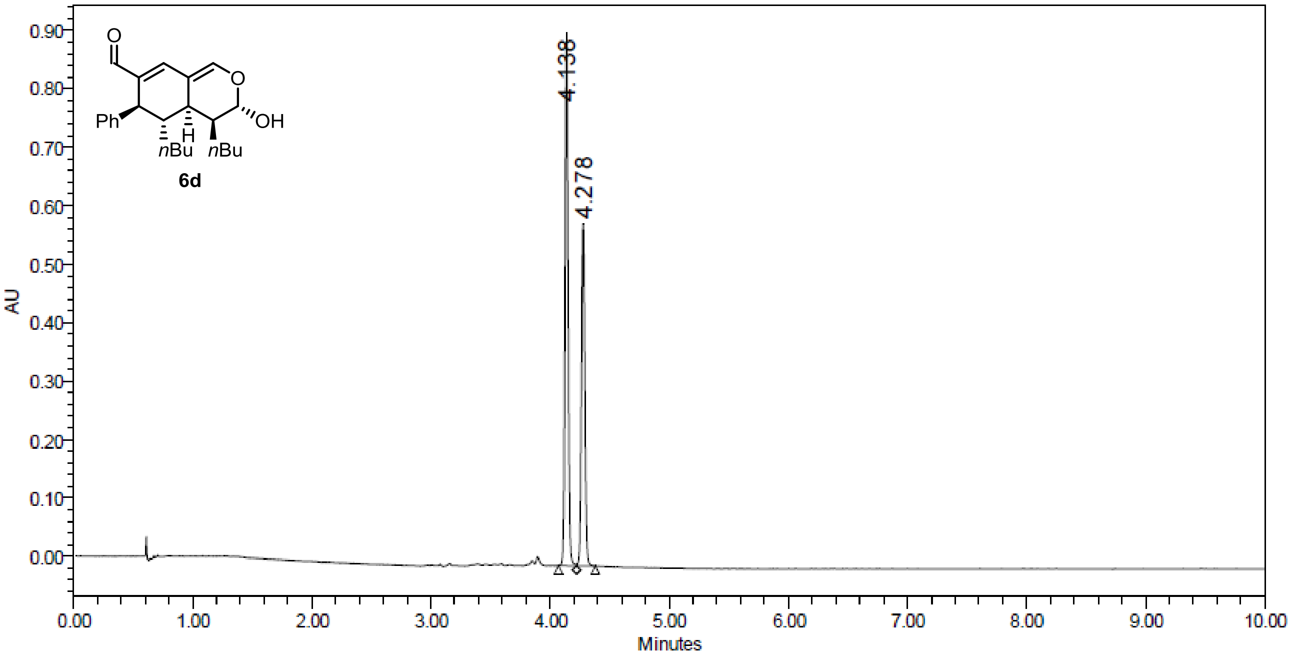

	Retention Time (min)	% Area
1	4.138	58.74
2	4.278	41.26

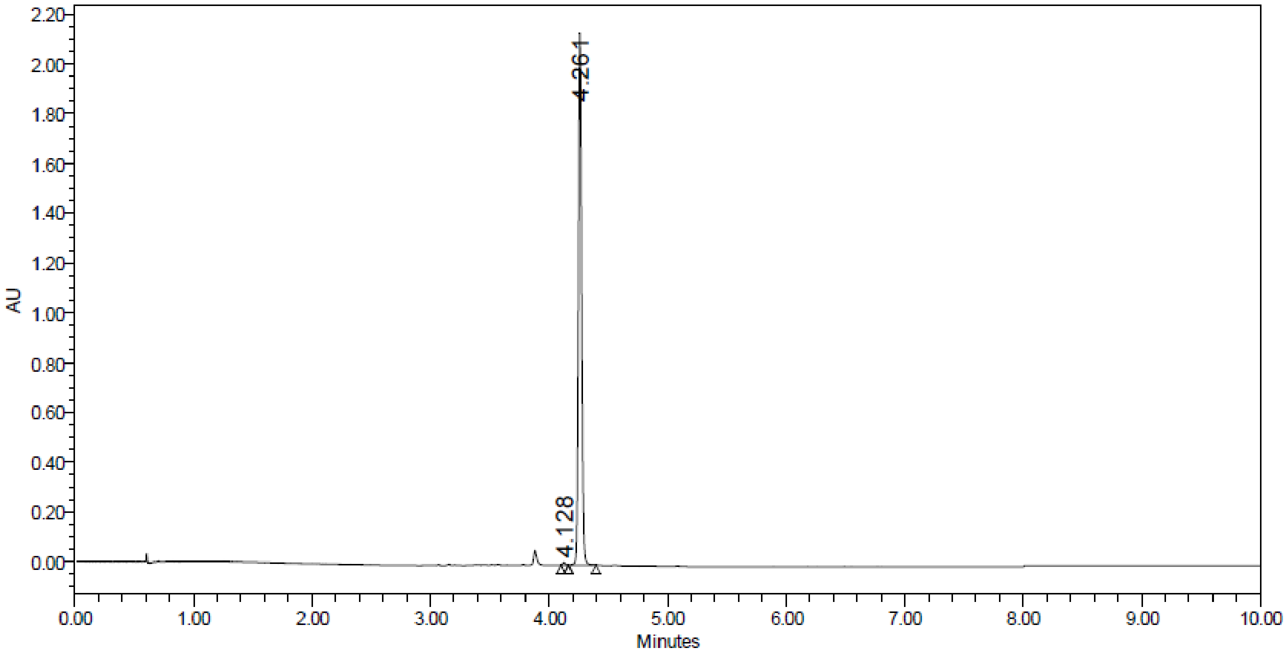

	Retention Time (min)	% Area
1	4.128	0.39
2	4.261	99.61

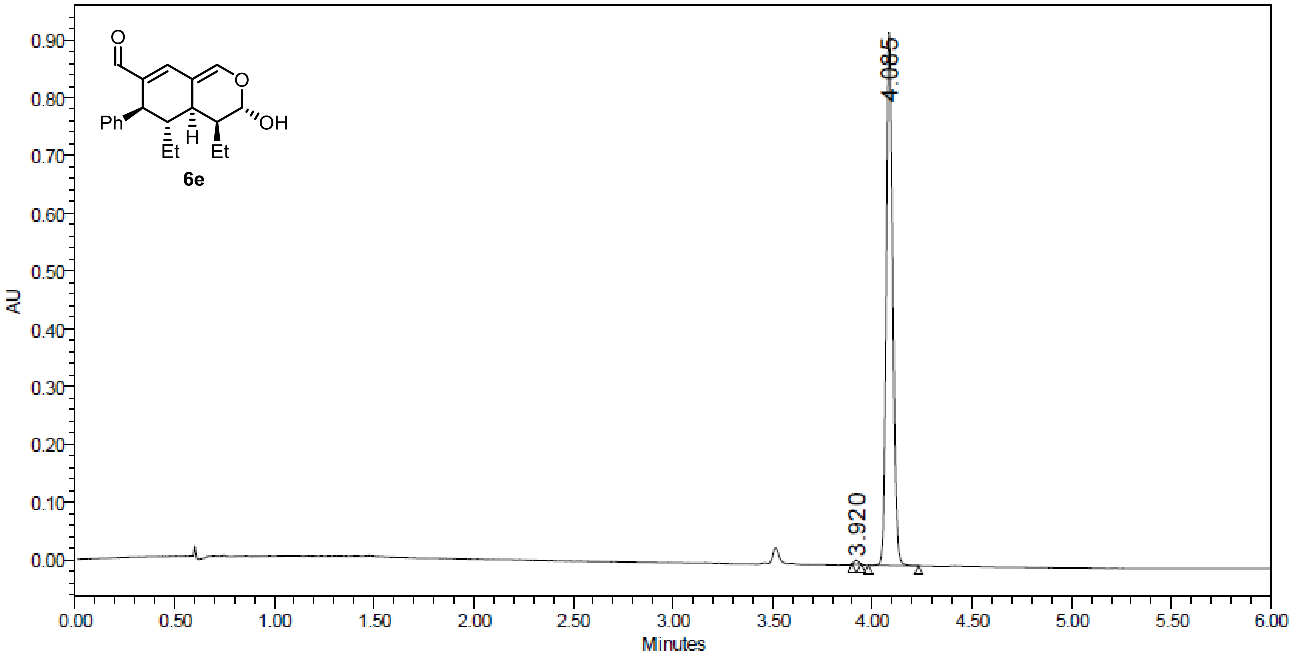

	Retention Time (min)	% Area
1	3.920	0.48
2	4.085	99.52

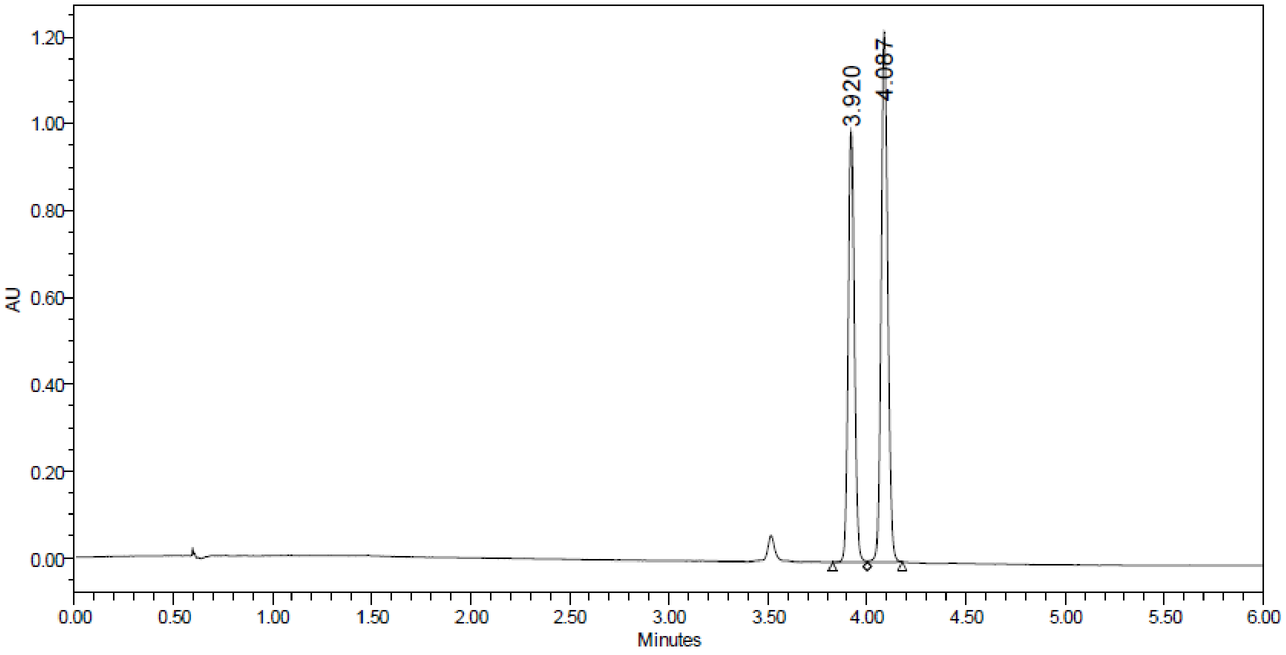

	Retention Time (min)	% Area
1	3.920	42.19
2	4.087	57.81
